# Sulfoximine *N*‑Functionalization with *N*‑Fluorobenzenesulfonamide

**DOI:** 10.1021/acs.joc.5c02077

**Published:** 2025-10-29

**Authors:** Žan Testen, Črtomir Podlipnik, Marjan Jereb

**Affiliations:** 112794 University of Ljubljana, Faculty of Chemistry and Chemical Technology, Večna pot 113, 1000 Ljubljana, Slovenia

## Abstract

An operationally simple, convenient, mild, metal-free, and scalable transformation of sulfoximines with *N*-fluorobenzenesulfonamide is presented. A wide range of structurally different *N*H-sulfoximines were prepared and reacted with *N*-fluorobenzenesulfonamide in the presence of TMP (2,2,6,6-Tetramethylpiperidine) and in the environmentally friendly EtOAc to obtain the desired products, mostly in yields above 75%. The method also supports various amine, hydrazide, phenol, and amino acid substrates as well as scale-up to gram reactions with minimal to no modification of the process. The products formed from the sulfoximines were structurally analyzed by NMR and X-ray crystallography and investigated for their stability and further reactivity as substrates in Suzuki-Miyaura coupling, methylation, and bromination reactions. In addition, DFT calculations were carried out with regard to the energy profile and mechanism of the reaction.

## Graphical abstract

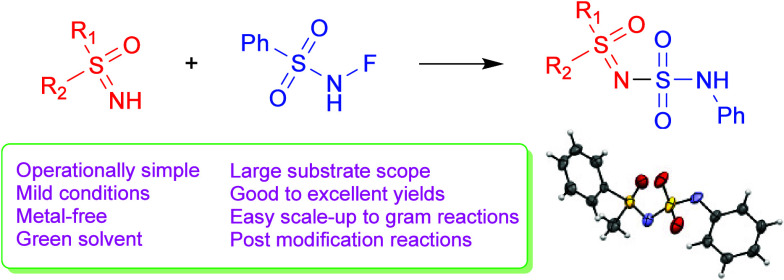

## Introduction

Research in the field of sulfoximines and their derivatives has experienced a significant upswing since their biologically active role has been more thoroughly explored.^1–6^ With newer and safer synthetic methods eliminating the use of sodium azide to obtain sulfoximines^7^ and while being both sulfonamide and sulfone bioisosteres, the number of reported functionalizations as well as clinical trials is increasing. Some biologically active sulfoximines are shown in Figure 1.^8,9^

**Figure 1:**
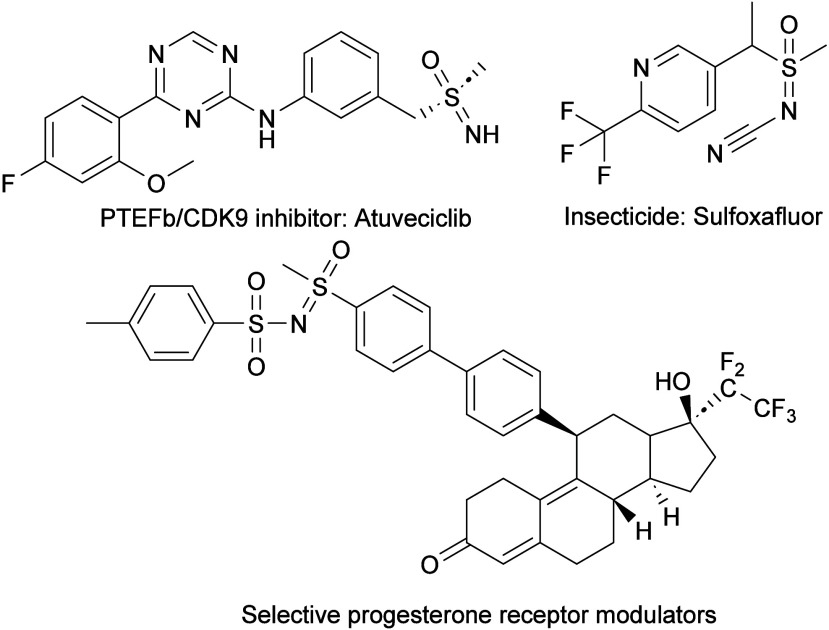
Selection of biologically active sulfoximines.

New ways to obtain sulfoximines and their analogs are one of the hot topics in chemistry.^10,11^ Recently, a late-stage and biomolecule-compatible imination with DPPH (2,2-Diphenyl-1-picrylhydrazyl) followed by oxidation with *m*-CPBA (*meta*-Chloroperoxybenzoic acid) has been reported.^12^ New reactions related to *N*-functionalization of sulfoximines include *N*-benzylation of sulfoximines with TBHP (*tert*-Butyl hydroperoxide) and catalytic amounts of iodine,^13^ copper-catalyzed *N*-alkylations with alkyl diacyl peroxides under visible light,^14^ and several electrochemical functionalizations forming *N*-alkyl/acyl^15,16^ and *N*-heteroaryl sulfoximines.^17^ Sulfonylhydrazones were also successfully attached to sulfoximines through sulfoximidoyl-based hypervalent iodine­(III) reagents.^18^ Sulfoximines could also be functionalized with dioxazolones, forming unsymmetric (sulfoxylidene)­ureas^19^ as well as complex cyclopentenyl scaffolds in a scandium-catalyzed variant of the aza-Piancatelli cyclization.^20^ An *N*-acylation and subsequent cyclization of 2-hydroxyaryl sulfoximines using CDI (1,1′-Carbonyldiimidazole) yielded compounds with an in-ring incorporated sulfoximine moiety.^21^ Rh-catalyzed C–H bond activation and subsequent cyclization reactions with hypervalent iodonium ylides were also reported.^22,23^

Cross-coupling reactions with sulfoximines are also on the rise.^24^ Rhodium/diphosphine-catalyzed asymmetric cross-coupling has been achieved, yielding *N*-silylated *Si*-stereogenic products.^25^ Sulfoximines have also been coupled with (hetero)­aryl chlorides in a palladium-catalyzed *N*-arylation in aqueous micellar media^26^ and with cinnamic acids in mild one-pot reactions without the use of metals.^27,28^

Recently, our group also published some sulfoximine functionalizations, including a one-pot synthesis of *N*-iodosulfoximines from starting sulfides^29^ and oxidations of *N*-SCF_3_ sulfoximines to the corresponding *N*-sulfinyl^30^ and *N*-sulfonyl^31^ analogs.

For *N*-S functionalized sulfoximines, recent literature describes the synthesis of *N*-sulfinyl sulfoximines using sodium benzenesulfinates and dibenzothiophenium salts in DMF (Scheme 1a),^32^ the synthesis of sulfoximine-based sulfinamidines from sulfenamides (Scheme 1b),^33^ and the reaction of sulfoximines with DABSO (1,4-Diazabicyclo[2.2.2]­octane bis­(sulfur dioxide) adduct), aryldiazonium tetrafluoroborates, and CuBr in DCE to produce *N*-sulfonyl sulfoximines (Scheme 1c).^9^ Interestingly, *N*-arylsulfenyl sulfoximines can be prepared in a variety of ways (Scheme 1d),^34–36^ while *N*-alkylsulfenyl sulfoximines could be obtained by mechanochemical means with dialkyl disulfides and Ag_2_O (Scheme 1e).^37^ Sulfoximines have also undergone a thiocarbamylation in a three-component one-pot electrochemical reaction using CS_2_ and amines (Scheme 1f).^38^ As can be seen, several of these reactions require transition-metal catalysts, unfavorable solvents, strong bases, use bulky leaving groups that generate additional waste, or have reaction times of more than 24 h.

**Scheme 1:**
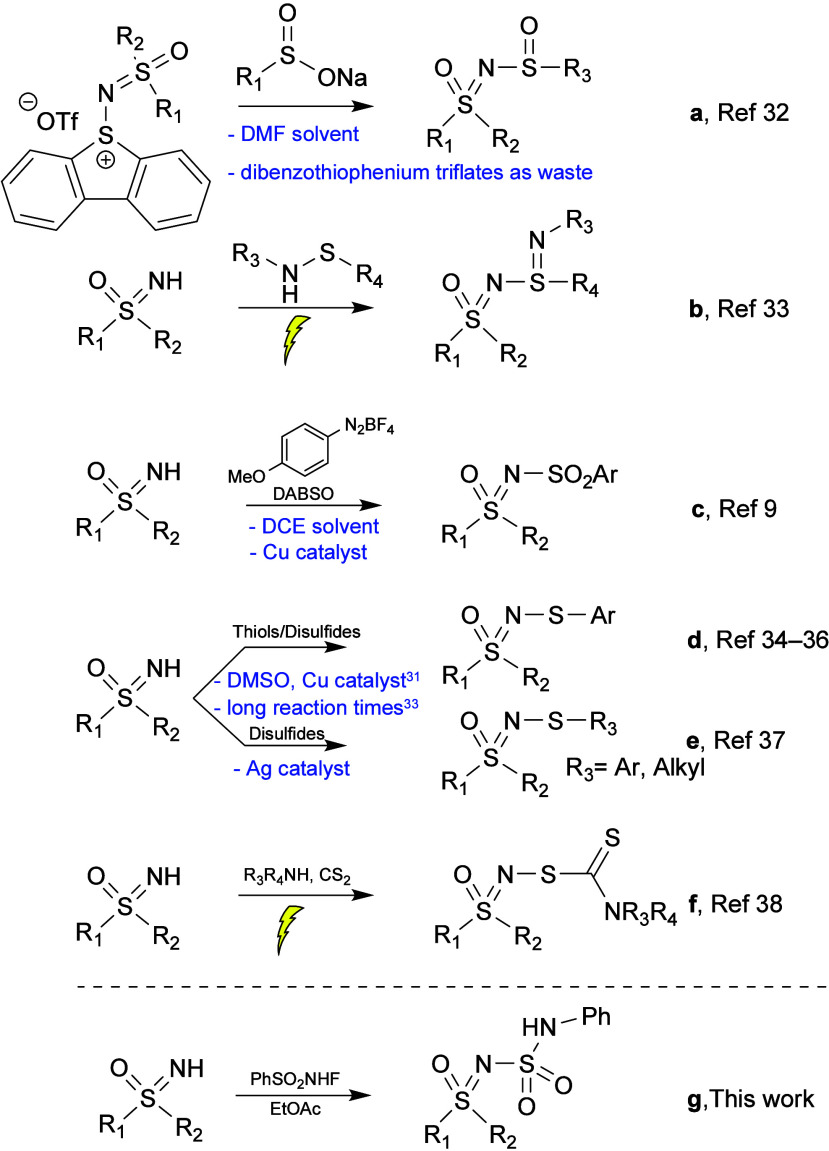
Recent *N*–S Functionalizations of Sulfoximines

*N*-fluorobenzenesulfonamide (Ph-SO_2_–NHF, **2**, Scheme 1g) can be readily obtained by the cleavage of NFSI (*N*-fluoro-*N*-(phenylsulfonyl)­benzenesulfonamide), a cost-effective reagent (cca. 0.1 €/g), with pyridine^39^ and usually serves as a means of adding the sulfonamide moiety to various electrophiles.^40,41^ From the products obtained, we assume that it can also undergo a rearrangement under our reaction conditions, likely forming the *N*-sulfonylimine intermediate (Ph-N = SO_2_). The products formed in this reaction are then similar to using phenylsulfamoyl fluorides (Ph-NH–SO_2_F, **2-SO**_**2**_ **F**), which are also of great interest in the emerging field of SuFEx click chemistry.^42–44^

Inspired by the unique chemistry of sulfoximines, the underdevelopment of the field, and the interesting rearrangement observed, we decided to further investigate the formation of *N*-(*N*-phenylsulfamoyl) sulfoximines, which have received little attention in the scientific literature so far. To the best of our knowledge, the only available procedure in the literature is a coupling reaction of a sulfoxide and a sulfamide, which requires the use of a rhodium catalyst.^45^ Herein, we present a new route to obtain these compounds by a reaction of sulfoximines **1** and *N*-fluorobenzenesulfonamide (**2**) in EtOAc in the presence of TMP at 50 °C for 16 h. The substrate scope comprised 30 sulfoximines with varying structures. We also tested the generality and robustness of the method using different nucleophiles. To our delight, phenol, benzhydrazide, phenylalanine, and various amines all produced the expected products. The *N*-sulfamoyl sulfoximines **3** exhibit stability to heat, acidic, and basic conditions and were also successfully used as coupling partners in the Suzuki-Miyaura coupling reaction. Solvent-dependent di- and tribromination with NBS was achieved. Methylation with MeI afforded the expected *N*-methyl derivative. This new class of compounds was fully characterized, and its structure was confirmed by a ^1^H–^15^N HMBC NMR experiment and X-ray crystallography. Calculations were also performed to determine the energy profile of the reaction.

## Results and Discussion

Initially, product **3a** was obtained by a reaction of sulfoximine **1a** with NFSI using *n*-BuLi in an inert atmosphere at −78 °C (Scheme 2). Optimization was further directed toward higher yields and more sustainable conditions, i.e., temperatures closer to rt, milder nonpyrophoric bases, and an air atmosphere (see Table S1 in ESI). A variety of bases and conditions were explored, and limited success was had with NaH in DCM and *t*-BuOK in neat conditions, but both approaches also produced the byproduct **4a** in quantities similar to product **3a**, and their separation might be challenging.

**Scheme 2:**
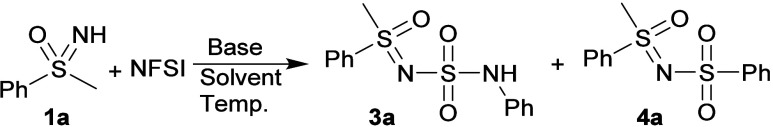
Initial Experiments with NFSI

Taking a step back, we examined using *N*-fluorobenzenesulfonamide (**2**), because we reasoned that it might improve selectivity and eliminate the unwanted byproduct **4a** (Table 1). It was a step in the right direction, but we still struggled with low yield when using different inorganic bases. Next, we switched to organic bases (Table 1, entries 2–12).

**Table 1:**
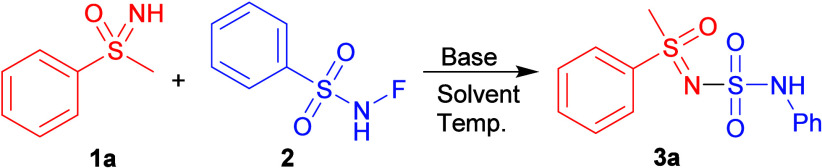
Optimization Reactions^a^

entry	base	equiv. of base	temp./solvent	equiv. of reactant	NMR yield (%)^b^
1			50 °C	1.2	27 (isolated)
2	DABCO	1.1	50 °C	1.2	24
3	Et_3_N	1.1	50 °C	1.2	50
4	*i*-Pr_2_NH	1.1	40 °C	1.5	75
5	*i*-Pr_2_NH	2	r.t.	1.5	45
6	*i*-Pr_2_NH	2	40 °C	1.5	60
7	*i*-Pr_2_NH	2.5	40 °C	1.5	58
8	TMP	1.1	40 °C	1.5	78
9	TMP	1.25	50 °C	2	100
10^c^	TMP	1.25	50 °C/DCM	1.75	94
11^c^	TMP	1.25	50 °C/MeCN	1.75	90
12^c^	TMP	1.25	50 °C/EtOAc	1.75	100

a Reaction conditions: **1a** (0.1 mmol), **2**, base, stirred for 16 h.

b Determined by ^1^H NMR with internal standard (1,3,5-trimethoxybenzene).

c **1a** (0.2 mmol), solvent, **2**, base, stirred for 16 h.

From the trends in Table 1, it was clear that slightly elevated temperatures are required (entries 5 and 6) and that a considerable excess of base has a negative effect on the conversion (entries 4 and 7). Reactions with the promising *i*-Pr_2_NH were also carried out in various solvents that performed similarly (see Table S2 in ESI), with the exception of methanol.

Of the organic bases analyzed, TMP proved the most promising. (Table 1, entries 8–12). The reactions proceeded well under all attempted conditions, except in water, where the NMR yield was 29%. Although we obtained a complete conversion under neat conditions (Table 1, entry 9), there were several byproducts in small quantities that would have reduced the isolated yield. The difficult stirring and heterogeneous reaction mixture were also a concern. We ultimately decided to use EtOAc as our solvent of choice and increased the amount of reagent **2** to 2 equiv. after obtaining lower yields with 1.75 equiv of **2** for the first few substituted substrates. The reaction of **1a** was completed in 4 h (See reaction conversion in ESI), although this was not general; e. g. the diphenyl substrate **1q** took more than 8 h. Since the reaction times were so different, we decided for convenience to stir the reactions overnight (about 16 h) to ensure complete conversion of all substrates. No change in the ^1^H NMR spectra was observed for the reaction of **3a** when the reaction time was 4 or 16 h.

With a simple method in hand, a series of sulfoximines was prepared and reacted under these conditions. The products and their corresponding isolated yields are listed in Table 2.

**Table 2:**
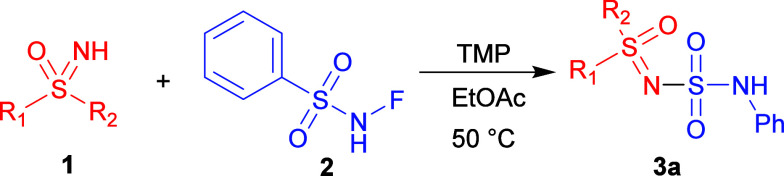

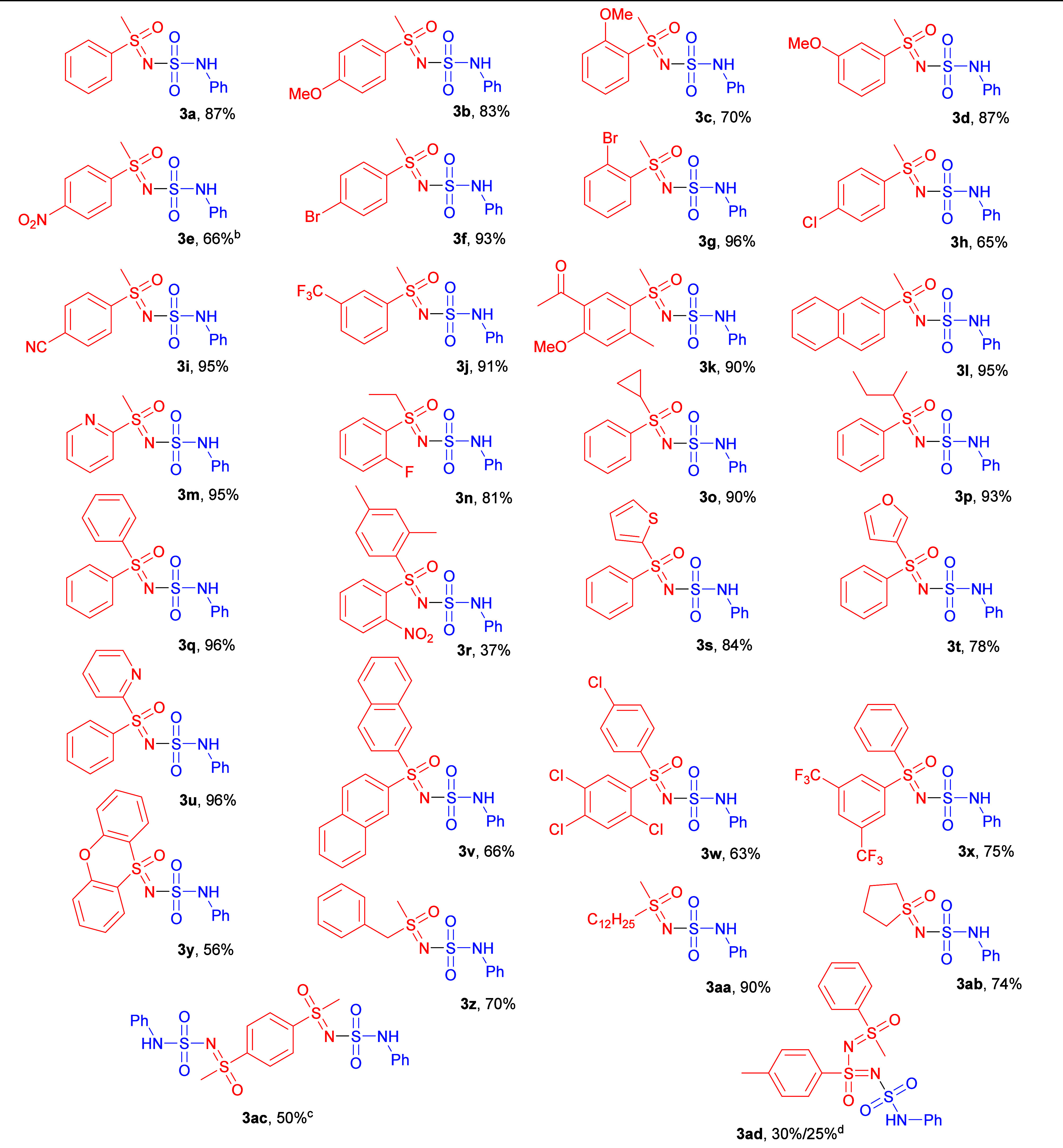
Substrate Scope^a^

a Reaction conditions: **1** (0.3 mmol), EtOAc (3 mL), **2** (2 equiv), and last TMP (1.25 equiv). Stirred for 16 h at 50 °C.

b Filtered from the reaction mixture and crystallized from DMSO/water.

c 4 equiv of **2**.

d Mix of diastereoisomers (30%)/single diastereoisomer (25%), overall yield 55%.

Most of the products were isolated in high to very high yields. The exceptions include the *p*-nitro **3e**, *p*-chloro **3h**, the substituted diaryl derivatives **3r**, **3v**–**3y**, as well as the benzyl **3z** and cyclopentyl **3ab** compounds. From the structures of these products, we can see a trend of bulky or rigid and *ortho*-susbtituted diaryl substrates showing lower reactivity under these conditions, most likely due to sterical hindrance. Products **3s**, **3v**, **3w**, and **3x** required an additional crystallization step after column chromatography to purify, additionally lowering the yield. The double sulfoximine substrates **3ac** and **3ad** also exhibit lower yields due to solubility issues during the isolation process. Interestingly, both *p*-methoxy **1b** and *p*-nitro **1e** substrates lead to complete conversion to their corresponding products. The reaction was also tested on a more complex, modified cholesterol substrate, and the product **3ae** was isolated in moderate yield (Scheme 3).

**Scheme 3:**
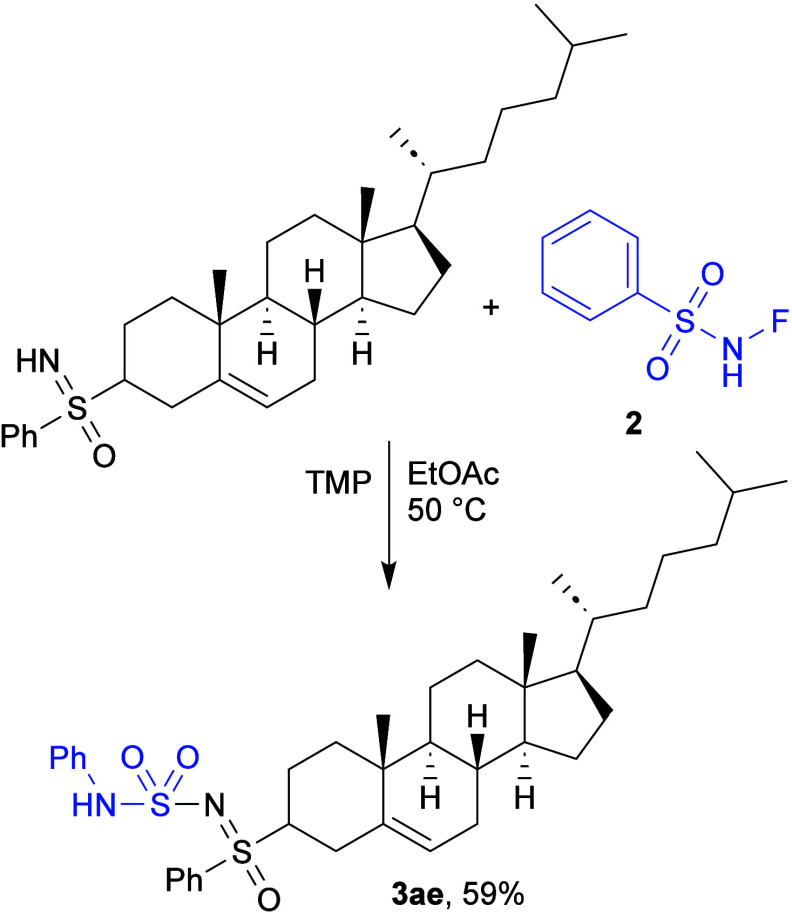
Reaction of Modified Cholesterol and **2**

A scale-up reaction was successfully accomplished on 1 g of **1a**, and product **3a** was obtained in 77% yield (Scheme 4), slightly less than 87% on the 0.3 mmol, but no changes in reaction conditions were required. Some green chemistry metrics were also calculated (see ESI).

**Scheme 4:**
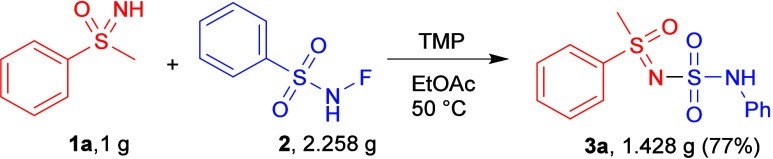
Gram-Scale Reaction

Different nucleophiles were also used instead of sulfoximines **1** using the same method (Table 3). The reactions proceeded in mostly very good yields, demonstrating the general nature and flexibility of this method.

**Table 3:**
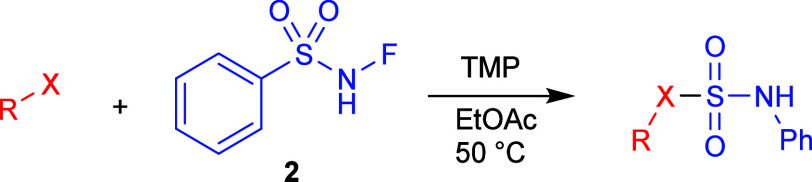

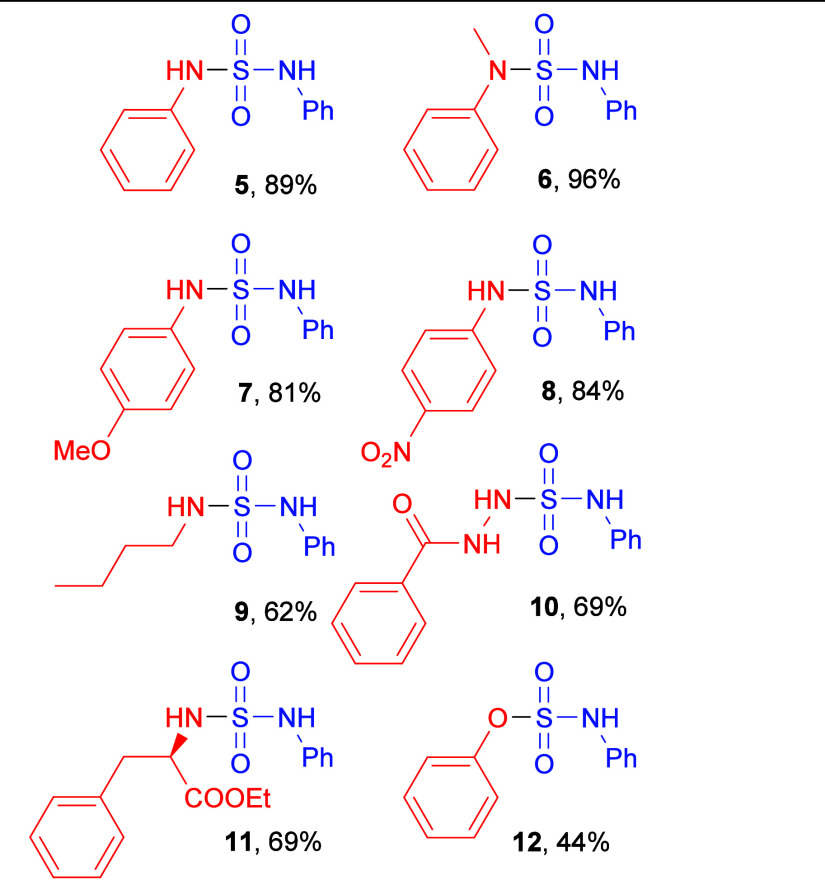
Nonsulfoximine Substrate Scope^a^

a Reaction conditions: (R-X, 0.3 mmol), EtOAc (3 mL), **2** (2 equiv), and last TMP (1.25 equiv). Stirred for 16 h at 50 °C.

The products **5**–**8** from the various anilines were obtained in high yields, and again, no appreciable substituent effect in cases of 4-NO_2_ and 4-OMe substrates was noted. The alkyl amine product **9** required additional purification and thus a decrease in yield. Interestingly, the hydrazide sulfamide derivative **10** was obtained in good yield, while the reaction with phenylhydrazine was not successful. A sulfamide **11** was formed from the amino acid l-phenylalanine. The product **12**, derived from phenol, exhibited a rather poor yield, but nevertheless supports the method. When the same reaction was attempted with benzoic acid, benzanilide **13** was isolated instead (Scheme 5), suggesting that a sulfonyl species may have been expelled from the product.

**Scheme 5:**
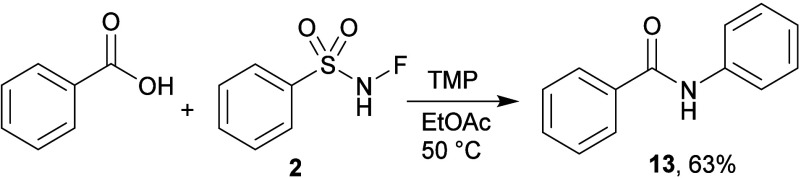
Reaction of Benzoic Acid and **2**

Next, the stability of the *N*-sulfamoyl sulfoximines was also investigated. Treatment of **3a** with aqueous solutions of 37% HCl and 50% NaOH after prolonged exposure (16 h) resulted in a partial decomposition (<10%) in the case of NaOH only. The compound **3a** remained stable up to 110 °C, while some compounds turned dark and most likely decomposed while their melting point was being acquired.

The functional group remained intact upon methylation with MeI (Scheme 6), although a stronger base than K_2_CO_3_ was required, furnishing **3af** in excellent yield.

**Scheme 6:**
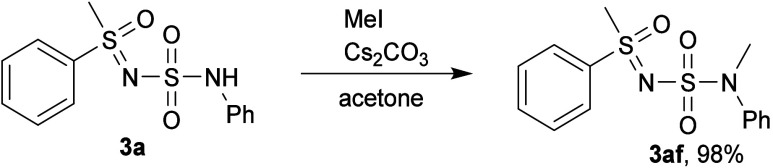
Methylation of **3a**

The Suzuki–Miyaura coupling reaction of *p*-bromo product **3f** with *p*-tolyl boronic acid in water with catalytic amounts of Pd/C gave the expected coupling product **3ag** in very good yield (Scheme 7). Surprisingly, varying the conditions of the Suzuki coupling and using Pd­(OAc)_2_ and XPhos in 1,4-dioxane resulted in the debromination reaction leading solely to **3a**.

**Scheme 7:**
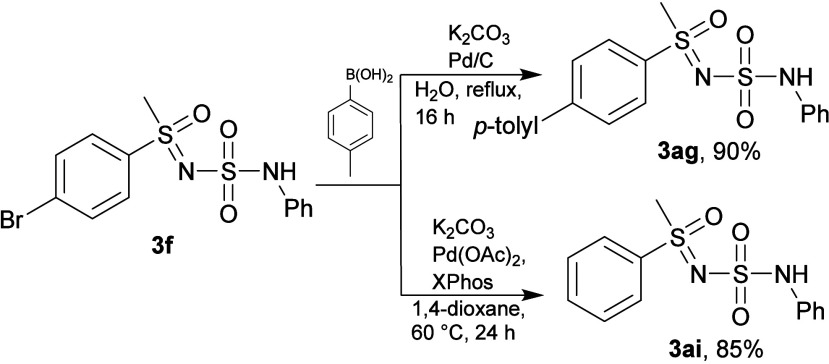
Suzuki-Miyaura Coupling Reactions

The newly introduced sulfamoyl **3a** is also sufficiently activated to effectively undergo electrophilic ring bromination with NBS (Scheme 8). In HFIP (1,1,1,3,3,3-Hexafluoro-2-propanol), dibromination was facilitated, yielding **3ah**, while in MeOH, a tribromination reaction took place, furnishing **3ai**. Conversion with NCS after several days of stirring was poor, while NIS does not appear to follow the same pattern, and the complex reaction mixture was not investigated further.

**Scheme 8:**
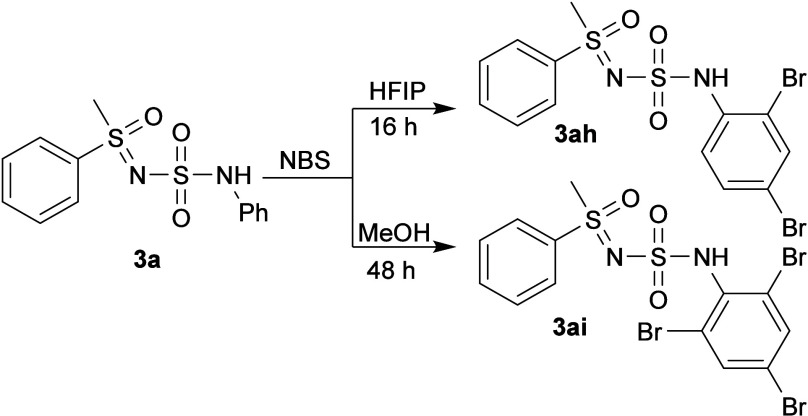
Bromination of **3a** in Different Solvents

To determine the mechanism, the reaction was first investigated by adding the radical scavenger TEMPO ((2,2,6,6-Tetramethylpiperidin-1-yl)­oxyl). No change was observed, likely excluding a radical mechanism. The structure of the products indicates that a rearrangement must have taken place, and with the reaction between **2** (^19^F NMR resonance at −92.65) and TMP, the isomeric phenylsulfamoyl fluoride (Ph-NH–SO_2_F, **2-SO**_**2**_ **F**, ^19^F NMR resonance at +50.22) was observed when the crude mixture was acidified, confirming this rearrangement (Scheme 9a). Higher temperatures and a base were essential for this step, as **2** remained unchanged at room temperature or without a base. After 5 min, all of the starting **2** was converted to **2-SO**_**2**_ **F**, which slowly decomposed into many byproducts under these conditions. We suppose that the base, while necessary to allow rearrangement, also decomposes **2-SO**_**2**_ **F** to some extent. In an additional experiment **2** (1.2 equiv) was added gradually (0.2 equiv. every 30 min) to a mixture of **1a** and TMP to limit the alleged decomposition. After 6 h, the conversion was 40%, indicating that an excess of **2** is needed in any case.

**Scheme 9:**
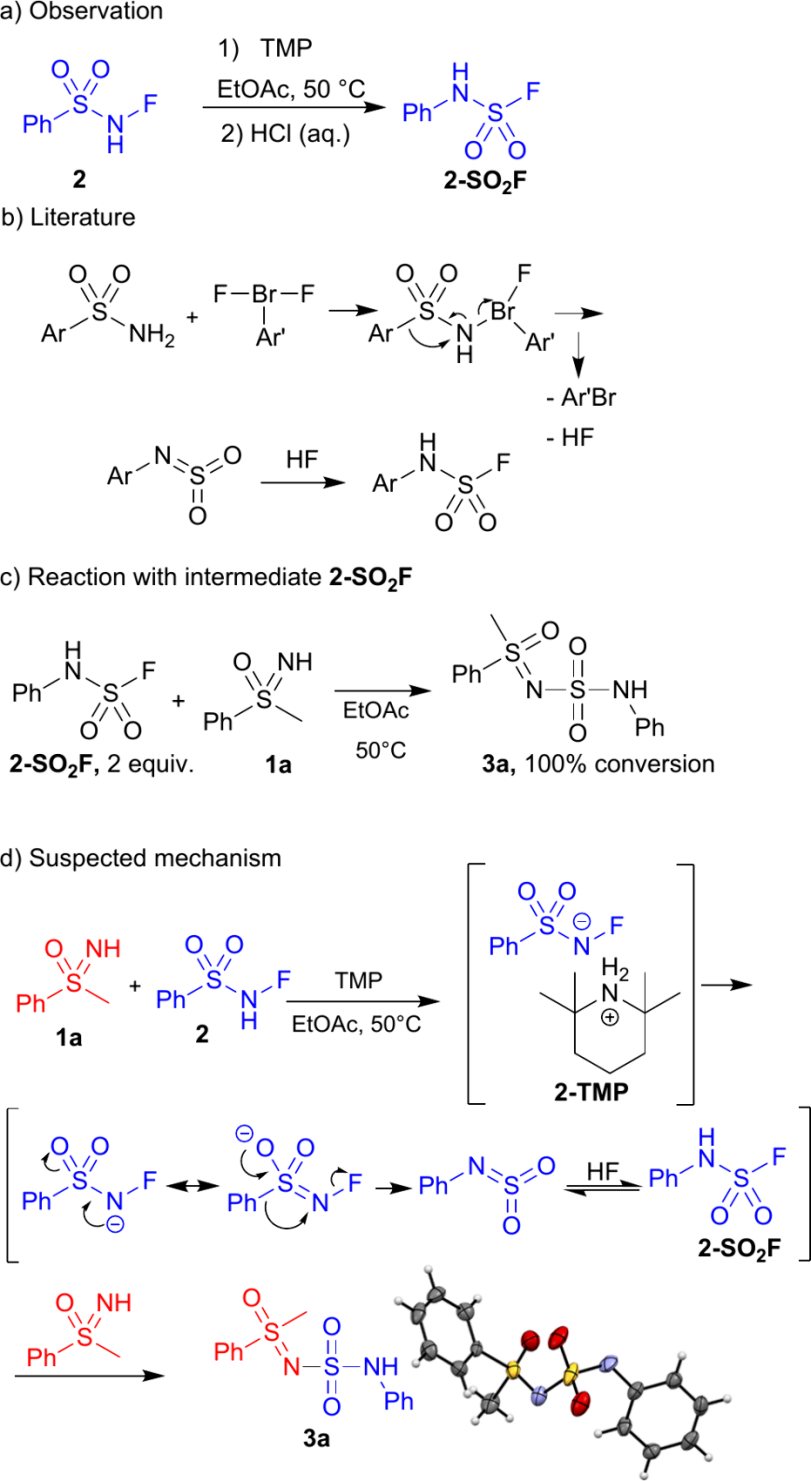
Proposed Mechanism for the Formation of **3a** and Its Crystal Structure

A similar reaction was described in literature in which arylsulfonamides (PhSO_2_NH_2_) were reacted with difluoro-λ^3^-bromanes (ArBrF_2_), yielding phenylsulfamoyl fluorides (Ar-NH–SO_2_F) (Scheme 9b).^46^ To test whether sulfamoyl fluorides are indeed capable of generating the same products as **2**, **2-SO**_**2**_ **F** was synthesized separately and reacted with **1a**, furnishing the corresponding product **3a** (Scheme 9c). The conversion was 100% after 8 h.

^19^F NMR showed no evidence of a TMP fluoride salt in the reaction mixture, indicating that TMP first deprotonates **2** and forms a precipitate in the reaction mixture (**2-TMP**, Scheme 9d). **2-TMP** then possibly undergoes a rearrangement, forming the electrophilic species that reacts with the nucleophile present.

DFT calculations were performed to analyze the reaction’s energy profile (Figure 2). The initial reference point (A) was defined using sulfoximine **1a** and the rearranged reactant **2**, with their combined energies set as the baseline. The rearrangement step was calculated to be energetically favorable, with a Δ*G* of −60.05 kJ/mol (M02 → M03 in ESI). As the reactants approached each other, a prereaction complex (B) formed, which led to further stabilization of the system. The nucleophilic attack of the imine group on the sulfoximine proceeded via a transition state (C), characterized by an imaginary frequency, with an energy barrier of 93.12 kJ/mol. This barrier is consistent with the mild heating conditions used for the reaction. Finally, product formation (D) leads to an overall change in Gibbs free energy (Δ*G*) of −104.28 kJ/mol, indicating a thermodynamically favorable transformation.

**Figure 2:**
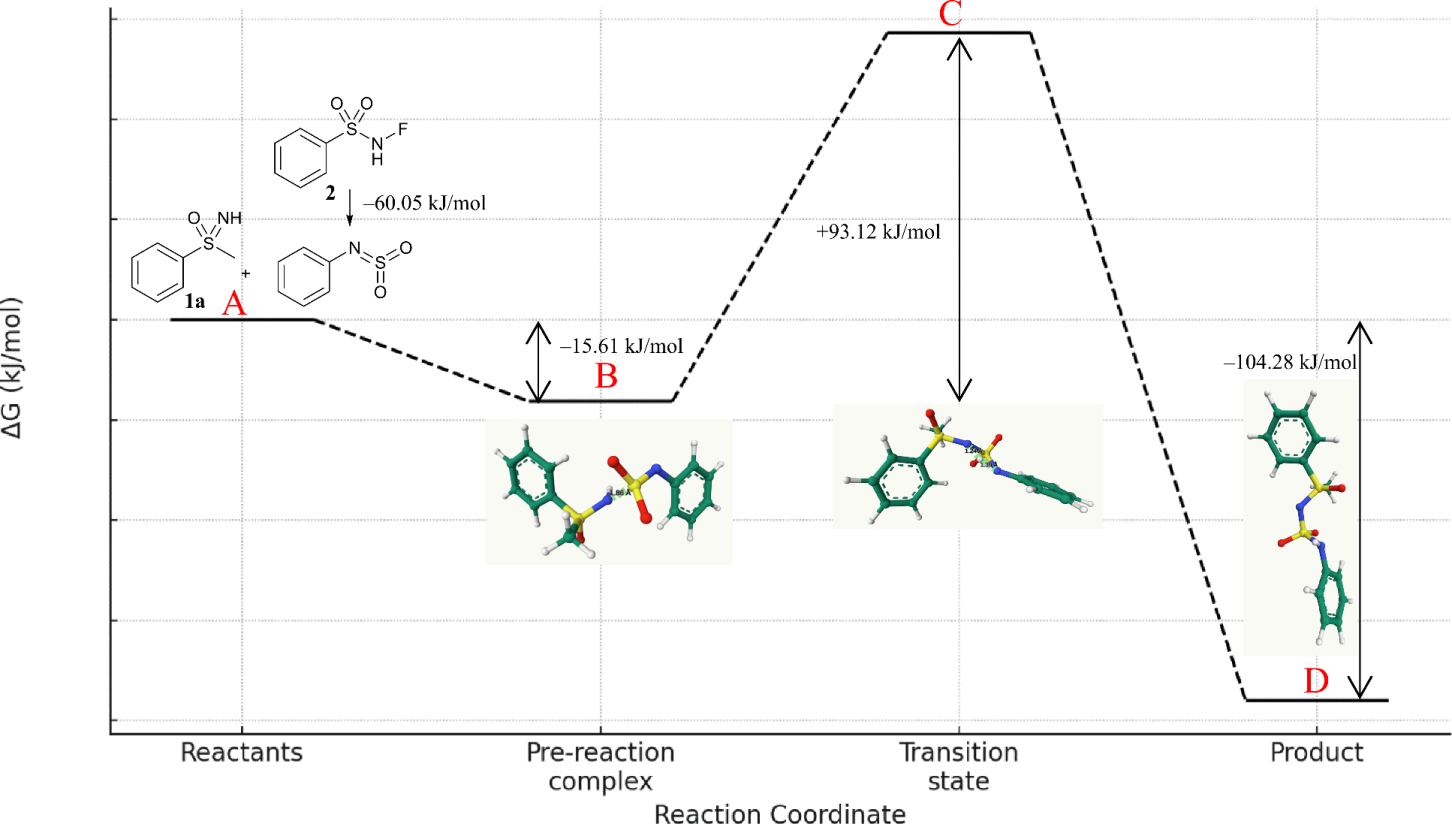
Calculated Changes of the Gibbs Free Energies for the Reaction of **1a** and **2**.

## Conclusions

In conclusion, new pathways to obtain structurally different *N*-(*N*-phenylsulfamoyl) sulfoximines **3** and their sulfamide **5**–**11** and sulfamate equivalents **12** have been reported. *N*-fluorobenzenesulfonamide (**2**) likely undergoes a rearrangement in the presence of TMP, forming the *N*-sulfonylimine intermediate that can interact with the available sulfoximine, amine, or phenol, furnishing the products in mostly very good yields. The *N*-sulfamoyl sulfoximines exhibit good stability, but some tend to decompose at temperatures close to their melting point. The same product **3a** was also isolated with isomeric phenylsulfamoyl fluoride (**2-SO**_**2**_ **F**). The newly synthesized products **3** could also be further functionalized by methylation (**3af**), Suzuki–Miyaura coupling (**3ag)**, and bromination with NBS in different solvents (**3ah**, **3ai**). The structure of **3a** was elucidated by a ^1^H–^15^N HMBC NMR experiment as well as X-ray crystallography, and DFT calculations were performed to further illuminate the energy profile of the reaction.

## Experimental Section

Chemicals and solvents were obtained from commercial sources. TLC was performed on Merck-60-F254 plates using mixtures of petroleum ether (PE), hexane, dichloromethane (DCM), ethyl acetate, and acetone. For flash chromatography, silica gel (63–200 μm, 70–230 mesh ASTM; Fluka) was used. Products were characterized by ^1^H, ^13^C, and ^19^F NMR spectroscopy, IR spectroscopy, HRMS, and melting points of the solids. All NMR spectra were recorded either in CDCl_3_ using Me_4_Si as an internal standard or in DMSO-*d*6. Chemical shifts are reported in δ (ppm) values relative to δ = 0 ppm (Me_4_Si) or 2.50 ppm (DMSO) for ^1^H NMR, and to the central line of CDCl_3_(δ = 77.16 ppm) for ^13^C NMR. ^19^F spectra were referenced to CFCl_3_ as an external standard at δ = 0.00 ppm. ^1^H, ^13^C, and ^19^F NMR spectra were recorded either with a Bruker Avance III 500 instrument at 500, 126, and 471 MHz, respectively, or with a Bruker Avance NEO 600 MHz NMR instrument at 600, 151, and 565 MHz. IR spectra were recorded with a Bruker FTIR Alpha Platinum spectrophotometer. LC-HRMS analyses were performed on a Shimadzu LCMS-IT-TOF system (Kyoto, Japan), composed of a Nexera XR liquid chromatograph hyphenated to a mass spectrometer with an ion trap and time-of-flight tube equipped with an electrospray ionization (ESI) source. The melting points were determined with an OptiMelt MPA100.

### Characterization of Unknown Sulfoximines **1w**, **1x**, and **1y** ^7^

#### *S*-(2,4,5-Trichlorophenyl)*-S-*(4-chlorophenyl) Sulfoximine (**1w**)

White solid (272 mg, 77%). ^1^H NMR (600 MHz, CDCl_3_) δ: 8.49 (s, 1H), 8.00–7.95 (m, 2H), 7.52 (s, 1H), 7.50–7.47 (m, 2H), 3.26 (s, 1H). ^13^C­{^1^H} NMR (151 MHz, CDCl_3_) δ: 140.3, 140.3, 138.6, 138.3, 133.5, 132.3, 132.2, 131.3, 130.5, 129.3. IR (neat): ν 3084, 1568, 1534, 1473, 1431, 1391, 1320, 1246, 1145, 1104, 1088, 1013, 964, 896, 865, 827, 749, 704, 685, 645, 618 (cm^–1^). HRMS (ESI-TOF) *m*/*z*: [M + H]^+^ Calcd for C_12_H_8_Cl_4_NOS 353.9075; Found 353.9074. Mp = 105.7–106.6 °C.

#### *S*-(3,5-Ditrifluoromethylphenyl)-*S-*phenyl Sulfoximine (**1x**)

White solid (334 mg, 95%). ^1^H NMR (600 MHz, CDCl_3_) δ: 8.49 (s, 2H), 8.10–8.04 (m, 2H), 8.01 (s, 1H), 7.65–7.58 (m, 1H), 7.58–7.54 (m, 2H), 3.25 (s, 1H). ^13^C­{^1^H} NMR (151 MHz, CDCl_3_) δ 146.8, 141.9, 133.8, 133.1 (q, *J* = 34.4 Hz), 129.9, 128.5–128.4 (m), 128.3, 126.4 (p, *J* = 3.5 Hz), 122.6 (q, *J* = 273.5 Hz). ^19^F NMR (565 MHz, Chloroform-*d*) δ −62.85. IR (neat): ν 3279, 3083, 1476, 1449, 1353, 1276, 1238, 1177, 1125, 1068, 1007, 969, 924, 903, 843, 818, 762, 721, 697, 680, 616 (cm^–1^). HRMS (ESI-TOF) *m*/*z*: [M + H]^+^ Calcd for C_14_H_10_F_6_NOS 354.0382; Found 354.0381. Mp = 82.7–83.3 °C.

#### 10-Imino-10H-10λ^4^-phenoxathiine 10-oxide (**1y**)

Colorless oil (241 mg, 97%). ^1^H NMR (600 MHz, CDCl_3_) δ: 8.12–8.08 (m, 2H), 7.63–7.56 (m, 2H), 7.41–7.33 (m, 4H), 3.27 (s, 1H). ^13^C­{^1^H} NMR (151 MHz, CDCl_3_) δ 151.2, 133.7, 127.6, 124.9, 123.6, 118.9. IR (neat): ν 3251, 3071, 1590, 1570, 1460, 1438, 1322, 1268, 1217, 1161, 1131, 1074, 972, 881, 813, 752, 709, 677, 639 (cm^–1^). HRMS (ESI-TOF) *m*/*z*: [M + H]^+^ Calcd for C_12_H_10_NO_2_S 232.0427; Found 232.0430.

### General Procedure for the Preparation of *N*-(*N*-Phenylsulfamoyl) Sulfoximines **3**

A 10 mL flask was charged with sulfoximine **1** (0.3 mmol), EtOAc (3 mL), and *N*-fluorobenzenesulfonamide (**2**). While the reaction mixture was stirred vigorously, TMP (1.25 equiv) was added, and the reaction was heated to 50 °C using a sand bath. The addition of TMP immediately precipitates a white, puffy solid, which slowly dissolves and forms a yellowish translucent mixture. After being stirred for 16 h, the reaction mixture is applied directly to a silica gel column and purified using column chromatography (petroleum ether/ethyl acetate = 1/1).

#### *N-*(*N-*Phenylsulfamoyl)*-S-*methyl*-S-*phenyl Sulfoximine (**3a**)

**1a** (0.3 mmol; 47 mg), 3 mL of EtOAc, 2 equiv of **2** (105 mg, 0.6 mmol) and 1.25 equiv of TMP (64 μL, 0.375 mmol). Purified using column chromatography (petroleum ether/ethyl acetate = 1/1). White solid (81 mg, 87%). ^1^H NMR (500 MHz, CDCl_3_) δ 8.00–7.86 (m, 2H), 7.69 (t, *J* = 7.4 Hz, 1H), 7.58 (t, *J* = 7.7 Hz, 2H), 7.38–7.24 (m, 4H), 7.14 (td, *J* = 7.1, 1.6 Hz, 1H), 6.58 (s, 1H), 3.31 (s, 3H). ^13^C­{^1^H} NMR (126 MHz, CDCl_3_) δ 138.0, 137.8, 134.6, 129.9, 129.3, 127.6, 124.9, 121.5, 45.8. IR (neat): ν 3258, 3030, 2929, 1600, 1496, 1474, 1447, 1402, 1321, 1304, 1220, 1137, 1092, 1057, 1021, 998, 973, 918, 903, 836, 792, 757, 735, 700, 683, 635 (cm^–1^). HRMS (ESI-TOF) *m*/*z*: [M + H]^+^ Calcd for C_13_H_15_N_2_O_3_S_2_ 311.0519; Found 311.0516. Mp = 152.0–152.5 °C.

#### *N-*(*N-*Phenylsulfamoyl)*-S-*(4-methoxyphenyl)*-S-*methyl Sulfoximine (**3b**)

**1b** (0.3 mmol; 56 mg), 3 mL of EtOAc, 2 equiv of **2** (105 mg, 0.6 mmol) and 1.25 equiv of TMP (64 μL, 0.375 mmol). Purified using column chromatography (petroleum ether/ethyl acetate = 1/1). White solid (85 mg, 83%). ^1^H NMR (500 MHz, CDCl_3_) δ: 7.91–7.81 (m, 2H), 7.36–7.27 (m, 4H), 7.17–7.10 (m, 1H), 7.06–6.98 (m, 2H), 6.41 (s, 1H), 3.89 (s, 3H), 3.29 (s, 3H). ^13^C­{^1^H} NMR (126 MHz, CDCl_3_) δ: 164.5, 137.9, 129.8, 129.3, 128.8, 124.8, 121.4, 115.1, 56.0, 46.4. IR (neat): ν 3263, 2924, 1592, 1494, 1469, 1450, 1414, 1332, 1319, 1303, 1267, 1233, 1219, 1194, 1146, 1094, 1044, 1018, 975, 955, 916, 896, 832, 804, 782, 762, 730, 696, 659, 639, 624, 608 (cm^–1^). HRMS (ESI-TOF) *m*/*z*: [M + H]^+^ Calcd for C_14_H_17_N_2_O_4_S_2_ 341.0624; Found 341.0619. Mp = 138.6–139.0 °C.

#### *N-*(*N-*Phenylsulfamoyl)*-S-*(2-methoxyphenyl)*-S-*methyl Sulfoximine (**3c**)

**1c** (0.3 mmol; 56 mg), 3 mL of EtOAc, 2 equiv of **2** (105 mg, 0.6 mmol) and 1.25 equiv of TMP (64 μL, 0.375 mmol). Purified using column chromatography (petroleum ether/ethyl acetate = 1/1). White solid (71 mg, 70%). ^1^H NMR (500 MHz, CDCl_3_) δ 7.98 (d, *J* = 8.0 Hz, 1H), 7.63 (t, *J* = 7.9 Hz, 1H), 7.32–7.25 (m, 2H), 7.24–7.18 (m, 2H), 7.11 (dt, *J* = 15.1, 7.5 Hz, 2H), 7.01 (d, *J* = 8.4 Hz, 1H), 6.47 (s, 1H), 3.86 (s, 3H), 3.46 (s, 3H). ^13^C­{^1^H} NMR (126 MHz, CDCl_3_) δ 157.1, 138.1, 136.7, 130.6, 129.2, 124.7, 124.3, 121.2, 120.7, 112.9, 56.5, 43.9. IR (neat): ν 3286, 3022, 2938, 1593, 1480, 1454, 1434, 1415, 1332, 1313, 1297, 1280, 1250, 1218, 1114, 1061, 1041, 1031, 1010, 982, 918, 829, 802, 747, 724, 688, 668, 641 (cm^–1^). HRMS (ESI-TOF) *m*/*z*: [M + H]^+^ Calcd for C_14_H_17_N_2_O_4_S_2_ 341.0624; Found 341.0624. Mp = 150.1–150.6 °C.

#### *N-*(*N-*Phenylsulfamoyl)*-S-*(3-methoxyphenyl)*-S-*methyl Sulfoximine (**3d**)

**1d** (0.3 mmol; 56 mg), 3 mL of EtOAc, 2 equiv of **2** (105 mg, 0.6 mmol) and 1.25 equiv of TMP (64 μL, 0.375 mmol). Purified using column chromatography (petroleum ether/ethyl acetate = 1/1). Yellow oil (89 mg, 87%). ^1^H NMR (600 MHz, CDCl_3_) δ: 7.52–7.44 (m, 2H), 7.42–7.39 (m, 1H), 7.35–7.27 (m, 4H), 7.22–7.17 (m, 1H), 7.16–7.11 (m, 1H), 6.73–6.39 (m, 1H), 3.85 (s, 3H), 3.30 (s, 3H). ^13^C­{^1^H} NMR (126 MHz, CDCl_3_) δ: 160.3, 139.0, 137.8, 130.8, 129.2, 124.7, 121.2, 121.1, 119.4, 111.8, 55.9, 45.7. IR (neat): ν 3276, 3021, 2931, 2836, 1596, 1482, 1420, 1336, 1314, 1289, 1239, 1169, 1141, 1084, 1060, 1036, 997, 985, 963, 919, 877, 846, 827, 784, 771, 753, 716, 692, 671, 642 (cm^–1^). HRMS (ESI-TOF) *m*/*z*: [M + H]^+^ Calcd for C_14_H_17_N_2_O_4_S_2_ 341.0624; Found 341.0624.

#### *N-*(*N-*Phenylsulfamoyl)*-S-*(4-nitrophenyl)*-S-*methyl Sulfoximine (**3e**)

**1e** (0.3 mmol; 60 mg), 3 mL of EtOAc, 2 equiv of **2** (105 mg, 0.6 mmol) and 1.25 equiv of TMP (64 μL, 0.375 mmol). The product precipitated from the reaction mixture and was filtered off. The solid was then dissolved in a small amount of DMSO and precipitated again by addition of 20 mL of water. The precipitate was filtered and washed with Et_2_O. Yellow solid (70 mg, 66%). ^1^H NMR (600 MHz, DMSO-*d*_6_) δ 9.87 (s, 1H), 8.43–8.38 (m, 2H), 8.17–8.13 (m, 2H), 7.27–7.21 (m, 2H), 7.11 (dt, *J* = 8.5, 1.0 Hz, 2H), 7.01 (td, *J* = 7.4, 1.1 Hz, 1H), 3.61 (s, 3H). ^13^C­{^1^H} NMR (151 MHz, DMSO-*d*_6_) δ 150.6, 143.9, 138.7, 129.4, 128.8, 124.4, 122.7, 118.8, 43.8. IR (neat): ν 3279, 3114, 3020, 2929, 1599, 1530, 1484, 1422, 1402, 1336, 1289, 1246, 1226, 1138, 1097, 1061, 1011, 986, 961, 920, 852, 819, 783, 756, 739, 713, 694, 646 (cm^–1^). HRMS (ESI-TOF) *m*/*z*: [M + H]^+^ Calcd for C_13_H_14_N_3_O_5_S_2_ 356.0369; Found 356.0371. Mp = 193.1 °C (decomposition).

#### *N-*(*N-*Phenylsulfamoyl)*-S-*(4-bromophenyl)*-S-*methyl Sulfoximine (**3f**)

**1f** (0.3 mmol; 70 mg), 3 mL of EtOAc, 2 equiv of **2** (105 mg, 0.6 mmol) and 1.25 equiv of TMP (64 μL, 0.375 mmol). Purified using column chromatography (petroleum ether/ethyl acetate = 1/1). White solid (109 mg, 93%). ^1^H NMR (500 MHz, CDCl_3_) δ 7.80–7.76 (m, 2H), 7.74–7.68 (m, 2H), 7.36–7.30 (m, 2H), 7.29–7.24 (m, 3H), 7.16 (t, *J* = 7.3 Hz, 1H), 6.49 (s, 1H), 3.29 (s, 3H). ^13^C­{^1^H} NMR (126 MHz, CDCl_3_) δ: 137.6, 137.1, 133.2, 130.3, 129.4, 129.1, 125.2, 121.7, 45.9. IR (neat): ν 3264, 3006, 2923, 1595, 1572, 1493, 1473, 1412, 1392, 1335, 1320, 1303, 1277, 1232, 1216, 1181, 1146, 1112, 1092, 1055, 1010, 975, 958, 918, 899, 828, 812, 784, 761, 741, 697, 654, 622 (cm^–1^). HRMS (ESI-TOF) *m*/*z*: [M + H]^+^ Calcd for C_13_H_14_BrN_2_O_3_S_2_ 388.9624; Found 388.9629. Mp = 154.9–155.5 °C.

#### *N-*(*N-*Phenylsulfamoyl)*-S-*(2-bromophenyl)*-S-*methyl Sulfoximine (**3g**)

**1g** (0.3 mmol; 70 mg), 3 mL of EtOAc, 2 equiv of **2** (105 mg, 0.6 mmol) and 1.25 equiv of TMP (64 μL, 0.375 mmol). Purified using column chromatography (petroleum ether/ethyl acetate = 1/1). Off-white solid (112 mg, 96%). ^1^H NMR (500 MHz, CDCl_3_) δ 8.23 (dt, *J* = 8.0, 1.4 Hz, 1H), 7.79 (dd, *J* = 7.7, 1.4 Hz, 1H), 7.59–7.48 (m, 2H), 7.35–7.25 (m, 4H), 7.14 (td, *J* = 7.2, 1.3 Hz, 1H), 6.48 (s, 1H), 3.52 (s, 3H). ^13^C­{^1^H} NMR (126 MHz, CDCl_3_) δ 137.7, 137.1, 136.2, 135.6, 131.9, 129.3, 128.5, 125.0, 121.5, 120.5, 43.3. IR (neat): ν 3267, 3017, 2925, 1597, 1571, 1498, 1484, 1445, 1421, 1097, 1063, 1019, 963, 916, 827, 785, 763, 740, 691, 638 (cm^–1^). HRMS (ESI-TOF) *m*/*z*: [M + H]^+^ Calcd for C_13_H_14_BrN_2_O_3_S_2_ 388.9624; Found 388.9617. Mp = 159.3–160.3 °C.

#### *N-*(*N-*Phenylsulfamoyl)*-S-*(4-chlorophenyl)*-S-*methyl Sulfoximine (**3h**)

**1h** (0.3 mmol; 57 mg), 3 mL of EtOAc, 2 equiv of **2** (105 mg, 0.6 mmol) and 1.25 equiv of TMP (64 μL, 0.375 mmol). Purified using column chromatography (petroleum ether/ethyl acetate = 1/1). After column chromatography the products was triturated with a small amount of CHCl_3_. White solid (67 mg, 65%). ^1^H NMR (600 MHz, DMSO-*d*_6_) δ 9.82 (s, 1H), 7.95–7.85 (m, 2H), 7.75–7.68 (m, 2H), 7.25 (t, *J* = 7.8 Hz, 2H), 7.16–7.10 (m, 2H), 7.01 (t, *J* = 7.4 Hz, 1H), 3.52 (s, 3H). ^13^C­{^1^H} NMR (151 MHz, DMSO-*d*_6_) δ 139.3, 138.9, 137.0, 129.5, 129.5, 128.8, 122.6, 118.7, 44.2. IR (neat): ν 3263, 3010, 2925, 1575, 1474, 1397, 1334, 1302, 1278, 1234, 1219, 1146, 1086, 1053, 1012, 974, 957, 918, 899, 829, 815, 785, 750, 714, 698, 653 (cm^–1^). HRMS (ESI-TOF) *m*/*z*: [M + H]^+^ Calcd for C_13_H_14_ClN_2_O_3_S_2_ 345.0129; Found 345.0128. Mp = 174.2–175.6 °C.

#### *N-*(*N-*Phenylsulfamoyl)*-S-*(4-cyanophenyl)*-S-*methyl Sulfoximine (**3i**)

**1i** (0.3 mmol; 54 mg), 3 mL of EtOAc, 2 equiv of **2** (105 mg, 0.6 mmol) and 1.25 equiv of TMP (64 μL, 0.375 mmol). Purified using column chromatography (petroleum ether/ethyl acetate = 1/1). Off-white solid (96 mg, 95%). ^1^H NMR (500 MHz, DMSO-*d*_6_) δ 9.85 (s, 1H), 8.12 (d, *J* = 8.7 Hz, 2H), 8.06 (d, *J* = 8.6 Hz, 2H), 7.29–7.21 (m, 2H), 7.13–7.08 (m, 2H), 7.04–6.99 (m, 1H), 3.58 (s, 3H). ^13^C­{^1^H} NMR (126 MHz, DMSO-*d*_6_) δ 142.5, 138.8, 133.4, 128.8, 128.4, 122.7, 118.8, 117.5, 116.4, 43.7. IR (neat): ν 3324, 3095, 3042, 3003, 2915, 2233, 1599, 1494, 1470, 1399, 1333, 1274, 1231, 1209, 1148, 1098, 1072, 1030, 1017, 992, 970, 919, 893, 846, 830, 791, 774, 754, 731, 709, 691, 650, 608 (cm^–1^). HRMS (ESI-TOF) *m*/*z*: [M + H]^+^ Calcd for C_14_H_14_N_3_O_3_S_2_ 336.0471; Found 336.0463. Mp = 175.3 °C (decomposition).

#### *N-*(*N-*Phenylsulfamoyl)*-S-*(3-trifluoromethylphenyl)*-S-*methyl Sulfoximine (**3j**)

**1j** (0.3 mmol; 67 mg), 3 mL of EtOAc, 2 equiv of **2** (105 mg, 0.6 mmol) and 1.25 equiv of TMP (64 μL, 0.375 mmol). Purified using column chromatography (petroleum ether/ethyl acetate = 1/1). Off-white solid (103 mg, 91%). ^1^H NMR (600 MHz, CDCl_3_) δ 8.19 (s, 1H), 8.13 (d, *J* = 8.0 Hz, 1H), 7.95 (d, *J* = 7.8 Hz, 1H), 7.74 (ddd, *J* = 9.3, 5.3, 2.0 Hz, 1H), 7.36–7.31 (m, 2H), 7.29–7.25 (m, 2H), 7.17 (t, *J* = 7.4 Hz, 1H), 6.60 (s, 1H), 3.33 (s, 3H). ^19^F NMR (565 MHz, CDCl_3_) δ−63.30. ^13^C­{^1^H} NMR (151 MHz, CDCl_3_) δ 139.5 (d, *J* = 3.2 Hz), 137.5, 133.1–132.3 (m), 131.4 (q, *J* = 3.5 Hz), 131.0, 130.8, 129.4, 125.3, 124.8 (d, *J* = 3.9 Hz), 122.9 (q, *J* = 273.1 Hz), 121.6 (d, *J* = 4.2 Hz), 45.7. IR (neat): ν 3314, 3031, 2923, 1601, 1500, 1479, 1433, 1410, 1324, 1282, 1219, 1175, 1160, 1128, 1109, 1068, 1032, 999, 971, 921, 899, 816, 778, 751, 733, 688, 640 (cm^–1^). HRMS (ESI-TOF) *m*/*z*: [M + H]^+^ Calcd for C_14_H_14_F_3_N_2_O_3_S_2_ 379.0392; Found 379.0396. Mp = 118.8–120.3 °C.

#### *N-*(*N-*Phenylsulfamoyl)*-S-*(5-acetyl-4-methoxy-2-methylphenyl)*-S-*methyl Sulfoximine (**3k**)

**1k** (0.3 mmol; 72 mg), 3 mL of EtOAc, 2 equiv of **2** (105 mg, 0.6 mmol) and 1.25 equiv of TMP (64 μL, 0.375 mmol). Column chromatography was performed using EtOAc as the mobile phase. The product was also triturated with a small amount of CHCl_3_. White solid (107 mg, 90%). ^1^H NMR (600 MHz, CDCl_3_) δ 8.39 (s, 1H), 7.35–7.24 (m, 4H), 7.14 (t, *J* = 7.2 Hz, 1H), 6.90 (s, 1H), 6.43 (s, 1H), 3.99 (s, 3H), 3.32 (s, 3H), 2.70 (s, 3H), 2.60 (s, 3H). ^13^C­{^1^H} NMR (151 MHz, CDCl_3_) δ 197.0, 162.5, 144.4, 137.8, 133.0, 129.3, 128.2, 126.7, 124.9, 121.5, 116.4, 56.4, 44.9, 31.8, 21.4. IR (neat): ν 3265, 2945, 1665, 1596, 1549, 1483, 1416, 1393, 1338, 1316, 1259, 1236, 1179, 1145, 1087, 1045, 984, 899, 824, 791, 757, 726, 709, 693, 626 (cm^–1^). HRMS (ESI-TOF) *m*/*z*: [M + H]^+^ Calcd for C_17_H_21_N_2_O_5_S_2_ 397.0886; Found 397.0879. Mp = 170.6–172.1 °C.

#### *N-*(*N-*Phenylsulfamoyl)*-S-*(2-naphtyl)*-S-*methyl Sulfoximine (**3l**)

**1l** (0.3 mmol; 62 mg), 3 mL of EtOAc, 2 equiv of **2** (105 mg, 0.6 mmol) and 1.25 equiv of TMP (64 μL, 0.375 mmol). Purified using column chromatography (petroleum ether/ethyl acetate = 1/1). White solid (103 mg, 95%). ^1^H NMR (500 MHz, CDCl_3_) δ 8.53 (d, *J* = 2.0 Hz, 1H), 8.02 (d, *J* = 8.8 Hz, 1H), 7.98 (d, *J* = 8.2 Hz, 1H), 7.94 (d, *J* = 8.2 Hz, 1H), 7.85 (dd, *J* = 8.7, 2.0 Hz, 1H), 7.71 (ddd, *J* = 8.2, 6.8, 1.3 Hz, 1H), 7.66 (ddd, *J* = 8.1, 6.8, 1.3 Hz, 1H), 7.35–7.28 (m, 4H), 6.50 (s, 1H), 3.39 (s, 3H). ^13^C­{^1^H} NMR (126 MHz, CDCl_3_) δ: 137.8, 135.7, 134.6, 132.3, 130.4, 130.1, 129.8, 129.7, 129.3, 128.3, 128.2, 125.0, 121.6, 121.6, 46.0. IR (neat): ν 3290, 3021, 2929, 1597, 1491, 1478, 1398, 1328, 1303, 1274, 1216, 1142, 1086, 1067, 1028, 978, 941, 919, 895, 878, 826, 784, 755, 735, 695, 667, 631 (cm^–1^). HRMS (ESI-TOF) *m*/*z*: [M + H]^+^ Calcd for C_17_H_17_N_2_O_3_S_2_ 361.0675; Found 361.0674. Mp = 146.3–146.8 °C.

#### *N-*(*N-*Phenylsulfamoyl)*-S-*(2-pyridyl)*-S-*methyl Sulfoximine (**3m**)

**1m** (0.3 mmol; 47 mg), 3 mL of EtOAc, 2 equiv of **2** (105 mg, 0.6 mmol) and 1.25 equiv of TMP (64 μL, 0.375 mmol). Purified using column chromatography (petroleum ether/ethyl acetate = 1/1). Off-white solid (88 mg, 95%). ^1^H NMR (500 MHz, CDCl_3_) δ 8.71 (ddd, *J* = 4.7, 1.8, 0.9 Hz, 1H), 8.10 (dt, *J* = 7.9, 1.0 Hz, 1H), 7.97 (td, *J* = 7.8, 1.7 Hz, 1H), 7.59 (ddd, *J* = 7.7, 4.7, 1.1 Hz, 1H), 7.32 (dd, *J* = 8.5, 7.2 Hz, 2H), 7.27 (d, *J* = 9.0 Hz, 2H), 7.15 (ddt, *J* = 7.6, 6.8, 1.2 Hz, 1H), 6.44 (s, 1H), 3.49 (s, 3H). ^13^C­{^1^H} NMR (126 MHz, CDCl_3_) δ 156.2, 150.3, 138.7, 137.8, 129.3, 128.2, 125.0, 122.9, 121.6, 40.9. IR (neat): ν 3257, 3007, 2923, 1595, 1579, 1557, 1493, 1475, 1449, 1413, 1331, 1303, 1281, 1235, 1217, 1142, 1123, 1084, 1061, 1026, 980, 958, 919, 901, 793, 759, 739, 696, 662, 612 (cm^–1^). HRMS (ESI-TOF) *m*/*z*: [M + H]^+^ Calcd for C_12_H_14_N_3_O_3_S_2_ 312.0471; Found 312.0471. Mp = 143.3–144.2 °C (decomposition).

#### *N-*(*N-*Phenylsulfamoyl)*-S-*(2-fluorophenyl)*-S-*ethyl Sulfoximine (**3n**)

**1n** (0.3 mmol; 56 mg), 3 mL of EtOAc, 2 equiv of **2** (105 mg, 0.6 mmol) and 1.25 equiv of TMP (64 μL, 0.375 mmol). Purified using column chromatography (petroleum ether/ethyl acetate = 1/1). Off-white solid (83 mg, 81%). ^1^H NMR (500 MHz, CDCl_3_) δ 7.97 (ddd, *J* = 7.9, 7.0, 1.8 Hz, 1H), 7.71–7.65 (m, 1H), 7.36 (td, *J* = 7.7, 1.1 Hz, 1H), 7.32–7.28 (m, 2H), 7.25–7.20 (m, 3H), 7.12 (tt, *J* = 7.3, 1.2 Hz, 1H), 6.57 (s, 1H), 3.58 (dq, *J* = 14.8, 7.4 Hz, 1H), 3.47 (dq, *J* = 14.6, 7.3 Hz, 1H), 1.21 (t, *J* = 7.4 Hz, 3H). ^19^F NMR (471 MHz, CDCl_3_) δ−107.52. ^13^C­{^1^H} NMR (126 MHz, CDCl_3_) δ 159.0 (d, *J* = 257.0 Hz), 137.2 (d, *J* = 8.6 Hz), 131.7, 129.2, 125.3 (d, *J* = 3.7 Hz), 124.7, 123.6 (d, *J* = 13.3 Hz), 121.0, 117.6 (d, *J* = 21.2 Hz), 51.4 (d, *J* = 3.4 Hz), 6.4. IR (neat): ν 3276, 3102, 2984, 2941, 1598, 1498, 1473, 1445, 1417, 1333, 1312, 1292, 1266, 1249, 1214, 1140, 1129, 1082, 1048, 911, 827, 789, 764, 734, 690, 669, 641 (cm^–1^). HRMS (ESI-TOF) *m*/*z*: [M + H]^+^ Calcd for C_14_H_16_FN_2_O_3_S_2_ 343.0581; Found 343.0548. Mp = 142.0–143.0 °C.

#### *N-*(*N-*Phenylsulfamoyl)*-S-*phenyl*-S-*cyclopropyl Sulfoximine (**3o**)

**1o** (0.3 mmol; 54 mg), 3 mL of EtOAc, 2 equiv of **2** (105 mg, 0.6 mmol) and 1.25 equiv of TMP (64 μL, 0.375 mmol). Purified using column chromatography (petroleum ether/ethyl acetate = 1/1). Off-white solid (91 mg, 90%). ^1^H NMR (500 MHz, CDCl_3_) δ 7.88 (d, *J* = 7.8 Hz, 2H), 7.66 (t, *J* = 7.4 Hz, 1H), 7.55 (t, *J* = 7.7 Hz, 2H), 7.35–7.22 (m, 4H), 7.13 (t, *J* = 7.3 Hz, 1H), 6.58 (s, 1H), 2.59 (ddd, *J* = 12.5, 7.8, 4.9 Hz, 1H), 1.47 (ddt, *J* = 11.0, 8.6, 4.5 Hz, 1H), 1.20–1.07 (m, 2H), 0.95 (td, *J* = 8.1, 3.5 Hz, 1H). ^13^C­{^1^H} NMR (126 MHz, CDCl_3_) δ 138.5, 138.1, 134.3, 129.7, 129.2, 127.8, 124.7, 121.2, 34.9, 7.5, 6.1. IR (neat): ν 3303, 3031, 1599, 1496, 1477, 1445, 1405, 1329, 1305, 1272, 1211, 1182, 1147, 1104, 1065, 1044, 1026, 998, 910, 878, 830, 795, 769, 755, 725, 685, 662, 623, 609 (cm^–1^). HRMS (ESI-TOF) *m*/*z*: [M + H]^+^ Calcd for C_15_H_17_N_2_O_3_S_2_ 337.0675; Found 337.0690. Mp = 135.9–136.4 °C.

#### *N-*(*N-*Phenylsulfamoyl)*-S-*(*sec*-butyl)*-S-*phenyl Sulfoximine (**3p**)

**1p** (0.3 mmol; 59 mg), 3 mL of EtOAc, 2 equiv of **2** (105 mg, 0.6 mmol) and 1.25 equiv of TMP (64 μL, 0.375 mmol). Purified using column chromatography (petroleum ether/ethyl acetate = 1/1). Off-white solid (99 mg, 93%). ^1^H NMR (600 MHz, CDCl_3_) δ 7.93–7.85 (m, 2H), 7.70 (tt, *J* = 7.2, 2.2 Hz, 1H), 7.63–7.55 (m, 2H), 7.34–7.27 (m, 4H), 7.13 (ddt, *J* = 8.6, 7.4, 1.3 Hz, 1H), 6.48–6.35 (m, 1H), 3.17 (ddqd, *J* = 20.4, 10.2, 6.8, 3.4 Hz, 1H), 2.01–1.76 (m, 1H), 1.38–1.20 (m, 1H), 1.17 (dd, *J* = 54.0, 6.7 Hz, 3H), 0.92–0.83 (m, 3H). ^13^C­{^1^H} NMR (151 MHz, CDCl_3_) δ 138.2, 134.9, 134.9, 134.5, 134.5, 129.6, 129.4, 129.3, 129.2, 124.6, 124.6, 121.2, 121.1, 64.1, 63.9, 22.0, 21.9, 12.0, 11.9, 11.0, 11.0 (both diastereoisomers). IR (neat): ν 3302, 2969, 2939, 2880, 1598, 1497, 1476, 1446, 1406, 1335, 1302, 1273, 1200, 1143, 1093, 1050, 1024, 997, 906, 890, 820, 790, 748, 715, 687, 639, 609 (cm^–1^). HRMS (ESI-TOF) *m*/*z*: [M + H]^+^ Calcd for C_16_H_21_N_2_O_3_S_2_ 353.0988; Found 353.0985. Mp = 112.1–112.9 °C.

#### *N-*(*N-*Phenylsulfamoyl)*-S*,*S-*diphenyl Sulfoximine (**3q**)

**1q** (0.3 mmol; 65 mg), 3 mL of EtOAc, 2 equiv of **2** (105 mg, 0.6 mmol) and 1.25 equiv of TMP (64 μL, 0.375 mmol). Purified using column chromatography (petroleum ether/ethyl acetate = 1/1). Off-white solid (108 mg, 96%). ^1^H NMR (600 MHz, CDCl_3_) δ 7.96–7.86 (m, 4H), 7.59 (tt, *J* = 7.0, 1.2 Hz, 2H), 7.50 (td, *J* = 8.1, 7.3, 1.6 Hz, 4H), 7.33–7.26 (m, 4H), 7.13 (tt, *J* = 7.0, 1.5 Hz, 1H), 6.55 (s, 1H). ^13^C­{^1^H} NMR (151 MHz, CDCl_3_) δ 139.3, 137.9, 134.0, 129.7, 129.3, 127.9, 124.8, 121.3. IR (neat): ν 3288, 3069, 1599, 1483, 1447, 1418, 1334, 1314, 1296, 1242, 1182, 1134, 1089, 1058, 1020, 993, 920, 825, 755, 744, 726, 679, 642, 611 (cm^–1^). HRMS (ESI-TOF) *m*/*z*: [M + H]^+^ Calcd for C_18_H_17_N_2_O_3_S_2_ 373.0675; Found 373.0665. Mp = 135.4 °C (decomposition).

#### *N-*(*N-P*henylsulfamoyl)*-S-*(2,4-dimethylphenyl)*-S-*(2-nitrophenyl) Sulfoximine (**3r**)

**1r** (0.3 mmol; 87 mg), 3 mL of EtOAc, 2 equiv of **2** (105 mg, 0.6 mmol) and 1.25 equiv of TMP (64 μL, 0.375 mmol). Purified using column chromatography (petroleum ether/ethyl acetate = 1/1). Light-brown solid (50 mg, 37%). ^1^H NMR (600 MHz, CDCl_3_) δ 8.38–8.32 (m, 1H), 7.84–7.75 (m, 4H), 7.31–7.25 (m, 4H), 7.17–7.10 (m, 2H), 7.05 (s, 1H), 6.54 (s, 1H), 2.36 (s, 3H), 2.28 (s, 3H). ^13^C­{^1^H} NMR (151 MHz, CDCl_3_) δ: 148.8, 145.9, 137.9, 137.6, 135.2, 134.1, 133.2, 132.8, 132.4, 132.4, 130.1, 129.3, 127.3, 125.7, 124.9, 121.1, 21.6, 20.1. IR (neat): ν 3264, 3095, 2898, 1600, 1540, 1495, 1412, 1346, 1235, 1146, 1076, 1027, 917, 851, 820, 752, 694, 666, 635 (cm^–1^). HRMS (ESI-TOF) *m*/*z*: [M + H]^+^ Calcd for C_20_H_20_N_3_O_5_S_2_ 446.0839; Found 446.0829. Mp = 89.1–90.0 °C (decomposition).

#### *N-*(*N-*Phenylsulfamoyl)*-S-*phenyl*-S-*(*2-*thienyl) Sulfoximine (**3s**)

**1s** (0.3 mmol; 67 mg), 3 mL of EtOAc, 2 equiv of **2** (105 mg, 0.6 mmol) and 1.25 equiv of TMP (64 μL, 0.375 mmol). Purified using column chromatography (petroleum ether/ethyl acetate = 2/1). After column chromatography the product was crystallized from DCM/hexane at 0 °C. White solid (95 mg, 84%). ^1^H NMR (600 MHz, CDCl_3_) δ 7.96–7.91 (m, 2H), 7.71 (dd, *J* = 5.0, 1.4 Hz, 1H), 7.65 (dd, *J* = 3.9, 1.4 Hz, 1H), 7.63–7.58 (m, 1H), 7.54–7.48 (m, 2H), 7.35–7.30 (m, 2H), 7.30–7.27 (m, 2H), 7.14 (tt, *J* = 7.1, 1.4 Hz, 1H), 7.08 (dd, *J* = 5.0, 3.9 Hz, 1H), 6.58–6.50 (m, 1H). ^13^C­{^1^H} NMR (151 MHz, CDCl_3_) δ 140.1, 139.9, 137.7, 135.7, 134.8, 134.1, 129.7, 129.3, 128.5, 127.5, 124.9, 121.3. IR (neat): ν 3262, 3208, 3092, 1599, 1488, 1445, 1422, 1396, 1337, 1318, 1300, 1241, 1222, 1144, 1093, 1053, 1013, 996, 925, 908, 856, 826, 752, 719, 682, 648, 613 (cm^–1^). HRMS (ESI-TOF) *m*/*z*: [M + H]^+^ Calcd for C_16_H_15_N_2_O_3_S_3_ 379.0239; Found 379.0233. Mp = 103.4–104.4 °C.

#### *N-*(*N-*Phenylsulfamoyl)*-S-*(3-furyl)*-S-*phenyl Sulfoximine (**3t**)

**1t** (0.3 mmol; 62 mg), 3 mL of EtOAc, 2 equiv of **2** (105 mg, 0.6 mmol) and 1.25 equiv of TMP (64 μL, 0.375 mmol). Purified using column chromatography (petroleum ether/ethyl acetate = 1/1). An additional column had to be performed (DCM as the mobile phase) to fully purify the product. Off-white solid (85 mg, 78%). ^1^H NMR (600 MHz, CDCl_3_) δ 7.99 (dd, *J* = 1.7, 0.9 Hz, 1H), 7.95–7.91 (m, 2H), 7.65–7.61 (m, 1H), 7.56–7.51 (m, 2H), 7.44 (t, *J* = 1.9 Hz, 1H), 7.34–7.30 (m, 2H), 7.29–7.26 (m, 2H), 7.14 (tt, *J* = 7.2, 1.3 Hz, 1H), 6.58 (s, 1H), 6.53 (dd, *J* = 2.1, 0.9 Hz, 1H). ^13^C­{^1^H} NMR (151 MHz, CDCl_3_) δ: 147.4, 145.5, 139.2, 137.8, 134.3, 129.7, 129.3, 127.6, 127.2, 124.9, 121.3, 108.7. IR (neat): ν 3258, 3143, 1600, 1543, 1490, 1447, 1422, 1336, 1321, 1304, 1245, 1230, 1211, 1180, 1140, 1094, 1061, 998, 923, 870, 825, 750, 726, 698, 681, 653, 634, 621 (cm^–1^). HRMS (ESI-TOF) *m*/*z*: [M + H]^+^ Calcd for C_16_H_15_N_2_O_4_S_2_ 363.0468; Found 363.0468. Mp = 107.1–108.2 °C.

#### *N-*(*N-*Phenylsulfamoyl)*-S-*phenyl*-S-*(2-pyridyl) Sulfoximine (**3u**)

**1u** (0.3 mmol; 65 mg), 3 mL of EtOAc, 2 equiv of **2** (105 mg, 0.6 mmol) and 1.25 equiv of TMP (64 μL, 0.375 mmol). Purified using column chromatography (petroleum ether/ethyl acetate = 1/1). White solid (107 mg, 96%). ^1^H NMR (600 MHz, CDCl_3_) δ 8.62 (dd, *J* = 4.7, 1.6 Hz, 1H), 8.23 (d, *J* = 8.0 Hz, 1H), 8.03 (d, *J* = 8.0 Hz, 2H), 7.91 (td, *J* = 7.8, 1.7 Hz, 1H), 7.62 (t, *J* = 7.5 Hz, 1H), 7.51 (t, *J* = 7.8 Hz, 2H), 7.46 (dd, *J* = 7.7, 4.7 Hz, 1H), 7.34–7.28 (m, 4H), 7.13 (tt, *J* = 5.9, 2.8 Hz, 1H), 6.66 (s, 1H). ^13^C­{^1^H} NMR (151 MHz, CDCl_3_) δ 157.3, 150.6, 138.6, 137.9, 136.2, 134.5, 129.5, 129.2, 129.2, 127.5, 124.8, 123.5, 121.4. IR (neat): ν 3261, 1598, 1577, 1487, 1446, 1422, 1334, 1239, 1141, 1092, 1047, 1020, 989, 906, 827, 754, 727, 696, 683, 649, 612 (cm^–1^). HRMS (ESI-TOF) *m*/*z*: [M + H]^+^ Calcd for C_17_H_16_N_3_O_3_S_2_ 374.0628; Found 374.0631. Mp = 118.8 °C (decomposition).

#### *N-*(*N-*Phenylsulfamoyl)*-S*,*S*-(2-dinaphtyl) Sulfoximine (**3v**)

**1v** (0.3 mmol; 107 mg), 3 mL of EtOAc, 2 equiv of **2** (95 mg, 0.6 mmol) and 1.25 equiv of TMP (64 μL, 0.375 mmol). Purified using column chromatography (petroleum ether/ethyl acetate = 1/1). After column chromatography the product was crystallized from DCM/hexane at 0 °C. Light-brown solid (94 mg, 66%). ^1^H NMR (600 MHz, Chloroform-*d*) δ ^1^H NMR (600 MHz, CDCl_3_) δ 8.61 (d, *J* = 2.0 Hz, 2H), 7.94 (d, *J* = 8.1 Hz, 2H), 7.89 (d, *J* = 8.9 Hz, 2H), 7.85 (d, *J* = 8.1 Hz, 2H), 7.82 (dd, *J* = 8.8, 2.0 Hz, 2H), 7.64 (ddd, *J* = 8.2, 6.8, 1.3 Hz, 2H), 7.60 (ddd, *J* = 8.2, 6.9, 1.3 Hz, 2H), 7.34–7.28 (m, 4H), 7.12 (tt, *J* = 6.9, 1.6 Hz, 1H), 6.63 (s, 1H). ^13^C­{^1^H} NMR (151 MHz, CDCl_3_) δ 138.0, 135.9, 135.4, 132.3, 130.1, 129.9, 129.8, 129.7, 129.3, 128.1, 128.0, 124.7, 122.2, 121.3. IR (neat): ν 3256, 3053, 1600, 1487, 1420, 1332, 1317, 1269, 1233, 1148, 1072, 1047, 920, 859, 812, 740, 679, 639, 605 (cm^–1^). HRMS (ESI-TOF) *m*/*z*: [M + H]^+^ Calcd for C_26_H_21_N_2_O_3_S_2_ 473.0988; Found 473.0986. Mp = 184.5–186.0 °C.

#### *N-*(*N-*Phenylsulfamoyl)*-S-*(2,4,5-trichlorophenyl)*-S-*(4-chlorophenyl) Sulfoximine (**3w**)

**1w** (0.3 mmol; 107 mg), 3 mL of EtOAc, 2 equiv of **2** (105 mg, 0.6 mmol) and 1.25 equiv of TMP (64 μL, 0.375 mmol). Purified using column chromatography (petroleum ether/ethyl acetate = 1/1). After column chromatography the product was crystallized from DCM/hexane at 0 °C. Off-white solid (96 mg, 63%). ^1^H NMR (600 MHz, CDCl_3_) δ 8.41 (d, *J* = 0.9 Hz, 1H), 7.84 (dd, *J* = 8.8, 1.9 Hz, 2H), 7.53–7.51 (m, 1H), 7.50–7.46 (m, 2H), 7.31 (ddd, *J* = 8.4, 7.2, 2.4 Hz, 2H), 7.26–7.23 (m, 2H), 7.19–7.13 (m, 1H), 6.66–6.49 (m, 1H). ^13^C­{^1^H} NMR (151 MHz, CDCl_3_) δ: 141.9, 140.3, 137.2, 135.5, 135.0, 133.8, 132.9, 132.9, 131.4, 130.3, 129.9, 129.3, 125.3, 121.5. IR (neat): ν 3259, 3088, 1601, 1573, 1534, 1496, 1482, 1471, 1433, 1416, 1393, 1348, 1319, 1248, 1154, 1104, 1077, 1048, 1007, 915, 896, 876, 861, 828, 743, 688, 659, 644, 629 (cm^–1^). HRMS (ESI-TOF) *m*/*z*: [M + H]^+^ Calcd for C_18_H_13_Cl_4_N_2_O_3_S_2_ 508.9116; Found 508.9110. Mp = 162.0–163.3 °C.

#### *N-*(*N-*Phenylsulfamoyl)*-S-*(3,5-ditrifluoromethylphenyl)-*S-*phenyl Sulfoximine (**3x**)

**1x** (0.3 mmol; 106 mg), 3 mL of EtOAc, 2 equiv of **2** (105 mg, 0.6 mmol) and 1.25 equiv of TMP (64 μL, 0.375 mmol). Purified using column chromatography (petroleum ether/ethyl acetate = 1/1). After column chromatography the product was crystallized from DCM/hexane at 0 °C. White solid (114 mg, 75%). ^1^H NMR (600 MHz, CDCl_3_) δ 8.31 (s, 2H), 8.05 (s, 1H), 7.94 (dt, *J* = 8.7, 1.5 Hz, 2H), 7.68 (td, *J* = 7.3, 1.3 Hz, 1H), 7.57 (td, *J* = 8.2, 7.4, 1.8 Hz, 2H), 7.33 (td, *J* = 8.0, 7.2, 1.6 Hz, 2H), 7.27 (dd, *J* = 8.3, 1.5 Hz, 2H), 7.17 (td, *J* = 7.3, 1.3 Hz, 1H), 6.70 (s, 1H). ^19^F NMR (565 MHz, CDCl_3_) δ−63.36. ^13^C­{^1^H} NMR (151 MHz, CDCl_3_) δ 143.0, 137.4, 137.2, 135.1, 133.6 (q, *J* = 34.8 Hz), 130.3, 129.4, 128.2, 128.1 (d, *J* = 4.0 Hz), 127.6 (q, *J* = 3.6 Hz), 125.3, 122.3 (q, *J* = 273.8 Hz), 121.5. IR (neat): ν 3226, 3095, 1599, 1483, 1448, 1415, 1359, 1323, 1305, 1278, 1256, 1221, 1195, 1153, 1128, 1102, 1076, 997, 924, 905, 843, 828, 763, 730, 696, 681, 635, 622, 611 (cm^–1^). HRMS (ESI-TOF) *m*/*z*: [M + H]^+^ Calcd for C_20_H_15_F_6_N_2_O_3_S_2_ 509.0423; Found 509.0416. Mp = 140.8–141.5 °C.

#### *N-*(*N-P*henylsulfamoyl)*-S-*phenoxathiinyl Sulfoximine (**3y**)

**1y** (0.3 mmol; 70 mg), 3 mL of EtOAc, 2 equiv of **2** (105 mg, 0.6 mmol) and 1.25 equiv of TMP (64 μL, 0.375 mmol). Purified using column chromatography (acetone). White solid (65 mg, 56%). ^1^H NMR (600 MHz, DMSO-*d*_*6*_) δ 9.83 (s, 1H), 7.99–7.81 (m, 4H), 7.66–7.45 (m, 4H), 7.24–7.10 (m, 2H), 7.07–6.81 (m, 3H). ^13^C­{^1^H} NMR (151 MHz, DMSO-*d*_*6*_) δ 150.8, 138.5, 136.1, 128.8, 125.6, 124.7, 122.8, 120.8, 119.1, 118.5. IR (neat): ν 3272, 3086, 1588, 1493, 1477, 1463, 1438, 1404, 1333, 1273, 1210, 1150, 1092, 1044, 920, 898, 886, 867, 777, 742, 706, 695, 664, 640, 623, 613 (cm^–1^). HRMS (ESI-TOF) *m*/*z*: [M + H]^+^ Calcd for C_18_H_15_N_2_O_4_S_2_ 387.0468; Found 387.0460. Mp = 201.2 °C (decomposition).

#### *N-*(*N-*Phenylsulfamoyl)*-S-*benzyl-*S-*methyl Sulfoximine (**3z**)

**1z** (0.3 mmol; 51 mg), 3 mL of EtOAc, 2 equiv of **2** (105 mg, 0.6 mmol) and 1.25 equiv of TMP (64 μL, 0.375 mmol). Purified using column chromatography (petroleum ether/ethyl acetate = 1/1). White solid (68 mg, 70%). ^1^H NMR (600 MHz, CDCl_3_) δ: 7.46–7.38 (m, 5H), 7.33–7.27 (m, 2H), 7.24–7.21 (m, 2H), 7.17–7.12 (m, 1H), 6.37 (s, 1H), 4.61 (s, 2H), 2.95 (s, 3H). ^13^C­{^1^H} NMR (151 MHz, CDCl_3_) δ 137.8, 131.3, 130.2, 129.5, 129.3, 126.4, 125.2, 121.9, 61.9, 39.4. IR (neat): ν 3356, 3066, 3042, 2990, 2931, 1600, 1495, 1471, 1405, 1313, 1300, 1285, 1224, 1156, 1136, 1059, 1029, 969, 912, 837, 784, 757, 744, 718, 690, 632, 606 (cm^–1^). HRMS (ESI-TOF) *m*/*z*: [M + H]^+^ Calcd for C_14_H_17_N_2_O_3_S_2_ 325.0675; Found 325.0691. Mp = 108.2–109.3 °C.

#### *N-*(*N-*Phenylsulfamoyl)*-S-*dodecyl-*S*-methyl Sulfoximine (**3aa**)

**1aa** (0.3 mmol; 74 mg), 3 mL of EtOAc, 2 equiv of **2** (105 mg, 0.6 mmol) and 1.25 equiv of TMP (64 μL, 0.375 mmol). Purified using column chromatography (petroleum ether/ethyl acetate = 1/1). Off-white solid (109 mg, 90%). ^1^H NMR (600 MHz, CDCl_3_) δ 7.34–7.29 (m, 2H), 7.28–7.25 (m, 2H), 7.16–7.12 (m, 1H), 6.50 (s, 1H), 3.34–3.11 (m, 5H), 1.82–1.61 (m, 2H), 1.40–1.18 (m, 18H), 0.88 (t, *J* = 7.0 Hz, 3H). ^13^C­{^1^H} NMR (151 MHz, CDCl_3_) δ: 138.0, 129.2, 125.0, 121.6, 55.8, 41.0, 32.0, 29.7, 29.7, 29.6, 29.5, 29.3, 29.1, 28.1, 22.8, 22.1, 14.3. IR (neat): ν 3308, 3023, 2950, 2914, 2851, 1601, 1499, 1480, 1469, 1411, 1339, 1305, 1277, 1213, 1150, 1073, 1033, 969, 907, 885, 828, 780, 754, 720, 694, 635, 607 (cm^–1^). HRMS (ESI-TOF) *m*/*z*: [M + H]^+^ Calcd for C_19_H_35_N_2_O_3_S_2_ 403.2084; Found 403.2077. Mp = 68.9–70.2 °C.

#### *N-*(*N-*Phenylsulfamoyl)*-S-*tetrahydrothienyl Sulfoximine (**3ab**)

**1ab** (0.3 mmol; 36 mg), 3 mL of EtOAc, 2 equiv of **2** (105 mg, 0.6 mmol) and 1.25 equiv of TMP (64 μL, 0.375 mmol). Purified using column chromatography (petroleum ether/ethyl acetate = 1/1). Off-white solid (61 mg, 74%). ^1^H NMR (600 MHz, CDCl_3_) δ: 7.37–7.31 (m, 2H), 7.31–7.25 (m, 2H), 7.19–7.12 (m, 1H), 6.50 (s, 1H), 3.70–3.59 (m, 2H), 3.28–3.19 (m, 2H), 2.39–2.12 (m, 4H). ^13^C­{^1^H} NMR (151 MHz, CDCl_3_) δ: 137.7, 129.3, 125.1, 121.8, 53.9, 23.4. IR (neat): ν 3295, 2961, 1596, 1493, 1412, 1317, 1298, 1273, 1201, 1141, 1041, 1024, 920, 824, 744, 716, 692, 637 (cm^–1^). HRMS (ESI-TOF) *m*/*z*: [M + H]^+^ Calcd for C_10_H_15_N_2_O_3_S_2_ 275.0519; Found 275.0518. Mp = 137.0–137.7 °C.

#### 1,4*-*Di*(N-*(*N-*phenylsulfamoyl)*-S-*methyl*)-S-*phenyl Sulfoximine (**3ac**)

(0.3 mmol; 70 mg), 3 mL of EtOAc, 4 equiv of **2** (210 mg, 1.2 mmol), and 2.5 equiv of TMP (128 μL, 0.75 mmol). The product precipitated from the reaction mixture, was filtered off, and washed twice with H_2_O. The solid was then triturated with CHCl_3_ and filtered again. White solid (82 mg, 50%). ^1^H NMR (600 MHz, DMSO-*d*_*6*_) δ 9.89 (d, *J* = 8.4 Hz, 2H), 8.11 (d, *J* = 6.2 Hz, 4H), 7.28–7.23 (m, 4H), 7.13 (ddd, *J* = 8.6, 5.9, 1.4 Hz, 4H), 7.01 (tdd, *J* = 7.4, 2.9, 1.3 Hz, 2H), 3.60 (d, *J* = 1.3 Hz, 6H). ^13^C­{^1^H} NMR (151 MHz, 600 MHz, DMSO-*d*_*6*_) δ 143.4, 143.3, 138.8, 128.9, 128.6, 128.6, 122.8, 122.8, 118.9, 118.9, 43.8, 43.8. IR (neat): ν 3254, 3087, 3022, 1599, 1496, 1476, 1415, 1327, 1241, 1146, 1094, 1051, 1012, 979, 917, 898, 836, 757, 730, 696, 636 (cm^–1^). HRMS (ESI-TOF) *m*/*z*: [M + H]^+^ Calcd for C_20_H_23_N_4_O_6_S_4_ 543.0495; Found 543.0494. Mp = 213.5 °C (decomposition).

#### 4-Methyl-*N*-(methyl­(oxo)­(phenyl)-λ^6^-sulfaneylidene)-*N′*-(*N*-phenylsulfamoyl)­benzenesulfonimidamide (**3ad**)

**1ad** (0.3 mmol; 88 mg), 3 mL of EtOAc, 2 equiv of **2** (105 mg, 0.6 mmol) and 1.25 equiv of TMP (64 μL, 0.375 mmol). Purified using column chromatography (petroleum ether/ethyl acetate = 1/1). Off white semisolid (35 mg, 25%). ^1^H NMR (600 MHz, DMSO-*d*6) δ 9.55 (s, 1H), 8.04–7.98 (m, 2H), 7.83–7.77 (m, 1H), 7.70–7.63 (m, 2H), 7.52–7.45 (m, 2H), 7.30–7.22 (m, 4H), 7.19–7.12 (m, 2H), 7.05–7.00 (m, 1H), 3.63 (s, 3H), 2.34 (s, 3H). ^13^C­{^1^H} NMR (151 MHz, DMSO-*d*6) δ 143.7, 139.8, 139.7, 138.0, 134.8, 129.6, 129.4, 128.9, 127.9, 126.6, 122.5, 118.9, 45.6, 21.1. IR (neat): ν 3302, 3071, 3031, 2928, 1593, 1493, 1476, 1445, 1404, 1337, 1274, 1240, 1211, 1149, 1106, 1078, 1031, 1017, 997, 972, 907, 886, 835, 815, 793, 756, 742, 693, 680, 647 (cm^–1^). HRMS (ESI-TOF) *m*/*z*: [2 M + Na]^+^ Calcd for C_40_H_42_N_6_NaO_8_S_6_ 949.1281; Found 949.1259. Mp = 200.1–201.2 °C (decomposition).

#### *N-*(*N-*Phenylsulfamoyl)*-S-*phenyl-*S*-((3*S*,8*S*,9*S*,10*R*,13*R*,14*S*,17*R*)-10,13-dimethyl-17-((*R*)-6-methylheptan-2-yl)-2,3,4,7,8,9,10,11,12,13,14,15,16,17-tetradecahydro-1*H*-cyclopenta­[a]­phenanthren-3-yl) (**3ae**)

((3*R*,8*S*,9*S*,10*R*,13*R*,14*S*,17*R*)-10,13-dimethyl-17-((*R*)-6-methylheptan-2-yl)-2,3,4,7,8,9,10,11,12,13,14,15,16,17-tetradecahydro-1*H*-cyclopenta­[a]­phenanthren-3-yl)­(imino)­(phenyl)- λ^6^-sulfanone^47^ (0.3 mmol; 153 mg), 3 mL of EtOAc, 2 equiv of **2** (105 mg, 0.6 mmol) and 1.25 equiv of TMP (64 μL, 0.375 mmol). Purified using column chromatography (ethyl acetate). White solid (118 mg, 59%). ^1^H NMR (600 MHz, CDCl_3_) δ 7.91 (td, *J* = 8.6, 1.3 Hz, 2H), 7.71–7.64 (m, 1H), 7.57 (m, 2H), 7.36–7.27 (m, 4H), 7.18–7.08 (m, 1H), 6.47 (s, 1H), 4.99 (m, 1H), 3.39–3.25 (m, 1H), 2.85–2.23 (m, 2H), 2.12–1.87 (m, 2H), 1.87–1.80 (m, 2H), 1.78–1.63 (m, 1H), 1.55 (m, 2H), 1.47–1.22 (m, 10H), 1.21–0.80 (m, 21H), 0.64 (s, 3H). ^13^C­{^1^H} NMR (151 MHz, CDCl_3_) δ 138.5, 138.4, 136.4, 136.4, 135.9, 135.6, 134.3, 134.3, 129.6, 129.6, 129.2, 129.2, 129.2, 129.2, 129.1, 124.4, 124.2, 124.1, 123.7, 120.8, 120.3, 62.6, 62.5, 56.7, 56.2, 49.2, 49.0, 42.4, 42.4, 39.8, 39.7, 39.6, 36.5, 36.5, 36.3, 35.9, 33.3, 32.9, 31.8, 31.8, 31.8, 29.8, 29.5, 28.4, 28.2, 24.3, 24.0, 23.0, 22.7, 21.2, 20.8, 20.8, 20.7, 19.6, 19.5, 18.9, 18.8, 12.0 (both diastereiosomers). IR (neat): ν 3275, 2933, 2867, 1603, 1499, 1466, 1417, 1380, 1335, 1314, 1290, 1241, 1210, 1180, 1140, 1094, 1059, 1023, 999, 957, 910, 825, 747, 710, 687, 668, 631, 612 (cm^–1^). HRMS (ESI-TOF) *m*/*z*: [M + H]^+^ Calcd for C_39_H_57_N_2_O_3_S_2_ 665.3805; Found 665.3797. Mp = 159.2–162.8 °C. α_*D*_^22^(10) = +29.47 (*c* = 0.32, CHCl_3_).

### Characterization of Phenylsulfamides **5**–**11**, Sulfamate **12**, and Benzamide **13** from Nonsulfoximine Substrates

#### *N,N′-*Diphenylsulfamide (**5**)^43^

Aniline (0.3 mmol; 28 mg), 3 mL of EtOAc, 2 equiv of **2** (105 mg, 0.6 mmol), and 1.25 equiv of TMP (64 μL, 0.375 mmol). The solvent was removed under reduced pressure and the crude product was purified by column chromatography. The column was first flashed with PE/EtOAc = 5/1 to remove the impurities and then with PE/EtOAc = 1/1 to elute the product. Brown semisolid (66 mg, 89%). ^1^H NMR (600 MHz, CDCl_3_) δ 7.31–7.26 (m, 4H), 7.15 (td, *J* = 7.4, 1.3 Hz, 2H), 7.07 (dt, *J* = 8.5, 1.3 Hz, 4H), 6.78 (s, 2H). ^13^C­{^1^H} NMR (151 MHz, CDCl_3_) δ 136.3, 129.6, 125.6, 121.4. IR (neat): ν 3330, 3258, 3055, 2970, 2896, 1596, 1486, 1409, 1343, 1298, 1214, 1147, 1079, 1031, 1001, 923, 832, 745, 689, 637 (cm^–1^). HRMS (ESI-TOF) *m*/*z*: [M + H]^+^ Calcd for C_12_H_13_N_2_O_2_S 249.0692; Found 249.0689.

#### *N*-Methyl-*N*,*N*′**-diphenylsulfamide (**6**)

*N*-Methylaniline (0.3 mmol; 32 mg), 3 mL of EtOAc, 2 equiv of **2** (105 mg, 0.6 mmol) and 1.25 equiv of TMP (64 μL, 0.375 mmol). The solvent was removed under reduced pressure and the crude product was purified by column chromatography. The column was first flashed with PE/EtOAc = 5/1 to remove the impurities and then with PE/EtOAc = 1/1 to elute the product. Yellow solid (77 mg, 96%). ^1^H NMR (600 MHz, CDCl_3_) δ 7.30 (ddd, *J* = 7.3, 4.9, and 3.6 Hz, 4H), 7.27–7.23 (m, 1H), 7.21 (dt, *J* = 8.3, 1.1 Hz, 2H), 7.16–7.10 (m, 3H), 6.77–6.63 (m, 1H), 3.27 (s, 3H). ^13^C­{^1^H} NMR (151 MHz, CDCl_3_) δ: 141.8, 137.3, 129.5, 129.3, 127.7, 127.0, 124.7, 120.0, 40.0. IR (neat): ν 3269, 3055, 2894, 1595, 1492, 1450, 1420, 1354, 1309, 1273, 1220, 1140, 1063, 1028, 923, 905, 882, 847, 754, 710, 691, 647 (cm^–1^). HRMS (ESI-TOF) *m*/*z*: [M + H]^+^ Calcd for C_13_H_15_N_2_O_2_S 263.0849; Found 263.0843. Mp = 90.1–92.0 °C.

#### *N*-Phenyl-*N*′**-4-methoxyphenylsulfamide (**7**)

4-Methoxyaniline (0.3 mmol; 37 mg), 3 mL of EtOAc, 2 equiv of **2** (105 mg, 0.6 mmol), and 1.25 equiv of TMP (64 μL, 0.375 mmol). The solvent was removed under reduced pressure and the crude product was purified by column chromatography. The column was first flashed with PE/EtOAc = 5/1 to remove the impurities and then with PE/EtOAc = 1/1 to elute the product. Dark red oil (67 mg, 81%). ^1^H NMR (600 MHz, CDCl_3_) δ 7.34–7.29 (m, 2H), 7.17–7.13 (m, 1H), 7.10 (dd, *J* = 8.5, 1.2 Hz, 2H), 7.00–6.96 (m, 2H), 6.80–6.76 (m, 2H), 6.75 (s, 1H), 6.59 (s, 1H), 3.76 (s, 3H). ^13^C­{^1^H} NMR (151 MHz, CDCl_3_) δ 158.2, 136.8, 129.6, 128.5, 125.7, 125.0, 120.4, 114.7, 55.6. IR (neat): ν 3264, 2838, 1600, 1508, 1416, 1334, 1286, 1247, 1213, 1145, 1029, 923, 826, 750, 692, 635 (cm^–1^). HRMS (ESI-TOF) *m*/*z*: [M + H]^+^ Calcd for C_13_H_15_N_2_O_3_S 279.0798; Found 279.0800.

#### *N*-Phenyl-*N*′**-4-nitrophenylsulfamide (**8**)

4-Nitroaniline (0.3 mmol; 41 mg), 3 mL of EtOAc, 2 equiv of **2** (105 mg, 0.6 mmol), and 1.25 equiv of TMP (64 μL, 0.375 mmol). The solvent was removed under reduced pressure and the crude product was purified by column chromatography. The column was first flashed with PE/EtOAc = 5/1 to remove the impurities and then with PE/EtOAc = 1/1 to elute the product. Yellow solid (74 mg, 84%). ^1^H NMR (600 MHz, DMSO-*d*_*6*_) δ 11.14 (s, 1H), 10.64 (s, 1H), 8.21–8.13 (m, 2H), 7.33–7.23 (m, 4H), 7.13–7.08 (m, 2H), 7.03 (tt, *J* = 7.3, 1.1 Hz, 1H). ^13^C­{^1^H} NMR (151 MHz, DMSO-*d*_*6*_) δ 144.6, 141.7, 137.4, 129.2, 125.3, 123.6, 118.9, 116.6. IR (neat): 3300, 3266, 3084, 1593, 1498, 1466, 1427, 1398, 1338, 1301, 1245, 1217, 1191, 1152, 1127, 1108, 1034, 940, 909, 861, 840, 747, 686, 625 (cm^–1^). HRMS (ESI-TOF) *m*/*z*: [M + H]^+^ Calcd for C_12_H_12_N_3_O_4_S 294.0543; Found 294.0539. Mp = 165.6–166.2 °C.

#### *N*-Butyl-*N*′**-phenylsulfamide (**9**)

Butylamine (0.3 mmol; 22 mg), 3 mL of EtOAc, 2 equiv of **2** (105 mg, 0.6 mmol), and 1.25 equiv of TMP (64 μL, 0.375 mmol). The solvent was removed under reduced pressure and the crude product was purified by column chromatography. The column was first flashed with PE/EtOAc = 5/1 to remove the impurities and then with pure DCM to elute the product. Off-white solid (42 mg, 62%). ^1^H NMR (600 MHz, CDCl_3_) δ 7.35–7.30 (m, 2H), 7.21–7.16 (m, 2H), 7.15–7.11 (m, 1H), 6.76 (s, 1H), 4.56 (t, *J* = 6.1 Hz, 1H), 3.07–3.02 (m, 2H), 1.48–1.41 (m, 2H), 1.31–1.22 (m, 2H), 0.84 (t, *J* = 7.4 Hz, 3H). ^13^C­{^1^H} NMR (151 MHz, CDCl_3_) δ: 137.3, 129.6, 124.7, 119.8, 43.2, 31.5, 19.8, 13.6. IR (neat): ν 3268, 2960, 2934, 2873, 1599, 1494, 1470, 1431, 1401, 1326, 1276, 1219, 1138, 1063, 1026, 1008, 952, 923, 895, 867, 840, 793, 752, 736, 695, 606 (cm^–1^). HRMS (ESI-TOF) *m*/*z*: [M + H]^+^ Calcd for C_10_H_17_N_2_O_2_S 229.1005; Found 229.1005. Mp = 48.0–48.7 °C.

#### *N*-Benzamidyl-*N*′**-phenylsulfamide (**10**)

Benzohydrazide (0.3 mmol; 41 mg), 3 mL of EtOAc, 2 equiv of **2** (105 mg, 0.6 mmol), and 1.25 equiv of TMP (64 μL, 0.375 mmol). After column chromatography (PE/EtOAc = 1/1) the product was triturated by using a small amount of CHCl_3_. White solid (60 mg, 69%). ^1^H NMR (600 MHz, DMSO-*d*_*6*_) δ 10.53 (d, *J* = 3.0 Hz, 1H), 9.93 (s, 1H), 9.53 (d, *J* = 3.0 Hz, 1H), 7.83–7.79 (m, 2H), 7.59–7.53 (m, 1H), 7.47 (t, *J* = 7.7 Hz, 2H), 7.29–7.21 (m, 4H), 6.99 (tt, *J* = 6.8, 1.8 Hz, 1H). ^13^C­{^1^H} NMR (151 MHz, DMSO-*d*_*6*_) δ 165.9, 138.6, 132.3, 131.9, 128.5, 128.3, 127.7, 122.5, 119.1. IR (neat): ν 3297, 3242, 3085, 2970, 2897, 1668, 1601, 1529, 1494, 1428, 1336, 1319, 1303, 1222, 1153, 1064, 1027, 1002, 960, 918, 866, 795, 754, 722, 690, 622, 606 (cm^–1^). HRMS (ESI-TOF) *m*/*z*: [M + H]^+^ Calcd for C_13_H_14_N_3_O_3_S 292.0750; Found 292.0750. Mp = 165.5–165.8 °C.

#### Ethyl-(*N*-phenylsulfamoyl)-*L*-phenylalaninate (**11**)

l-phenylalanine ethyl ester hydrochloride (0.3 mmol; 69 mg), 3 mL of EtOAc, 2 equiv of **2** (105 mg, 0.6 mmol), and 2.5 equiv of TMP (128 μL, 0.75 mmol). The solvent was removed under reduced pressure and the crude product was purified by column chromatography. The column was first flashed with PE/EtOAC = 5/1 to remove the impurities and then with PE/EtOAC = 1/1 to elute the product. After column chromatography, the product was triturated using a small amount of CHCl_3_. White solid (72 mg, 69%). ^1^H NMR (600 MHz, CDCl_3_) δ 7.32–7.26 (m, 2H), 7.25–7.20 (m, 3H), 7.12 (t, *J* = 7.5 Hz, 1H), 7.07 (t, *J* = 6.5 Hz, 4H), 6.45 (s, 1H), 5.08 (d, *J* = 8.7 Hz, 1H), 4.34–4.28 (m, 1H), 4.00–4.10 (m, 2H), 3.03 (d, *J* = 6.1 Hz, 2H), 1.16 (t, *J* = 7.2 Hz, 3H). ^13^C­{^1^H} NMR (151 MHz, CDCl_3_) δ: 171.3, 136.8, 135.1, 129.6, 129.5, 128.8, 127.5, 125.0, 120.5, 62.1, 57.4, 39.1, 14.1. IR (neat): ν 3282, 3234, 3035, 2972, 1716, 1603, 1491, 1434, 1419, 1369, 1352, 1288, 1212, 1192, 1148, 1100, 1029, 961, 913, 855, 759, 740, 724, 696, 663, 605 (cm^–1^). HRMS (ESI-TOF) *m*/*z*: [M + H]^+^ Calcd for C_17_H_21_N_2_O_4_S 349.1217; Found 349.1215. Mp = 157.8–159.3 °C. α_D_^22^(10) = −7.0 (*c* = 0.2, MeOH).

#### Phenyl Phenylsulfamate (**12**)^48^

Phenol (0.3 mmol; 28 mg), 3 mL of EtOAc, 2 equiv of **2** (105 mg, 0.6 mmol), and 1.25 equiv of TMP (64 μL, 0.75 mmol). The solvent was removed under reduced pressure and the crude product was purified by column chromatography (PE/EtOAc = 20/1). Yellow semisolid (33 mg, 44%). ^1^H NMR (600 MHz, CDCl_3_) δ: 7.40–7.35 (m, 2H), 7.35–7.31 (m, 2H), 7.30–7.26 (m, 1H), 7.23–7.18 (m, 3H), 7.16–7.13 (m, 2H), 6.80 (s, 1H). ^13^C­{^1^H} NMR (151 MHz, CDCl_3_) δ 150.0, 136.1, 130.0, 129.8, 127.5, 125.4, 122.2, 119.8. IR (neat): ν 3284, 3060, 1599, 1485, 1451, 1364, 1287, 1222, 1187, 1169, 1143, 1072, 1024, 1007, 937, 860, 826, 775, 750, 728, 687, 641, 618 (cm^–1^). HRMS (ESI-TOF) *m*/*z*: [M + H]^+^ Calcd for C_12_H_12_NO_3_S 250.0532; Found 250.0517.

#### *N*-Phenylbenzamide (**13**)^49^

Benzoic acid (0.3 mmol; 37 mg), 3 mL of EtOAc, 2 equiv of **2** (105 mg, 0.6 mmol), and 1.25 equiv of TMP (64 μL, 0.75 mmol). The solvent was removed under reduced pressure and the crude product was purified by column chromatography. PE/EtOAc = 5/1 was used as mobile phase. The product was then further purified by crystallization from DCM/hexane at 0 °C. White solid (33 mg, 63%). ^1^H NMR (600 MHz, CDCl_3_) δ 7.90–7.86 (m, 2H), 7.82 (s, 1H), 7.67–7.63 (m, 2H), 7.59–7.53 (m, 1H), 7.52–7.47 (m, 2H), 7.40–7.36 (m, 2H), 7.16 (tt, *J* = 7.5, 1.2 Hz, 1H). ^13^C­{^1^H} NMR (151 MHz, CDCl_3_) δ: 165.9, 138.1, 135.2, 132.0, 129.3, 129.0, 127.1, 124.7, 120.3. IR (neat): ν 3341, 3040, 1653, 1598, 1578, 1524, 1489, 1436, 1319, 1254, 1164, 1074, 1026, 1000, 927, 909, 883, 791, 748, 715, 689, 643, 615 (cm^–1^). HRMS (ESI-TOF) *m*/*z*: [M + H]^+^ Calcd for C_13_H_12_NO 198.0913; Found 198.0911. Mp = 162.4–162.7 °C.

### Post-Modification Reactions

#### Methylation

A round-bottom flask was charged with *N-*(*N-*phenylsulfamoyl)*-S-*methyl*-S-*phenyl sulfoximine (**3a**) (93 mg, 0.3 mmol) and Cs_2_CO_3_ (147 mg, 1.5 equiv). Acetone (5 mL) and MeI (37 μL, 2 equiv) were added, and the reaction mixture was stirred for 4 h at room temperature. The solvent was removed under reduced pressure. Water (30 mL) was added, and the product was extracted twice with EtOAc. The combined organic phase was dried under anhydrous Na_2_SO_4_ and the solvent was removed under reduced pressure, yielding **3af** (95 mg, 98%).

##### *N-*Methyl-*N-*(*N-*phenylsulfamoyl)*-S-*(phenyl)*-S-*methyl Sulfoximine (**3af**)

Off-white solid (95 mg, 98%). ^1^H NMR (600 MHz, CDCl_3_) δ: 7.98–7.94 (m, 2H), 7.72–7.67 (m, 1H), 7.61–7.56 (m, 2H), 7.50–7.44 (m, 2H), 7.38–7.33 (m, 2H), 7.28–7.24 (m, 1H), 3.33 (s, 3H), 3.29 (s, 3H). ^13^C­{^1^H} NMR (151 MHz, CDCl_3_) δ: 142.9, 138.5, 134.4, 129.8, 128.9, 127.7, 127.1, 126.7, 45.8, 38.9. IR (neat): ν 3056, 3029, 2930, 1595, 1491, 1451, 1414, 1323, 1220, 1166, 1140, 1097, 1055, 1021, 986, 918, 870, 793, 767, 748, 708, 695, 684, 605 (cm^–1^). HRMS (ESI-TOF) *m*/*z*: [M + H]^+^ Calcd for C_14_H_17_N_2_O_3_S_2_ 325.0675; Found 325.0668. Mp = 109.7–110.2 °C.

#### Suzuki–Miyaura Coupling

A round-bottom flask was charged with *N-*(*N-*phenylsulfamoyl)*-S-*(4-bromophenyl)*-S-*methyl sulfoximine (**3f**) (78 mg, 0.2 mmol), 4-methylphenyl boronic acid (54 mg, 2 equiv) and K_2_CO_3_ (82 mg, 3 equiv). Water (5 mL) was added along with 10% Pd/C (3 mg), and the reaction mixture was stirred under reflux for 16 h. The reaction mixture was cooled to room temperature and extracted with EtOAc. The solvent was removed under reduced pressure and the residue was purified by trituration with CHCl_3_ yielding **3ag** (72 mg, 90%).

##### *N-*(*N-*Phenylsulfamoyl)*-S-*(4*′*-methyl-[1,1*′*-biphenyl]-4-yl)*-S-*methyl Sulfoximine (**3ag**)

White solid (72 mg, 90%). ^1^H NMR (600 MHz, DMSO-*d*_6_) δ 9.82 (d, *J* = 1.8 Hz, 1H), 7.96–7.89 (m, 4H), 7.69–7.64 (m, 2H), 7.34 (d, *J* = 7.9 Hz, 2H), 7.30–7.22 (m, 2H), 7.21–7.13 (m, 2H), 7.00 (tt, *J* = 7.2, 1.2 Hz, 1H), 3.53 (s, 3H), 2.37 (s, 3H). ^13^C­{^1^H} NMR (151 MHz, DMSO-*d*_6_) δ 145.5, 139.1, 138.5, 136.4, 135.3, 129.8, 128.8, 128.1, 127.3, 127.1, 122.5, 118.7, 44.5, 20.8. IR (neat): ν 3285, 3027, 2930, 1591, 1477, 1397, 1328, 1303, 1276, 1214, 1145, 1105, 1073, 1028, 1002, 973, 920, 899, 850, 811, 781, 753, 722, 696, 666, 623, 604 (cm^–1^). HRMS (ESI-TOF) *m*/*z*: [M + H]^+^ Calcd for C_20_H_21_N_2_O_3_S_2_ 401.0988; Found 401.0986. Mp = 197.7–198.2 °C.

#### Pd-Catalyzed Debromination

A Schlenk tube was charged with *N-*(*N-*phenylsulfamoyl)*-S-*(4-bromophenyl)*-S-*methyl sulfoximine (**3f**) (78 mg, 0.2 mmol), 4-methylphenyl boronic acid (30 mg, 1.1 equiv), Pd­(OAc)_2_ (5 mg, 10 mol %), and XPhos (10 mg, 10 mol %). The tube was placed under argon and sealed with a rubber septum. The mixture was stirred at room temperature for 10 min, and then a 2 M aqueous degassed solution of K_2_CO_3_ (0.5 mL, 5.0 equiv) was added by syringe, and the mixture was heated at 60 °C for 24 h using a sand bath. After the mixture was cooled to room temperature, the solvents were removed under reduced pressure and the residue was purified by column chromatography (petroleum ether/ethyl acetate = 1/1), furnishing **3a** (53 mg, 85%).

#### Dibromination

A round-bottom flask was charged with *N-*(*N-*phenylsulfamoyl)*-S-*methyl*-S-*phenyl sulfoximine (**3a**) (62 mg, 0.2 mmol) and NBS (78 mg, 2.2 equiv). HFIP (1,1,1,3,3,3-hexafluoropropan-2-ol) (1 mL) was added, and the reaction mixture was stirred for 16 h at room temperature. The reaction mixture was diluted with water and extracted with EtOAc. The organic phase was dried using anhydrous Na_2_SO_4_, and the solvent was removed under reduced pressure, yielding **3ah** (83 mg, 88%).

##### *N-*(*N-*(2,4-Dibromophenylsulfamoyl)*-S-*(phenyl)*-S-*methyl Sulfoximine (**3ah**)

Red semisolid (83 mg, 88%). ^1^H NMR (600 MHz, CDCl_3_) δ 7.98–7.94 (m, 2H), 7.75–7.71 (m, 1H), 7.66 (d, *J* = 2.2 Hz, 1H), 7.62 (t, *J* = 8.2 Hz, 3H), 7.41 (dd, *J* = 8.8, 2.2 Hz, 1H), 7.06 (s, 1H), 3.35 (s, 3H). ^13^C­{^1^H} NMR (151 MHz, CDCl_3_) δ 137.7, 135.4, 134.9, 134.8, 131.6, 130.0, 127.6, 121.7, 116.6, 114.2, 46.1. IR (neat): ν 3314, 3023, 2927, 1710, 1582, 1474, 1447, 1411, 1376, 1326, 1236, 1147, 1096, 1059, 997, 976, 903, 812, 789, 730, 682, 651 (cm^–1^). HRMS (ESI-TOF) *m*/*z*: [M + H]^+^ Calcd for C_13_H_13_Br_2_N_2_O_3_S_2_ 466.8729; Found 466.8723.

#### Tribromination

A round-bottom flask was charged with *N-*(*N-*phenylsulfamoyl)*-S-*methyl*-S-*phenyl sulfoximine (**3a**) (62 mg, 0.2 mmol) and NBS (118 mg, 3.3 equiv). MeOH (3 mL) was added, and the reaction mixture was stirred for 48 h at room temperature. The solvent was removed under reduced pressure, and the residue was purified by column chromatography (DCM), yielding **3ai** (87 mg, 80%).

##### *N-*(*N-*(2,4,6-Tribromophenylsulfamoyl)*-S-*(phenyl)*-S-*methyl Sulfoximine (**3ai**)

Purified using column chromatography (DCM). Off-white solid (87 mg, 80%). ^1^H NMR (600 MHz, CDCl_3_) δ: 8.02–7.99 (m, 2H), 7.75 (s, 2H), 7.75–7.70 (m, 1H), 7.65–7.61 (m, 2H), 6.49 (s, 1H), 3.38 (s, 3H). ^13^C­{^1^H} NMR (151 MHz, CDCl_3_) δ 138.2, 135.5, 134.7, 134.2, 130.0, 127.6, 125.1, 121.7, 46.2. IR (neat): 3221, 3036, 2940, 1561, 1541, 1476, 1418, 1362, 1325, 1219, 1152, 1101, 1083, 995, 966, 888, 848, 801, 744, 728, 682, 631 (cm^–1^). HRMS (ESI-TOF) *m*/*z*: [M + H]^+^ Calcd for C_13_H_12_Br_3_N_2_O_3_S_2_ 544.7834; Found 544.7825. Mp = 157.3 °C (decomposition).

## Supplementary Material



## Data Availability

The data underlying this study are available in the published article and its Supporting Information.

## Notes

Additional optimization tables, reaction profile, green chemistry metrics, crystal data, copies of ^1^H, ^19^F, and ^13^C NMR spectra, and computational details (PDF)
