# Supplementary material for: Sulfoximine N‑Functionalization with N‑Fluorobenzenesulfonamide
Source: J Org Chem. 2025 Oct 29;90(44):15790–803. doi: 10.1021/acs.joc.5c02077 (PMC12604044; doi:10.1021/acs.joc.5c02077)
Supplement: Supplementary file 1 [file jo5c02077_si_001.pdf]

Electronic Supporting Information (ESI)

Sulfoximine *N*-Functionalization with *N*-Fluorobenzenesulfonamide

Žan Testen, Črtomir Podlipnik and Marjan Jereb*

University of Ljubljana, Faculty of Chemistry and Chemical Technology, Večna pot 113, 1000 Ljubljana, Slovenia

Table of contents

Optimization tables S3

Reaction profile S4

Green chemistry metrics S5

NMR structure determination S6

Crystal data S7

Copies of NMR spectra S9

Computational details S60

Calculated energies and cartesian coordinates for different species S61

Rearrangement M02 -> M03 ..... S77

Reaction Profile Calculations S77

References S78

Optimization tables

Table S1. ESI table 1; Screening of Inorganic Bases[a]

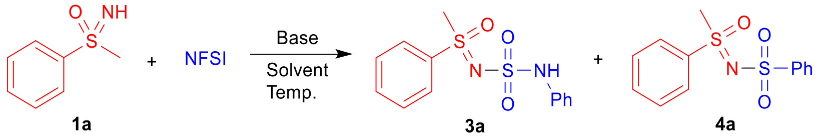

Entry	Base	Solvent	Equiv. of base	Temp.	Equiv. of reactant	Conversion[b]	
						**3a**	**4a**
1	LDA[c]	THF	1.5	0 °C	1.2	26%	/
2	LiTMP[c]	THF	1.5	0 °C	1.2	11%	/
3	PhLi[c]	THF	1.5	-78 °C	1.2	65%	/
4	PhC≡CLi[c]	THF	1.5	-78 °C	1.2	59%	/
5	*n*-BuLi[c]	THF	1.5	-78 °C	1.2	84%	/
6	*n*-BuLi[c]	THF	1.5	-78 °C	1.2 [d]	61%	/
7	*n*-BuLi[c]	Et_2O	1.1	-78 °C	2	41%	trace
8	*n*-BuLi[c]	PhMe	1.1	-78 °C	2	42%	trace
9	*sec*-BuLi[c]	THF	1.5	-78 °C	1.2	61%	/
10	NaOH	DMSO	2	r.t.	1.2 [d]	/	24%
11	NaH	DCM	1.5	r.t.	1.2 [d]	31%	60%
12	NaH	THF	1.5	r.t.	1.2 [d]	36%	30%
13	*t*-BuOK	neat	1.2	70 °C	2 [d]	65%[e]	35%

[a] Reaction conditions: A flask was charged with **1a** (0.1 mmol) and solvent (1 mL). The base was added at the corresponding temperature and then NFSI was added as a solution. The reaction was then slowly warmed to r.t. and stirred for 16 h. [b] Determined by ^{1}H NMR. [c] Reactions involving lithium bases were carried out under an inert atmosphere (Ar), with anhydrous solvents and in dried glassware. [d] NFSI added as solid. [e] Highly exothermic reaction.

Table S2. ESI table 2; Screening of Solvents for *i*-Pr_2NH[a]

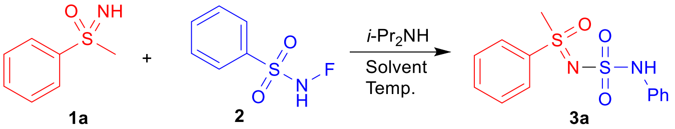

Entry	Solvent	Equiv. of base	Temp.	Equiv. of reactant	NMR yield[b]
1	DCM	2	r.t.	1.5	69%
2	DCM	2	40 °C	1.5	73%
3	MeCN	2	40 °C	1.5	65%
4	MeCN	2	60 °C	1.5	69%
5	THF	2	40 °C	1.5	40%[c]
6	MeOH	2	40 °C	1.5	/

[a] Reaction conditions: A flask was charged with **1a** (0.1 mmol), **2**, base and stirred for 16 h.
[b] Determined by ^{1}H NMR with internal standard (1,3,5-trimethoxybenzene). [c] Many by-products.

Reaction profile

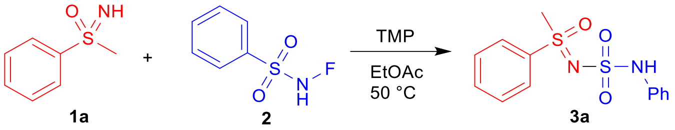

A 100 mL flask was charged with sulfoximine **1** (6 mmol, 1 g), EtOAc (40 mL) and *N*-fluorobenzenesulfonamide (**2**) (2 equiv., 2.26 g). While the reaction mixture was stirred vigorously, TMP (1.25 equiv., 1.28 mL) was added and the reaction was heated to 50 °C using a sand bath. Aliquots were taken in 30 min intervals and examined by ^{1}H NMR to determine the conversion.

Time [min]	Conversion
30	9%
60	43%
90	83%
120	93%
180	97%
240	99%

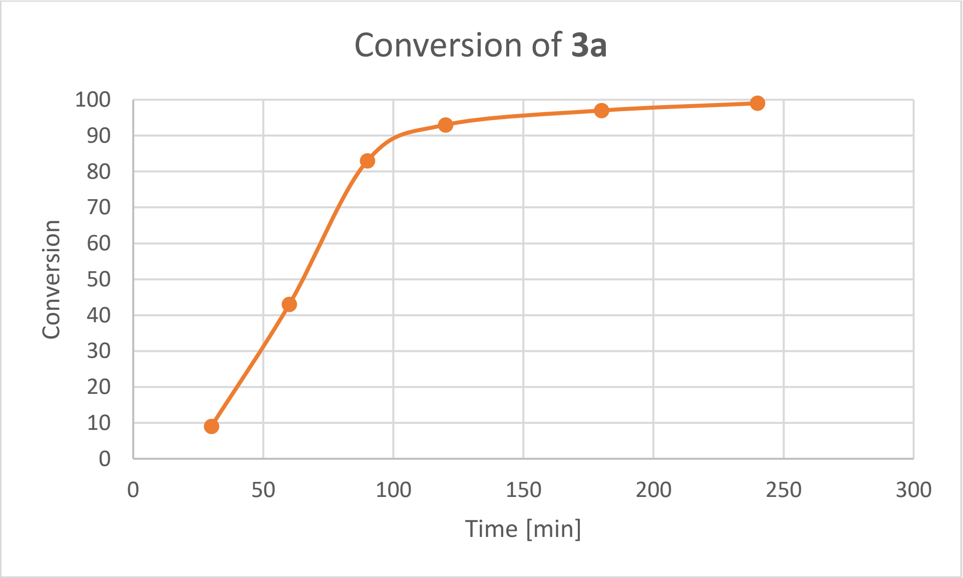

Green chemistry metrics

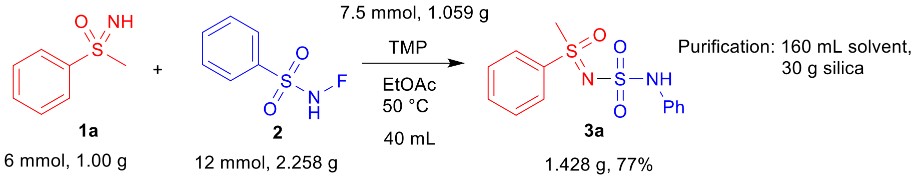

E-factor (simple: no solvents):[1,2]

Amount of reactants: 1.00 g + 2.258 g + 1.059 g = 4.317 g

Amount of waste from reaction: 4.317 − 1.428 = 2.889 g

(Amount of waste / amount of product) = 2.889 g / 1.428 g = **2.023**

E-factor (more comprehensive):

Amount of reactants: 1.00 g + 2.258 g + 1.059 g = 4.317 g

Amount of waste from reaction: 4.317 − 1.428 = 2.889 g

Amount of waste from purification: 20 mL of EtOAc (d = 0.902 g/mL) = 18.04 g (10% of the 200 mL solvents used and recycled and 30 g of silica.

Amount of waste: 2.889 g + 18.04 g + 30 g = 50.929 g

(Amount of waste / amount of product) = 50.929 g / 1.428 g = **35.66**

EcoScale:[3]

Points deducted for yield (−11.5), EtOAC is flammable (−5), heating (-3), classic chromatography (-10). Final score: **70.5.**

NMR structure determination

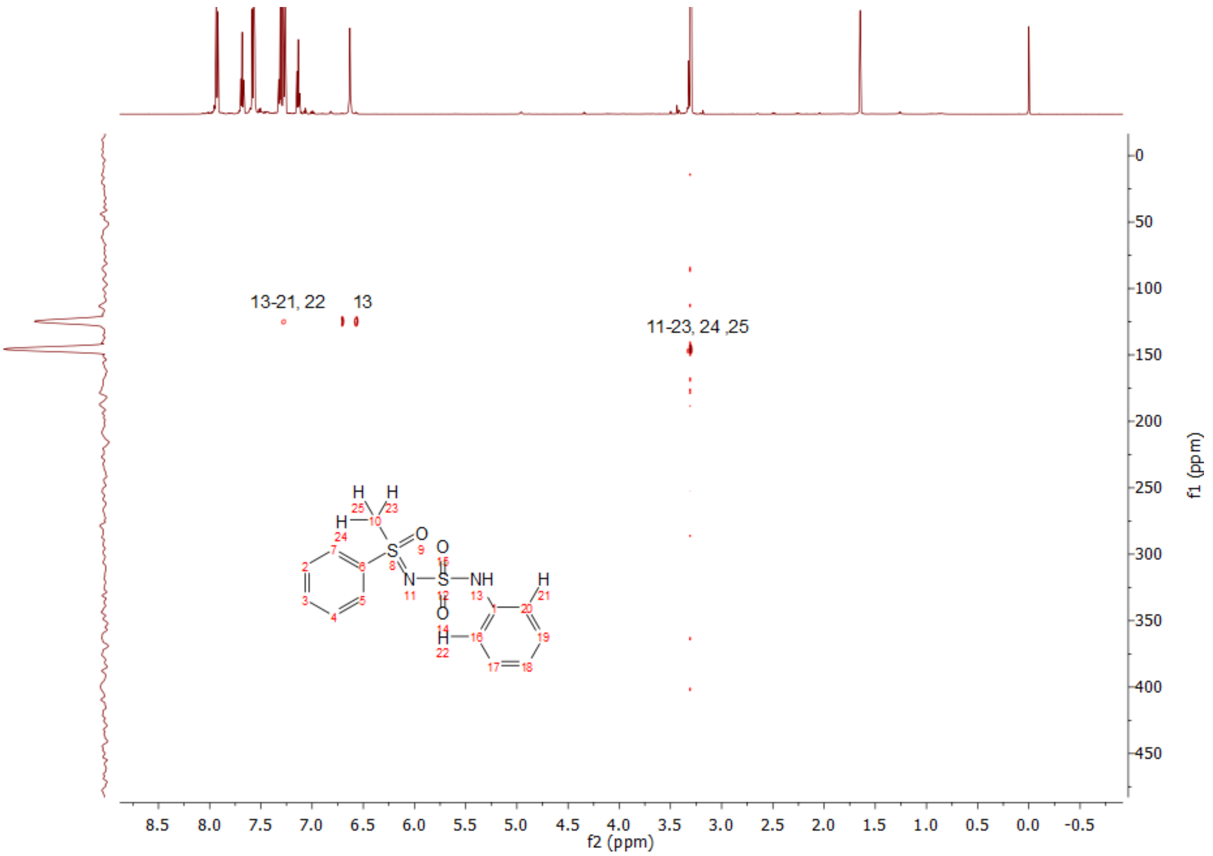

Crystal data

Single-crystal X-ray diffraction data were collected at 150 K on an Agilent Technologies SuperNova Dual diffractometer with an Atlas detector using monochromated Cu-Kα radiation (λ = 1.54184 Å). The data were processed using CrysAlis Pro.[4] Structures were solved by SHELXT[5] program and refined by a full-matrix least-squares procedure based on F2 with SHELXL[5] using the Olex2 program suite.[6] All non-hydrogen atoms were refined anisotropically. Hydrogen atoms were placed in geometrically calculated positions and were refined using a riding model. The figures were produced using Mercury.[7]

Sample preparation: 10 mg of compound **3a** was dissolved in 1 mL of DCM inside a 5 mL glass container. This container was placed inside a larger 20 mL glass container which was filled with 10 mL of *n*-hexane. The large container was closed with a plastic stopper and cooled to 5 °C for 48 h.

	**3a**
**Empirical formula**	$C_{13}H_{13}N_2O_3S_2$
**Formula weight**	309.37
**Temperature/K**	150.00(10)
**Crystal system**	monoclinic
**Space group**	Pc
**a/Å**	5.51291(15)
**b/Å**	9.7980(2)
**c/Å**	12.9951(3)
**α/°**	90
**β/°**	100.041(3)
**γ/°**	90
**Volume/Å^3**	691.18(3)
**Z**	2
**ρ_{calc}g/cm^3**	1.487
**μ/mm^{-1}**	3.581
**F(000)**	322.0
**Crystal size/mm^3**	0.3 × 0.1 × 0.03
**Radiation**	Cu Kα (λ = 1.54184)
**2Θ range for data collection/°**	9.026 to 149.62
**Index ranges**	-4 ≤ h ≤ 6, -12 ≤ k ≤ 11, -16 ≤ l ≤ 16
**Reflections collected**	5556
**Independent reflections**	2072 [R_{int} = 0.0346, R_{sigma} = 0.0421]
**Data/restraints/parameters**	2072/2/182
**Goodness-of-fit on F^2**	1.029
**Final R indexes [I>=2σ (I)]**	R_1 = 0.0667, wR_2 = 0.1719
**Final R indexes [all data]**	R_1 = 0.0701, wR_2 = 0.1760
**Largest diff. peak/hole / e Å^{-3}**	1.64/-0.68

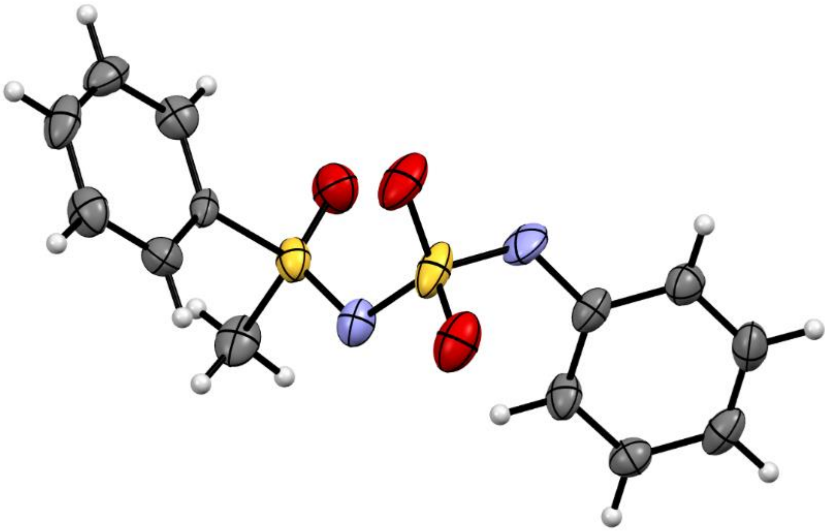

Figure S1. Crystal Structure of Product **3a**, Elipsoids Depicted at 50% Probability

CCDC 2454111 contains the supplementary crystallographic data for this paper, including structure factors and refinement instructions. These data can be obtained free of charge from The Cambridge Crystallographic Data Centre, 12 Union Road, Cambridge CB2 1EZ, UK (fax: +44(1223)-336-033; e-mail: deposit@ccdc.cam.ac.uk), or via https://www.ccdc.cam.ac.uk/structures.

Copies of NMR spectra

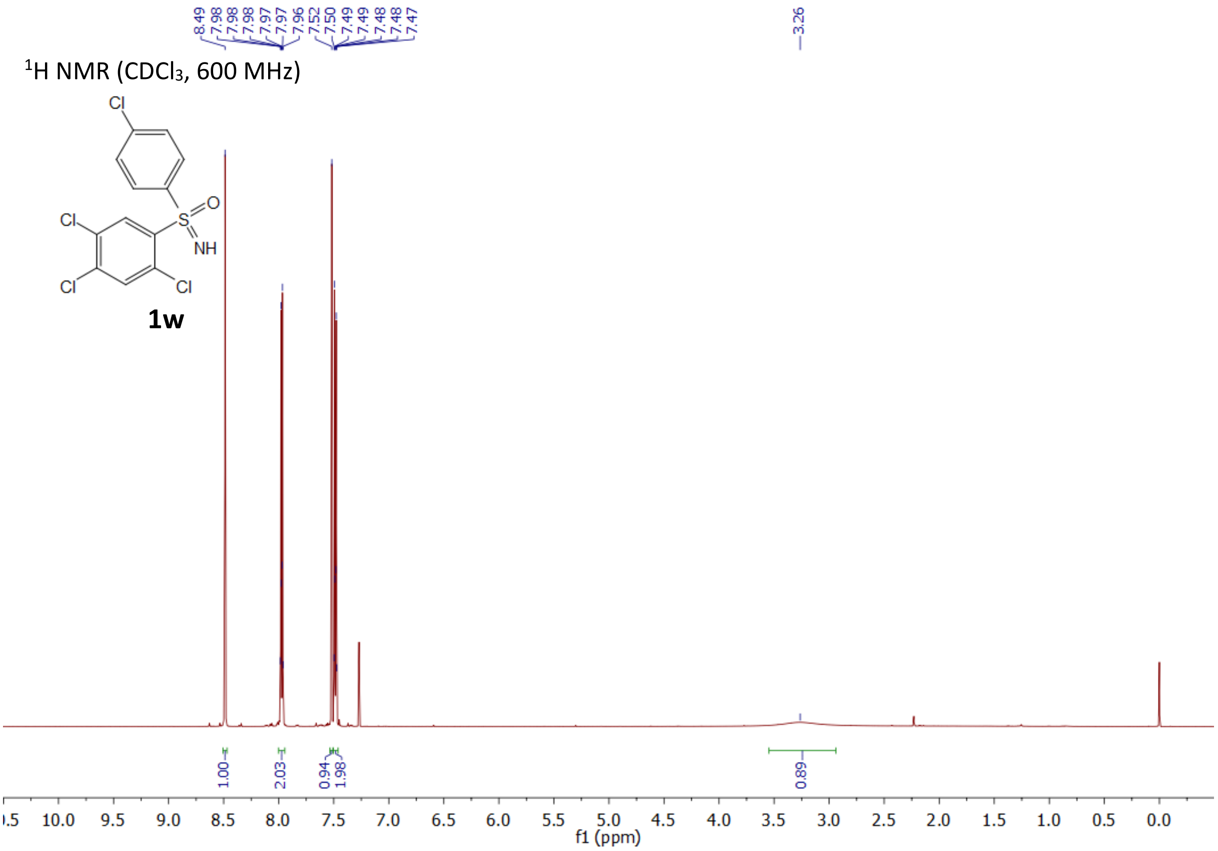

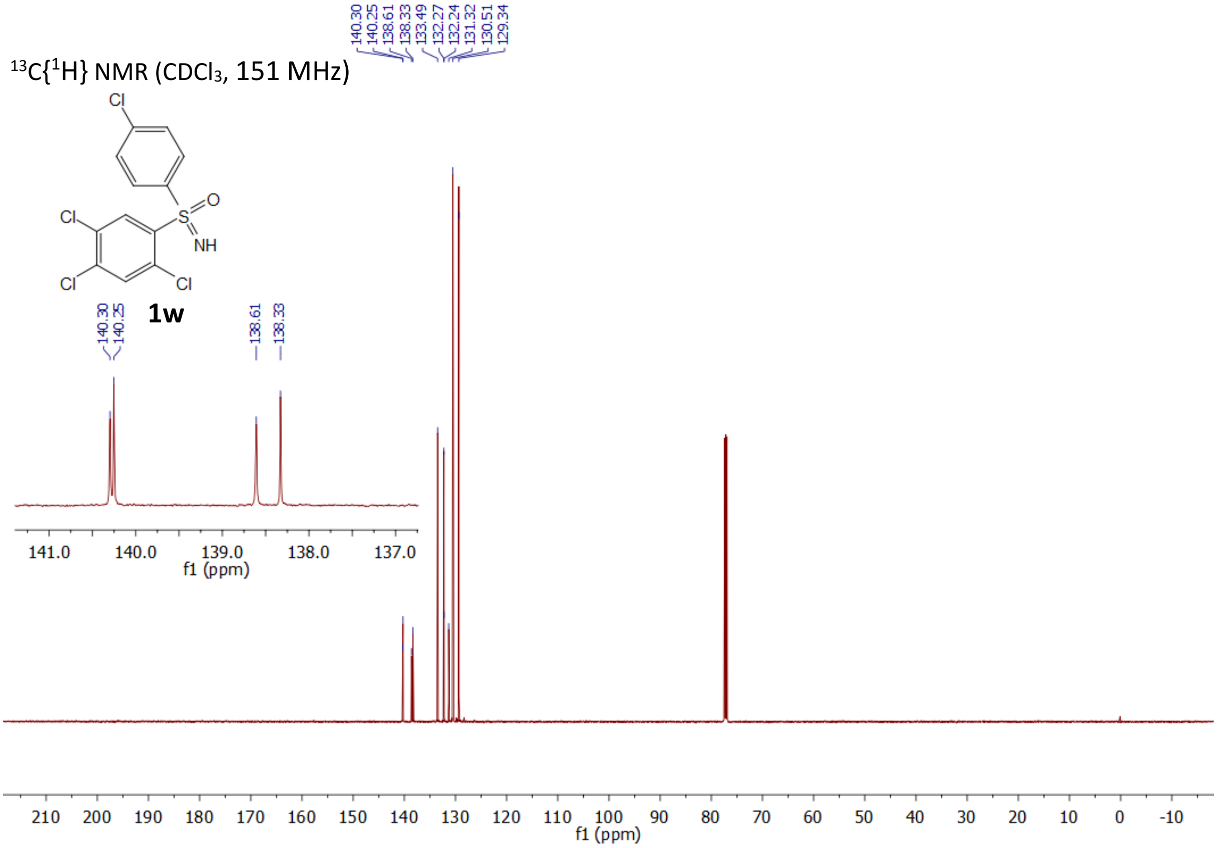

^{1}H NMR ($CDCl_3$, 600 MHz)

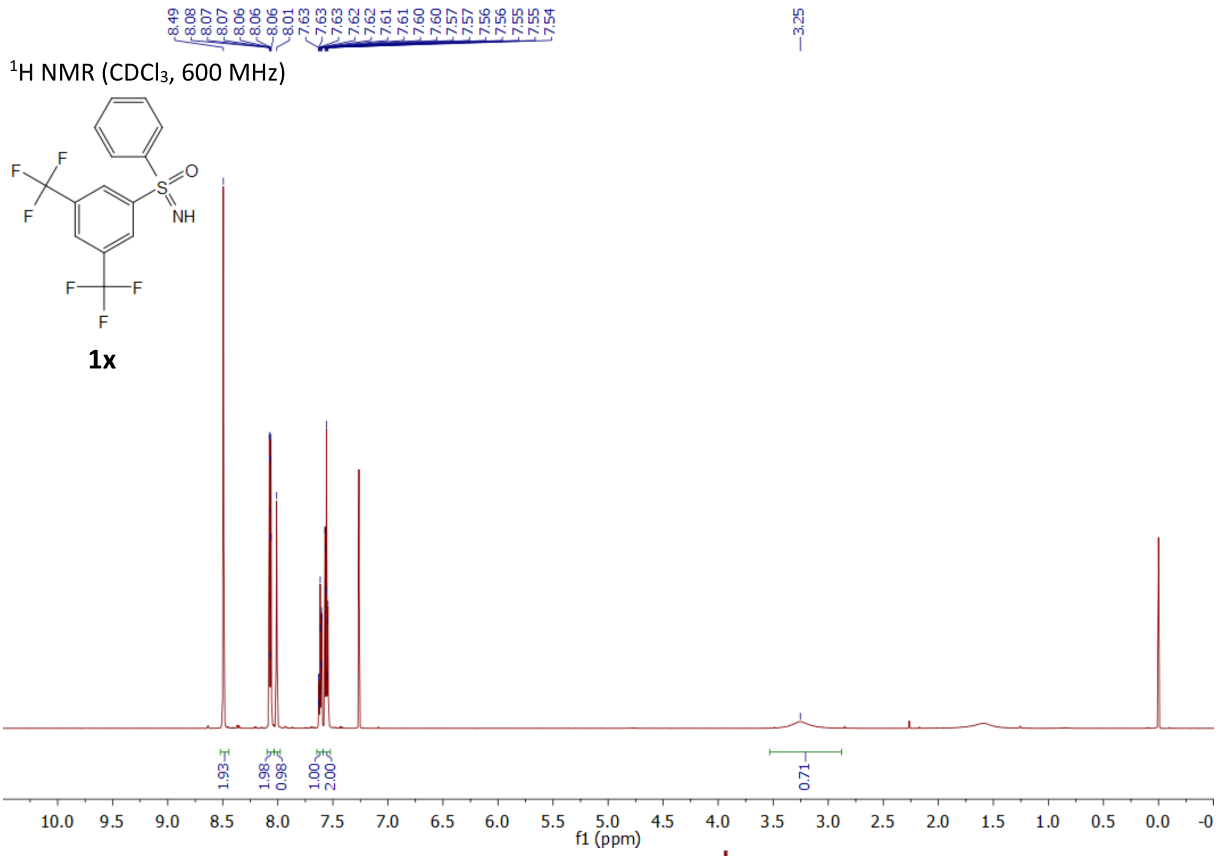

^{13}C{^{1}H} NMR ($CDCl_3$, 151 MHz)

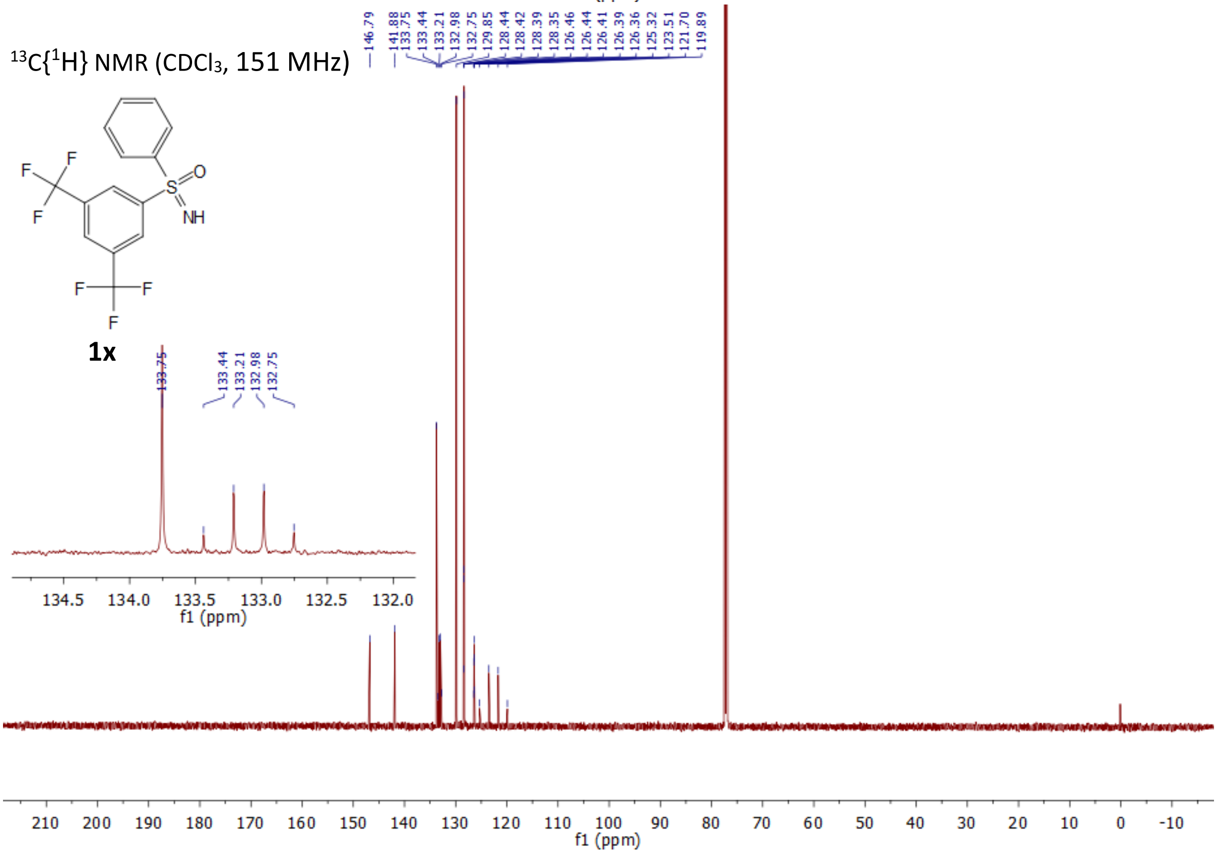

^{19}F NMR ($CDCl_3$, 565 MHz)

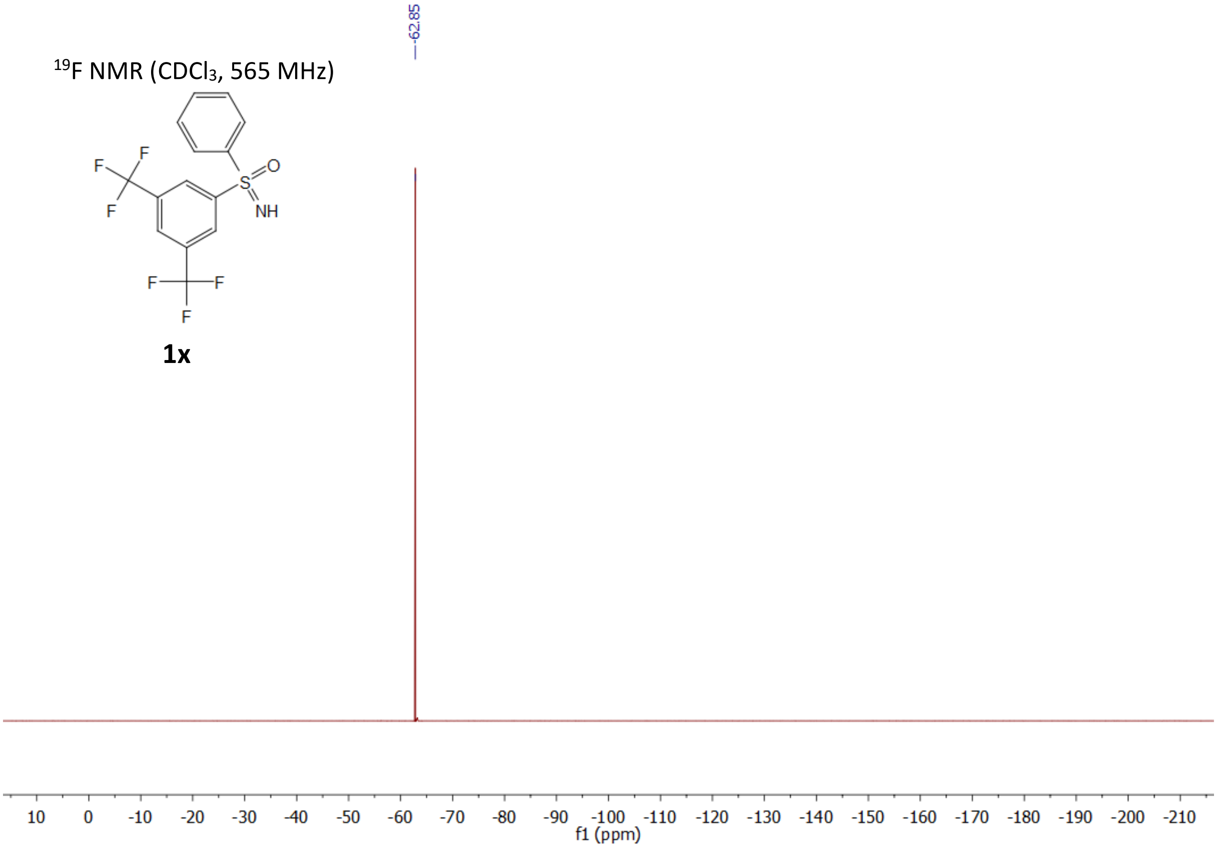

^{1}H NMR ($CDCl_3$, 600 MHz)

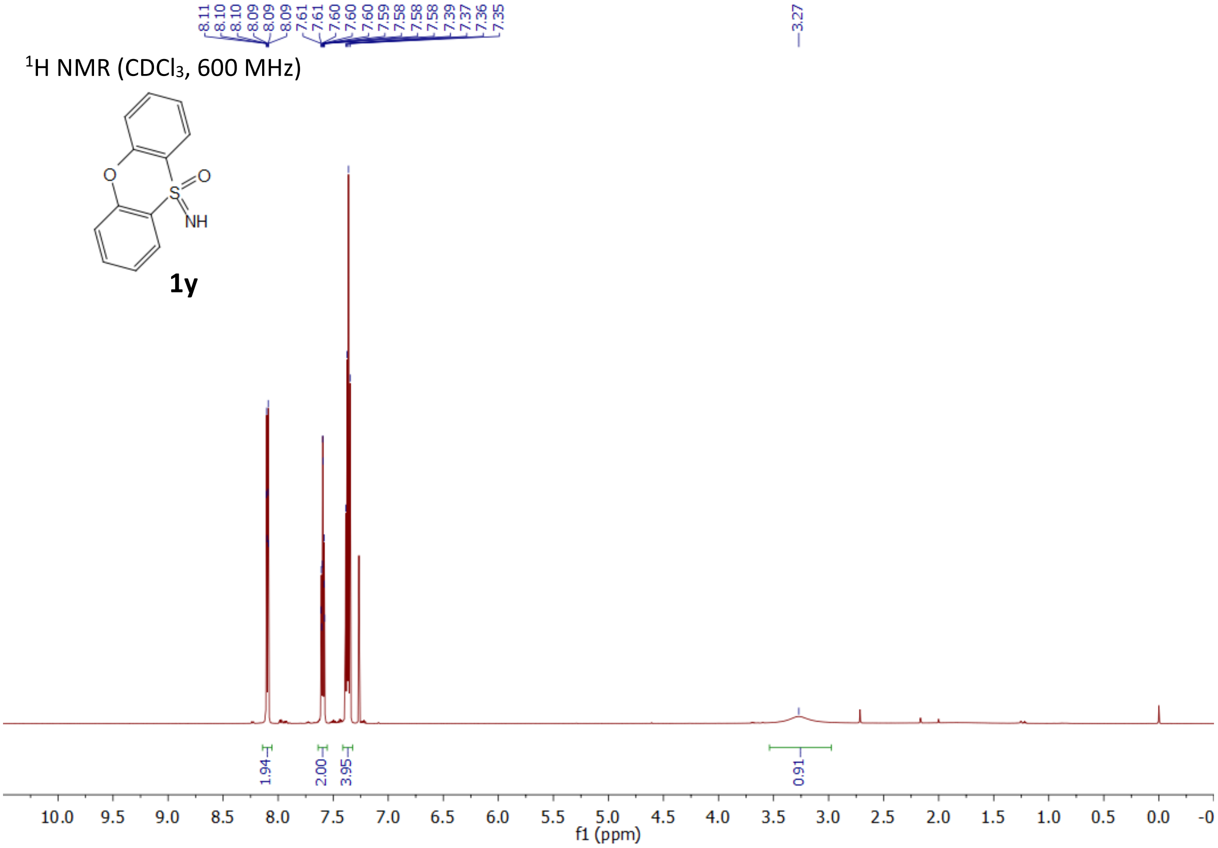

1y

^{13}C{^{1}H} NMR ($CDCl_3$, 151 MHz)

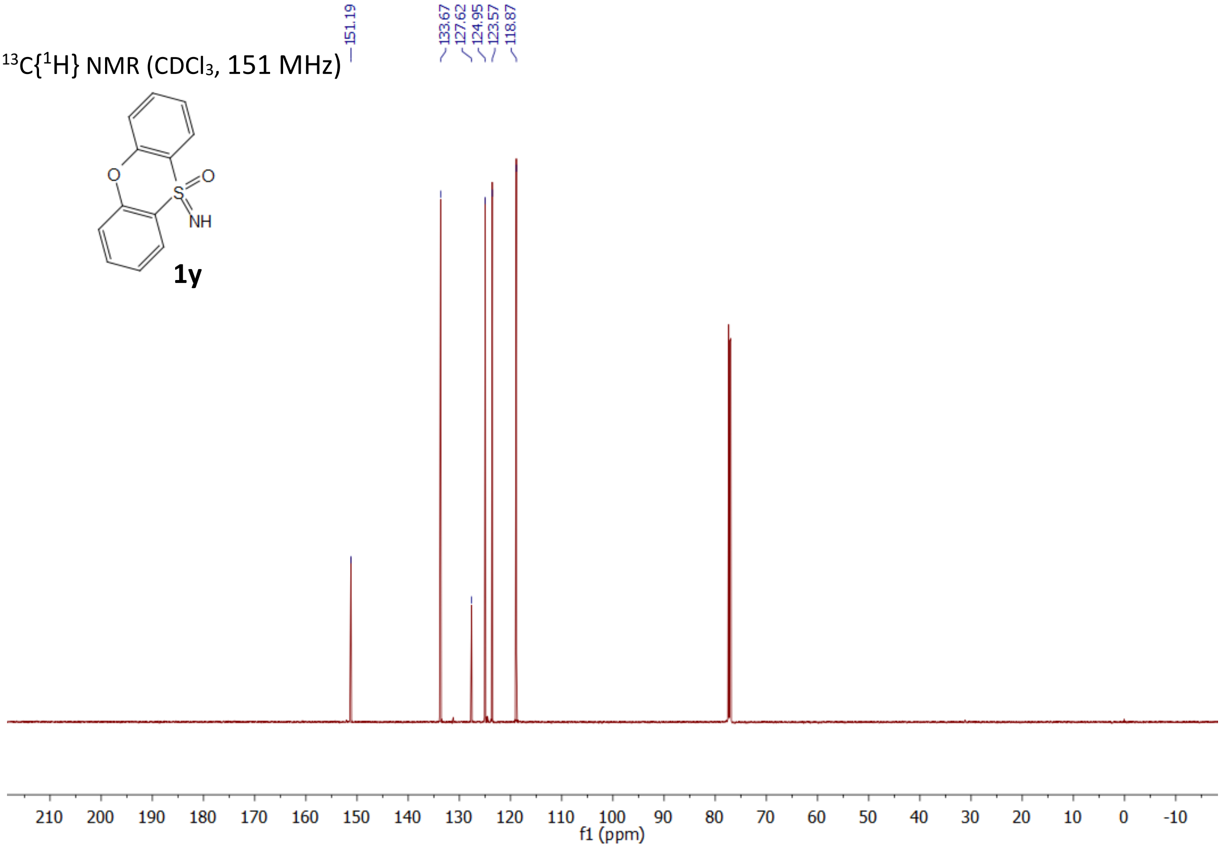

1y

^{1}H NMR ($CDCl_3$, 500 MHz)

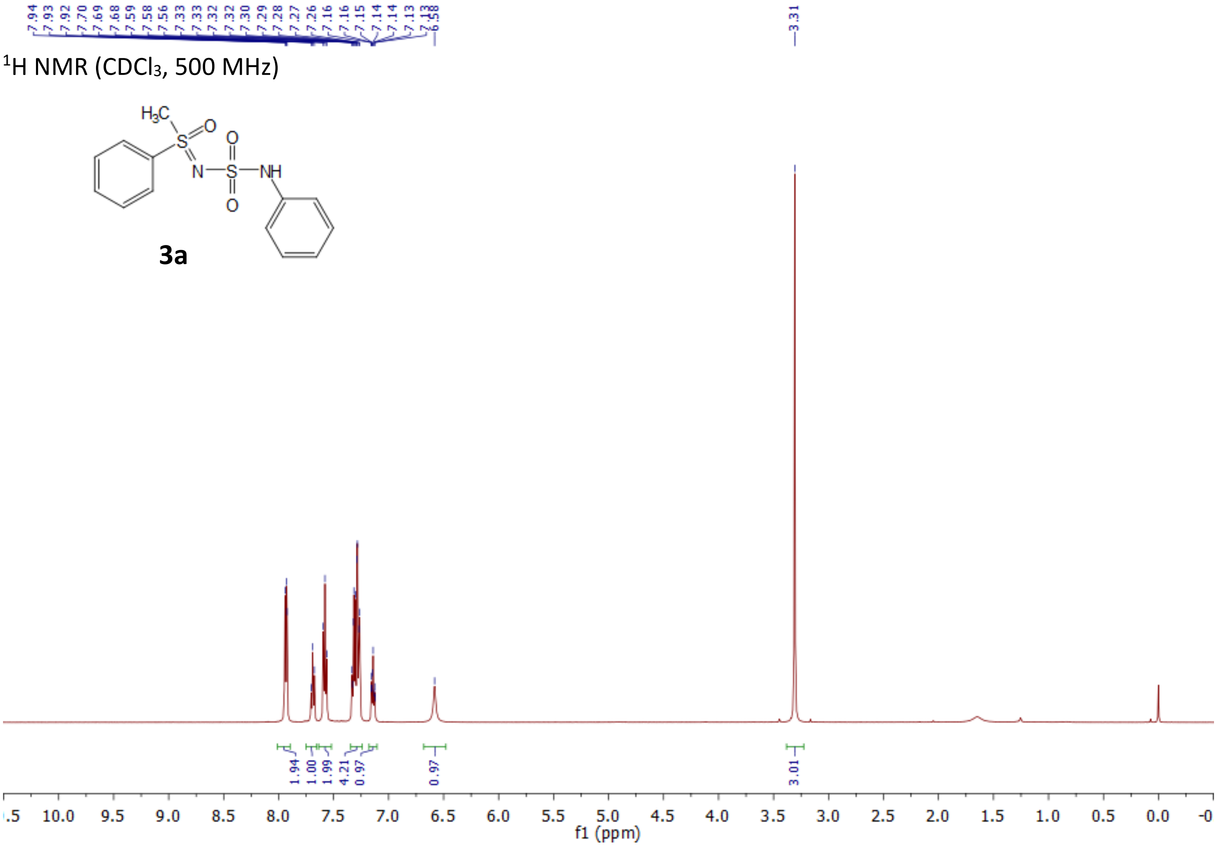

$^{13}C\{^{1}H\}$ NMR ($CDCl_3$, 126 MHz)

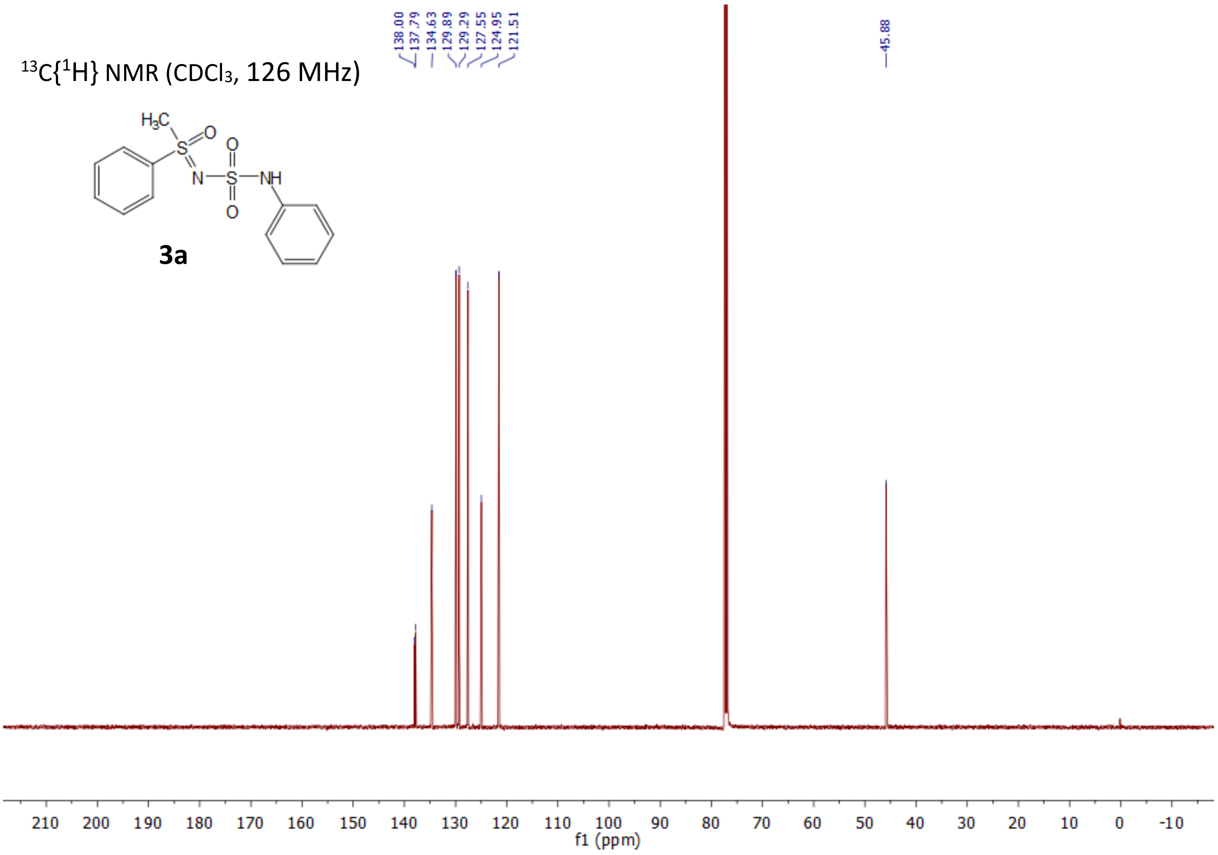

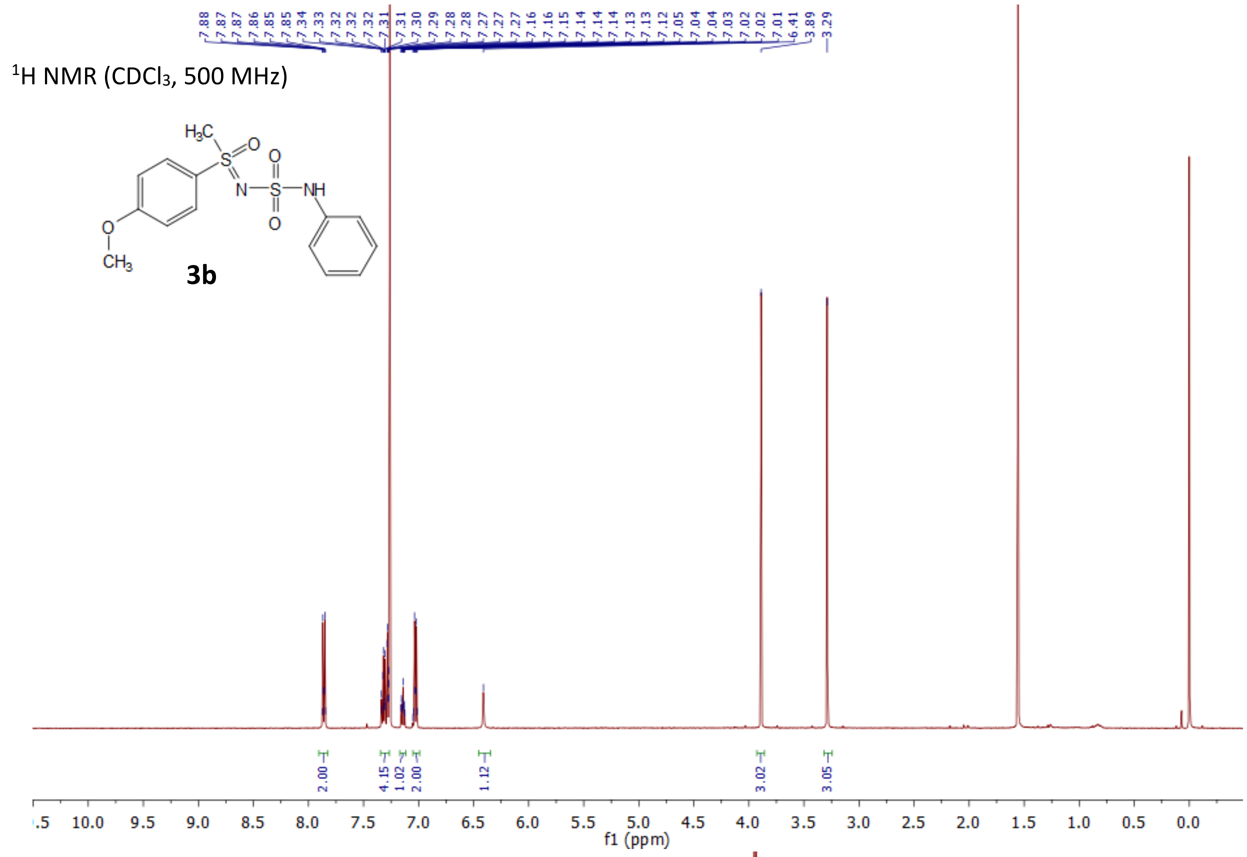

7.88 7.87 7.87 7.86 7.85 7.85 7.34 7.33 7.32 7.32 7.32 7.32 7.31 7.31 7.30 7.29 7.28 7.28 7.27 7.27 7.27 7.16 7.16 7.15 7.14 7.14 7.14 7.13 7.13 7.12 7.05 7.04 7.04 7.03 7.02 7.02 7.01 6.41 3.89 3.29
^1H NMR (CDCl₃, 500 MHz)
H₃C
O
O
N
S
NH
O
O
CH₃
3b
2.00 4.15 1.02 2.00 1.12 3.02 3.05
.5 10.0 9.5 9.0 8.5 8.0 7.5 7.0 6.5 6.0 5.5 5.0 4.5 4.0 3.5 3.0 2.5 2.0 1.5 1.0 0.5 0.0
f1 (ppm)

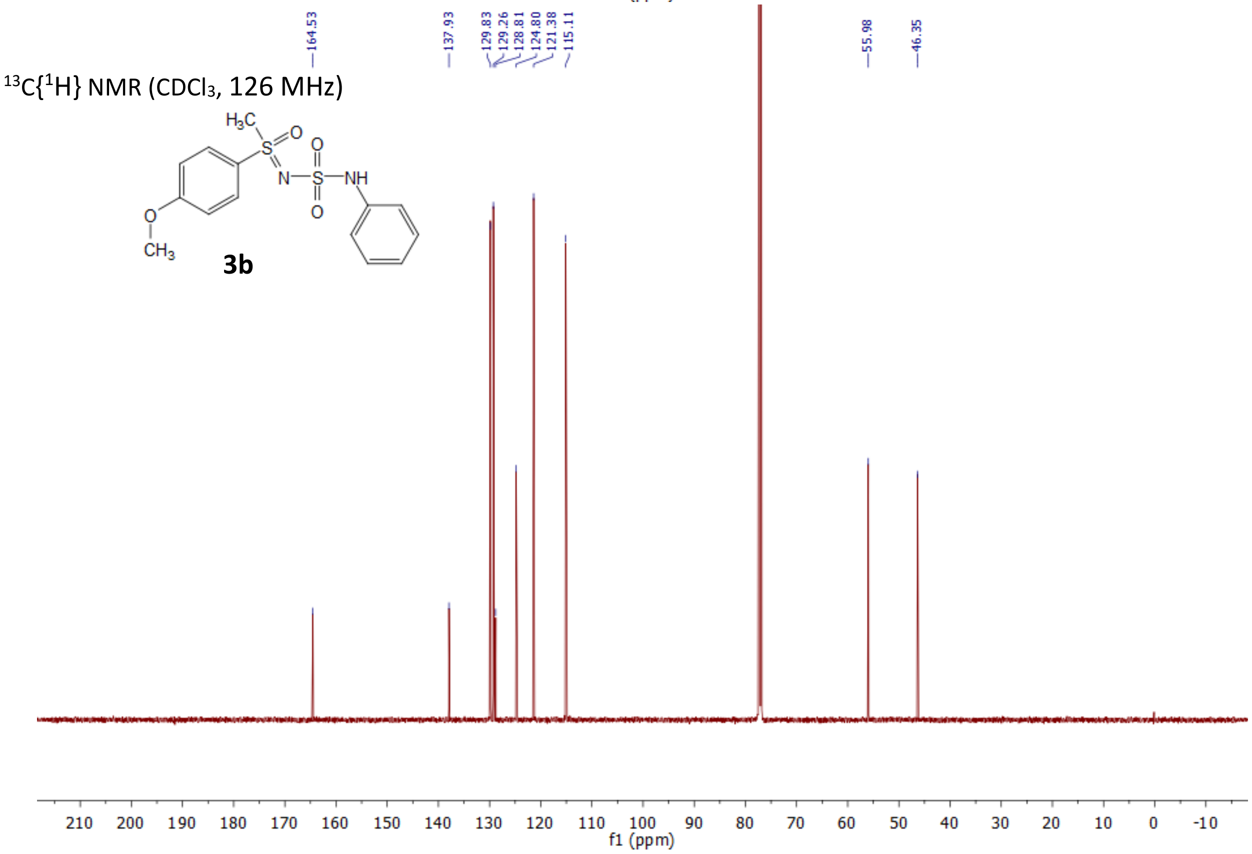

164.53 137.93 129.83 129.26 128.81 124.80 121.38 115.11 55.98 46.35
$^{13}C\{^1H\}$ NMR (CDCl₃, 126 MHz)
H₃C
O
O
N
S
NH
O
O
CH₃
3b
210 200 190 180 170 160 150 140 130 120 110 100 90 80 70 60 50 40 30 20 10 0 -10
f1 (ppm)

^1H NMR ($CDCl_3$, 500 MHz)

3c

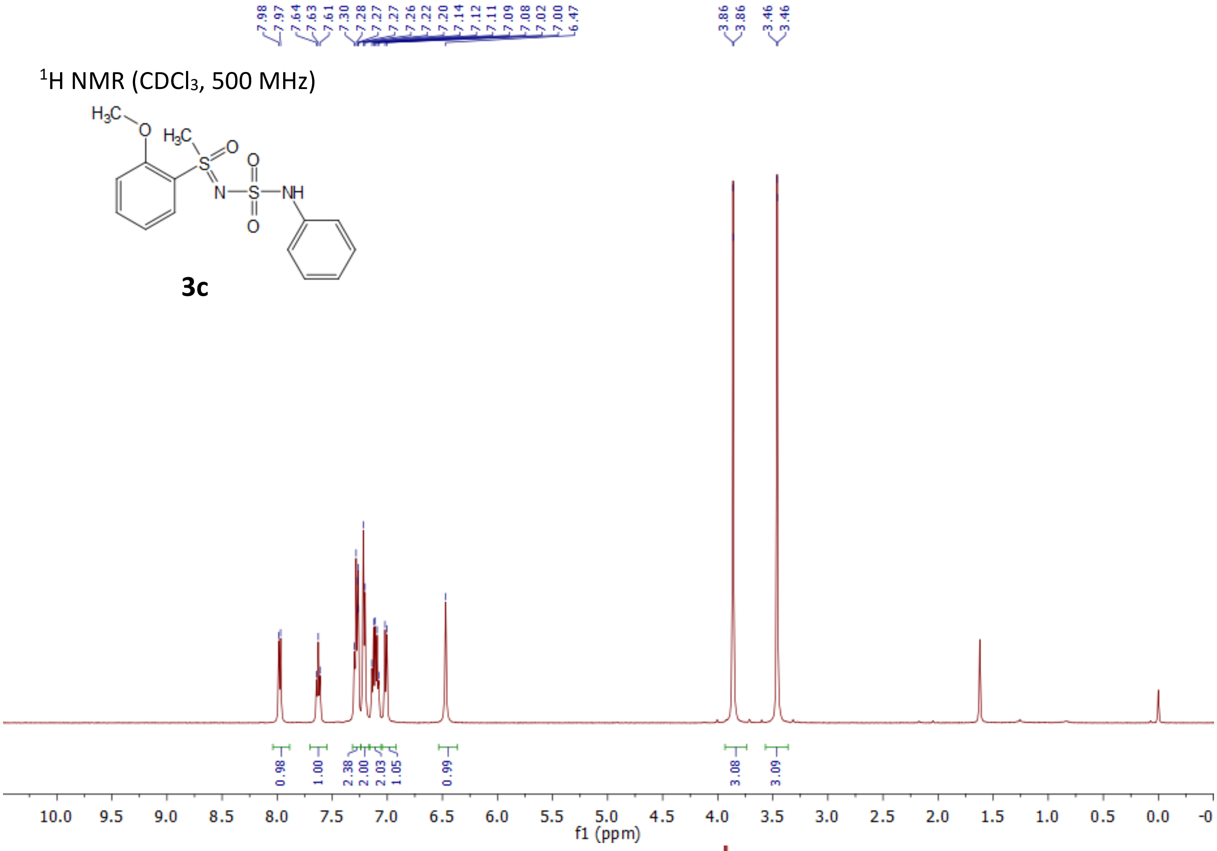

$^{13}C\{^1H\}$ NMR ($CDCl_3$, 126 MHz)

3c

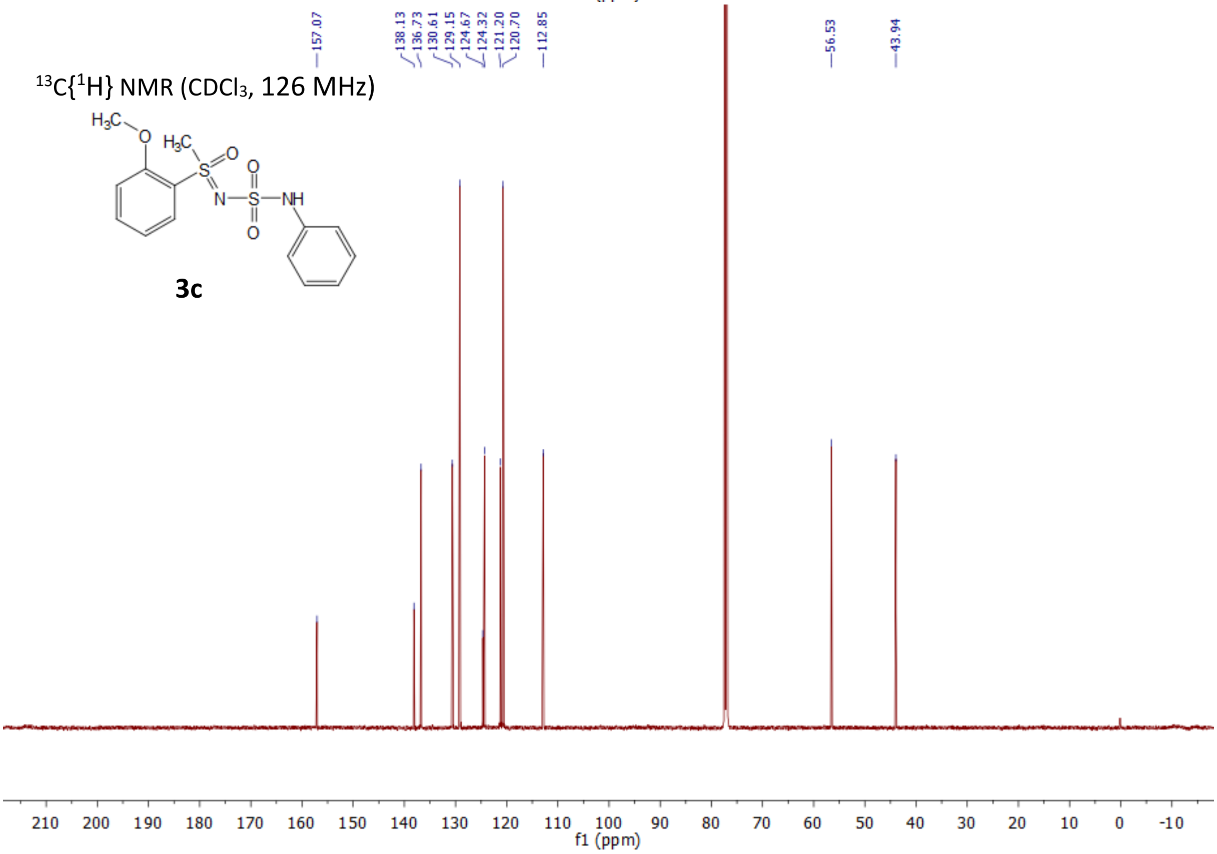

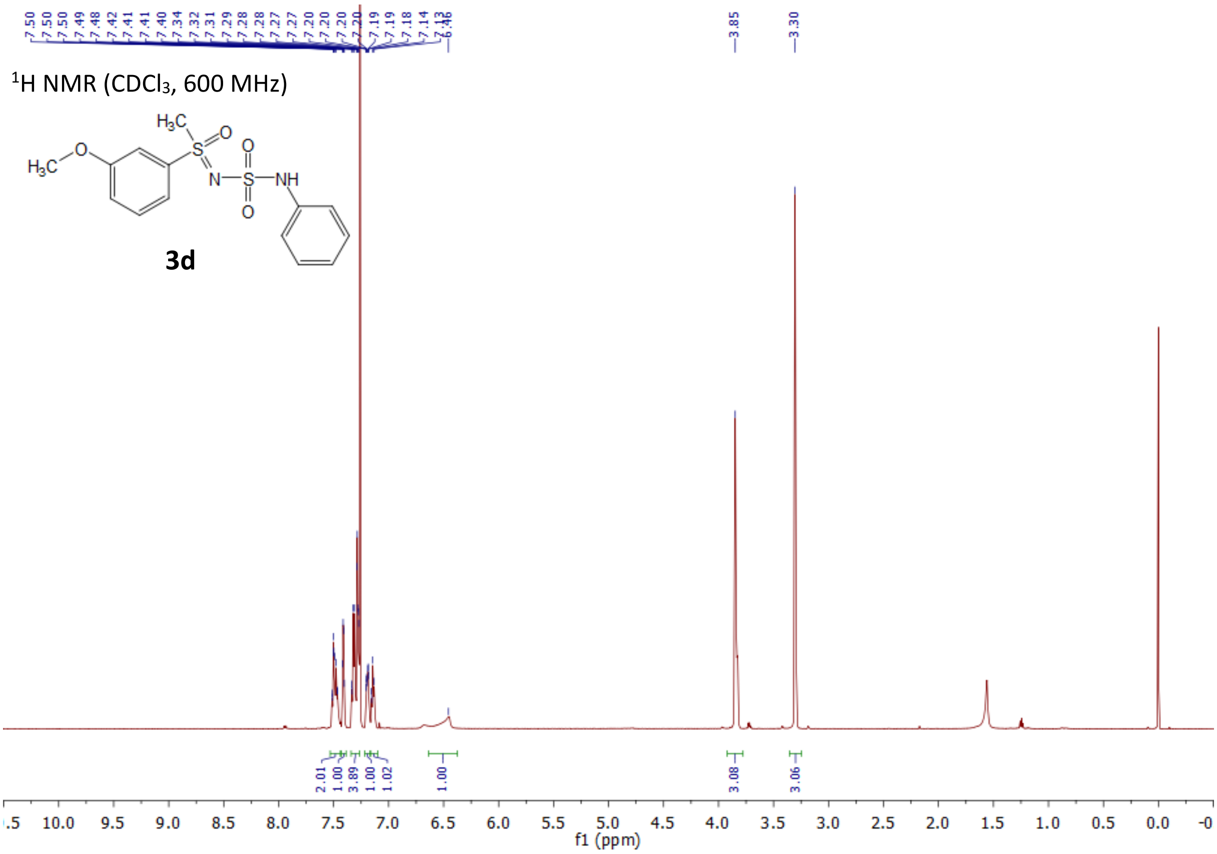
1H NMR (CDCl3, 600 MHz)
3d
7.50
7.50
7.50
7.49
7.48
7.42
7.41
7.41
7.40
7.34
7.32
7.31
7.29
7.28
7.28
7.27
7.27
7.20
7.20
7.20
7.20
7.19
7.19
7.18
7.14
7.13
6.46
3.85
3.30
2.01
1.00
3.89
1.00
1.02
1.00
3.08
3.06
f1 (ppm)

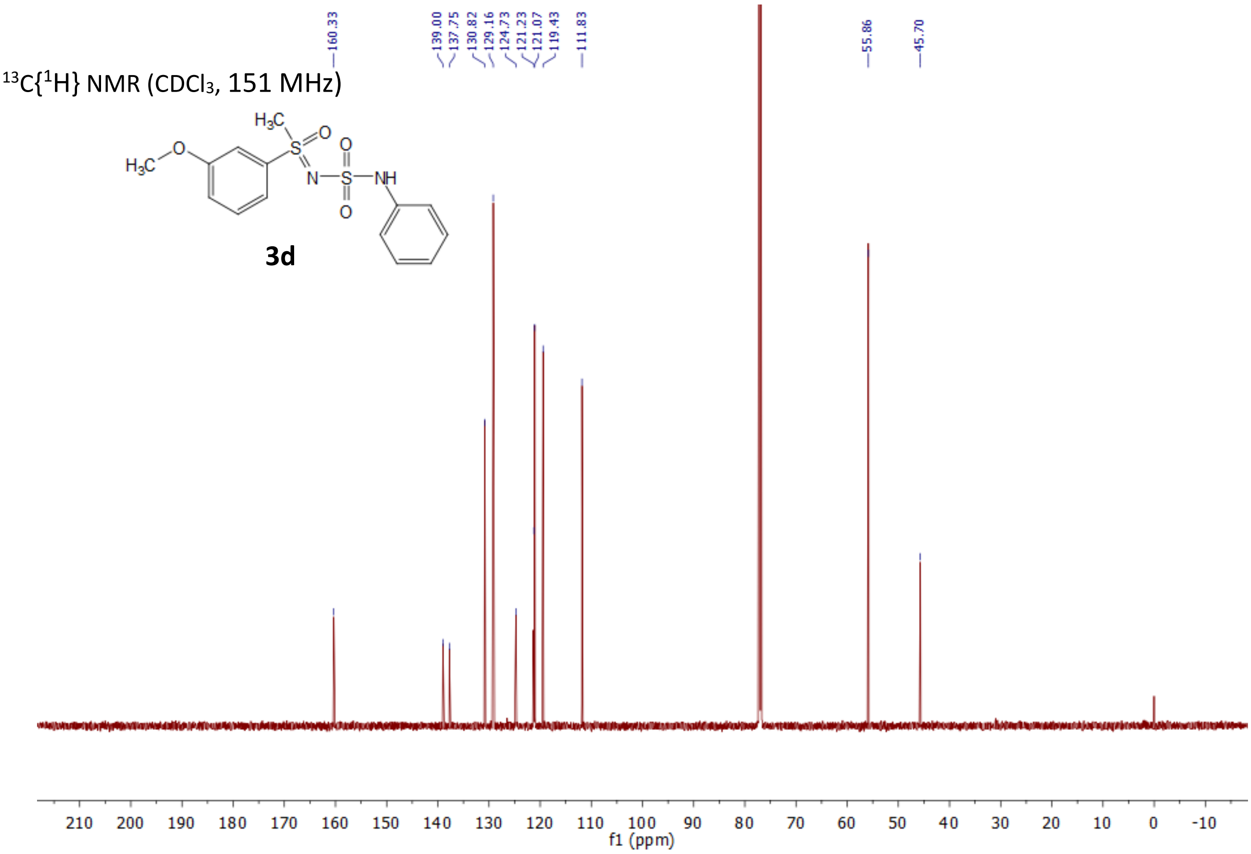
13C{1H} NMR (CDCl3, 151 MHz)
3d
160.33
139.00
137.75
130.82
129.16
124.73
121.23
121.07
119.43
111.83
55.86
45.70
f1 (ppm)

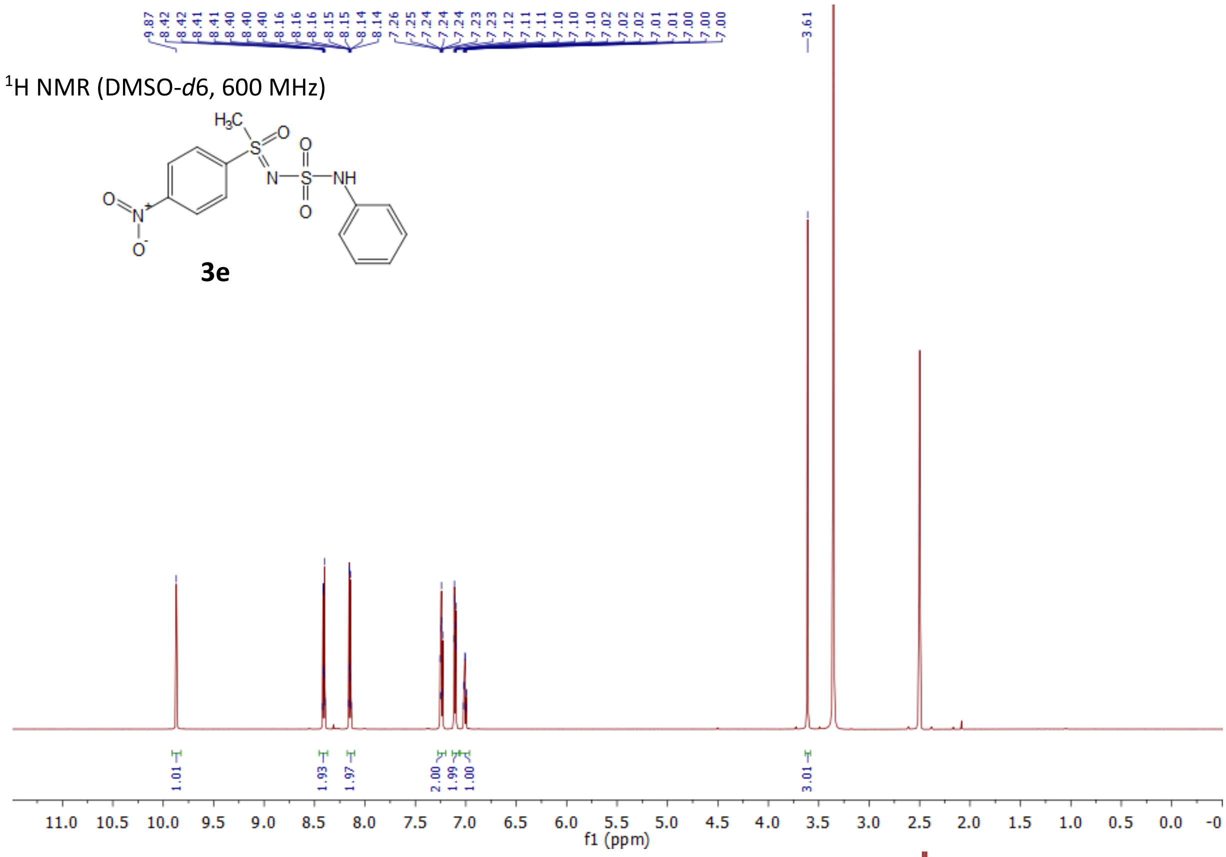
1H NMR (DMSO-d6, 600 MHz)
3e
f1 (ppm)

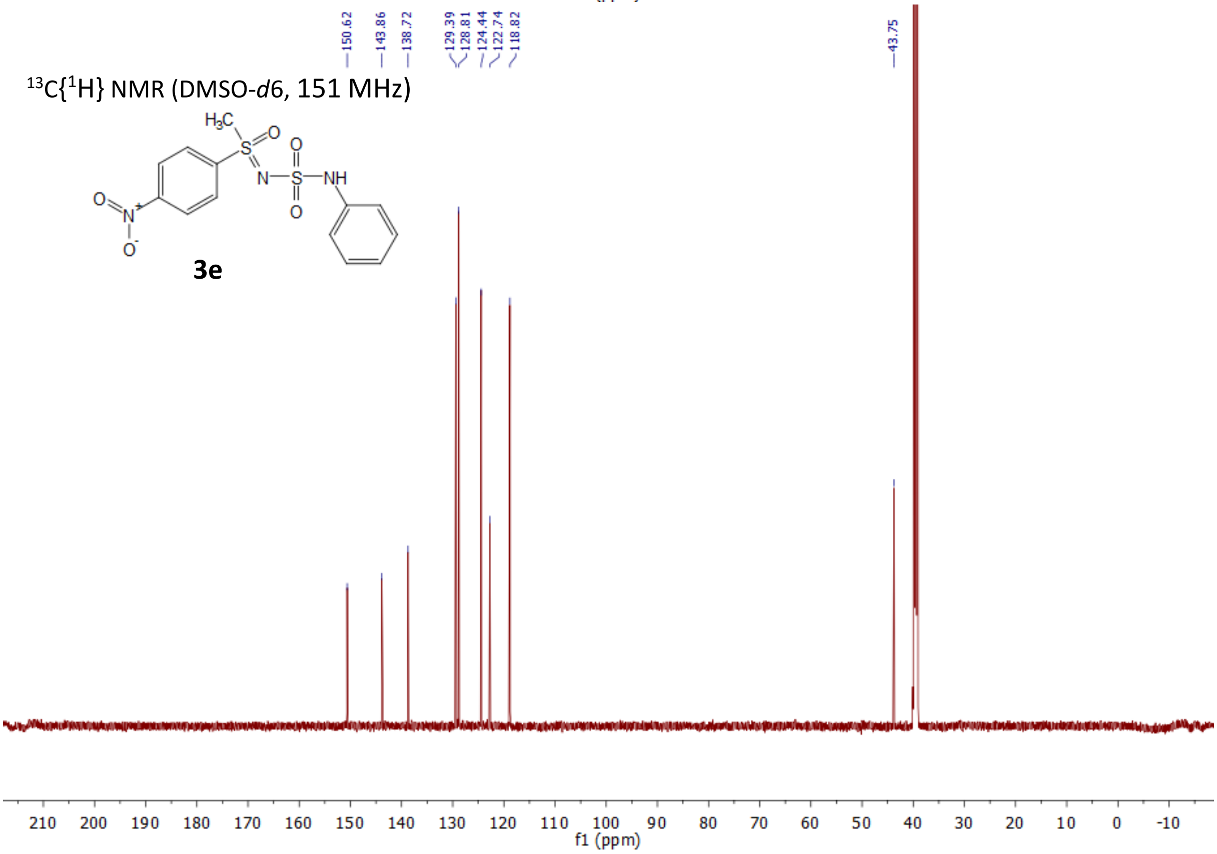
13C{1H} NMR (DMSO-d6, 151 MHz)
3e
f1 (ppm)

^{1}H NMR ($CDCl_3$, 500 MHz)

3f

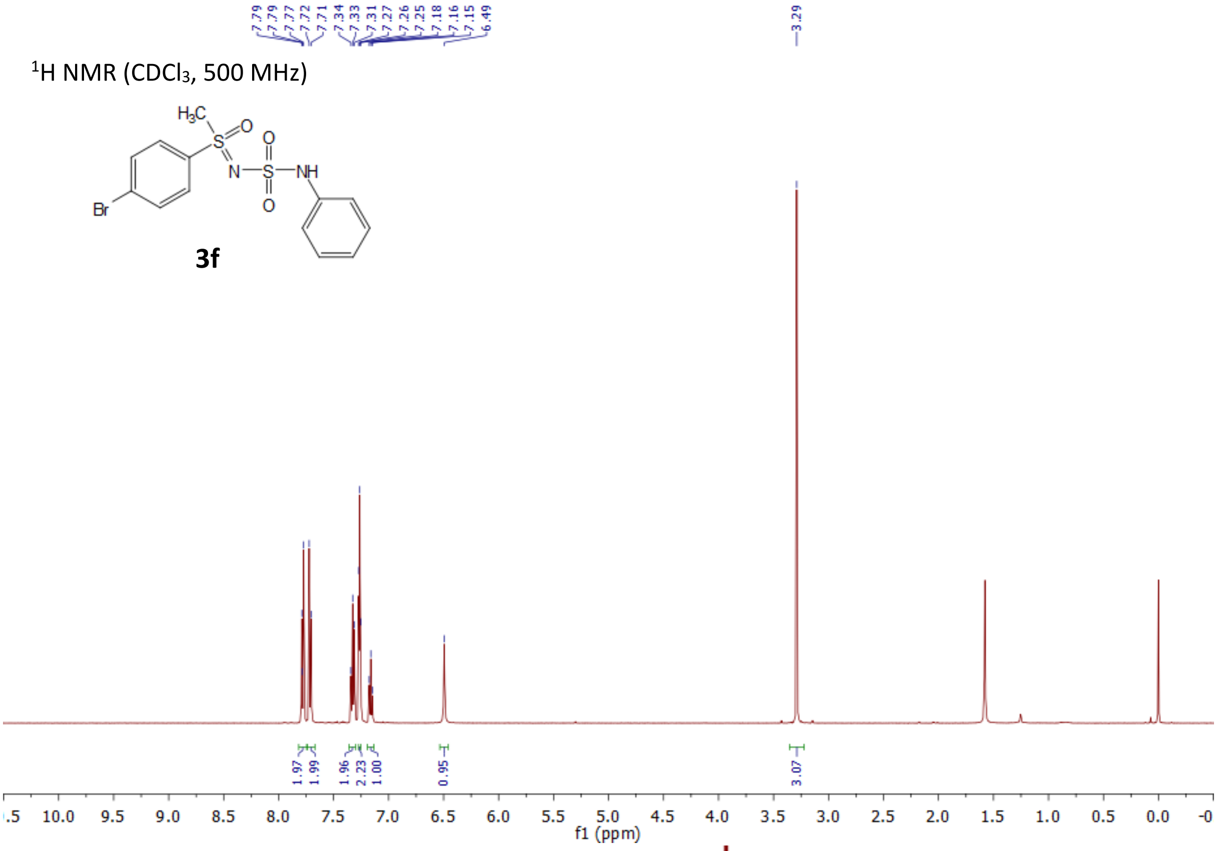

^{13}C{^{1}H} NMR ($CDCl_3$, 126 MHz)

3f

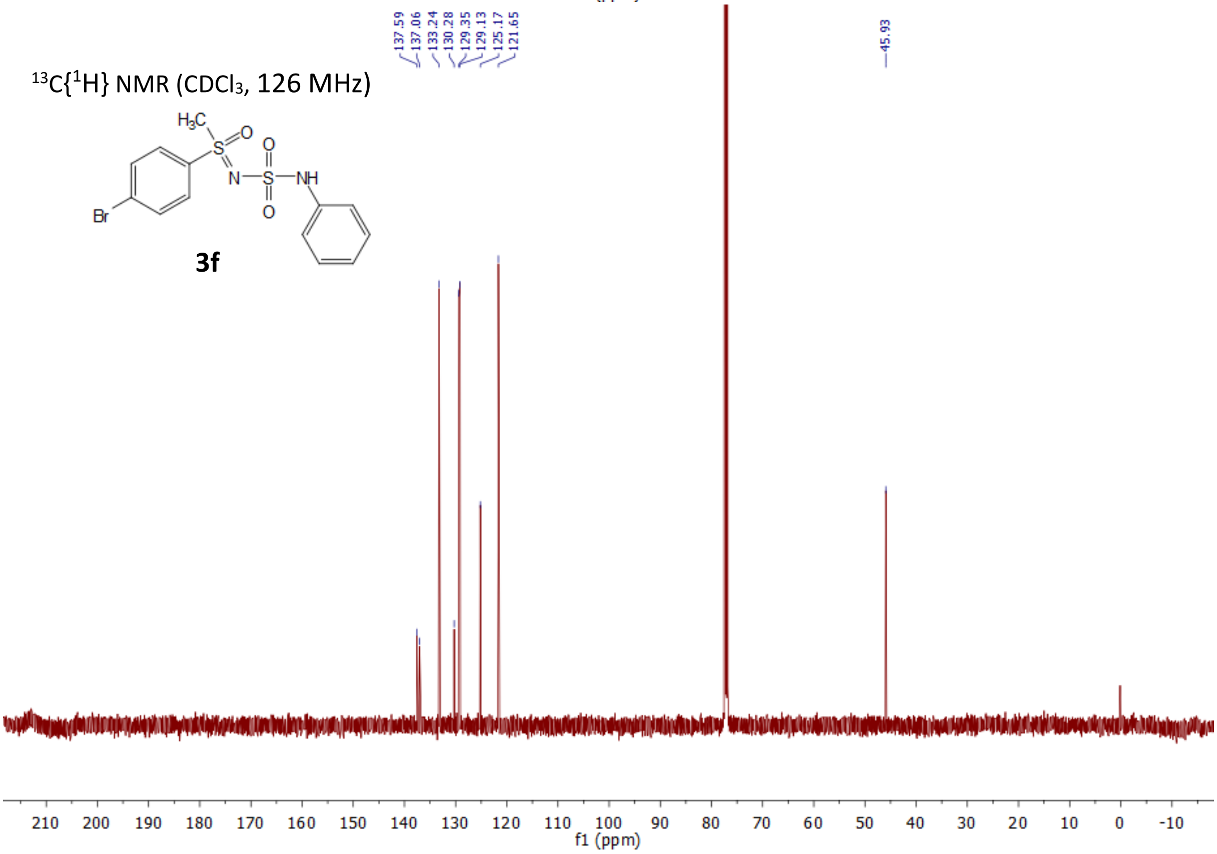

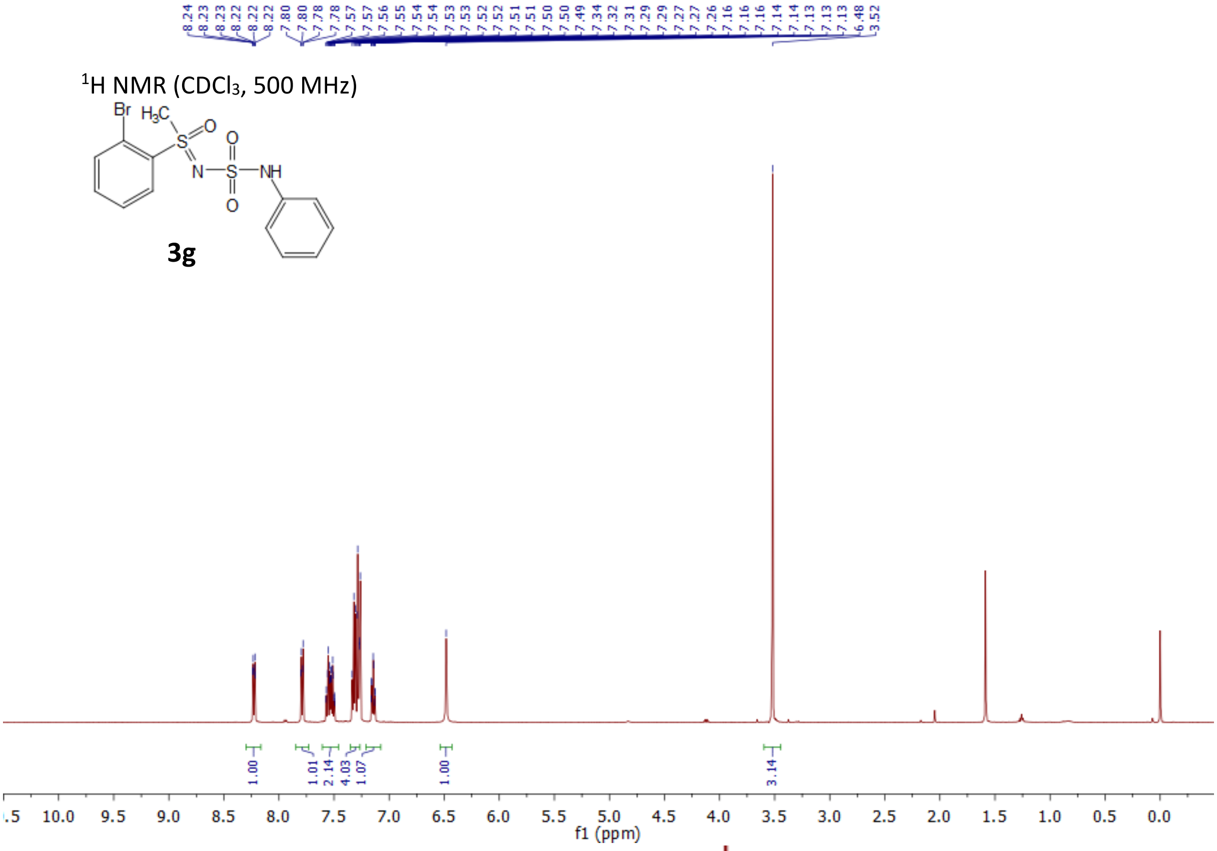

8.24 8.23 8.23 8.22 8.22 8.22 7.80 7.80 7.78 7.78 7.57 7.57 7.56 7.55 7.54 7.54 7.53 7.53 7.52 7.52 7.51 7.51 7.50 7.50 7.49 7.34 7.32 7.31 7.29 7.29 7.27 7.27 7.26 7.16 7.16 7.16 7.14 7.14 7.13 7.13 7.13 6.48 3.52
^{1}H NMR (CDCl$_3$, 500 MHz)
3g
1.00
1.01
2.14
4.03
1.07
1.00
3.14
f1 (ppm)

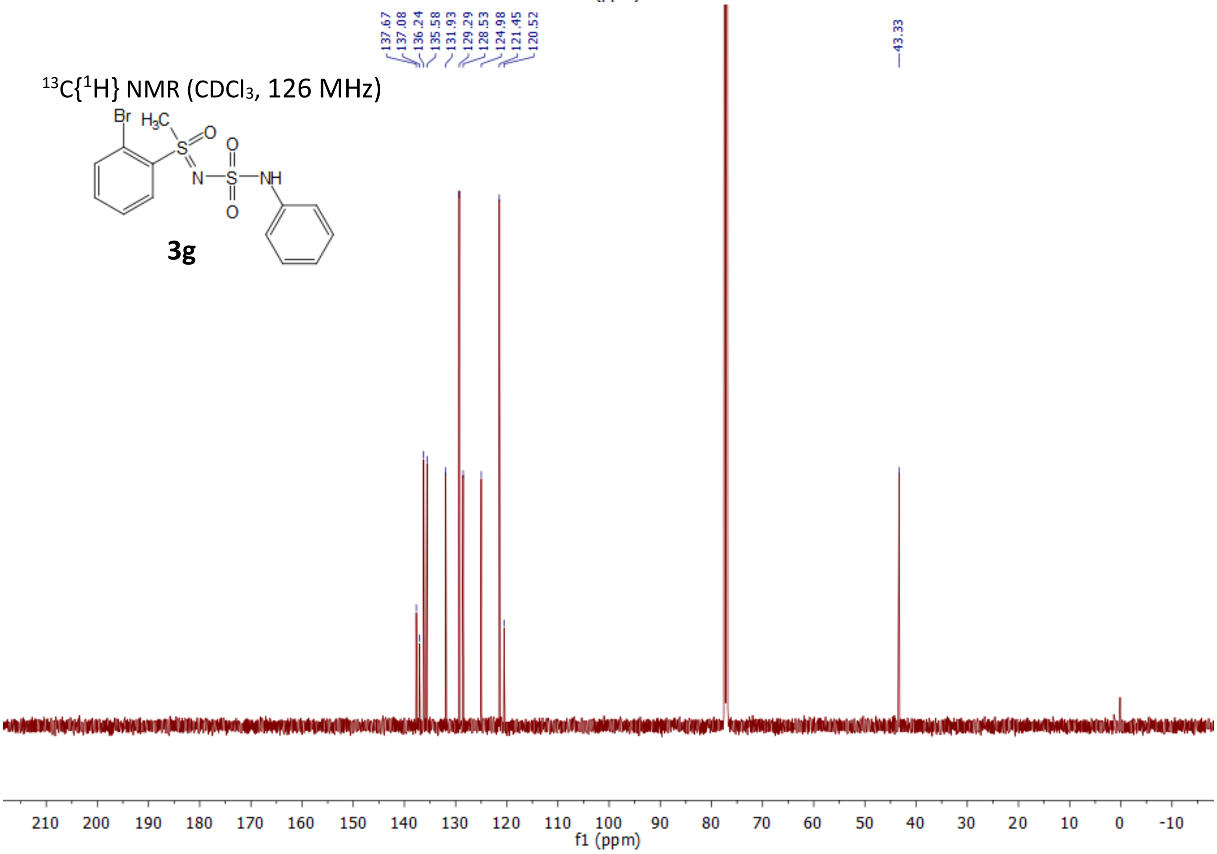

137.67 137.08 136.24 135.58 131.93 129.29 128.53 124.98 121.45 120.52
43.33
^{13}C{^{1}H} NMR (CDCl$_3$, 126 MHz)
3g
f1 (ppm)

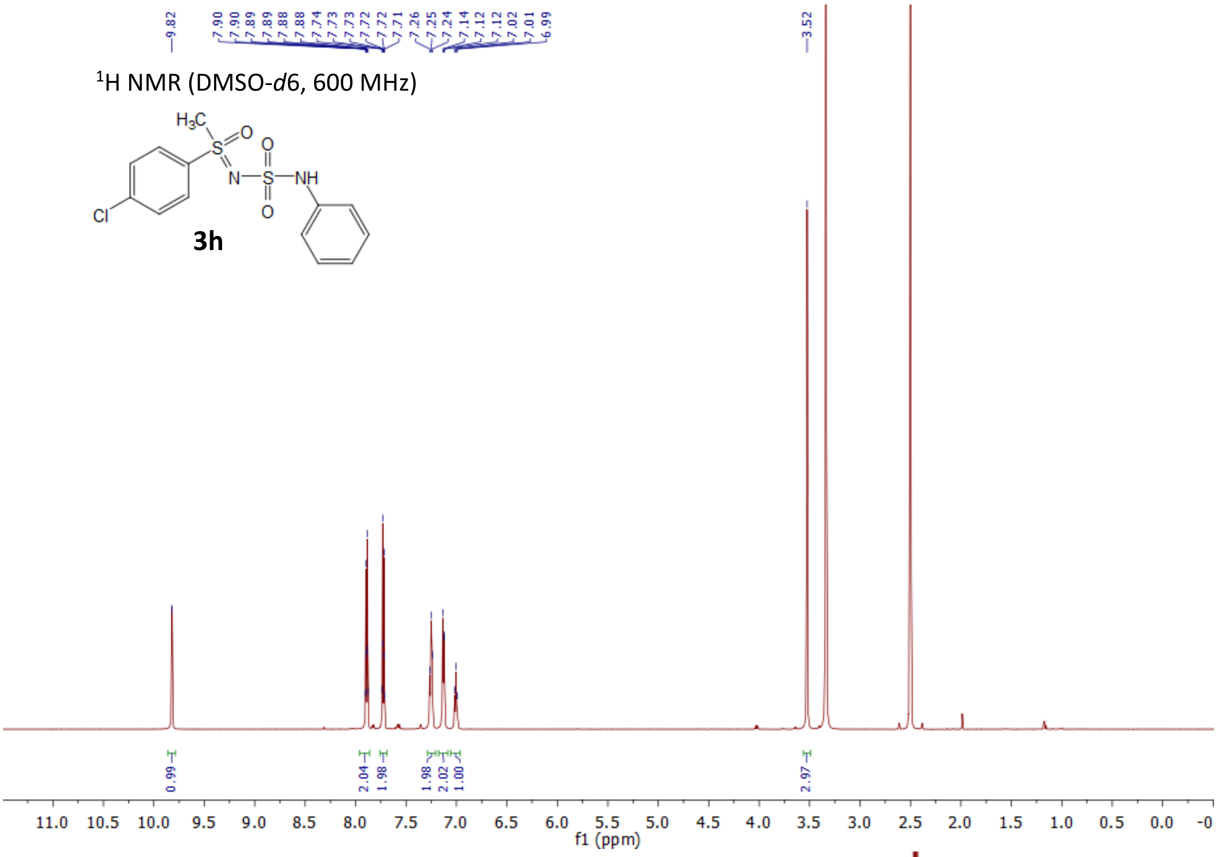
9.82
7.90
7.90
7.89
7.89
7.88
7.88
7.74
7.73
7.73
7.72
7.72
7.71
7.26
7.25
7.24
7.14
7.12
7.12
7.02
7.01
6.99
3.52
¹H NMR (DMSO-d6, 600 MHz)
3h
0.99
2.04
1.98
1.98
2.02
1.00
2.97
f1 (ppm)

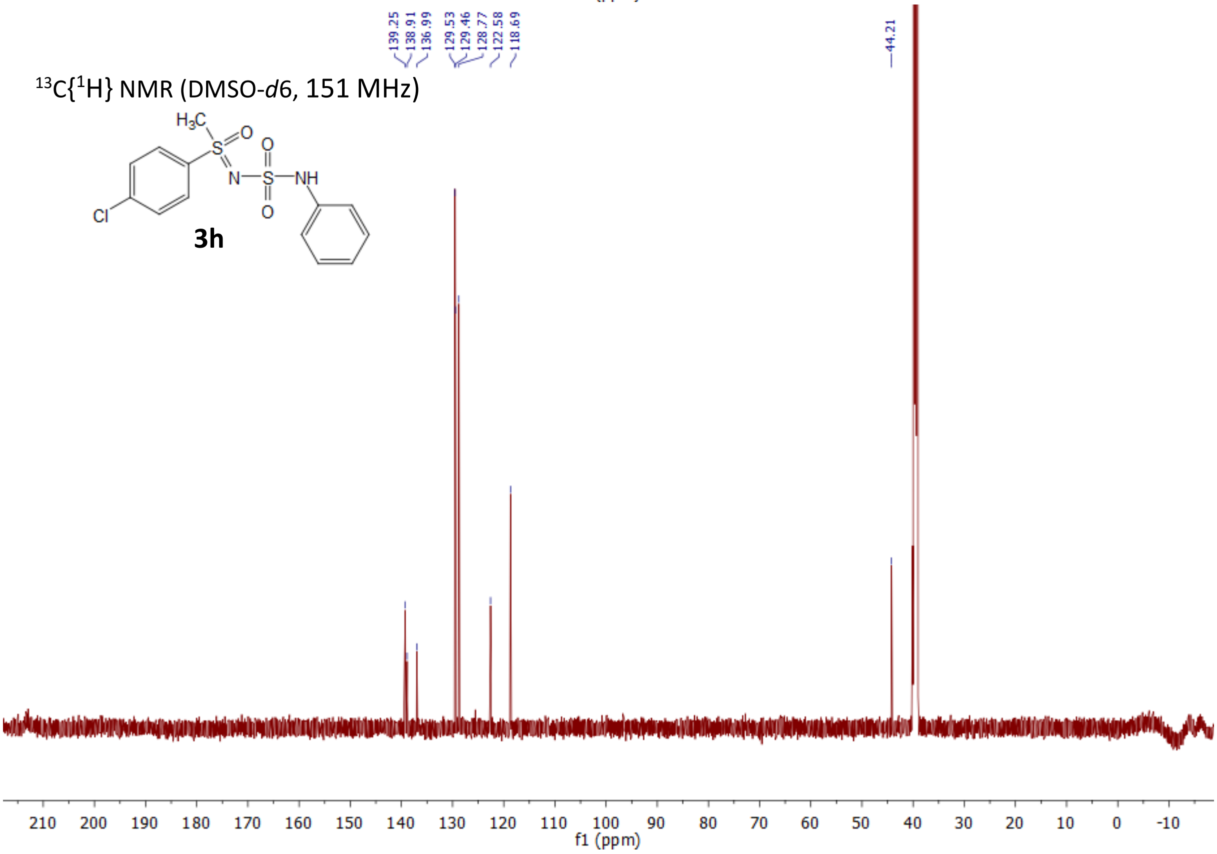
139.25
138.91
136.99
129.53
129.46
128.77
122.58
118.69
44.21
¹³C{¹H} NMR (DMSO-d6, 151 MHz)
3h
f1 (ppm)

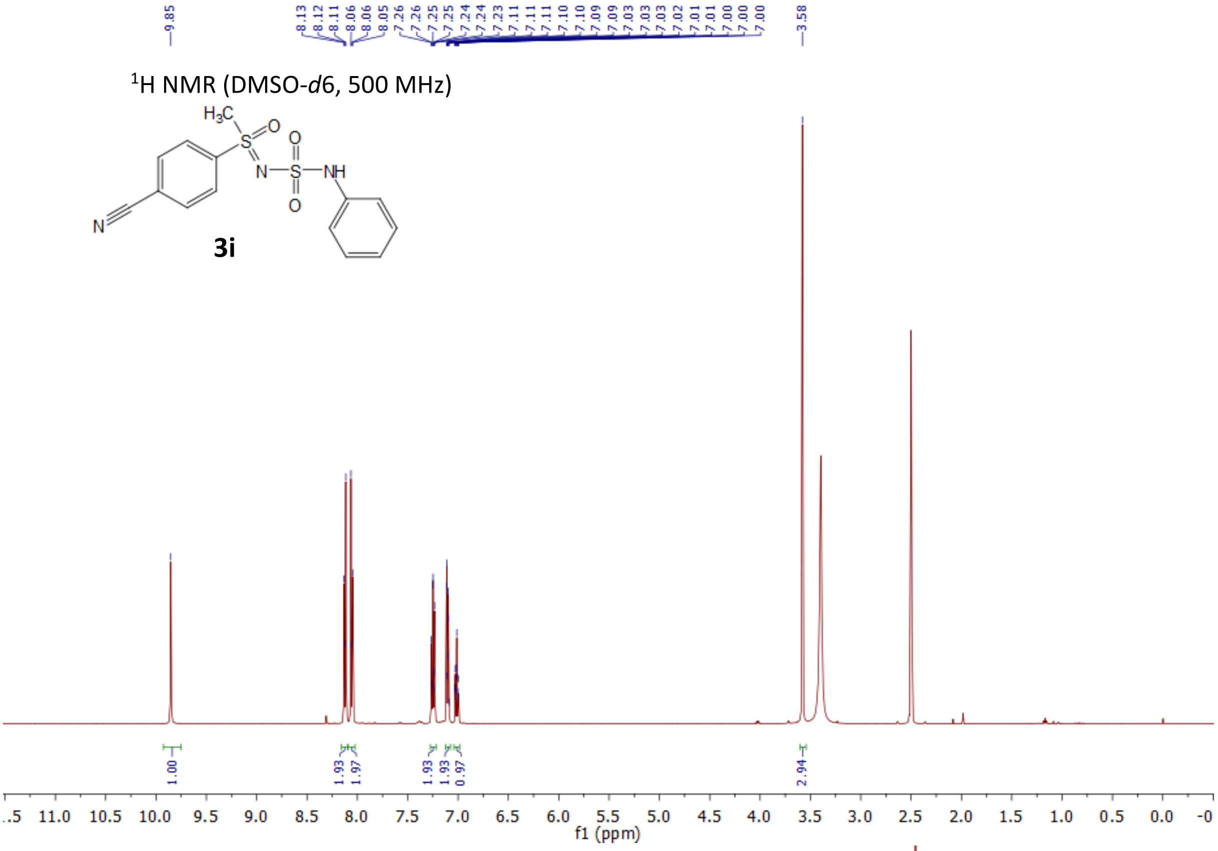

^{1}H NMR (DMSO-$d6$, 500 MHz)
3i
f1 (ppm)

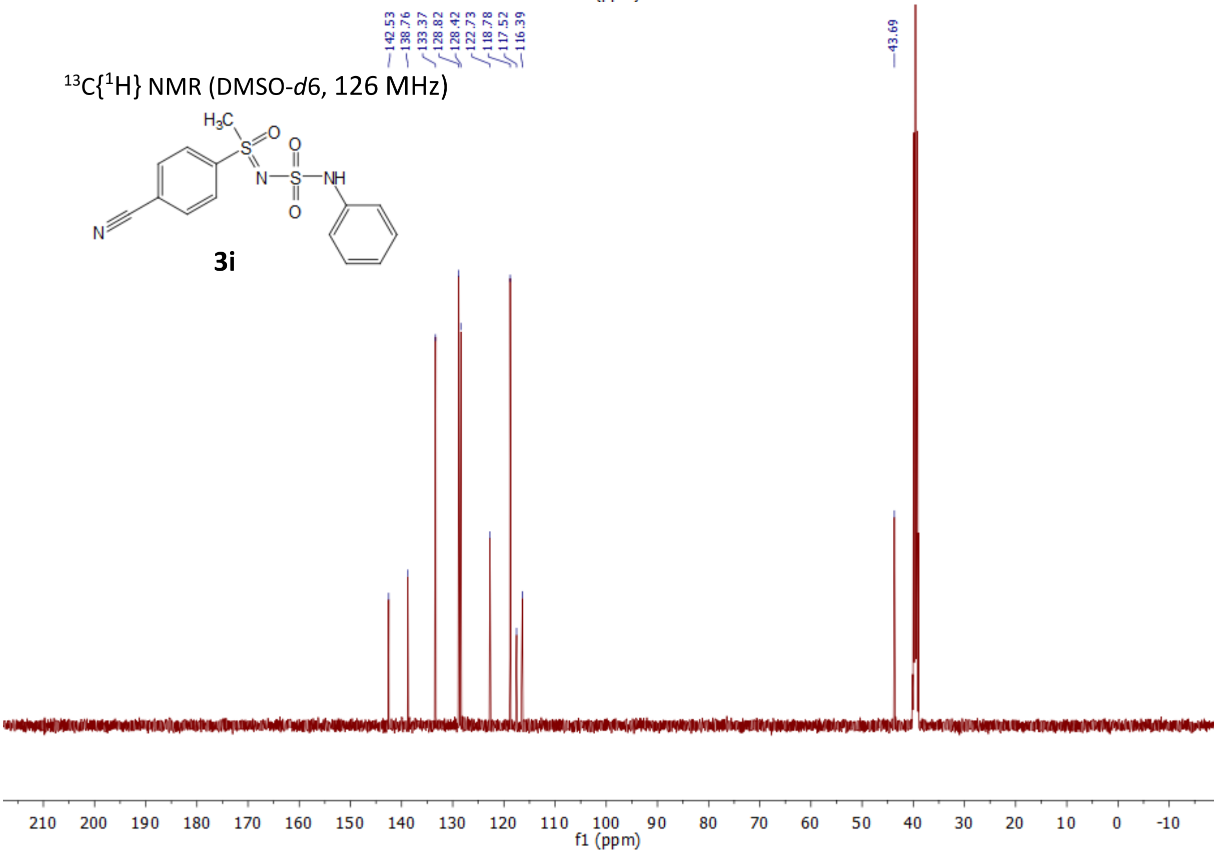

^{13}C{^{1}H} NMR (DMSO-$d6$, 126 MHz)
3i
f1 (ppm)

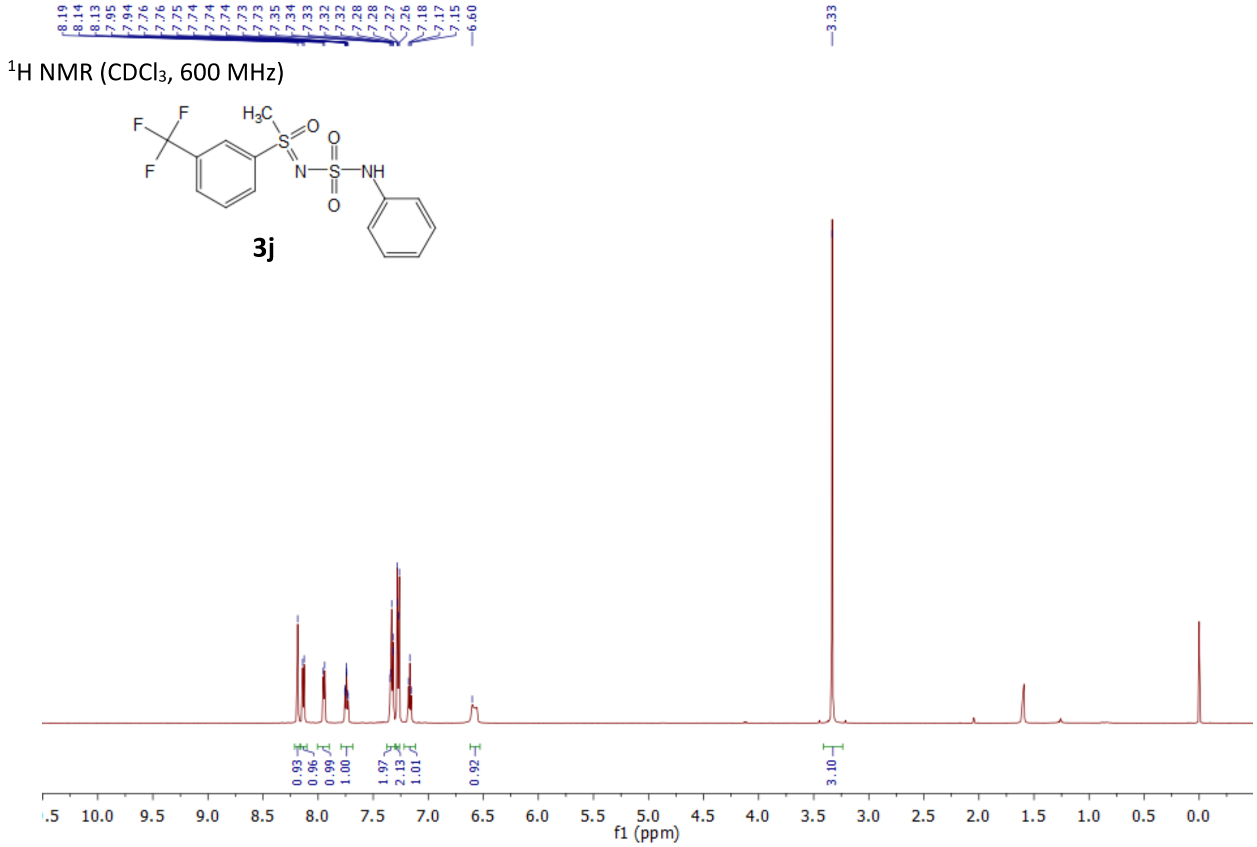
8.19
8.14
8.13
7.95
7.94
7.76
7.76
7.75
7.74
7.74
7.74
7.73
7.73
7.35
7.34
7.33
7.32
7.32
7.28
7.28
7.27
7.26
7.18
7.17
7.15
6.60
3.33
^{1}H NMR ($CDCl_3$, 600 MHz)
3j
0.93
0.96
0.99
1.00
1.97
2.13
1.01
0.92
3.10
f1 (ppm)

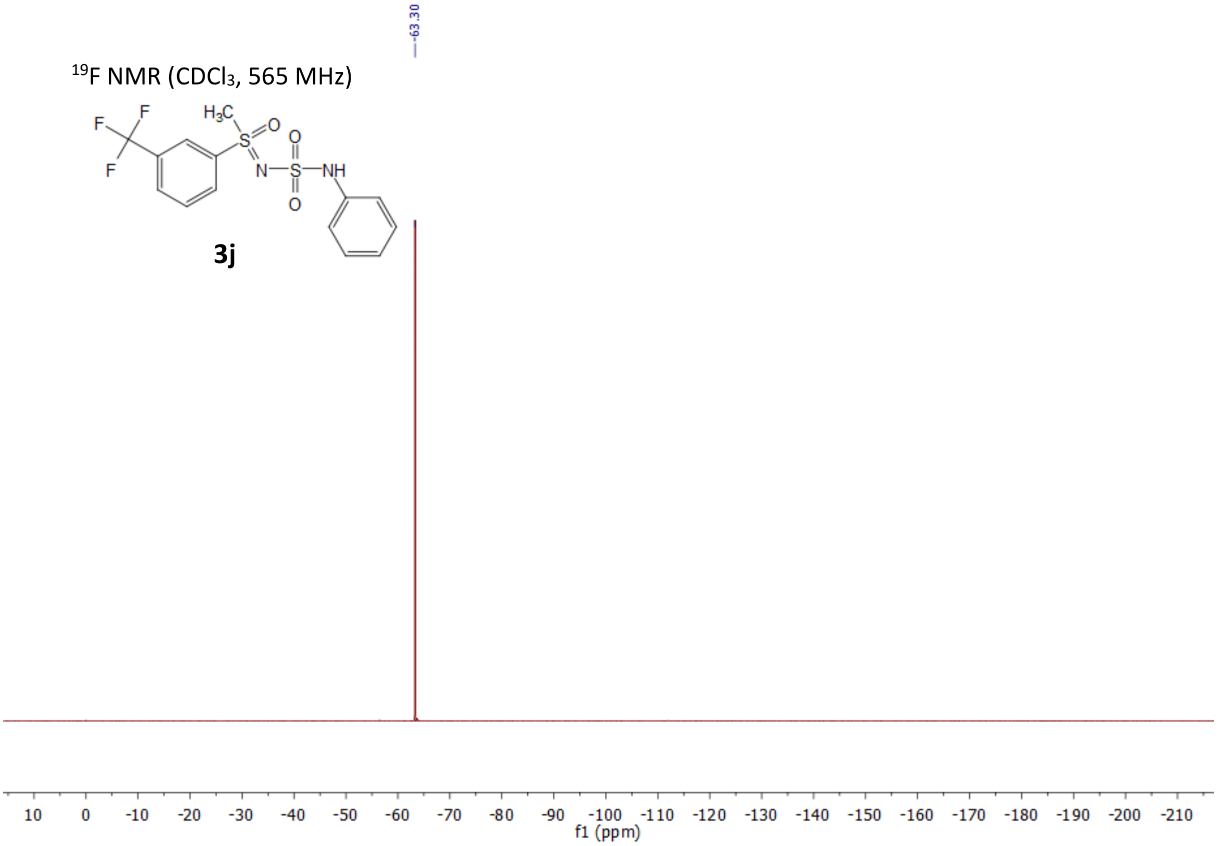
-63.30
^{19}F NMR ($CDCl_3$, 565 MHz)
3j
f1 (ppm)

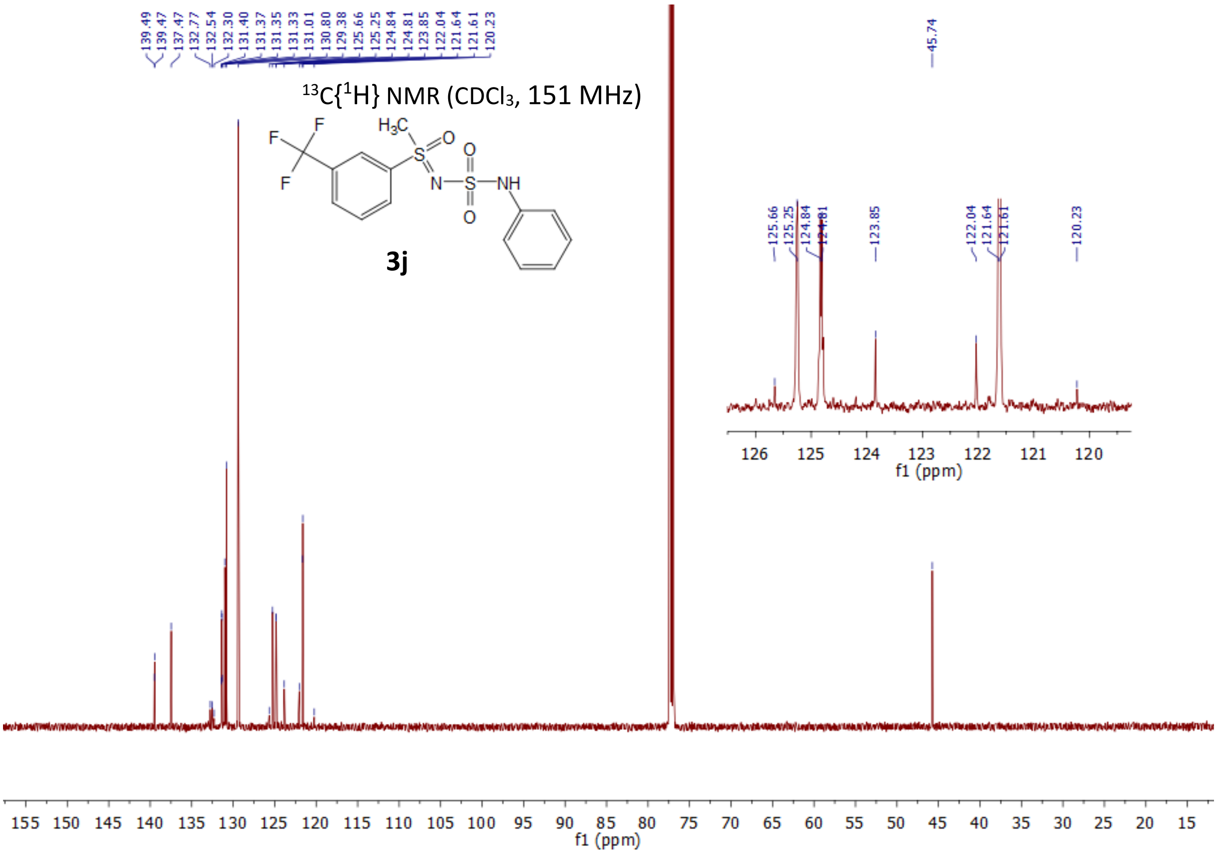

139.49
139.47
137.47
132.77
132.54
132.30
131.40
131.37
131.35
131.33
131.01
130.80
129.38
125.66
125.25
124.84
124.81
123.85
122.04
121.64
121.61
120.23
45.74
$^{13}C\{^1H\}$ NMR ($CDCl_3$, 151 MHz)
F
F
F
H_3C
S
O
N
O
S
NH
O
3j
f1 (ppm)

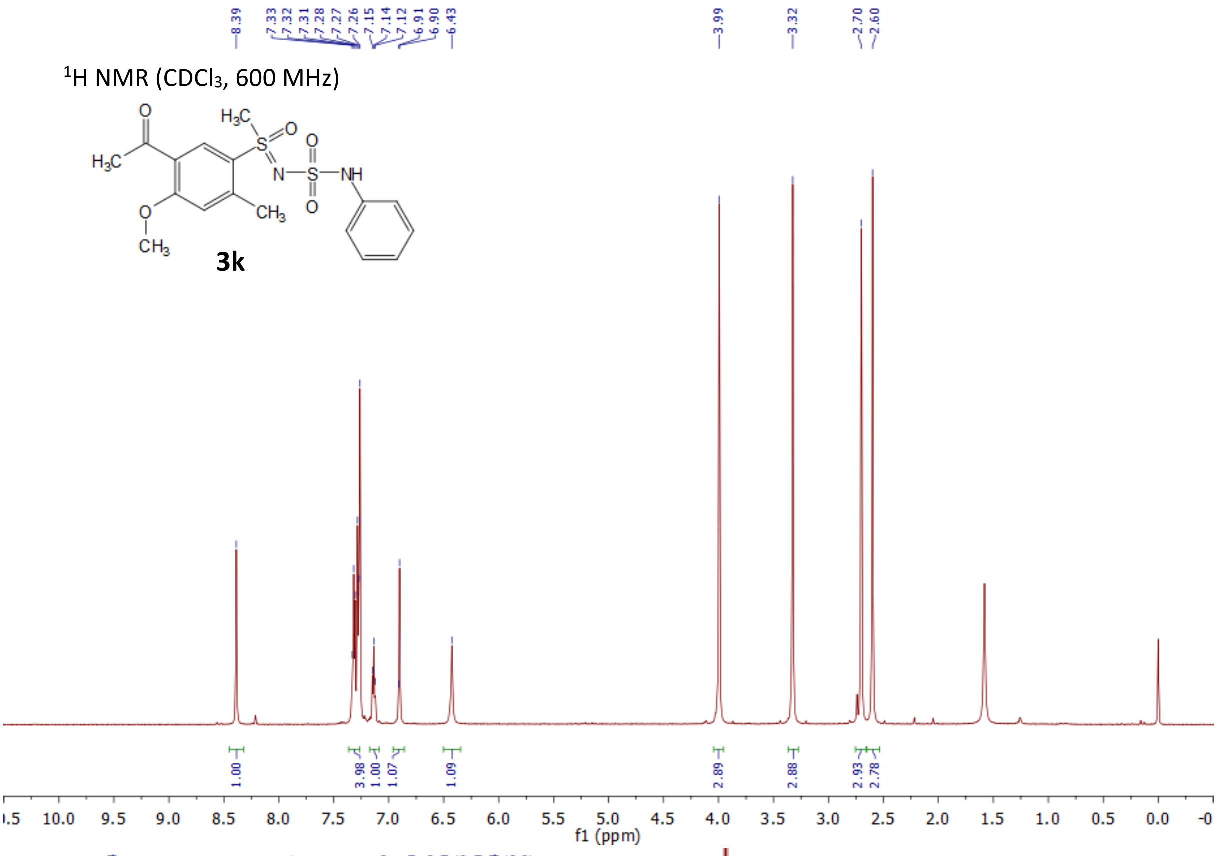

8.39
7.33 7.32 7.31 7.28 7.27 7.26 7.15 7.14 7.12 6.91 6.90 6.43
3.99
3.32
2.70 2.60
^{1}H NMR (CDCl$_3$, 600 MHz)
3k
1.00 3.98 1.00 1.07 1.09 2.89 2.88 2.93 2.78
f1 (ppm)

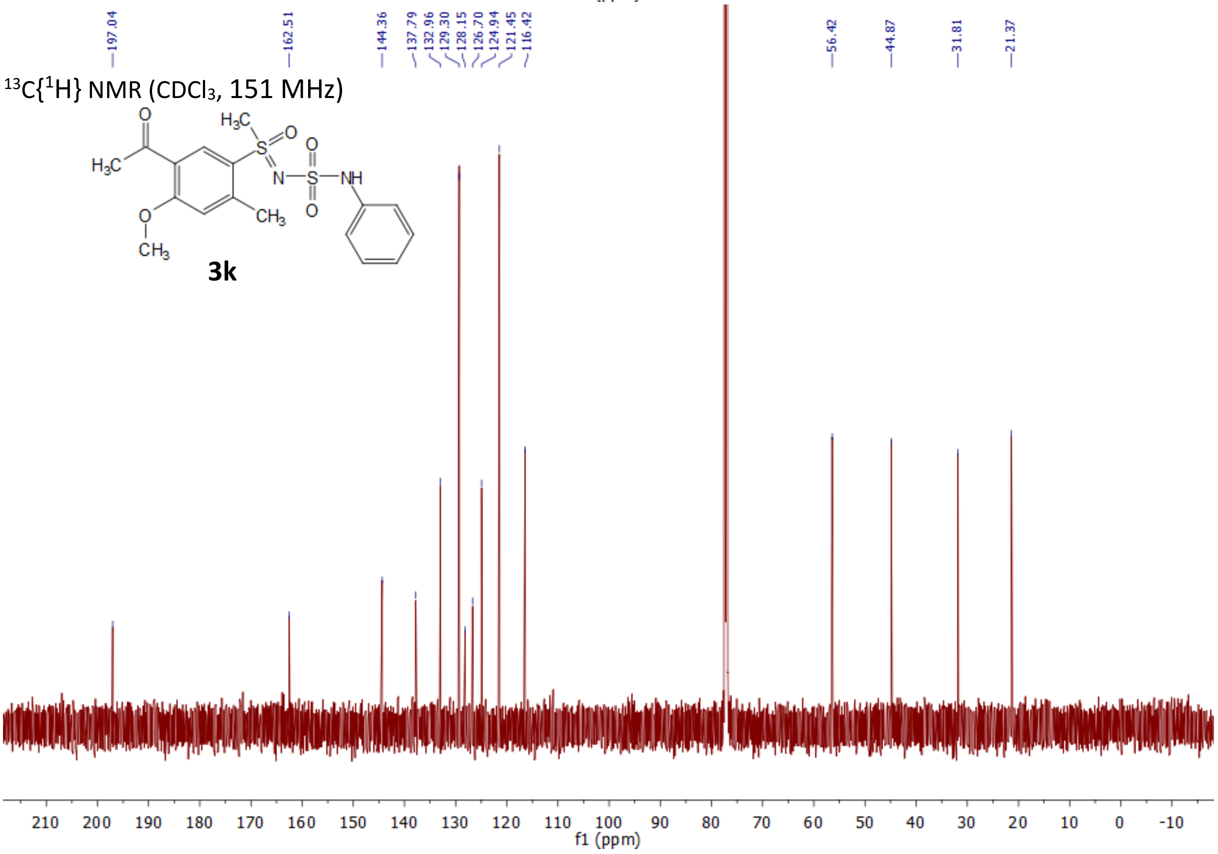

197.04
162.51
144.36 137.79 132.96 129.30 128.15 126.70 124.94 121.45 116.42
56.42
44.87
31.81
21.37
^{13}C{^{1}H} NMR (CDCl$_3$, 151 MHz)
3k
f1 (ppm)

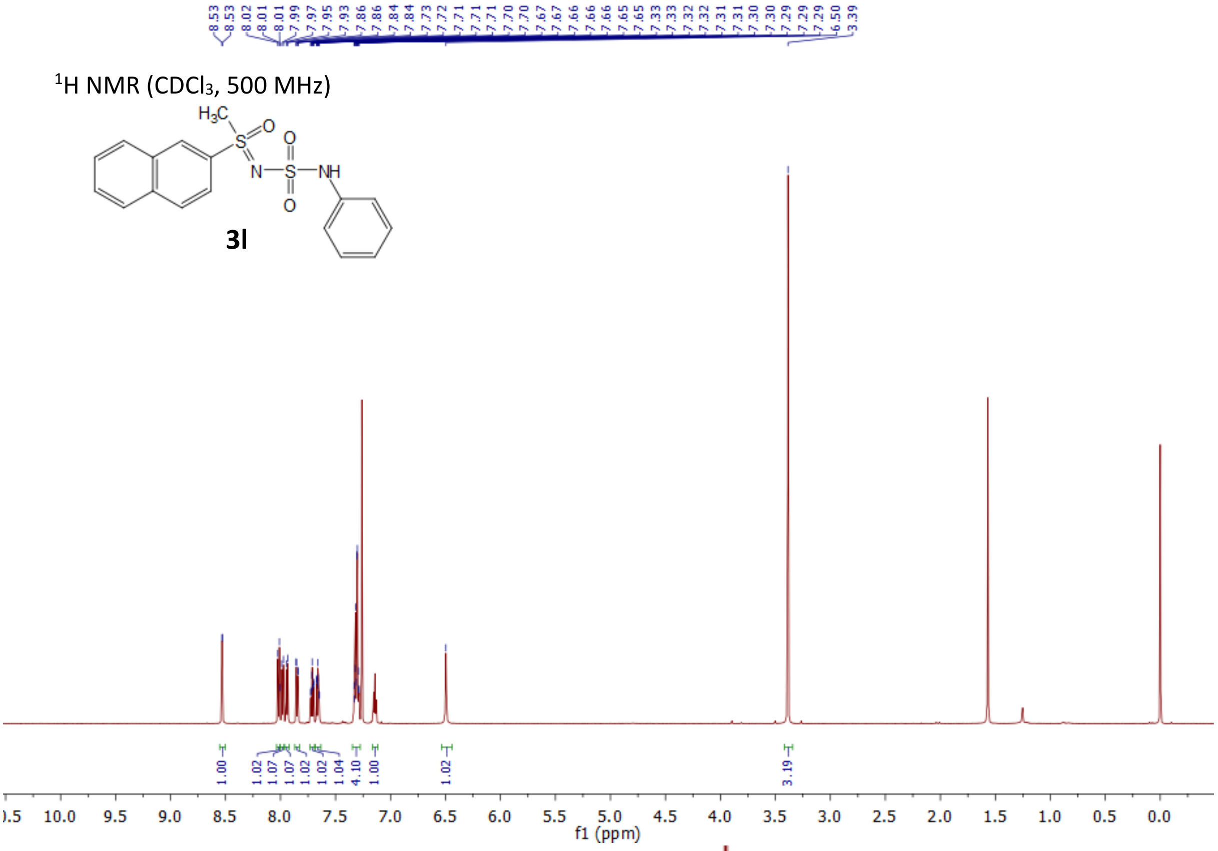

8.53 8.53 8.02 8.01 7.99 7.97 7.95 7.93 7.86 7.86 7.84 7.84 7.73 7.72 7.71 7.71 7.71 7.70 7.70 7.67 7.67 7.66 7.66 7.65 7.65 7.33 7.33 7.32 7.32 7.31 7.31 7.30 7.30 7.29 7.29 7.29 6.50 3.39
^{1}H NMR (CDCl$_3$, 500 MHz)
H_3C
S
O
N
O
S
NH
O
3l
1.00 1.02 1.07 1.07 1.02 1.02 1.04 4.10 1.00 1.02 3.19
f1 (ppm)

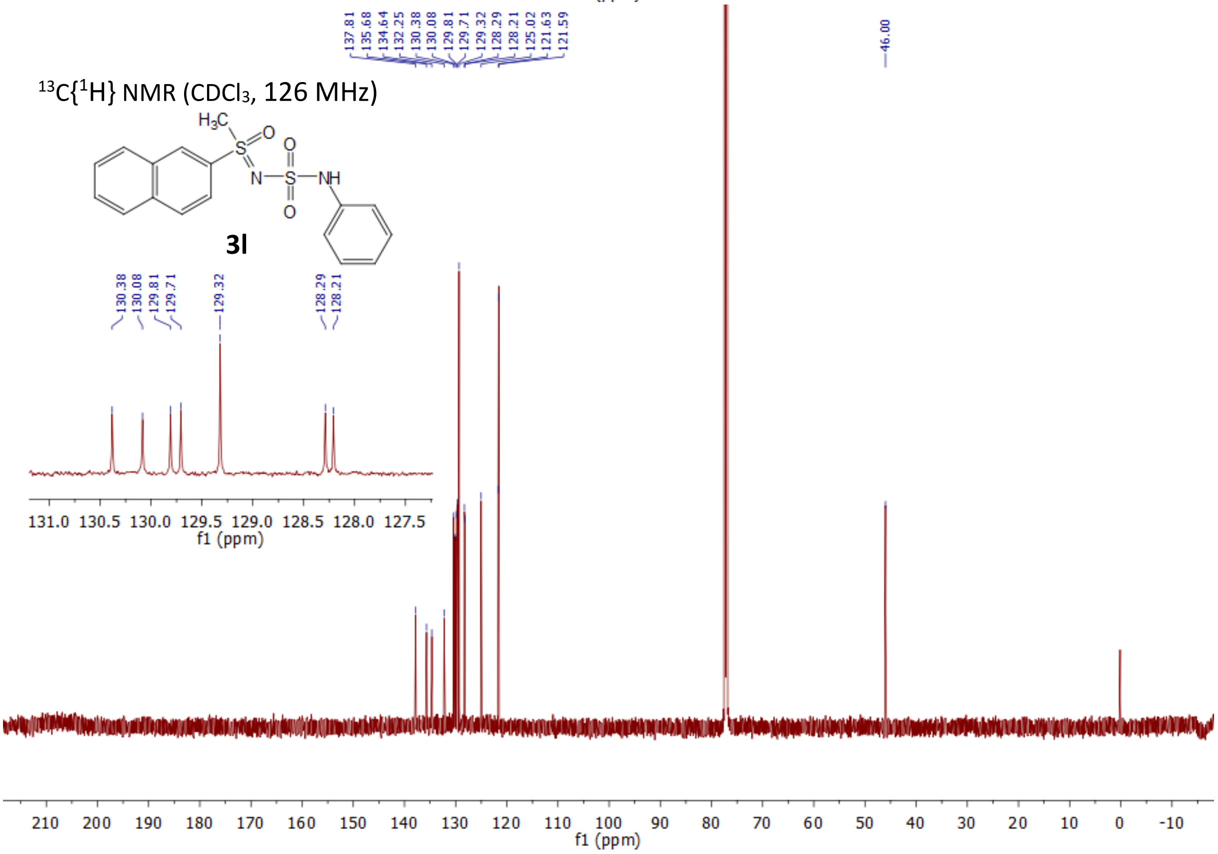

137.81 135.68 134.64 132.25 130.38 130.08 129.81 129.71 129.32 128.29 128.21 125.02 121.63 121.59
46.00
^{13}C{^{1}H} NMR (CDCl$_3$, 126 MHz)
H_3C
S
O
N
O
S
NH
O
3l
130.38 130.08 129.81 129.71 129.32 128.29 128.21
131.0 130.5 130.0 129.5 129.0 128.5 128.0 127.5
f1 (ppm)
f1 (ppm)

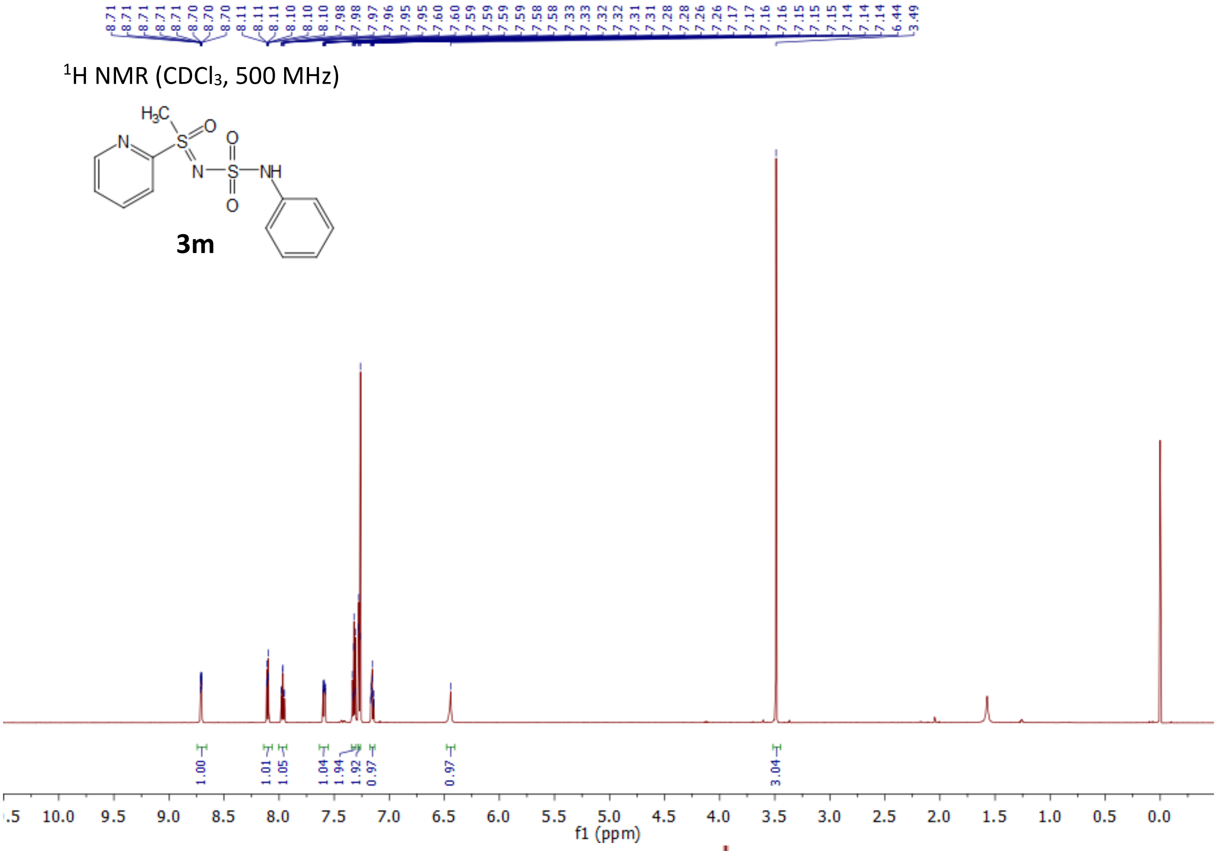
^1H NMR ($CDCl_3$, 500 MHz)
3m
f1 (ppm)

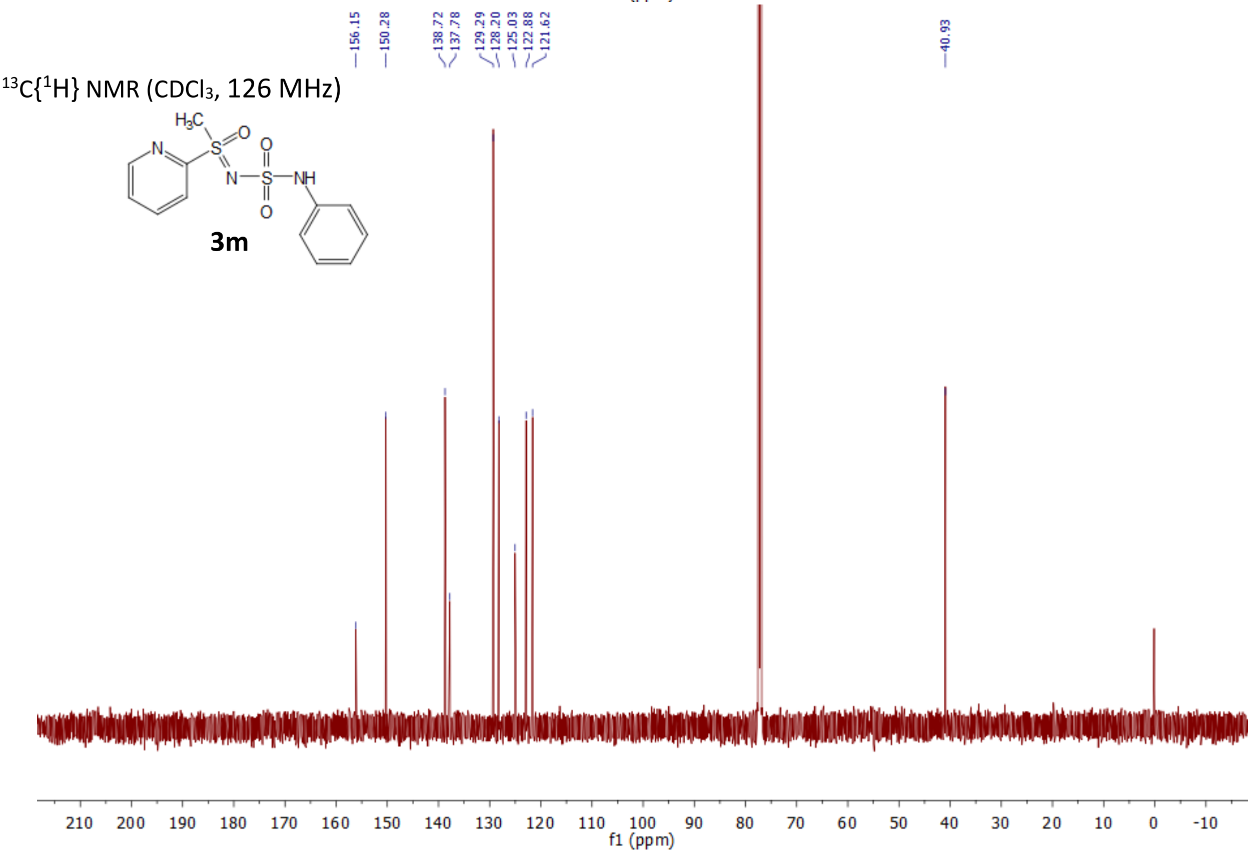
$^{13}C\{^1H\}$ NMR ($CDCl_3$, 126 MHz)
3m
156.15
150.28
138.72
137.78
129.29
128.20
125.03
122.88
121.62
40.93
f1 (ppm)

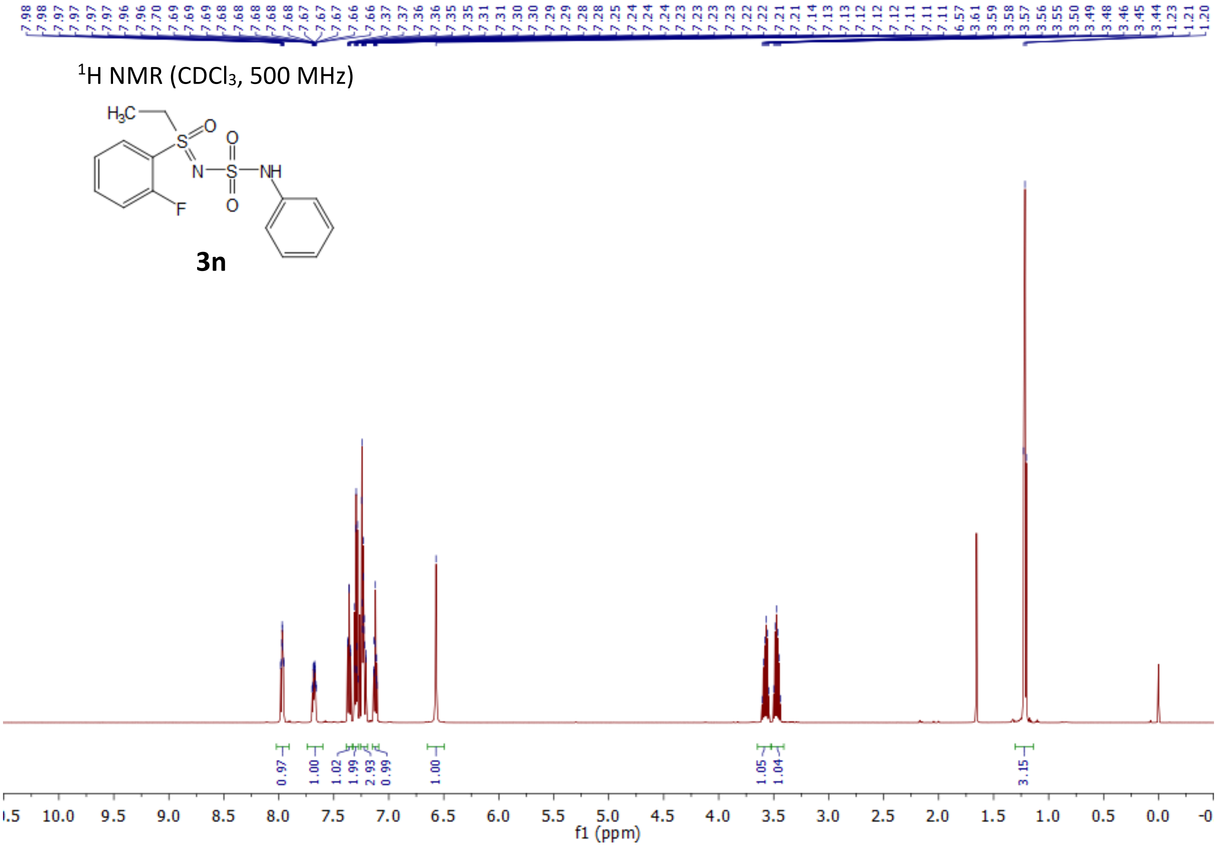

^{1}H NMR ($CDCl_3$, 500 MHz)
3n
f1 (ppm)

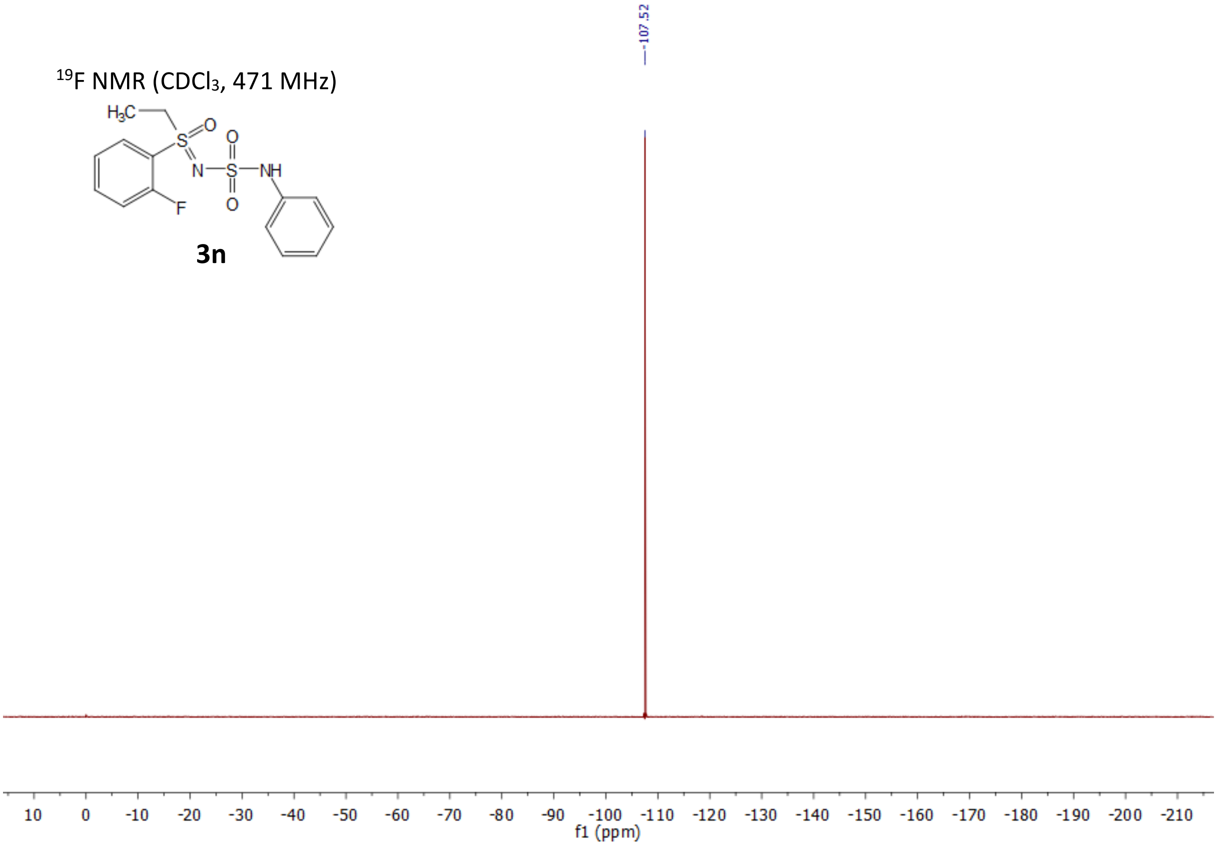

-107.52
^{19}F NMR ($CDCl_3$, 471 MHz)
3n
f1 (ppm)

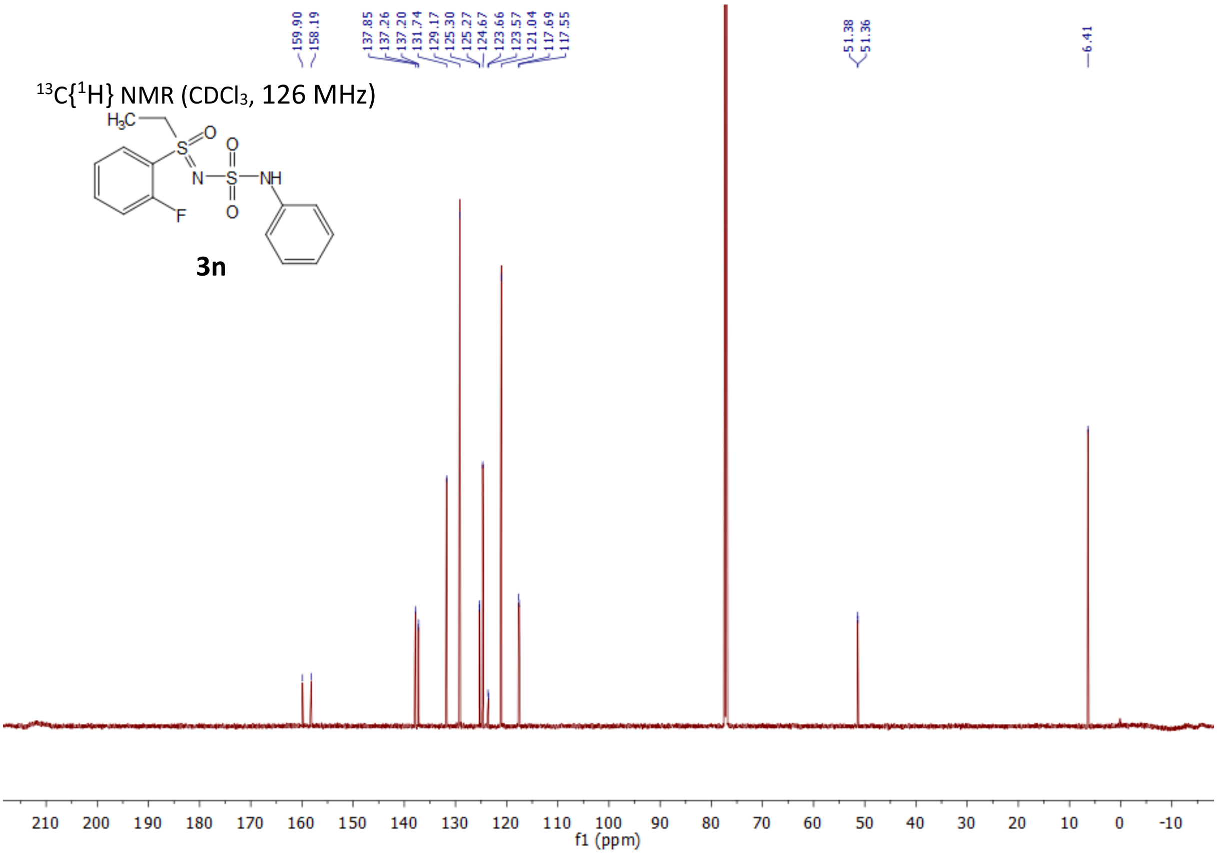

$^{13}C\{^1H\}$ NMR ($CDCl_3$, 126 MHz)
3n
159.90
158.19
137.85
137.26
137.20
131.74
129.17
125.30
125.27
124.67
123.66
123.57
121.04
117.69
117.55
51.38
51.36
6.41
210 200 190 180 170 160 150 140 130 120 110 100 90 80 70 60 50 40 30 20 10 0 -10
f1 (ppm)

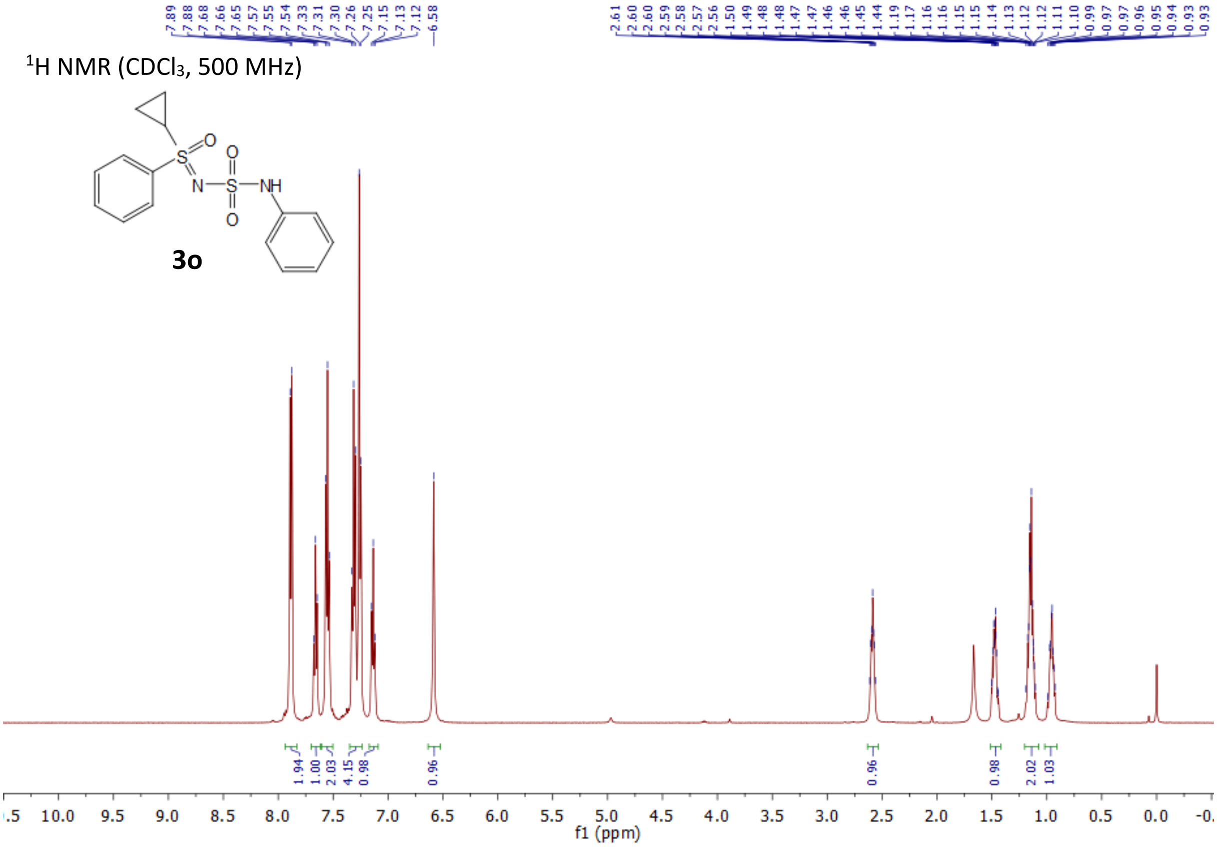

^{1}H NMR ($CDCl_3$, 500 MHz)
3o
f1 (ppm)

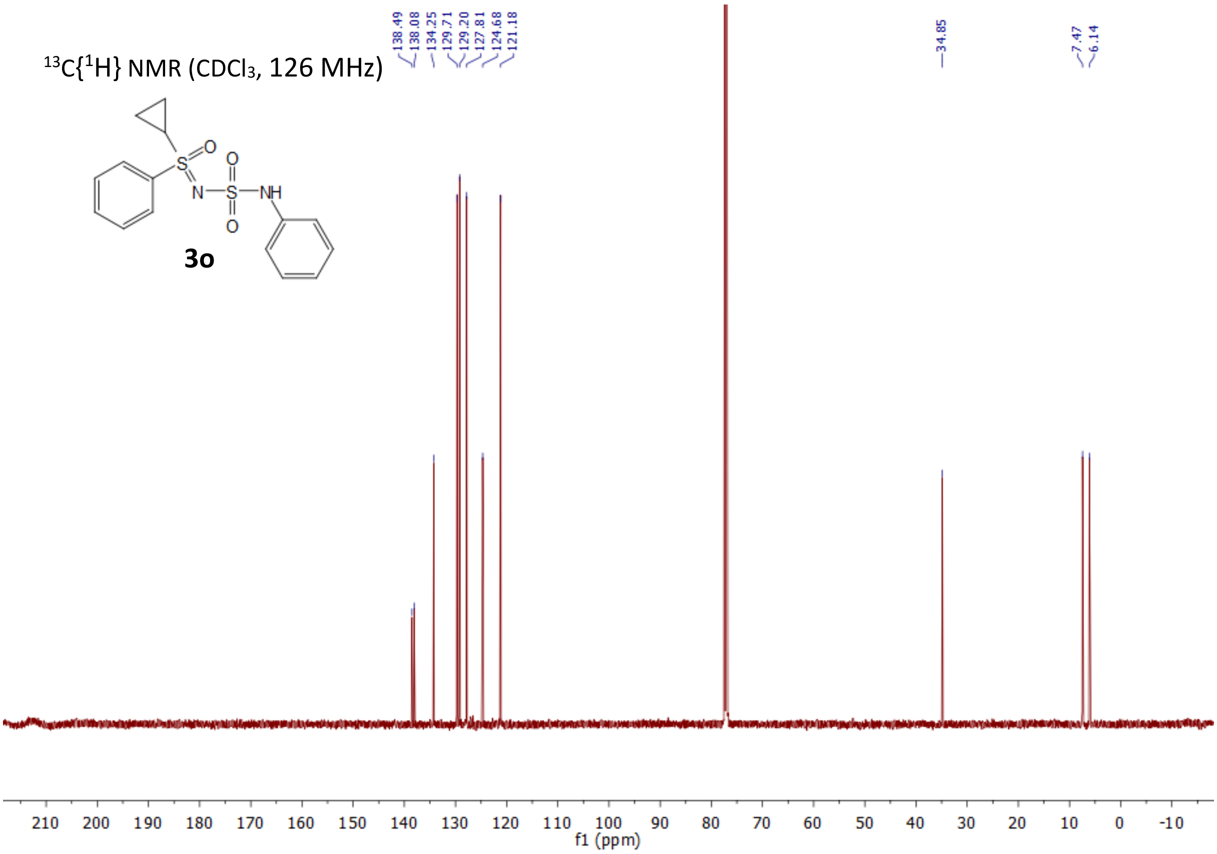

$^{13}C\{^1H\}$ NMR ($CDCl_3$, 126 MHz)
3o
f1 (ppm)

^{1}H NMR ($CDCl_3$, 600 MHz)

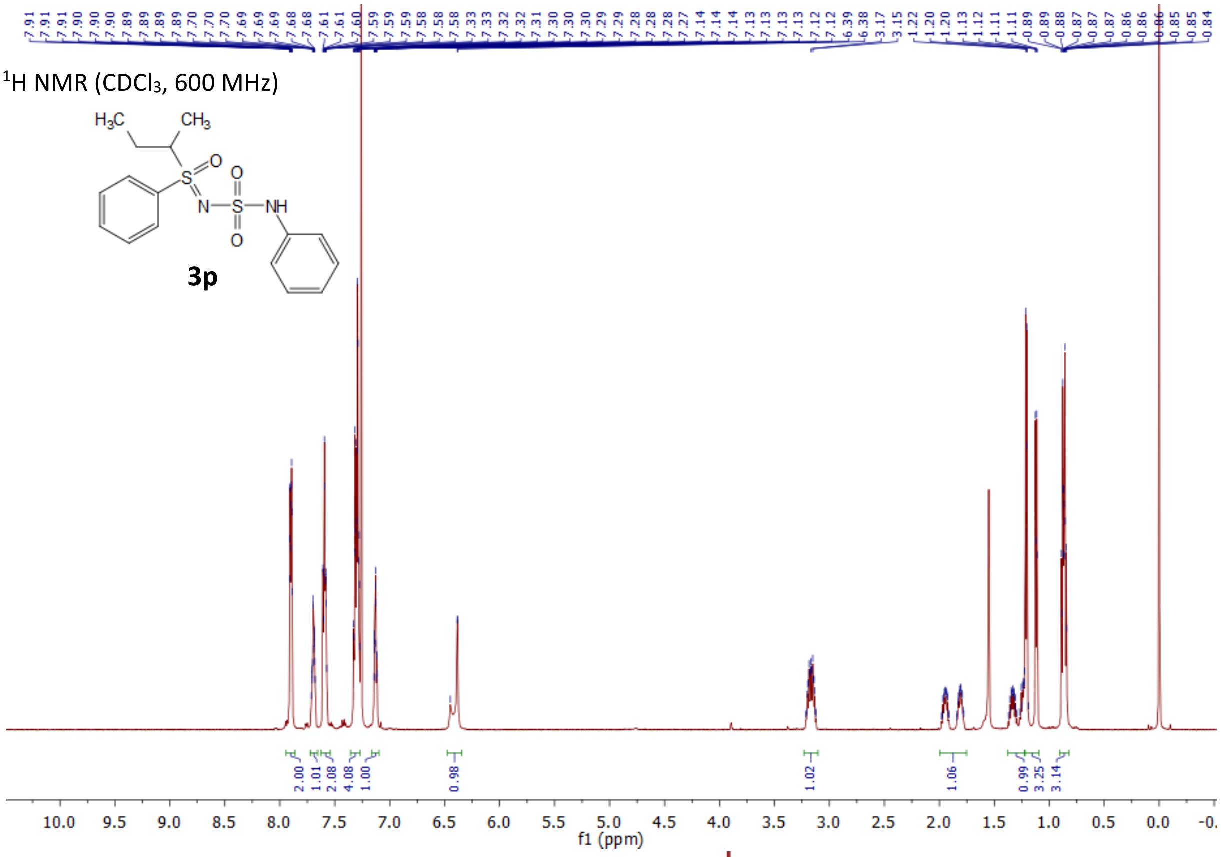

$^{13}C\{^{1}H\}$ NMR ($CDCl_3$, 151 MHz)

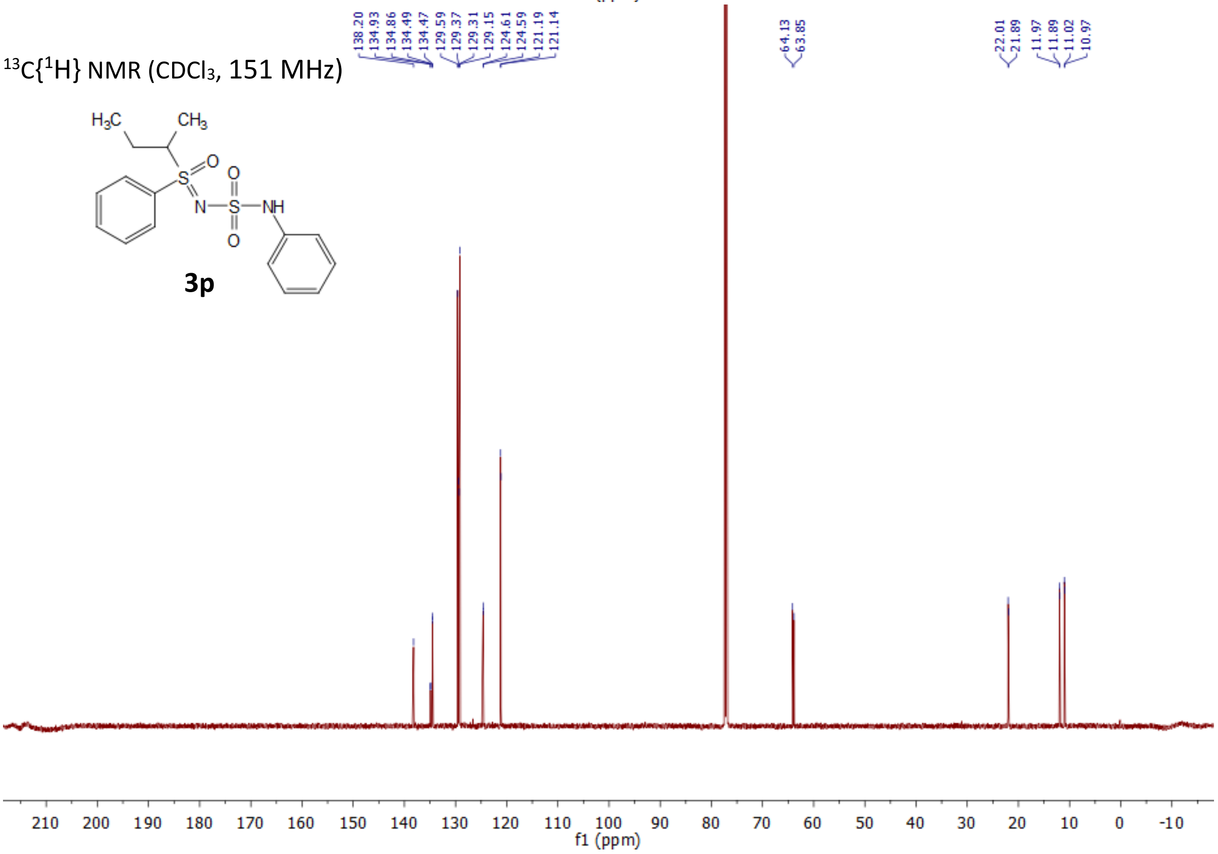

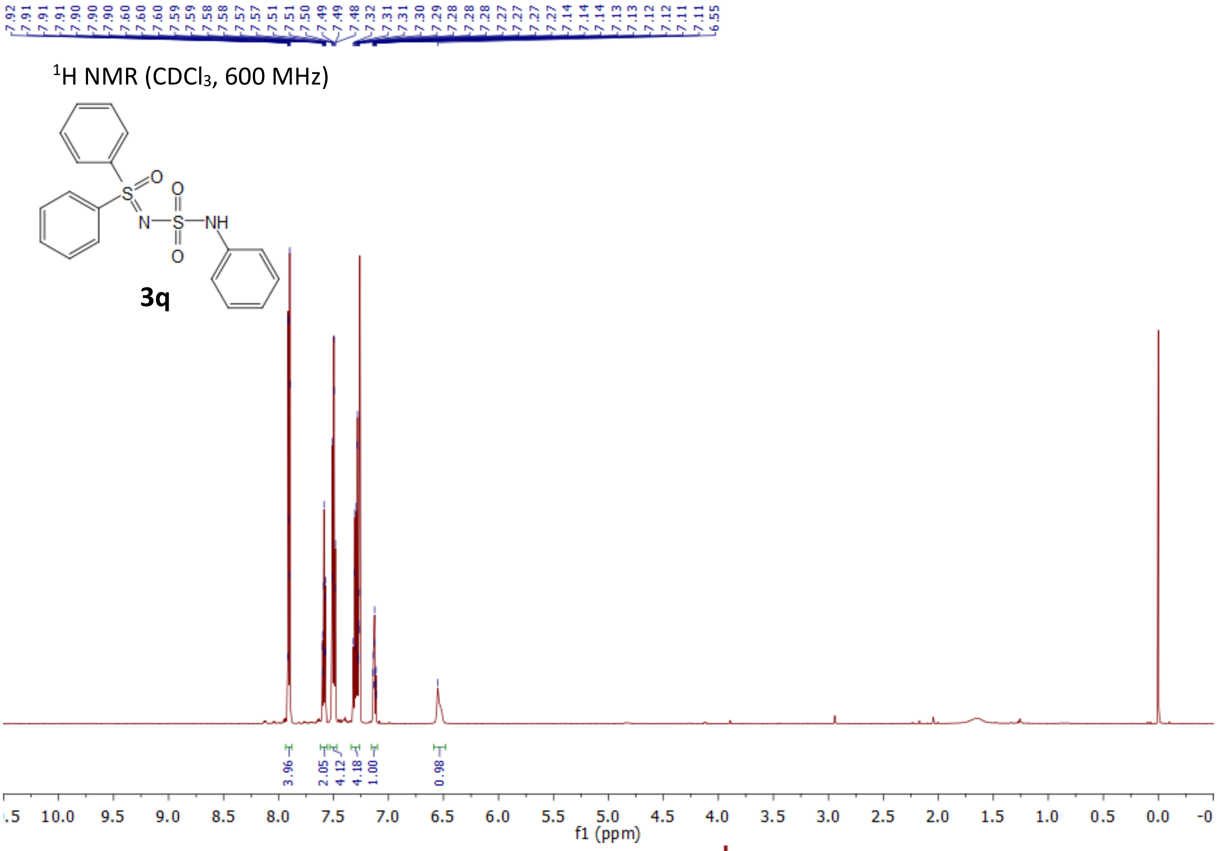
7.92 7.91 7.91 7.90 7.90 7.90 7.60 7.60 7.60 7.59 7.59 7.58 7.58 7.57 7.57 7.51 7.51 7.50 7.49 7.49 7.48 7.32 7.31 7.31 7.30 7.29 7.28 7.28 7.28 7.27 7.27 7.27 7.14 7.14 7.13 7.13 7.12 7.12 7.11 7.11 6.55
1H NMR (CDCl3, 600 MHz)
3q
3.96
2.05
4.12
4.18
1.00
0.98
f1 (ppm)

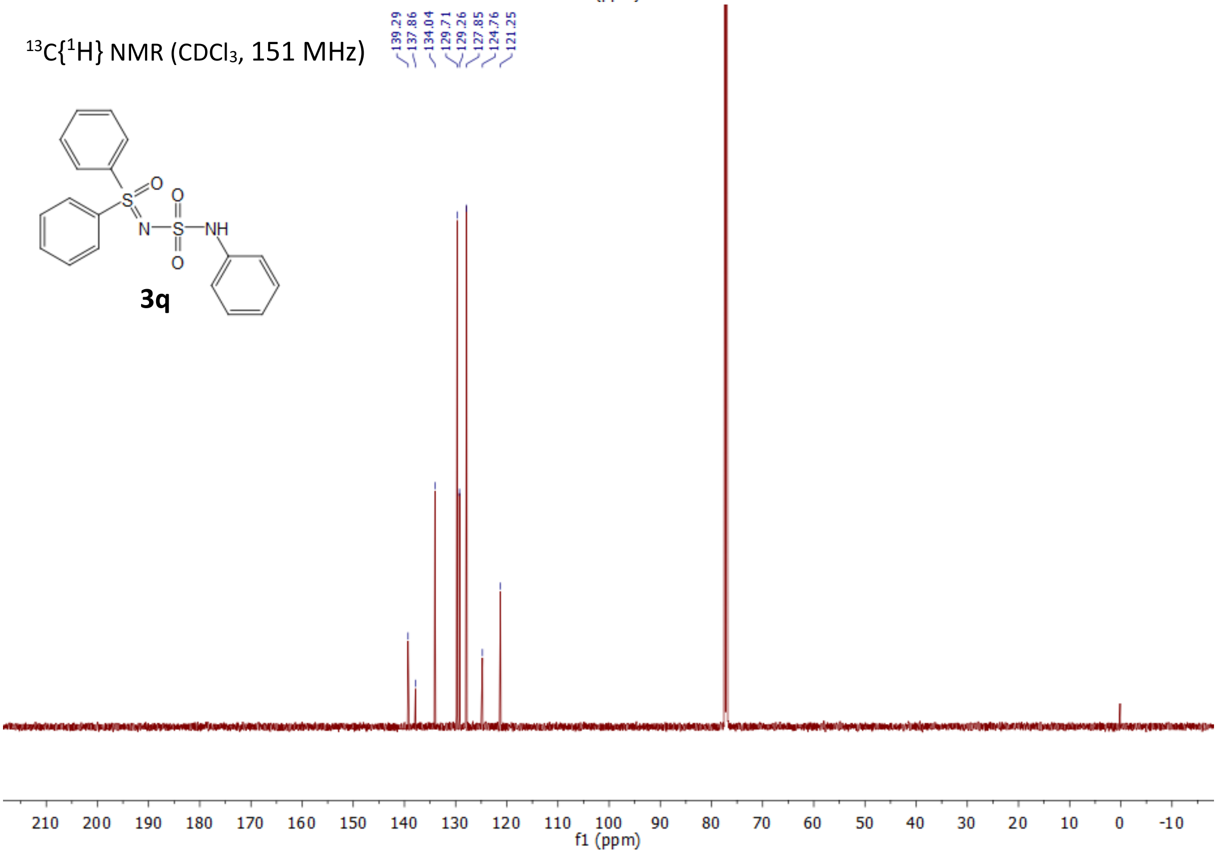
139.29 137.86 134.04 129.71 129.26 127.85 124.76 121.25
13C{1H} NMR (CDCl3, 151 MHz)
3q
f1 (ppm)

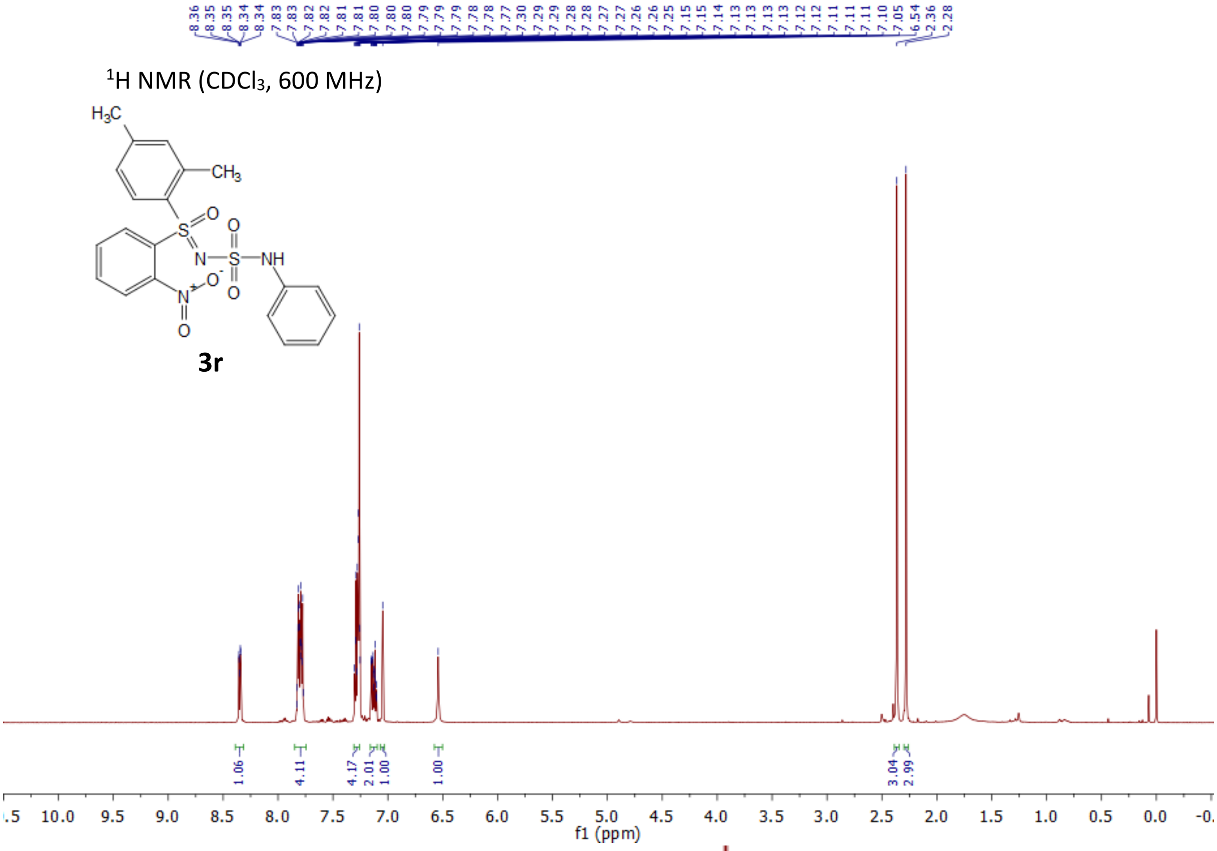

8.36 8.35 8.35 8.34 8.34 7.83 7.83 7.82 7.82 7.81 7.81 7.80 7.80 7.80 7.79 7.79 7.79 7.78 7.78 7.78 7.77 7.30 7.29 7.29 7.28 7.28 7.27 7.27 7.26 7.26 7.25 7.15 7.15 7.14 7.13 7.13 7.13 7.13 7.12 7.12 7.11 7.11 7.11 7.10 7.05 6.54 2.36 2.28
^{1}H NMR (CDCl$_3$, 600 MHz)
3r
1.06 4.11 4.17 2.01 1.00 1.00 3.04 2.99
f1 (ppm)

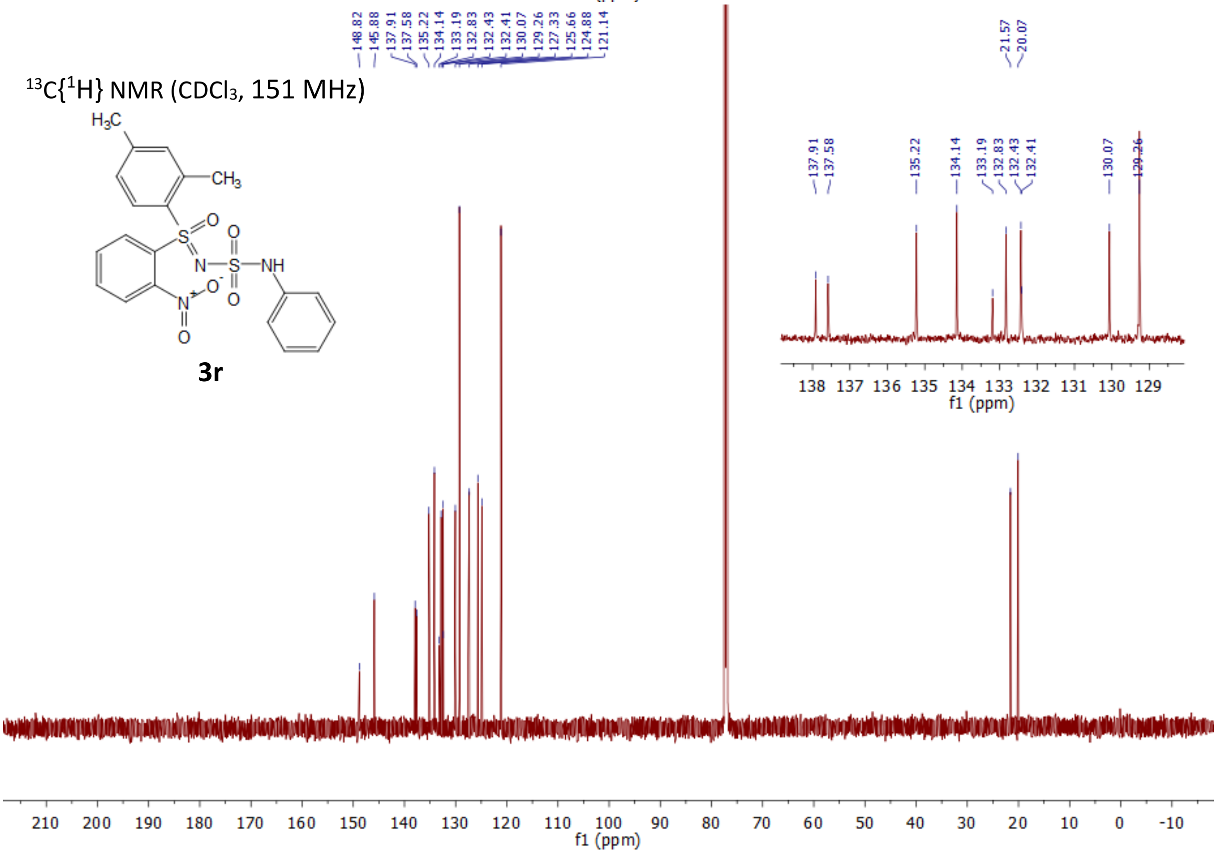

148.82 145.88 137.91 137.58 135.22 134.14 133.19 132.83 132.43 132.41 130.07 129.26 127.33 125.66 124.88 121.14
21.57 20.07
^{13}C{^{1}H} NMR (CDCl$_3$, 151 MHz)
3r
f1 (ppm)

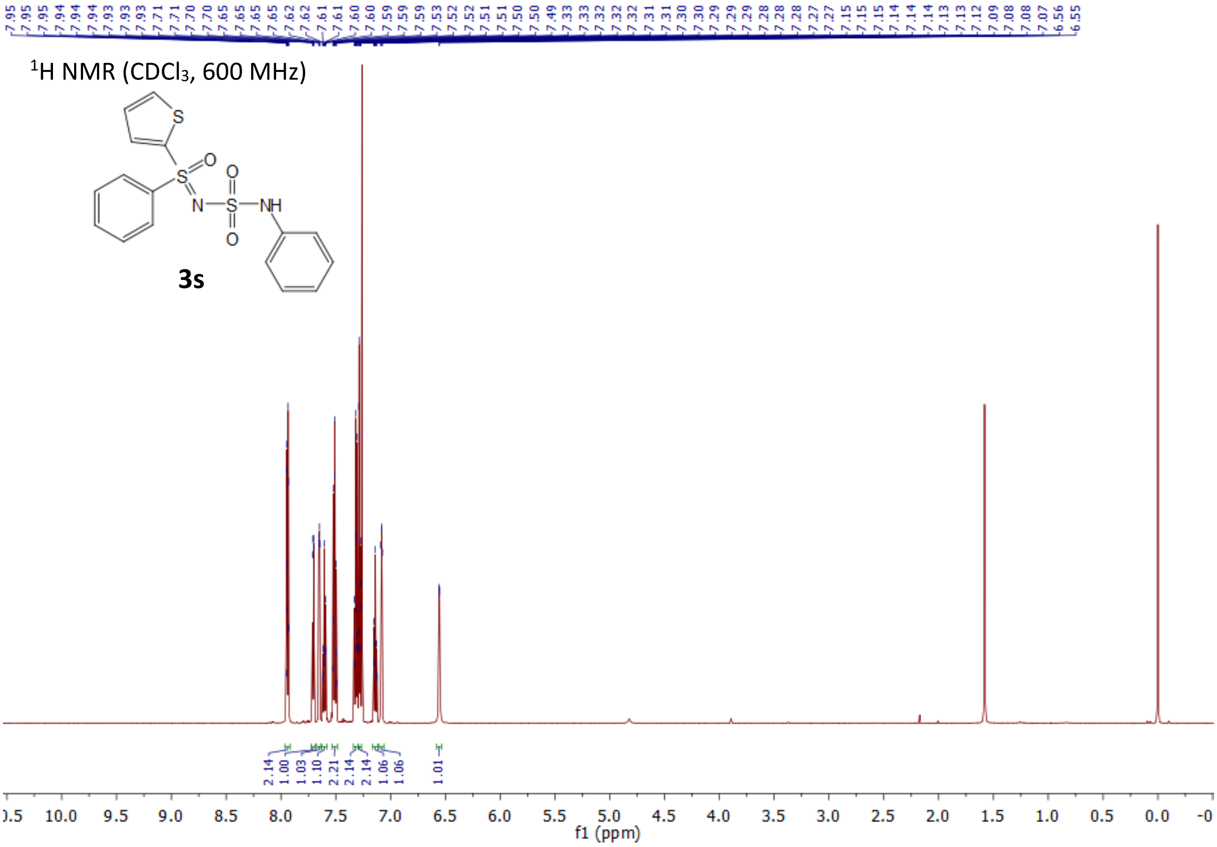
1H NMR (CDCl3, 600 MHz)
3s
f1 (ppm)

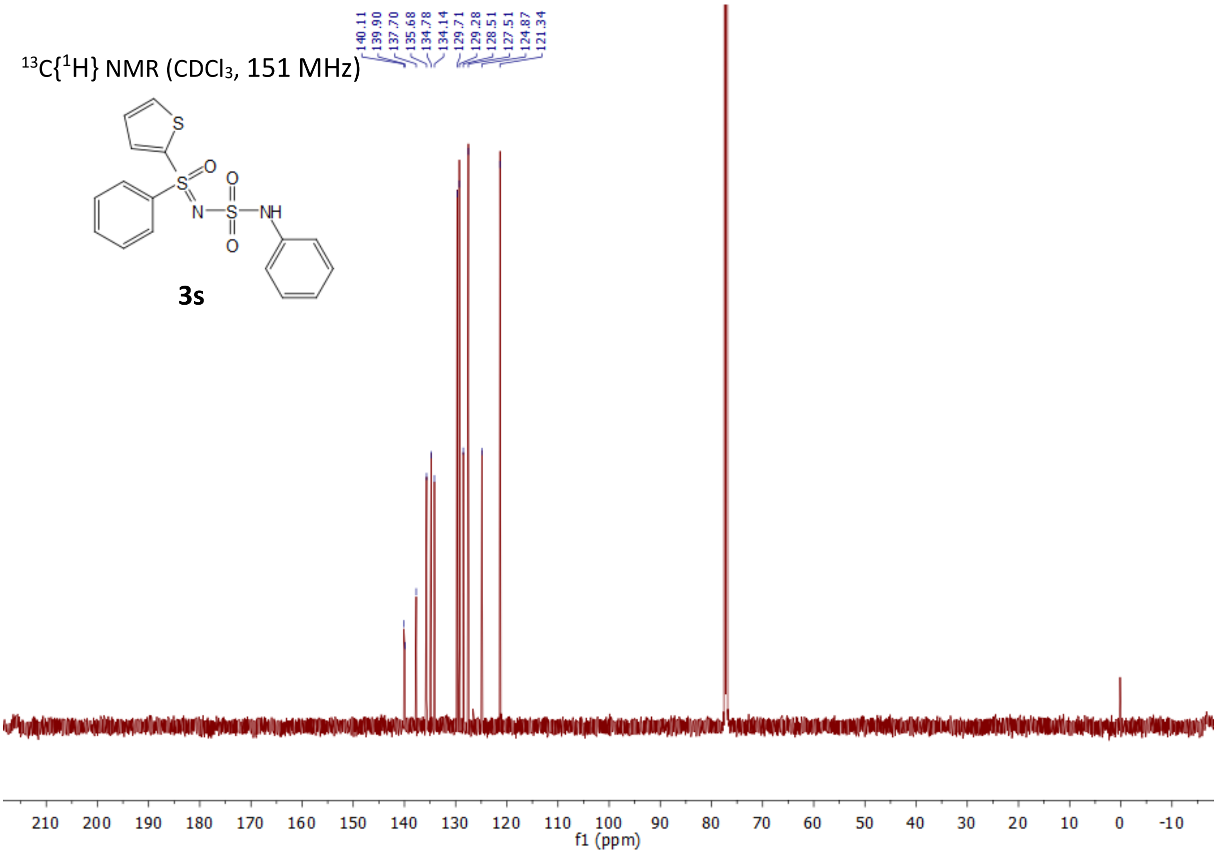
13C{1H} NMR (CDCl3, 151 MHz)
140.11
139.90
137.70
135.68
134.78
134.14
129.71
129.28
128.51
127.51
124.87
121.34
3s
f1 (ppm)

^{1}H NMR ($CDCl_3$, 600 MHz)

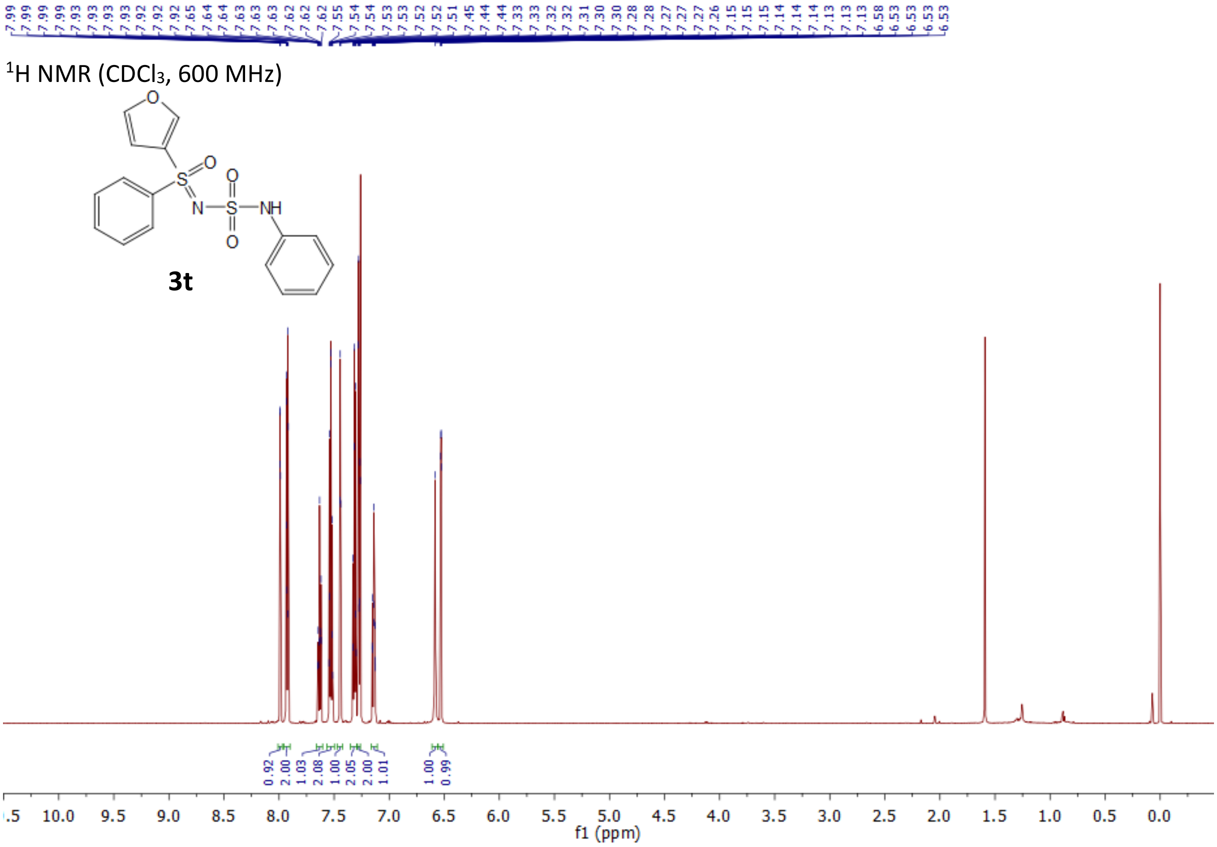

$^{13}C\{^{1}H\}$ NMR ($CDCl_3$, 151 MHz)

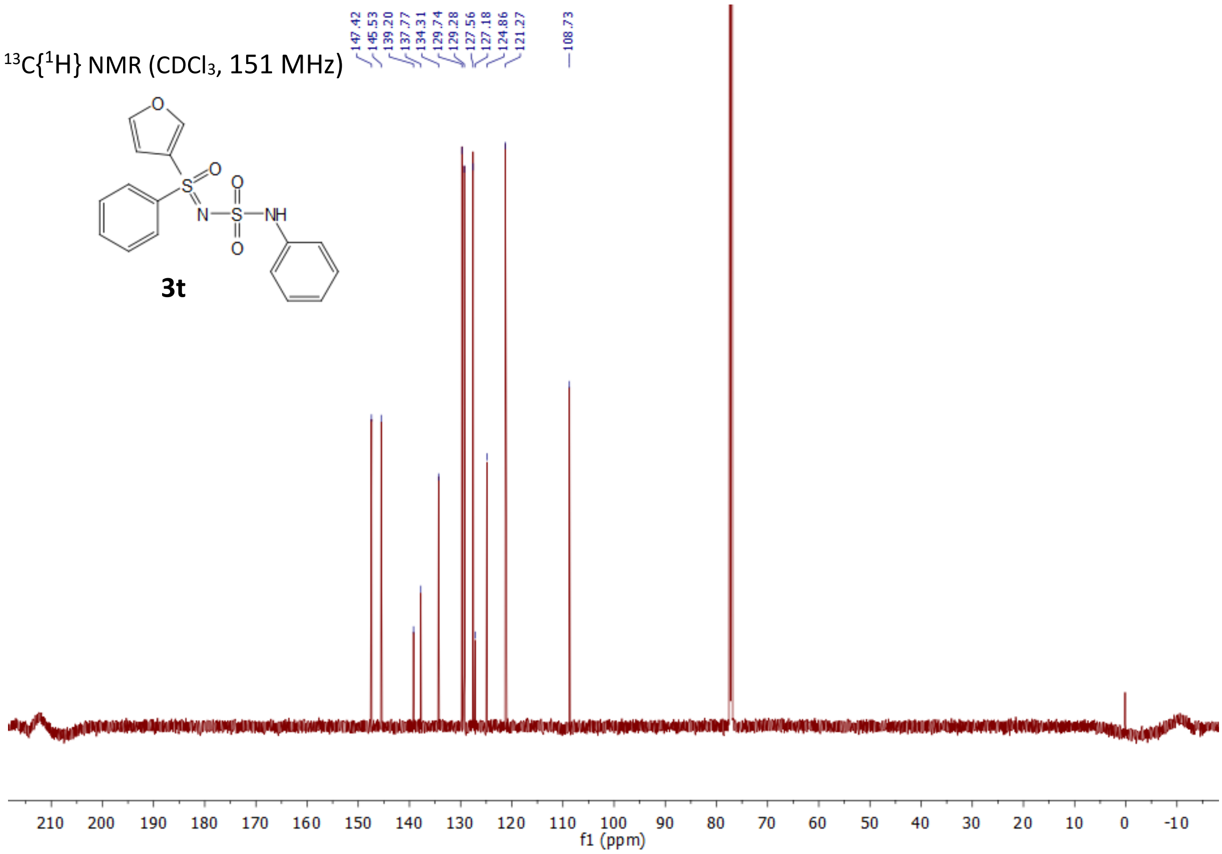

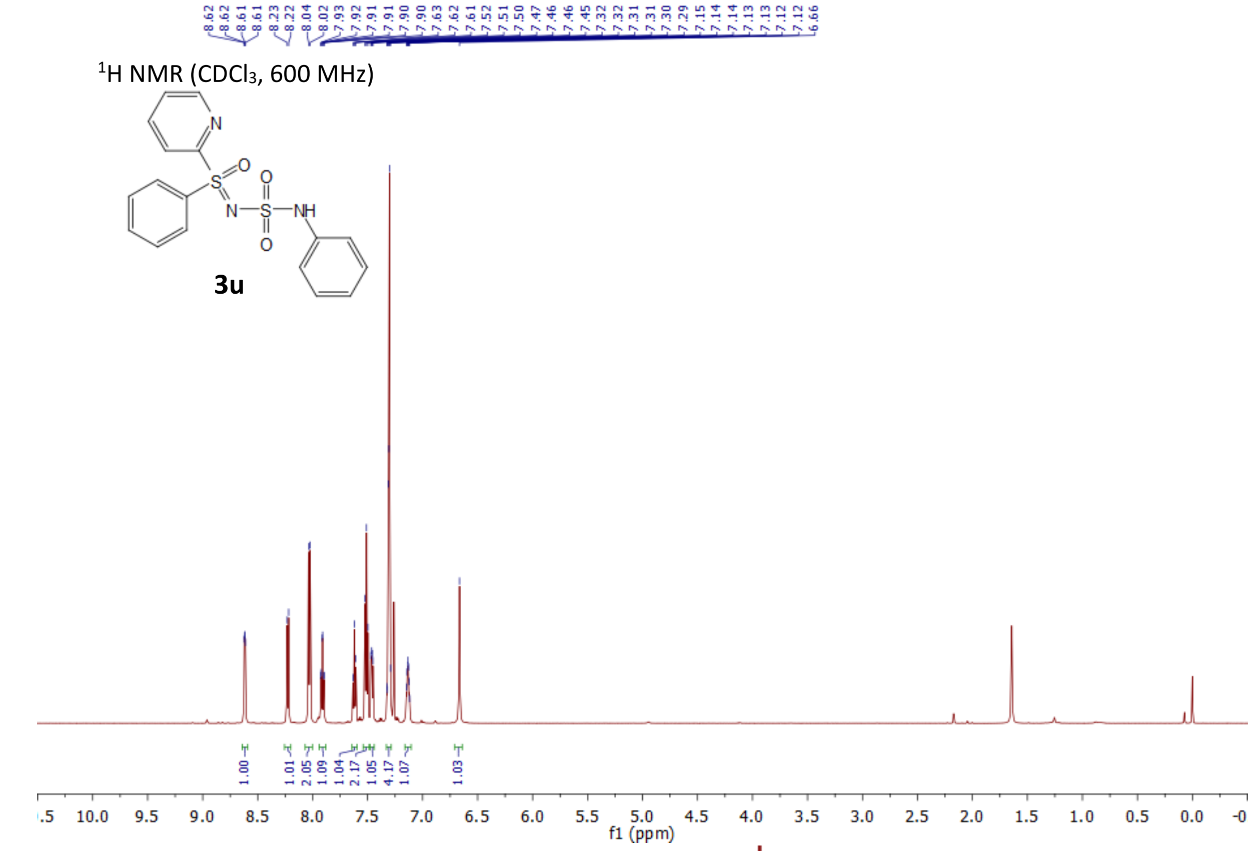
8.62 8.62 8.61 8.61 8.23 8.22 8.04 8.02 7.93 7.92 7.91 7.91 7.90 7.63 7.62 7.61 7.52 7.51 7.50 7.47 7.46 7.46 7.45 7.32 7.32 7.31 7.30 7.29 7.15 7.14 7.14 7.13 7.13 7.12 7.12 6.66
^{1}H NMR (CDCl$_3$, 600 MHz)
N
S
O
N
O
S
NH
O
3u
1.00 1.01 2.05 1.09 1.04 2.17 1.05 4.17 1.07 1.03
f1 (ppm)

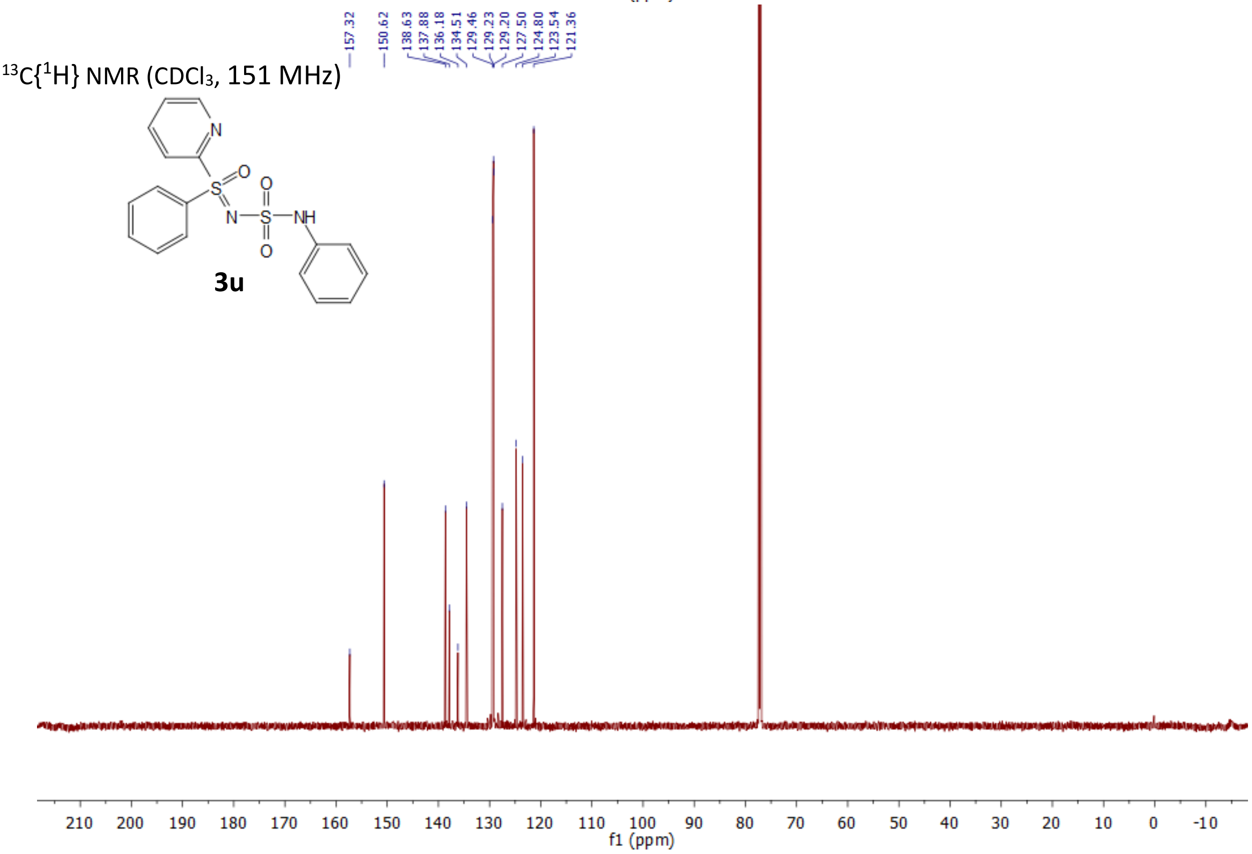
157.32 150.62 138.63 137.88 136.18 134.51 129.46 129.23 129.20 127.50 124.80 123.54 121.36
^{13}C{^{1}H} NMR (CDCl$_3$, 151 MHz)
N
S
O
N
O
S
NH
O
3u
f1 (ppm)

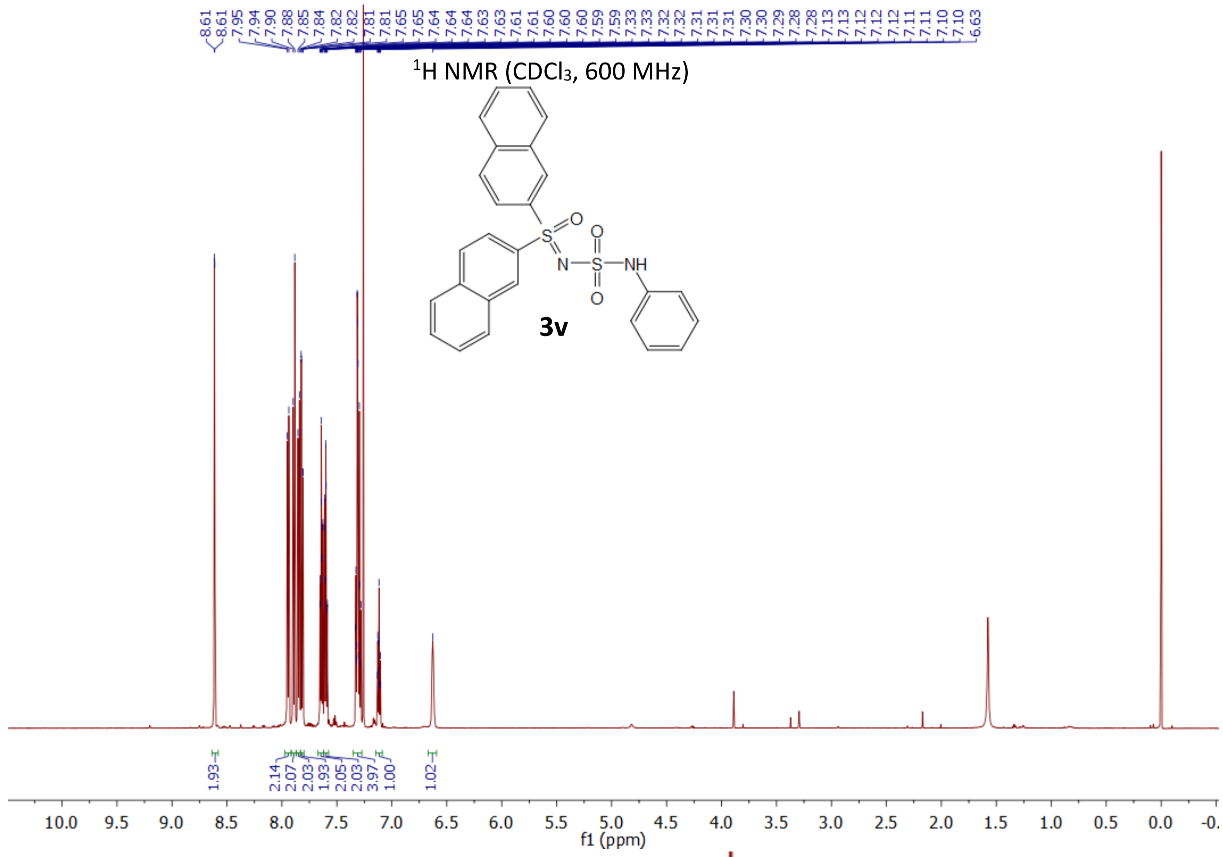
8.61
8.61
7.95
7.94
7.90
7.88
7.85
7.84
7.82
7.82
7.81
7.81
7.65
7.64
7.64
7.64
7.63
7.63
7.61
7.61
7.60
7.60
7.60
7.59
7.59
7.33
7.33
7.32
7.32
7.31
7.31
7.31
7.30
7.30
7.29
7.28
7.28
7.13
7.13
7.12
7.12
7.12
7.11
7.10
7.10
6.63
1H NMR (CDCl3, 600 MHz)
O
S
O
N
S
NH
O
3v
1.93
2.14
2.07
2.03
1.93
2.05
2.03
3.97
1.00
1.02
10.0 9.5 9.0 8.5 8.0 7.5 7.0 6.5 6.0 5.5 5.0 4.5 4.0 3.5 3.0 2.5 2.0 1.5 1.0 0.5 0.0 -0.
f1 (ppm)

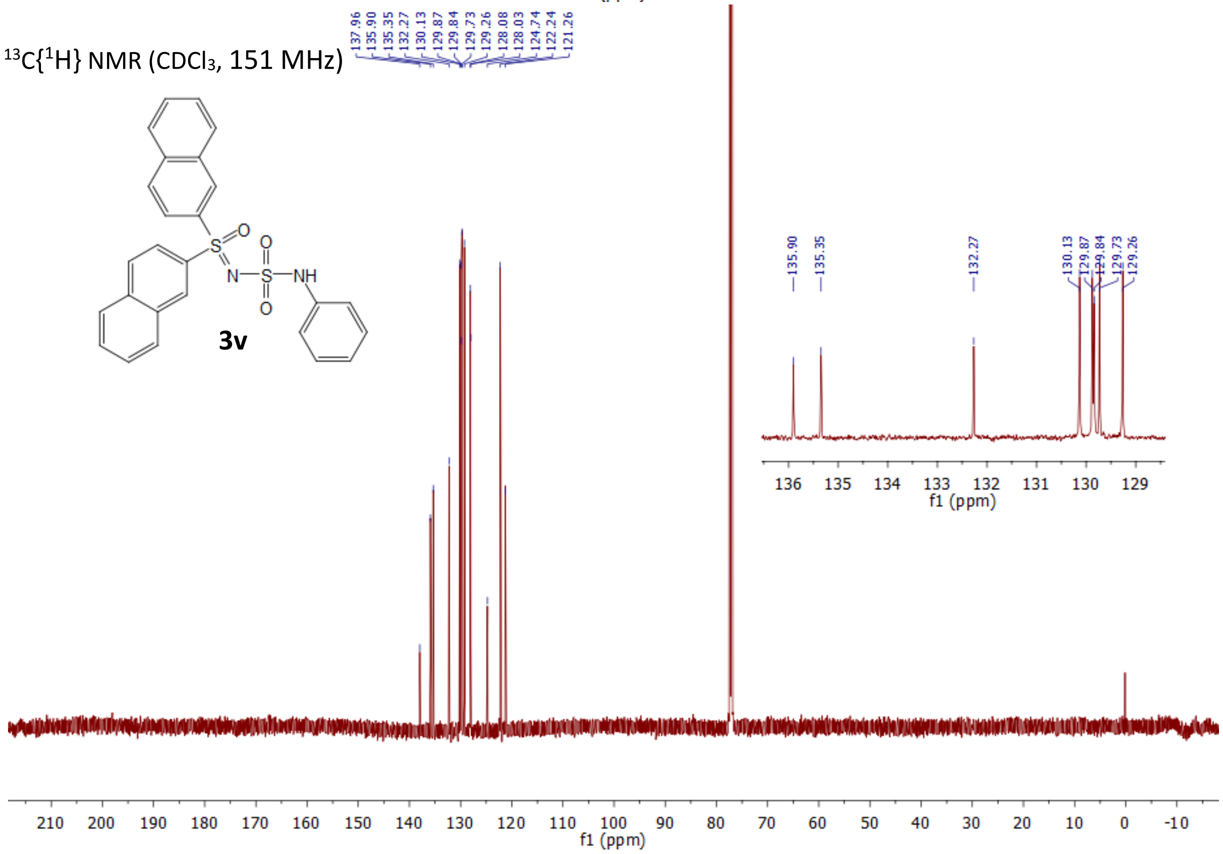
13C{1H} NMR (CDCl3, 151 MHz)
137.96
135.90
135.35
132.27
130.13
129.87
129.84
129.73
129.26
128.08
128.03
124.74
122.24
121.26
O
S
O
N
S
NH
O
3v
135.90
135.35
132.27
130.13
129.87
129.84
129.73
129.26
136 135 134 133 132 131 130 129
f1 (ppm)
210 200 190 180 170 160 150 140 130 120 110 100 90 80 70 60 50 40 30 20 10 0 -10
f1 (ppm)

^{1}H NMR ($CDCl_3$, 600 MHz)

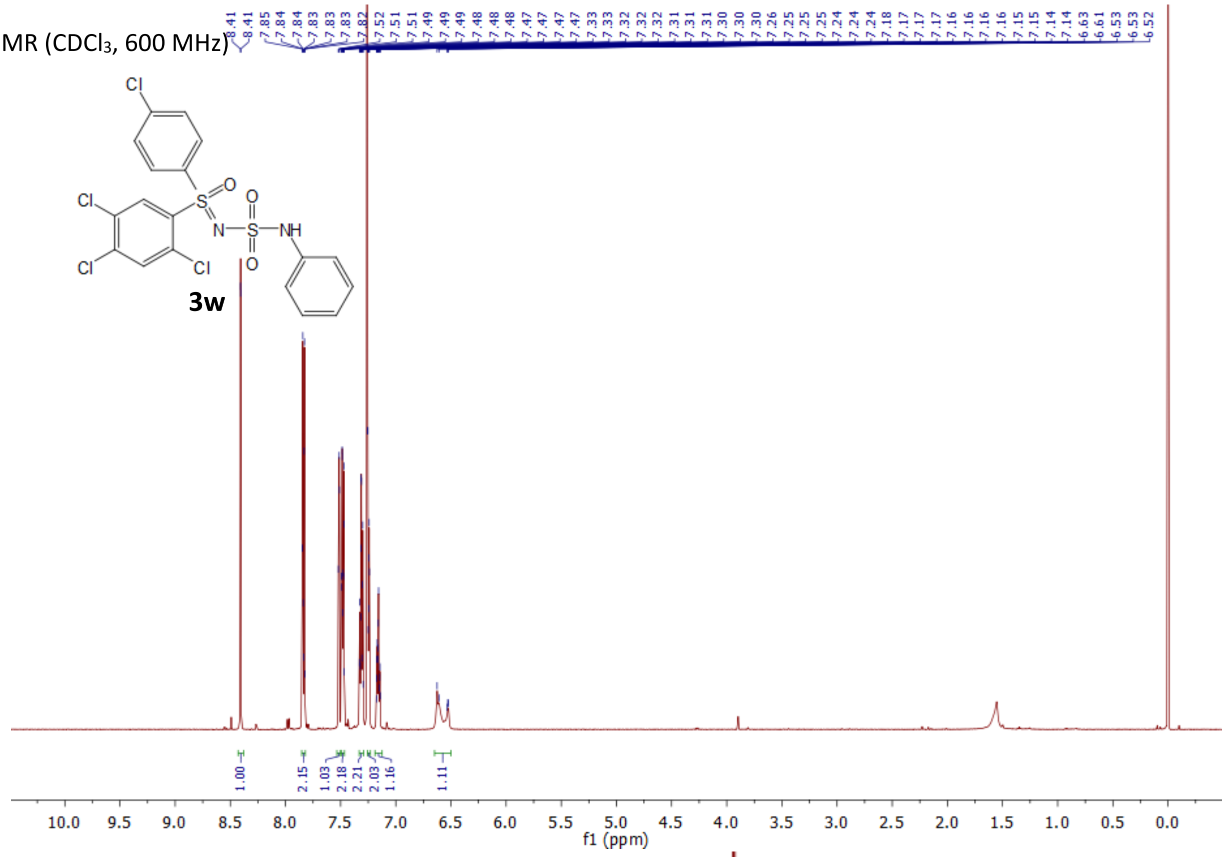

$^{13}C\{^{1}H\}$ NMR ($CDCl_3$, 151 MHz)

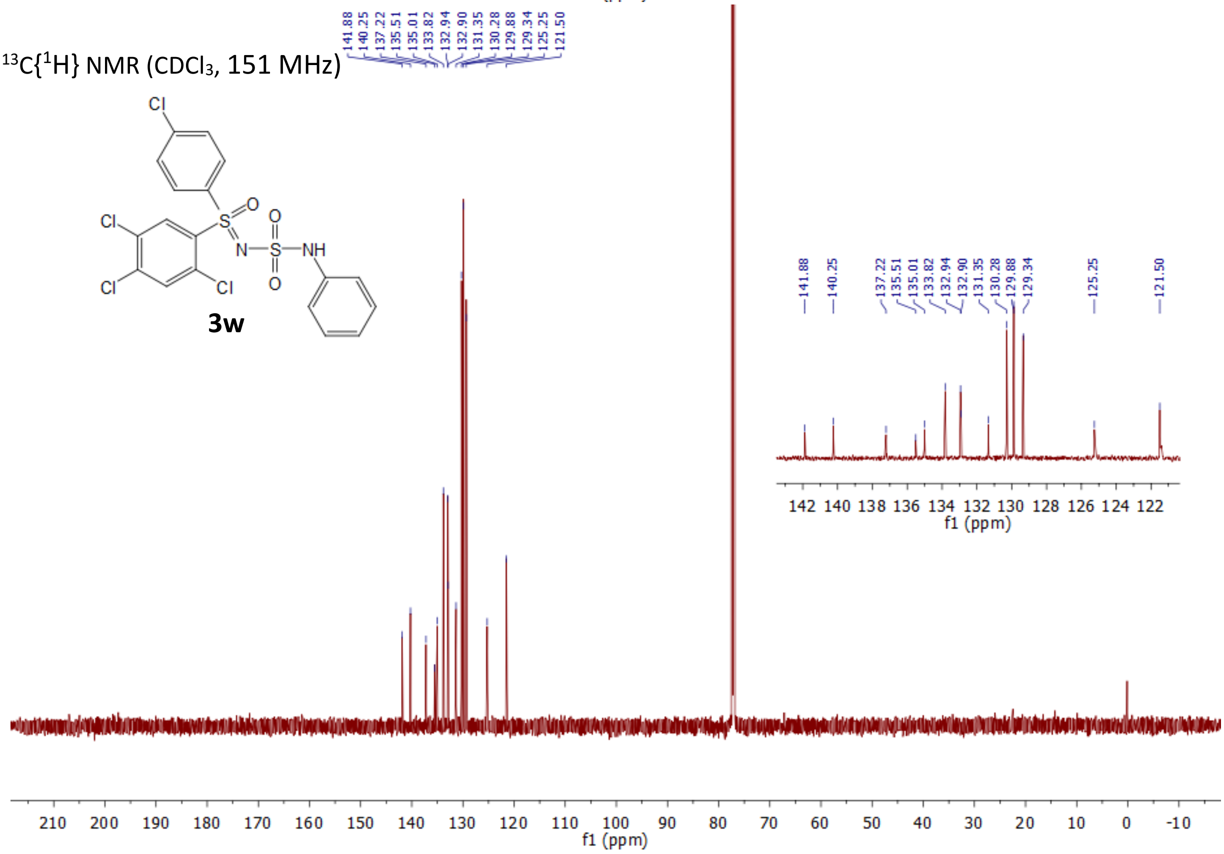

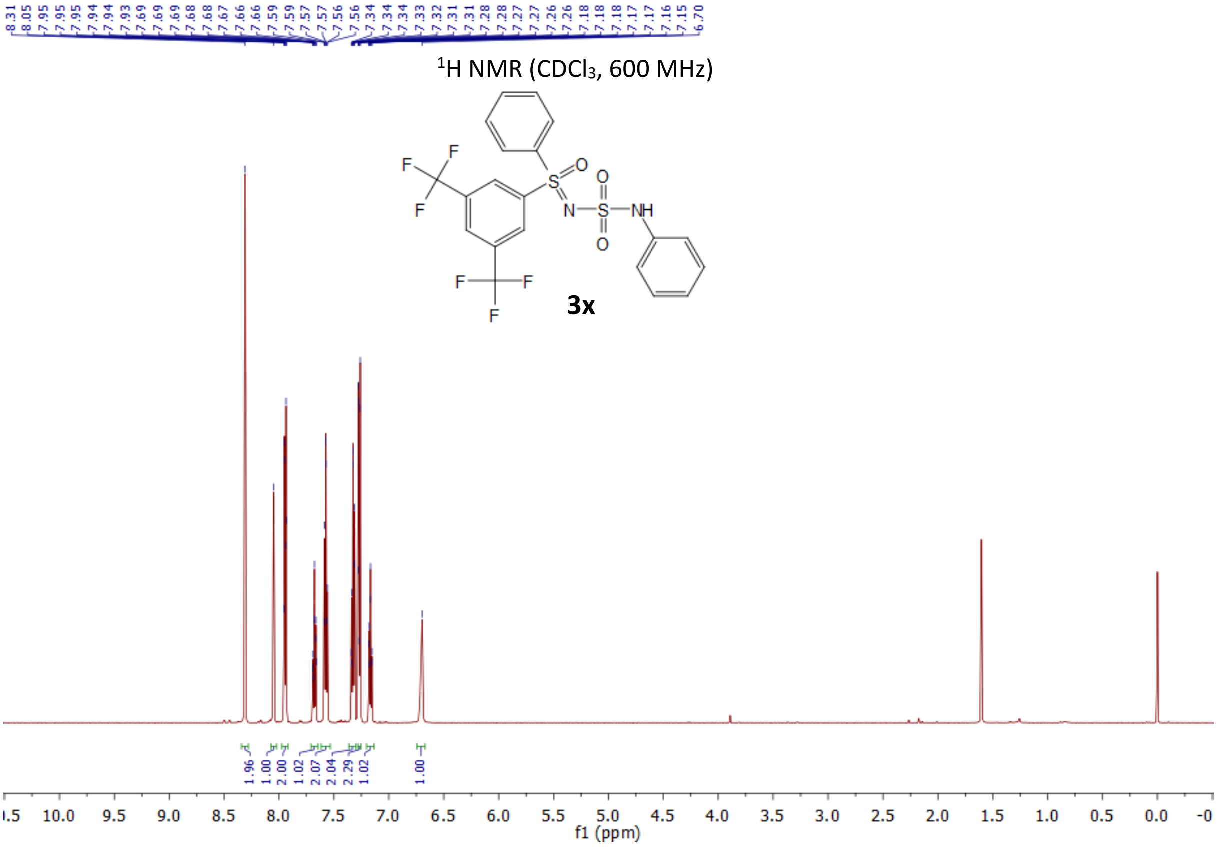
8.31
8.05
7.95
7.95
7.95
7.94
7.94
7.93
7.69
7.69
7.69
7.68
7.67
7.66
7.66
7.59
7.59
7.57
7.57
7.56
7.56
7.34
7.34
7.34
7.33
7.32
7.31
7.31
7.28
7.28
7.27
7.27
7.26
7.26
7.18
7.18
7.18
7.17
7.17
7.16
7.15
6.70
1H NMR (CDCl3, 600 MHz)
3x
1.96
1.00
2.00
1.02
2.07
2.04
2.29
1.02
1.00
f1 (ppm)

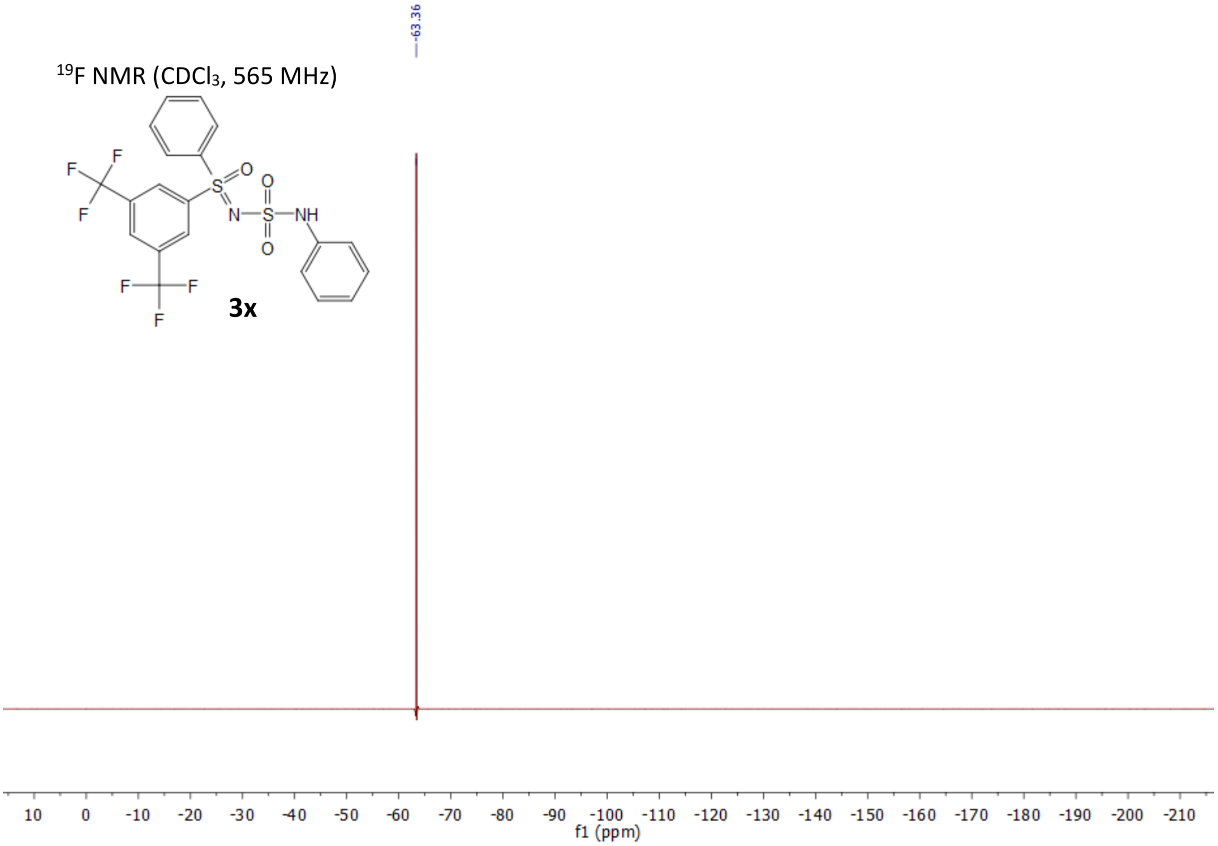
-63.36
19F NMR (CDCl3, 565 MHz)
3x
f1 (ppm)

$^{13}C\{^1H\}$ NMR ($CDCl_3$, 151 MHz)

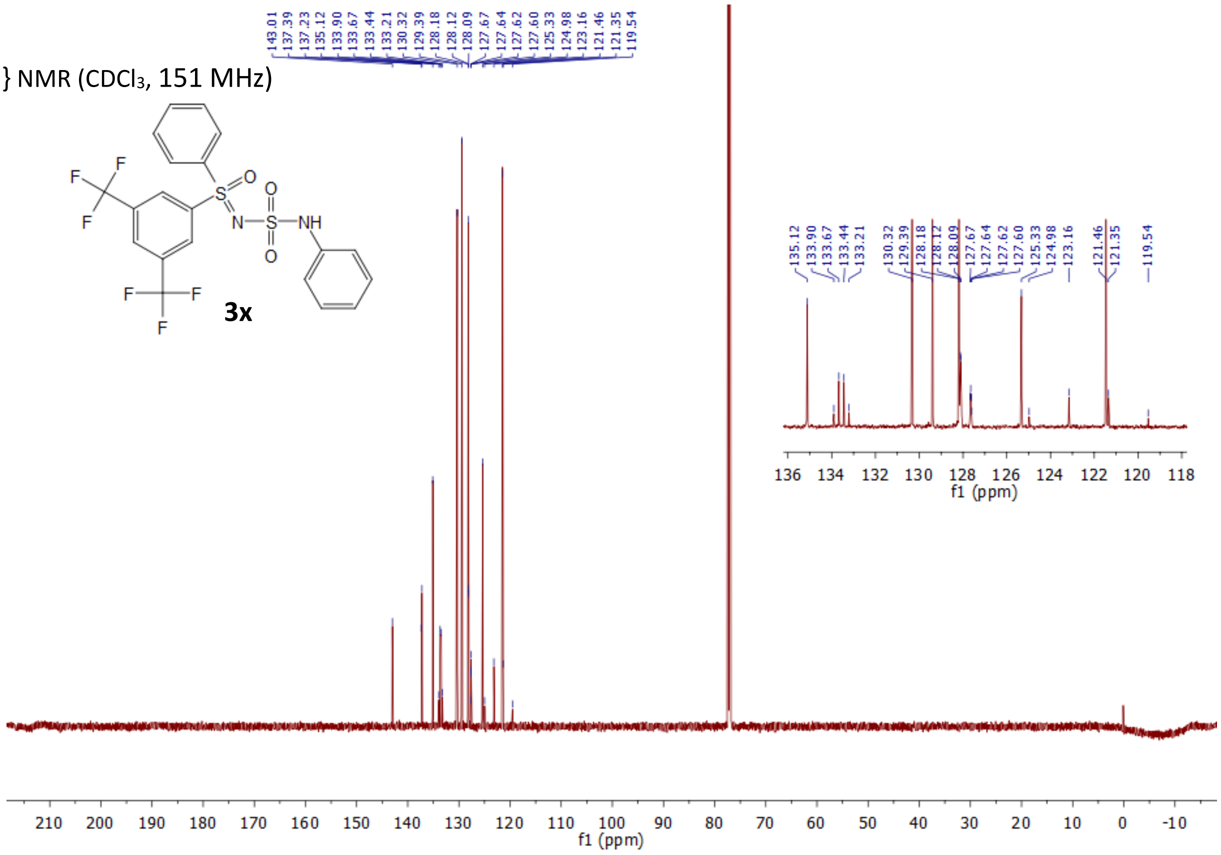

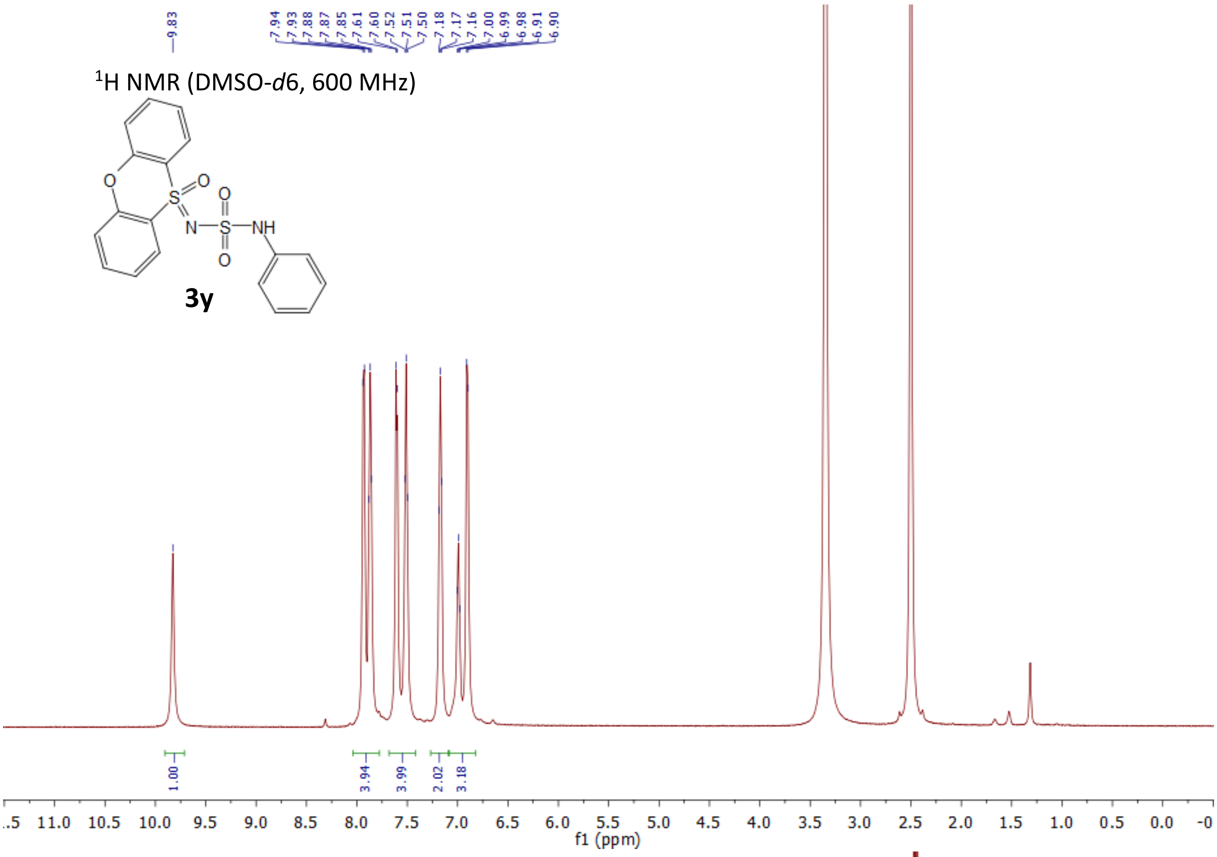
9.83
7.94
7.93
7.88
7.87
7.85
7.61
7.60
7.52
7.51
7.50
7.18
7.17
7.16
7.00
6.99
6.98
6.91
6.90
^{1}H NMR (DMSO-*d*6, 600 MHz)
3y
1.00
3.94
3.99
2.02
3.18
f1 (ppm)

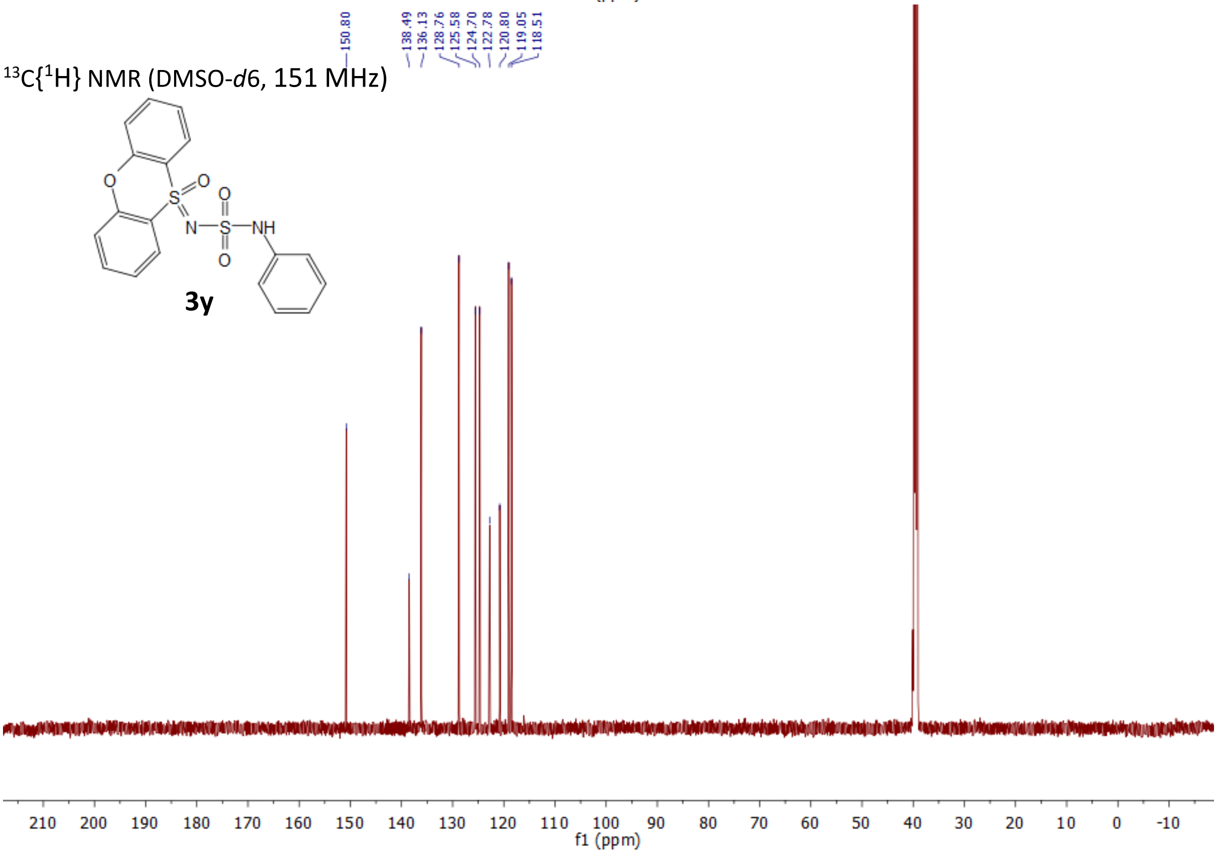
150.80
138.49
136.13
128.76
125.58
124.70
122.78
120.80
119.05
118.51
^{13}C{^{1}H} NMR (DMSO-*d*6, 151 MHz)
3y
f1 (ppm)

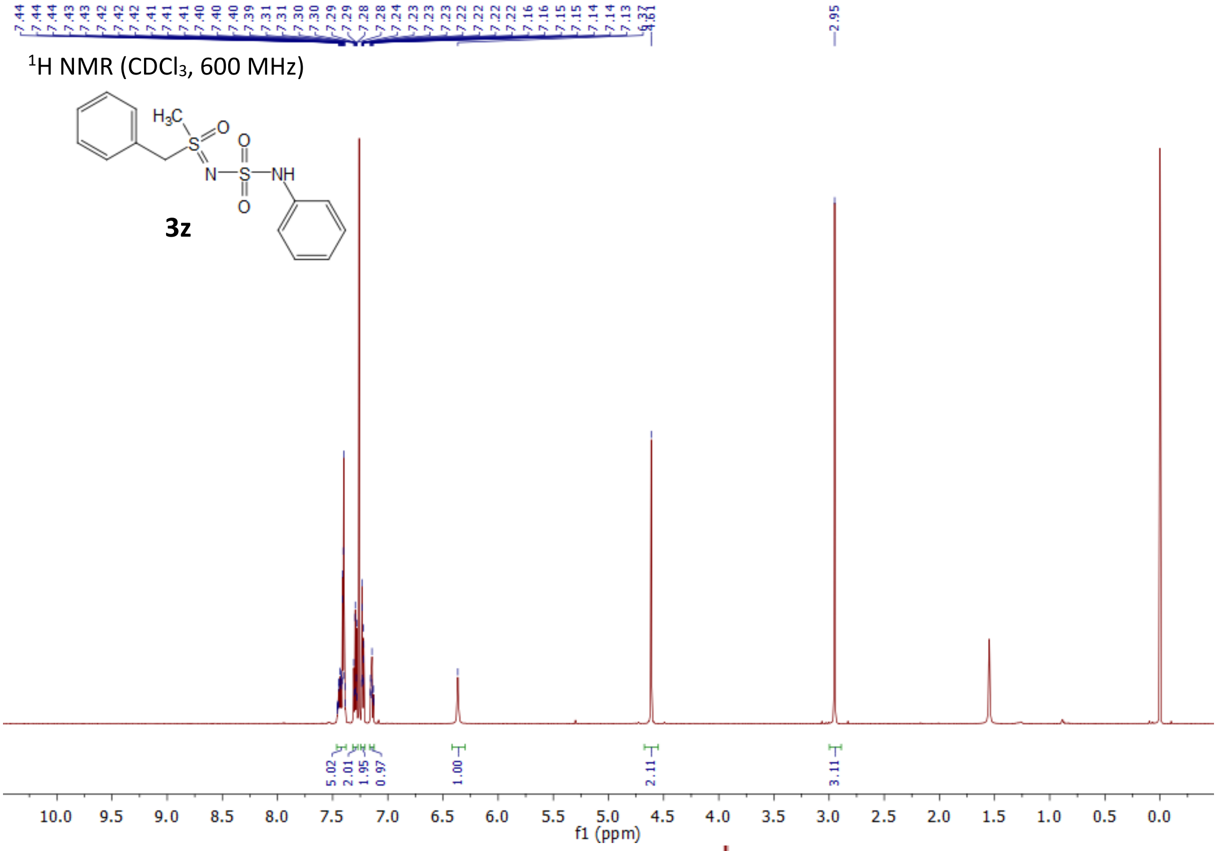

7.44
7.44
7.44
7.43
7.43
7.42
7.42
7.42
7.41
7.41
7.41
7.40
7.40
7.40
7.39
7.31
7.31
7.30
7.30
7.29
7.29
7.28
7.28
7.24
7.23
7.23
7.23
7.23
7.22
7.22
7.22
7.22
7.22
7.16
7.16
7.15
7.15
7.14
7.14
7.13
6.37
4.61
2.95
^{1}H NMR ($CDCl_3$, 600 MHz)
H_3C
S
O
N
NH
3z
5.02
2.01
1.95
0.97
1.00
2.11
3.11
10.0
9.5
9.0
8.5
8.0
7.5
7.0
6.5
6.0
5.5
5.0
4.5
4.0
3.5
3.0
2.5
2.0
1.5
1.0
0.5
0.0
f1 (ppm)

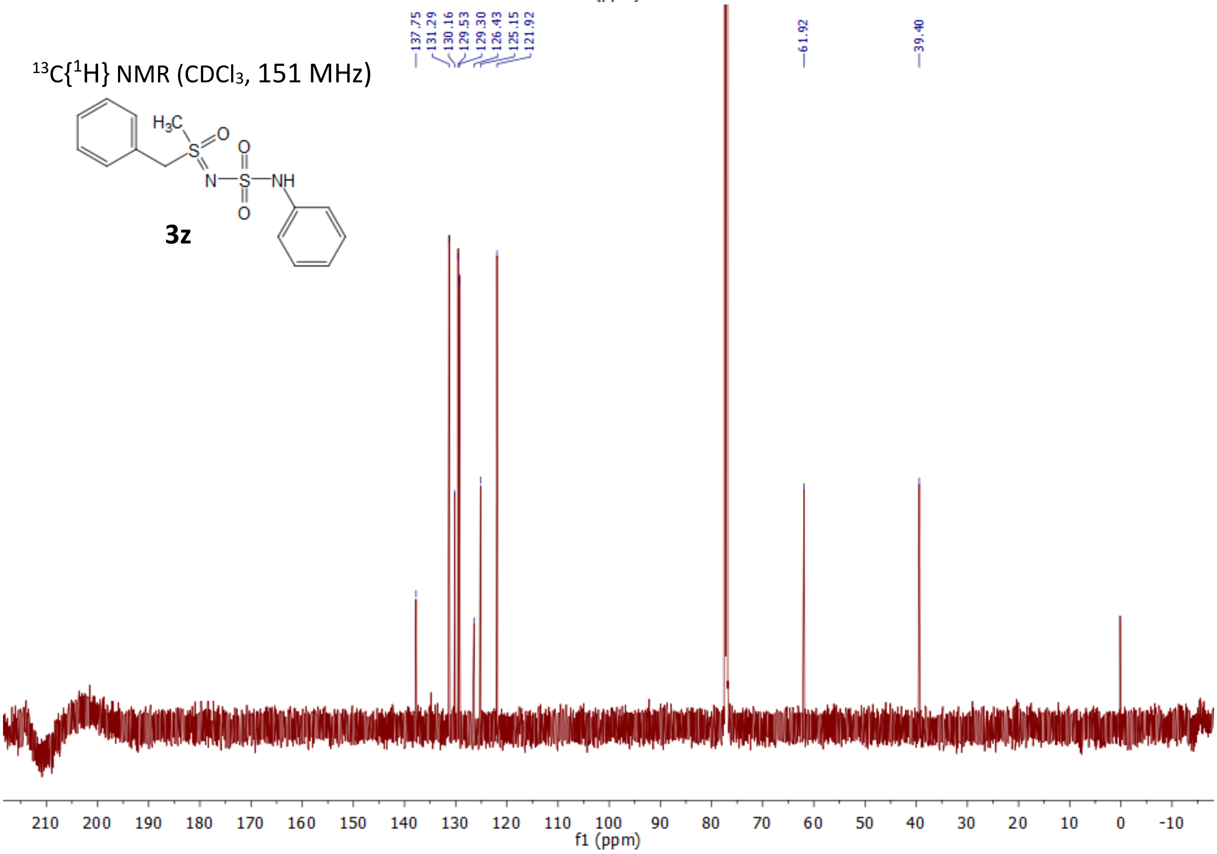

137.75
131.29
130.16
129.53
129.30
126.43
125.15
121.92
61.92
39.40
^{13}C{^{1}H} NMR ($CDCl_3$, 151 MHz)
H_3C
S
O
N
NH
3z
210
200
190
180
170
160
150
140
130
120
110
100
90
80
70
60
50
40
30
20
10
0
-10
f1 (ppm)

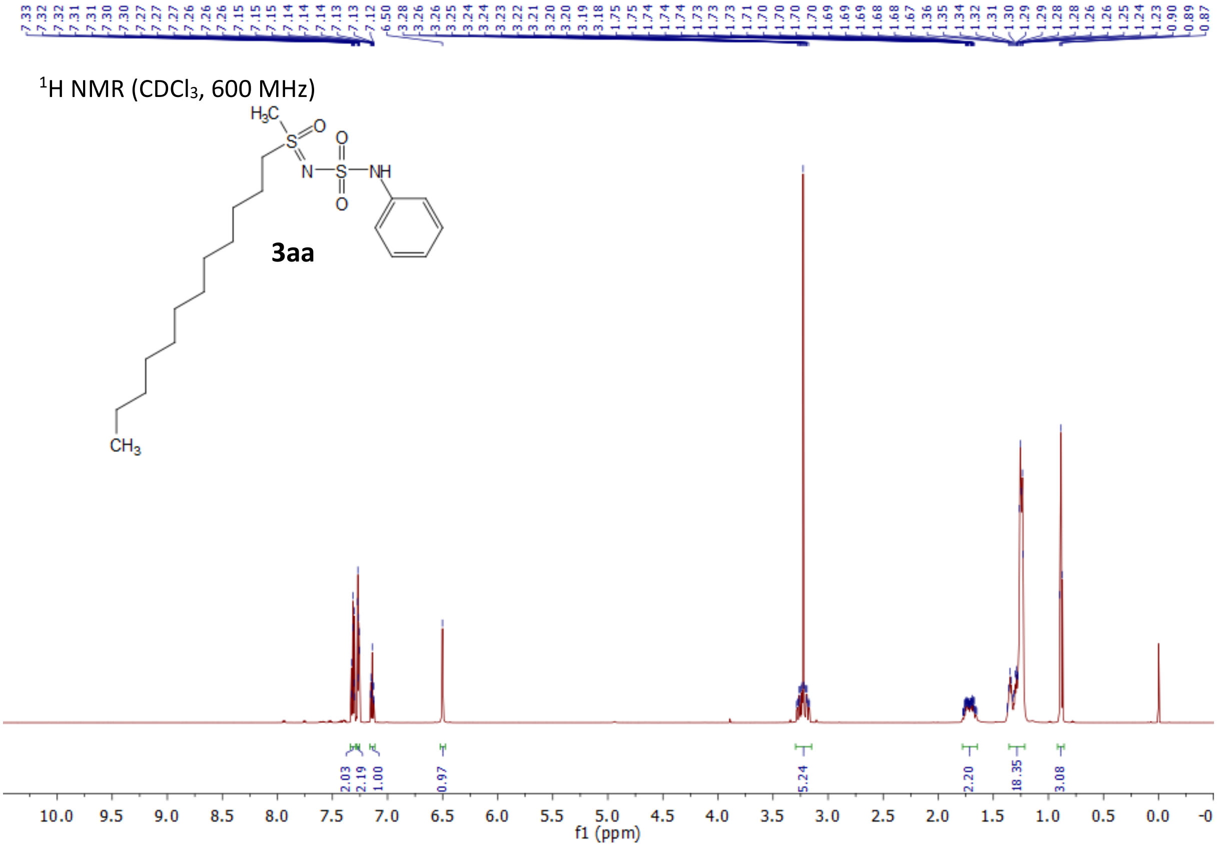

1H NMR (CDCl3, 600 MHz)
H3C
S
O
N
O
S
NH
O
3aa
CH3
7.33
7.32
7.31
7.31
7.30
7.30
7.27
7.27
7.27
7.26
7.26
7.26
7.15
7.15
7.15
7.14
7.14
7.14
7.13
7.13
7.12
6.50
3.28
3.26
3.26
3.25
3.24
3.24
3.23
3.22
3.21
3.20
3.20
3.19
3.18
1.75
1.75
1.74
1.74
1.74
1.73
1.73
1.73
1.71
1.70
1.70
1.70
1.70
1.69
1.69
1.69
1.68
1.67
1.36
1.35
1.34
1.32
1.31
1.30
1.29
1.29
1.28
1.28
1.26
1.26
1.25
1.24
1.23
0.90
0.89
0.87
2.03
2.19
1.00
0.97
5.24
2.20
18.35
3.08
10.0 9.5 9.0 8.5 8.0 7.5 7.0 6.5 6.0 5.5 5.0 4.5 4.0 3.5 3.0 2.5 2.0 1.5 1.0 0.5 0.0 -0
f1 (ppm)

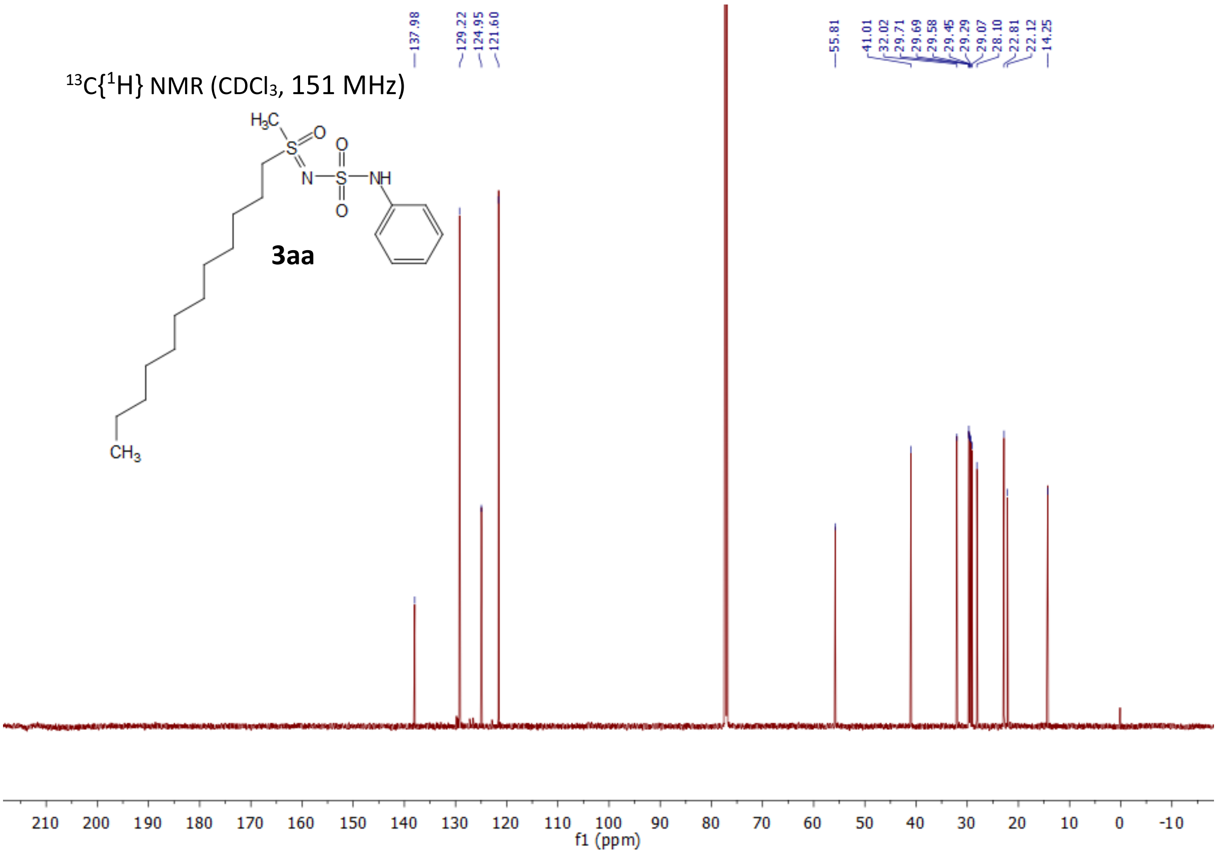

13C{1H} NMR (CDCl3, 151 MHz)
H3C
S
O
N
O
S
NH
O
3aa
CH3
137.98
129.22
124.95
121.60
55.81
41.01
32.02
29.71
29.69
29.58
29.45
29.29
29.07
28.10
22.81
22.12
14.25
210 200 190 180 170 160 150 140 130 120 110 100 90 80 70 60 50 40 30 20 10 0 -10
f1 (ppm)

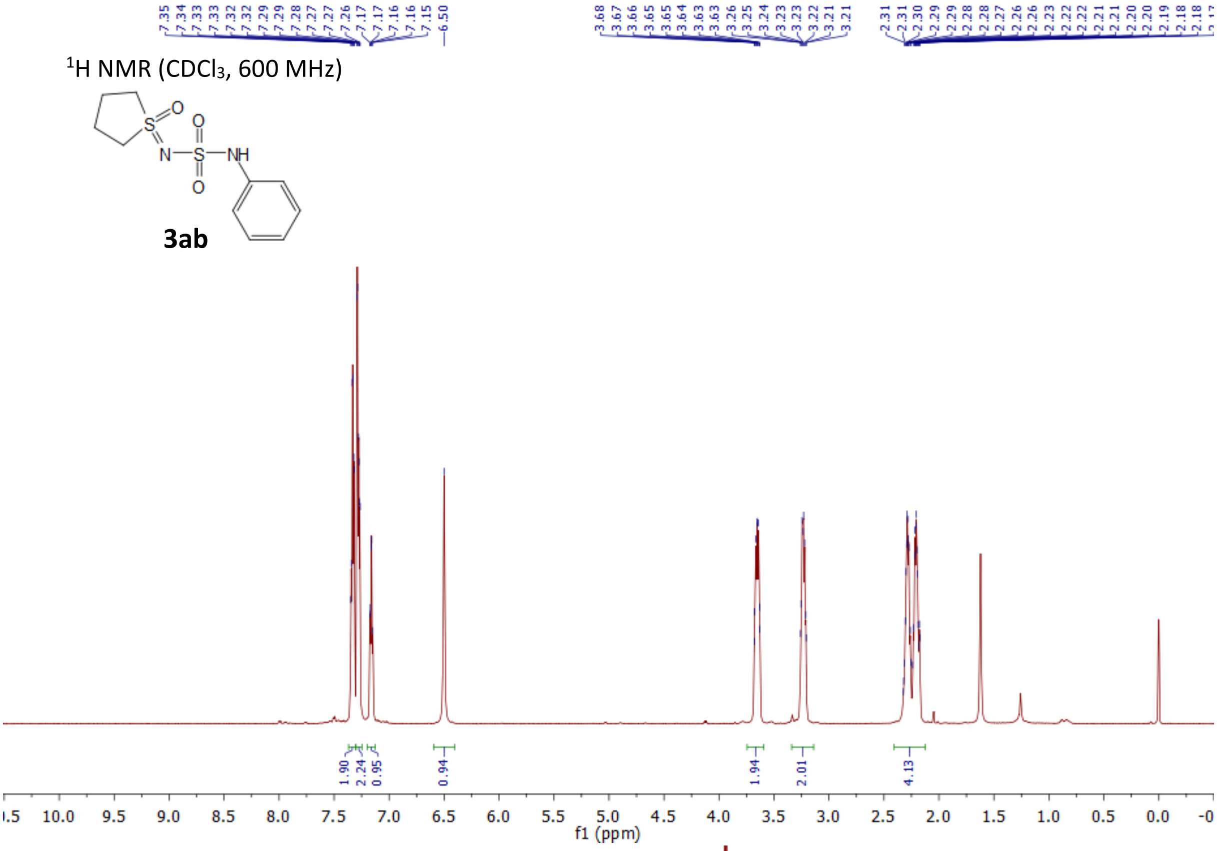

^{1}H NMR ($CDCl_3$, 600 MHz)
3ab
7.35 7.34 7.33 7.33 7.32 7.32 7.29 7.29 7.28 7.28 7.27 7.27 7.26 7.17 7.17 7.16 7.16 7.15 6.50
3.68 3.67 3.66 3.65 3.65 3.64 3.63 3.63 3.26 3.25 3.24 3.23 3.23 3.22 3.21 3.21
2.31 2.31 2.30 2.29 2.29 2.28 2.28 2.27 2.26 2.26 2.23 2.22 2.22 2.21 2.21 2.20 2.20 2.19 2.18 2.18
1.90 2.24 0.95 0.94 1.94 2.01 4.13
f1 (ppm)

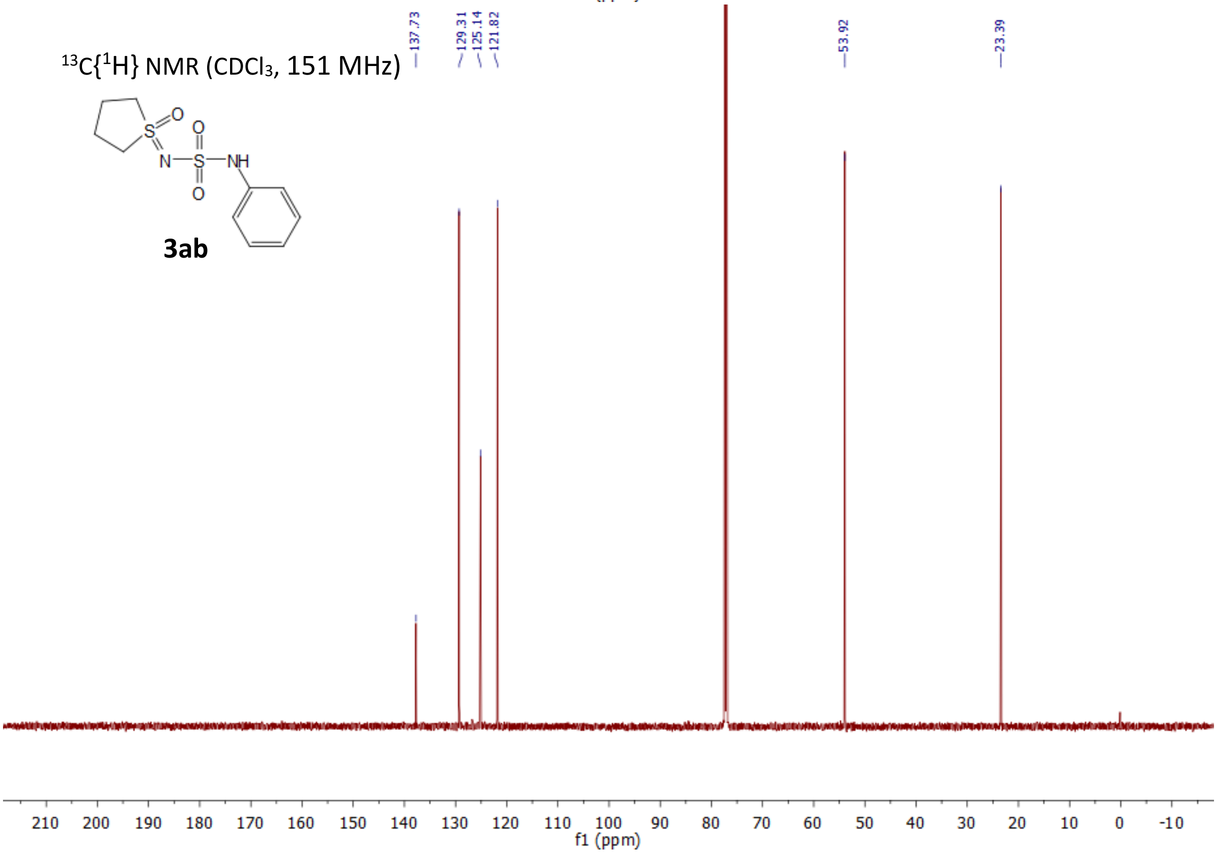

^{13}C{^{1}H} NMR ($CDCl_3$, 151 MHz)
3ab
137.73
129.31
125.14
121.82
53.92
23.39
f1 (ppm)

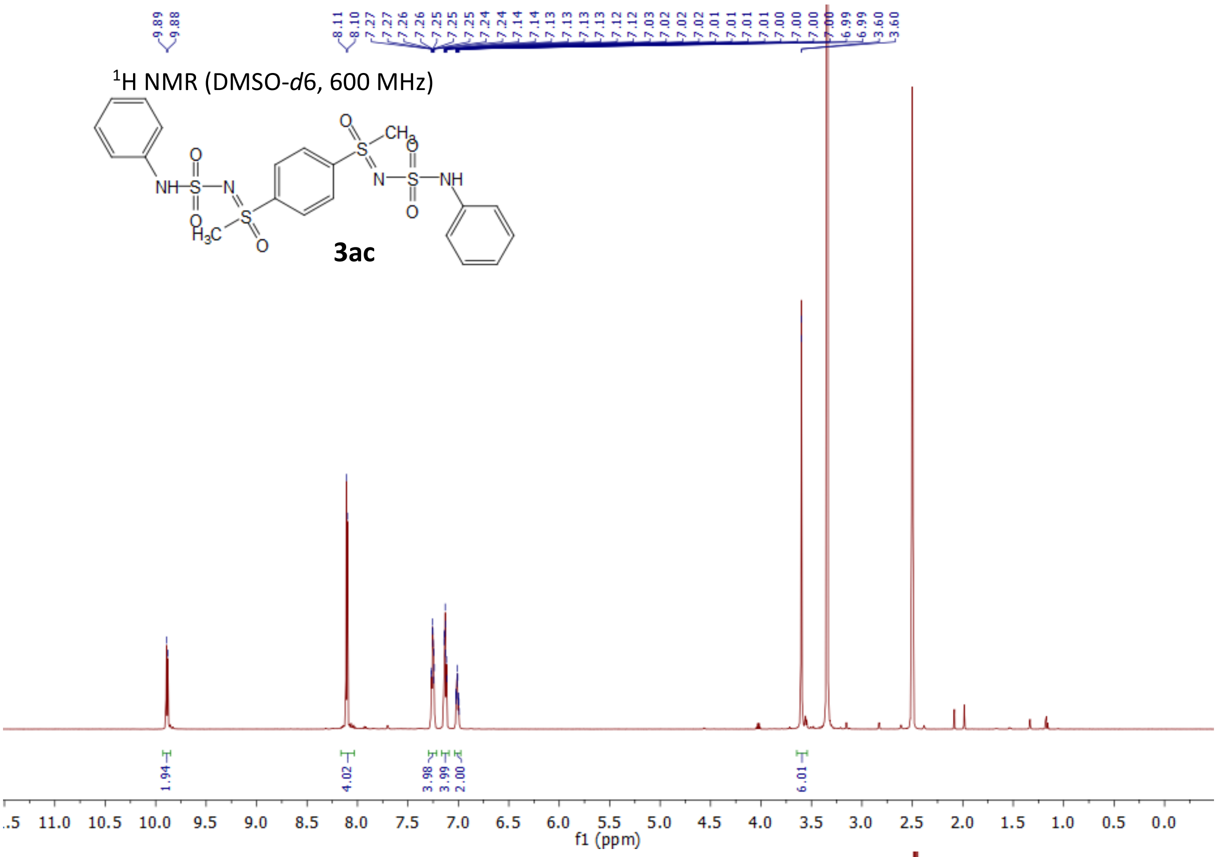
1H NMR (DMSO-d6, 600 MHz)
3ac
f1 (ppm)

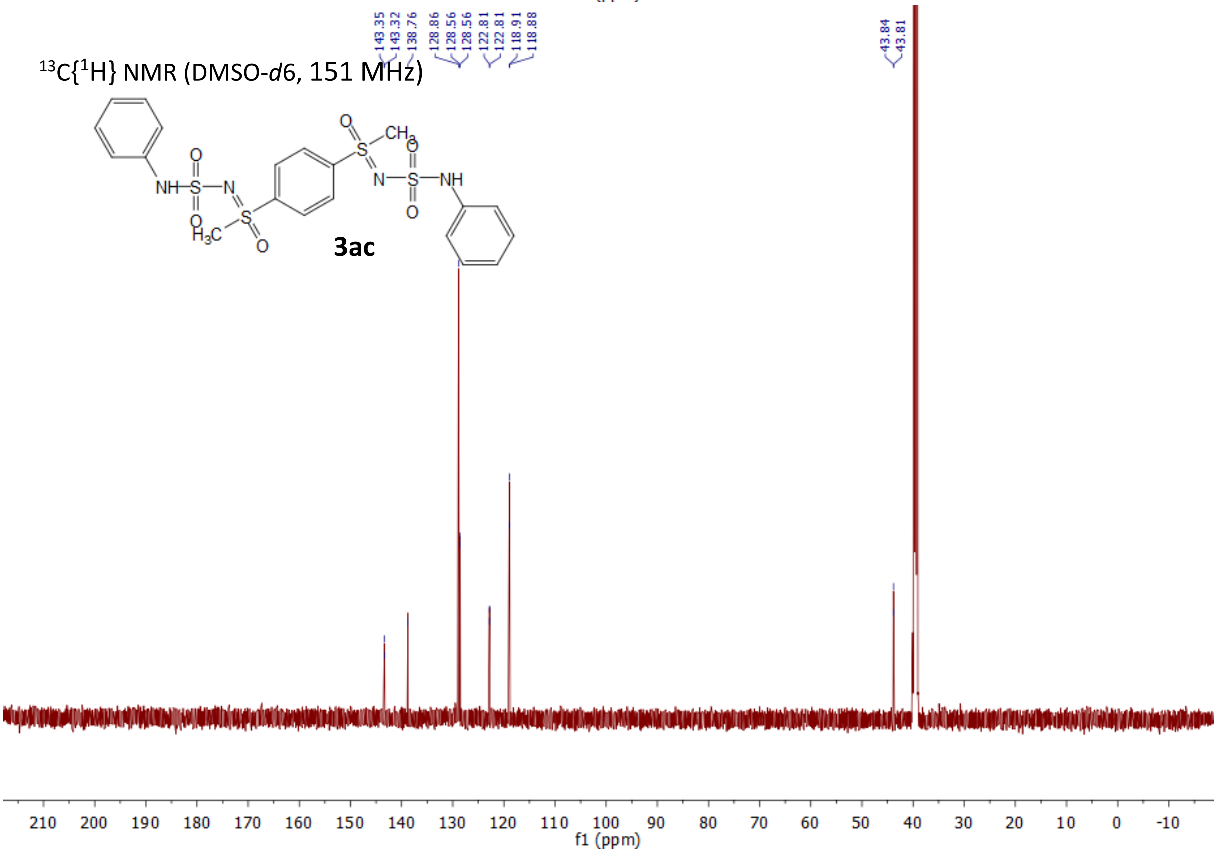
13C{1H} NMR (DMSO-d6, 151 MHz)
3ac
f1 (ppm)

^{1}H NMR (DMSO-*d*6, 600 MHz)

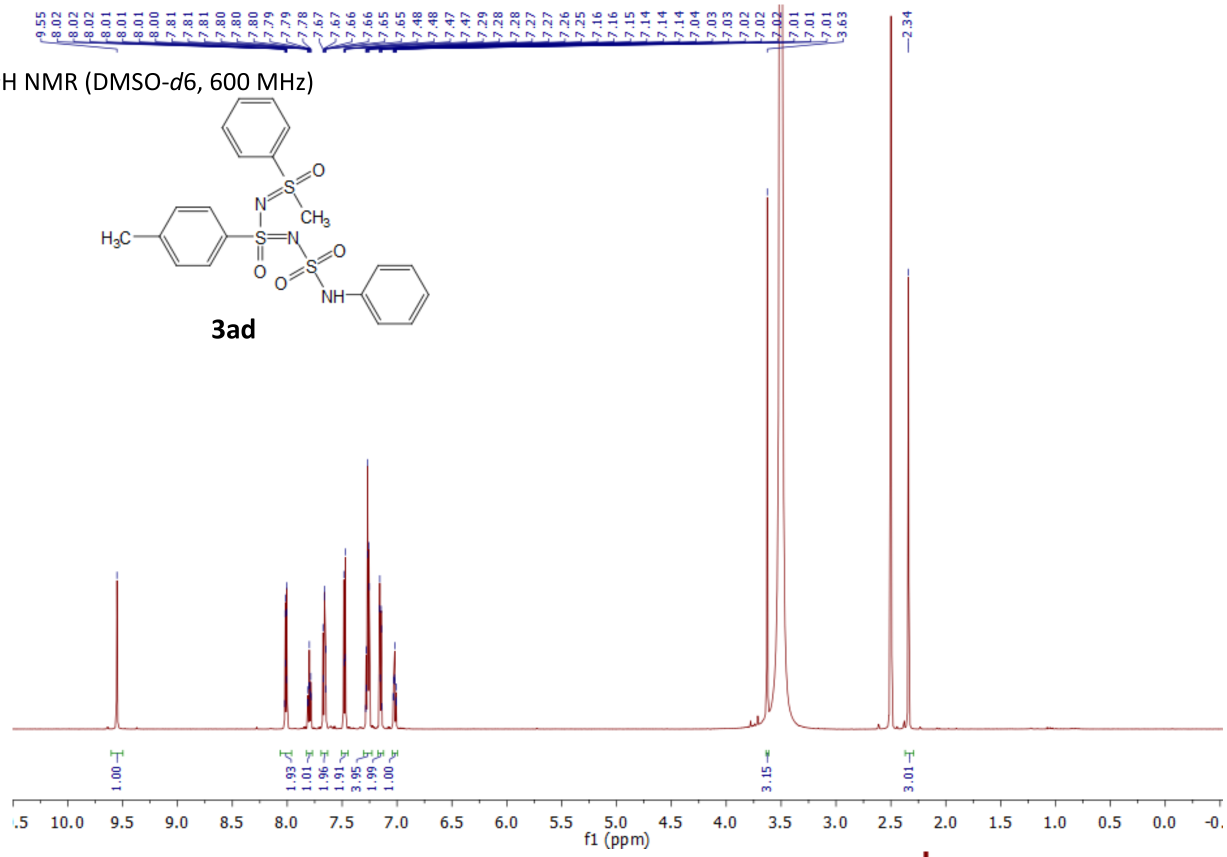

$^{13}C\{^{1}H\}$ NMR (DMSO-*d*6, 151 MHz)

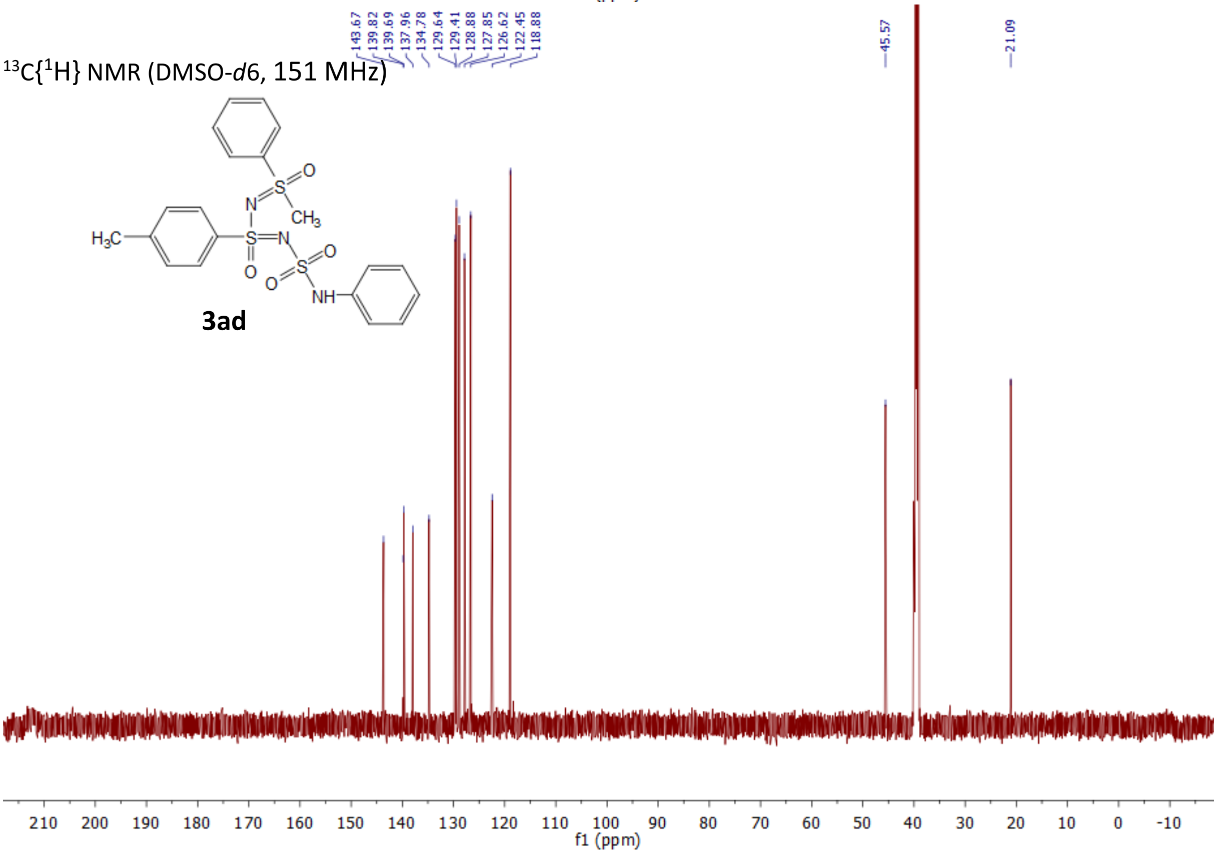

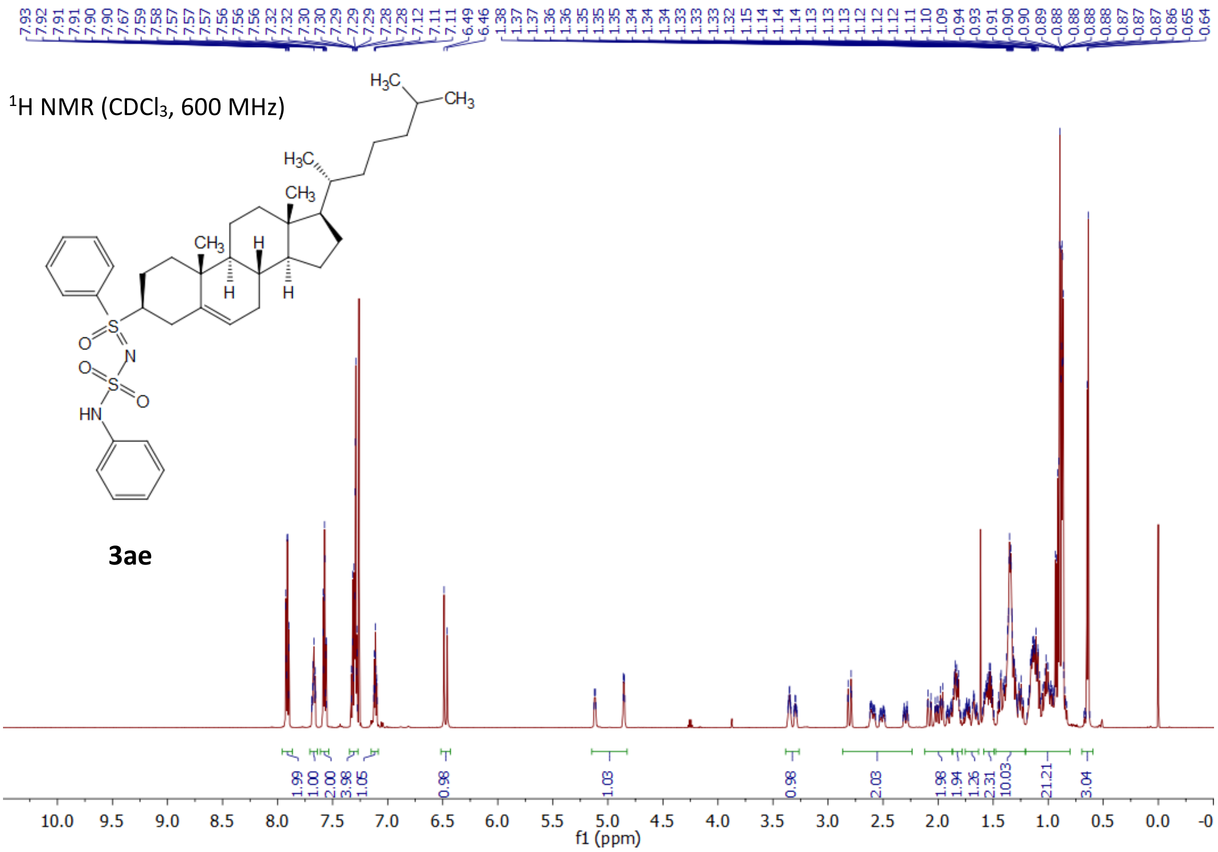

3ae

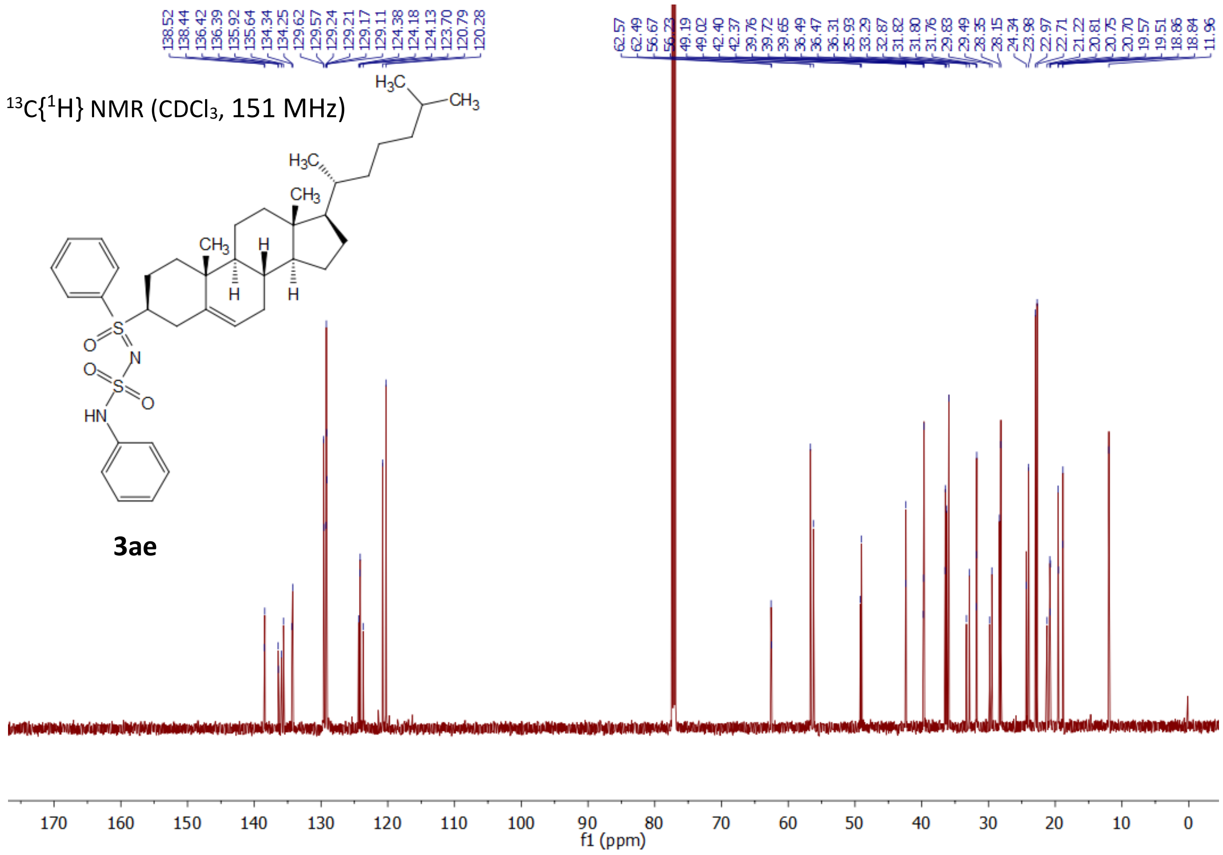

3ae

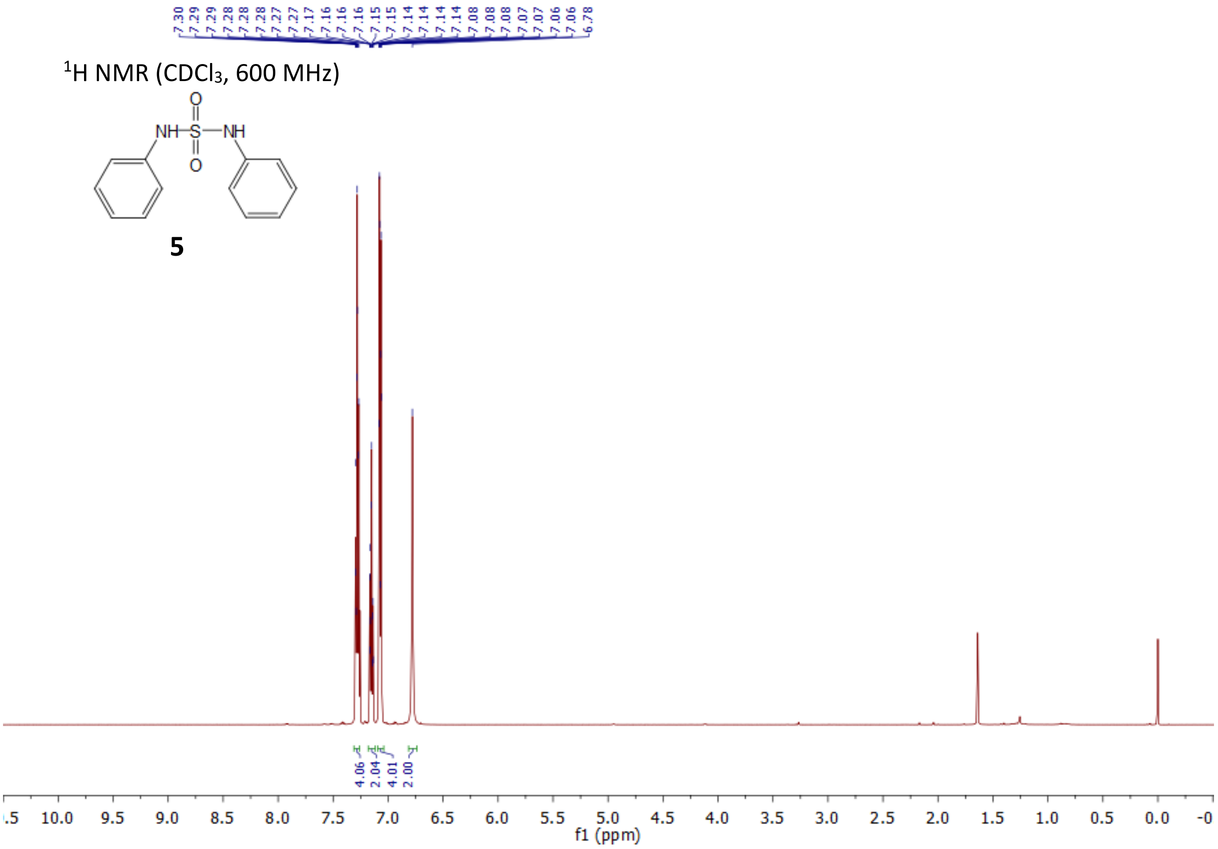

7.30
7.29
7.29
7.28
7.28
7.28
7.27
7.27
7.17
7.16
7.16
7.16
7.15
7.15
7.14
7.14
7.14
7.14
7.08
7.08
7.08
7.07
7.07
7.06
7.06
6.78
¹H NMR (CDCl₃, 600 MHz)
NH
S
NH
O
O
5
4.06
2.04
4.01
2.00
f1 (ppm)

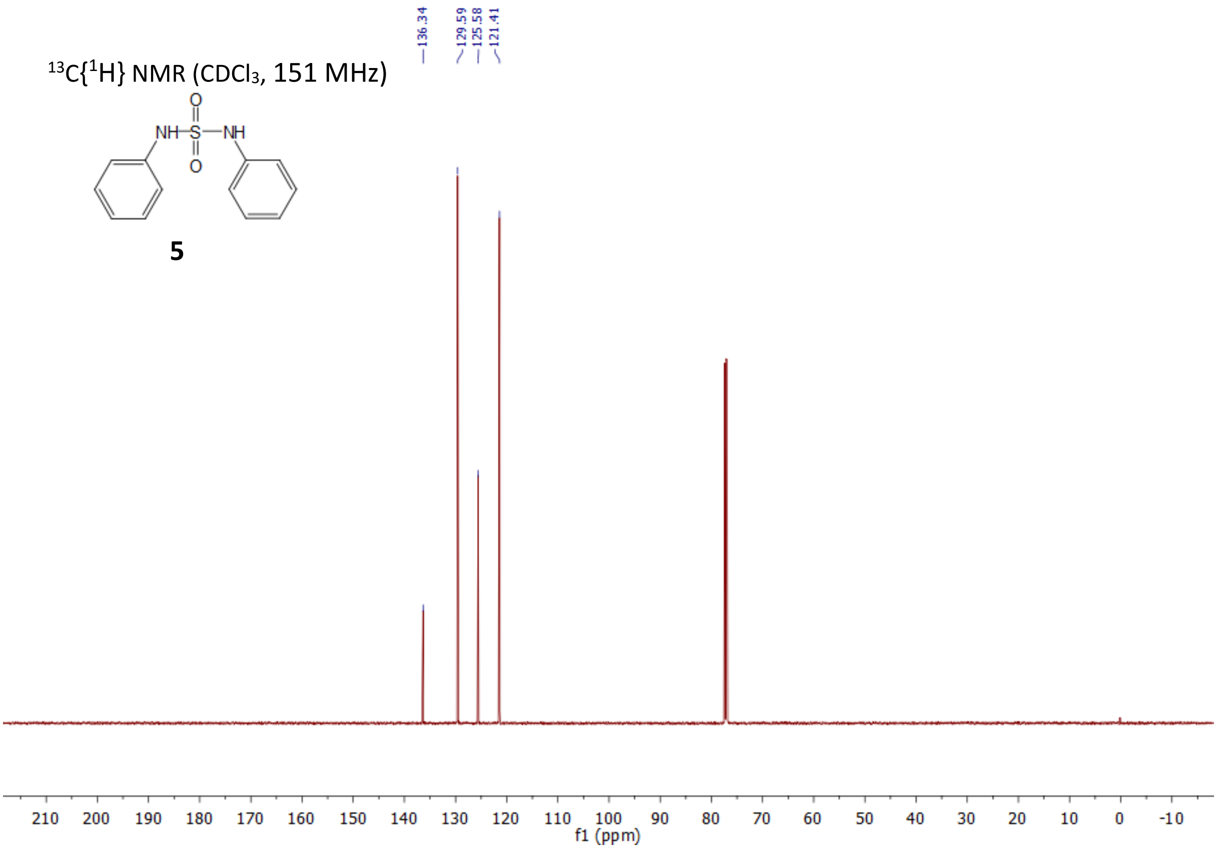

136.34
129.59
125.58
121.41
¹³C{¹H} NMR (CDCl₃, 151 MHz)
NH
S
NH
O
O
5
f1 (ppm)

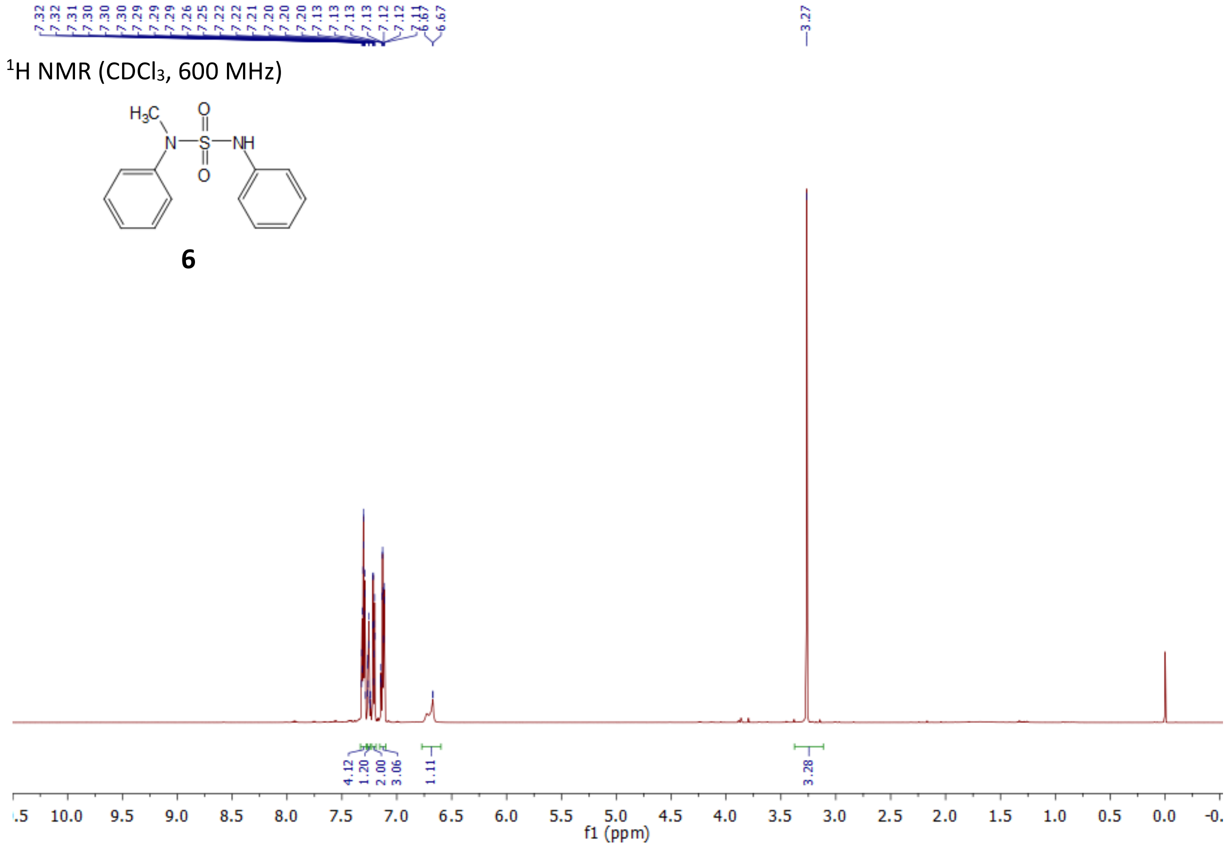
1H NMR (CDCl3, 600 MHz)
H3C
N
S
O
O
NH
6
7.32 7.32 7.31 7.30 7.30 7.30 7.29 7.29 7.29 7.26 7.25 7.22 7.22 7.21 7.20 7.20 7.20 7.13 7.13 7.13 7.13 7.12 7.12 7.11 6.67 6.67
3.27
4.12 1.20 2.00 3.06
1.11
3.28
f1 (ppm)

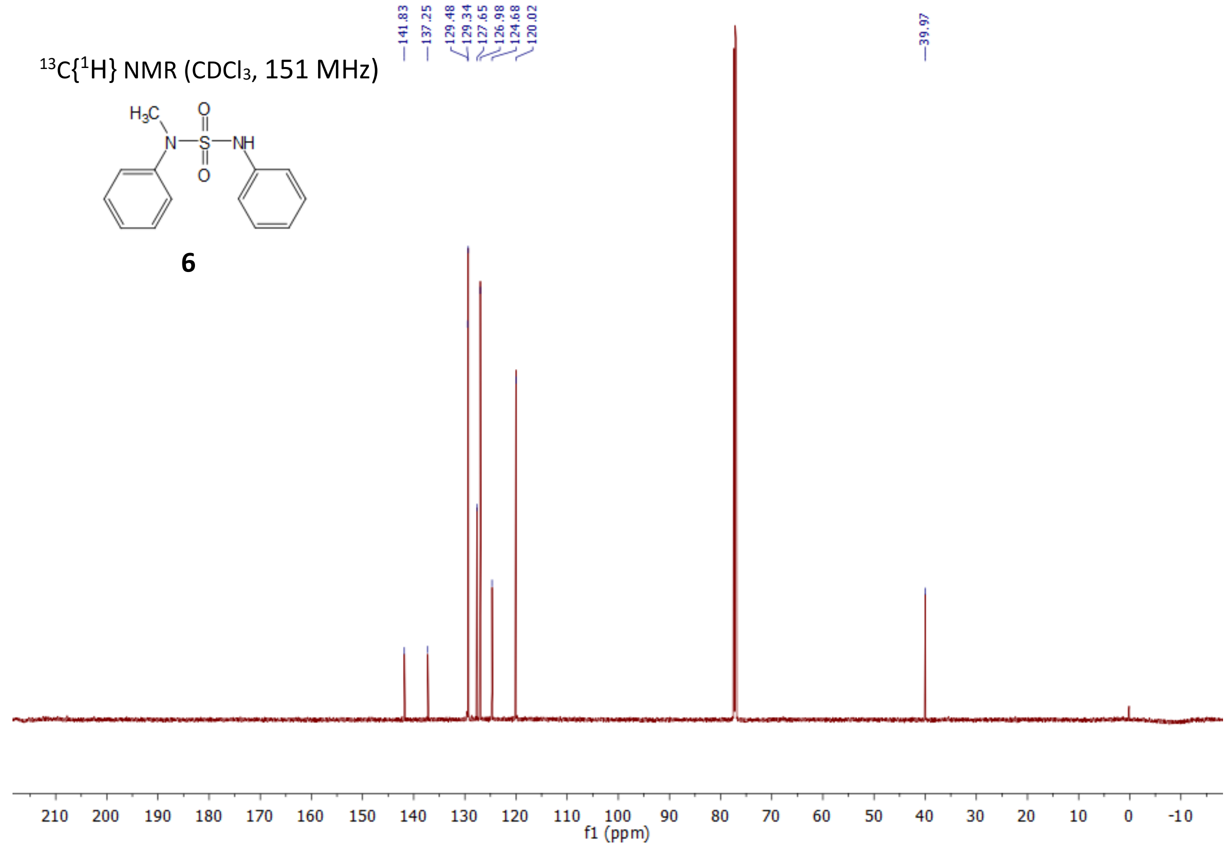
13C{1H} NMR (CDCl3, 151 MHz)
H3C
N
S
O
O
NH
6
141.83
137.25
129.48 129.34 127.65 126.98 124.68 120.02
39.97
f1 (ppm)

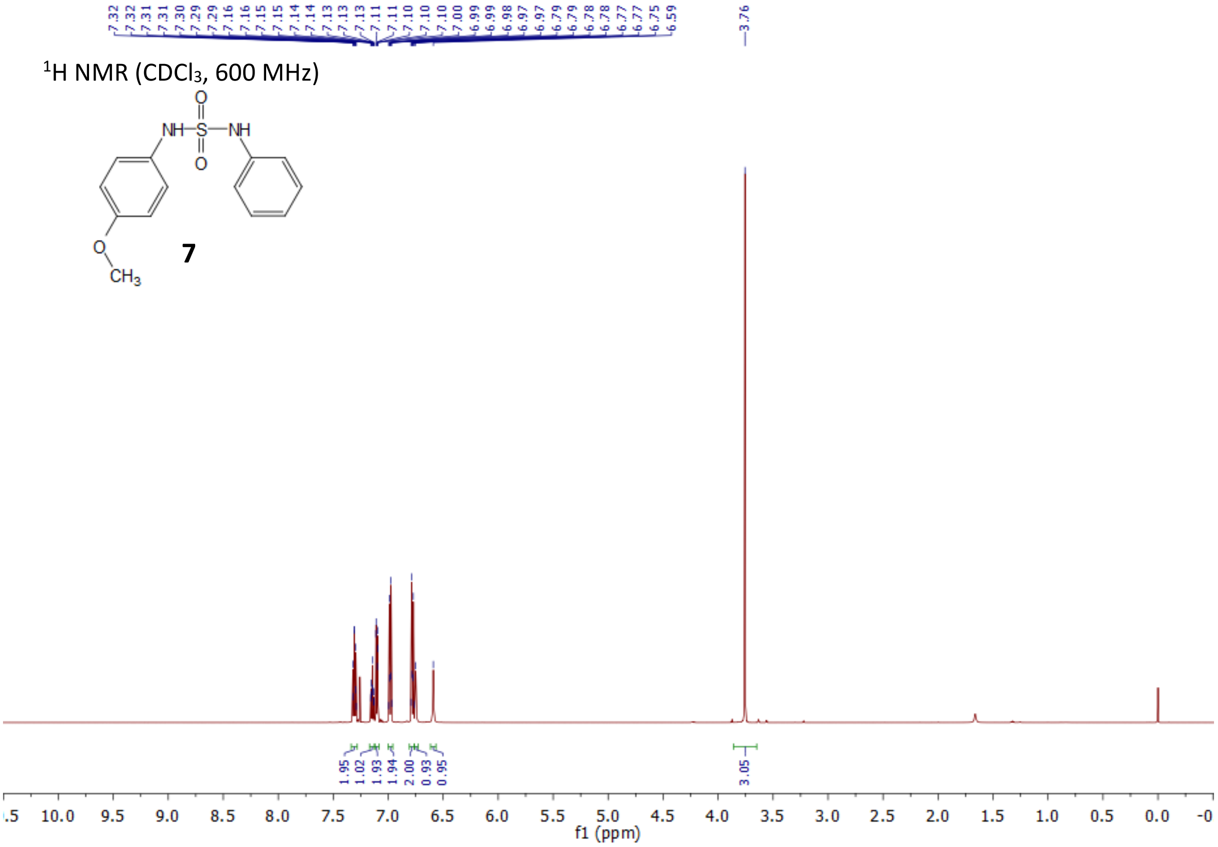

^{1}H NMR ($CDCl_3$, 600 MHz)
7
7.32, 7.32, 7.31, 7.31, 7.30, 7.29, 7.29, 7.16, 7.16, 7.15, 7.15, 7.14, 7.14, 7.13, 7.13, 7.13, 7.11, 7.11, 7.10, 7.10, 7.00, 6.99, 6.99, 6.98, 6.97, 6.97, 6.79, 6.79, 6.79, 6.78, 6.77, 6.77, 6.75, 6.59
3.76
1.95, 1.02, 1.93, 1.94, 2.00, 0.93, 0.95
3.05
f1 (ppm)

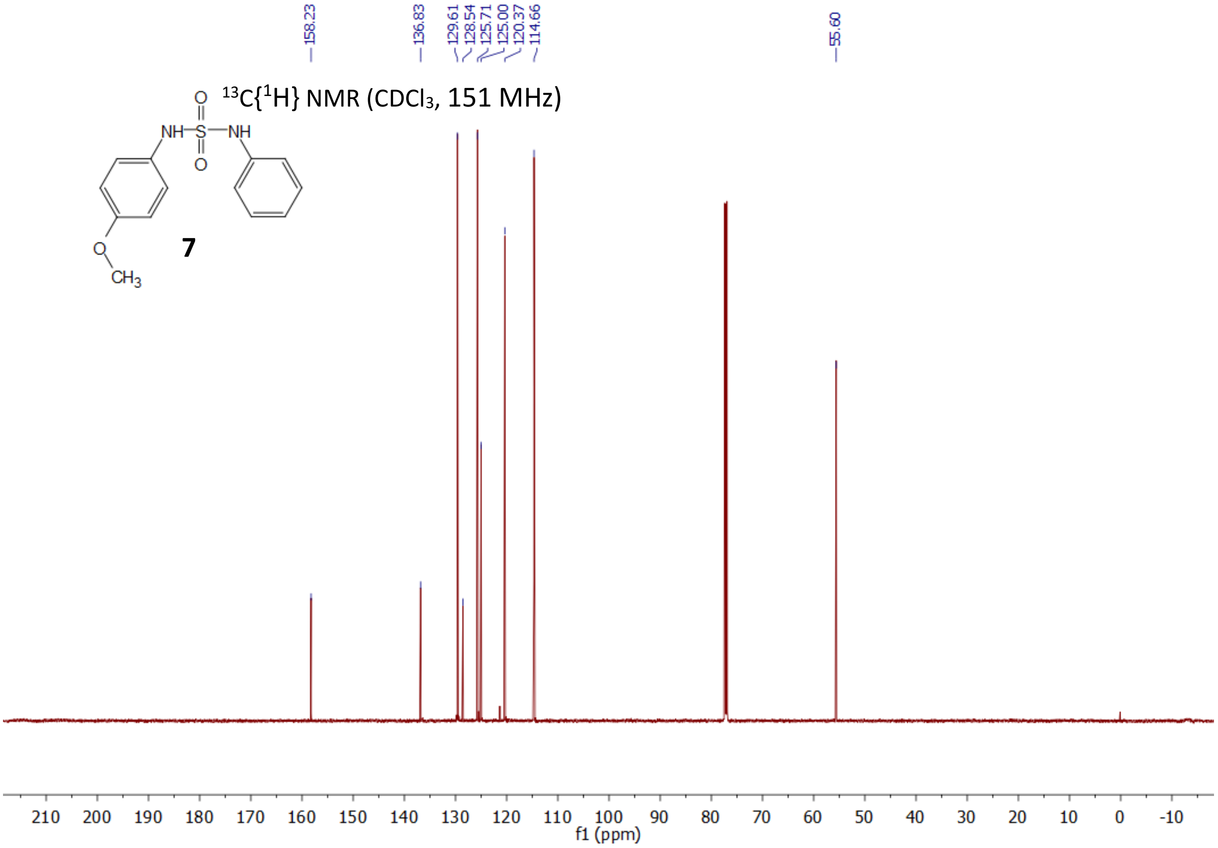

^{13}C{^{1}H} NMR ($CDCl_3$, 151 MHz)
7
158.23
136.83
129.61, 128.54, 125.71, 125.00, 120.37, 114.66
55.60
f1 (ppm)

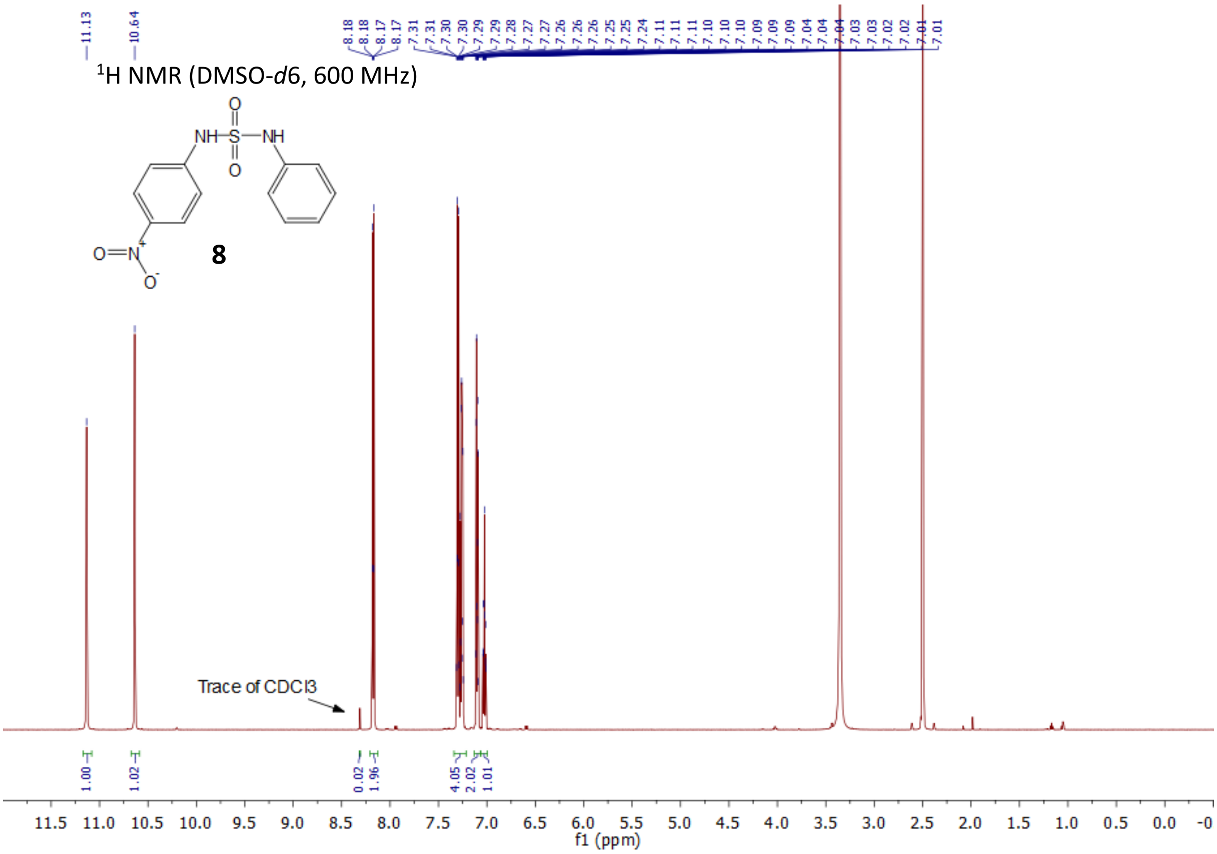

^{1}H NMR (DMSO-$d6$, 600 MHz)
11.13
10.64
8.18 8.18 8.17 8.17 7.31 7.31 7.30 7.30 7.29 7.29 7.28 7.27 7.27 7.26 7.26 7.25 7.25 7.24 7.11 7.11 7.11 7.10 7.10 7.09 7.09 7.09 7.04 7.04 7.04 7.03 7.03 7.02 7.02 7.01 7.01
NH
S
NH
O
O
O=N
O
8
Trace of CDCl3
1.00
1.02
0.02
1.96
4.05
2.02
1.01
f1 (ppm)

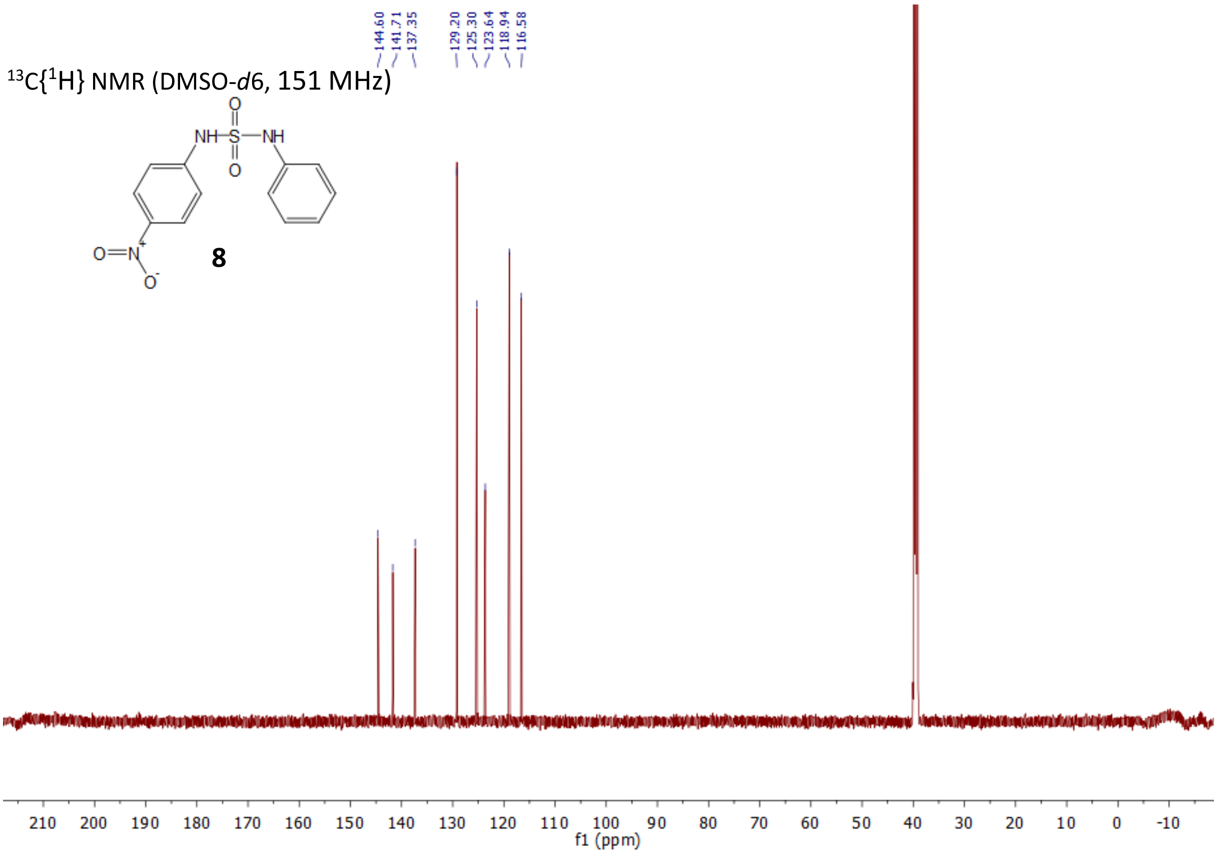

^{13}C{^{1}H} NMR (DMSO-$d6$, 151 MHz)
144.60
141.71
137.35
129.20
125.30
123.64
118.94
116.58
NH
S
NH
O
O
O=N
O
8
f1 (ppm)

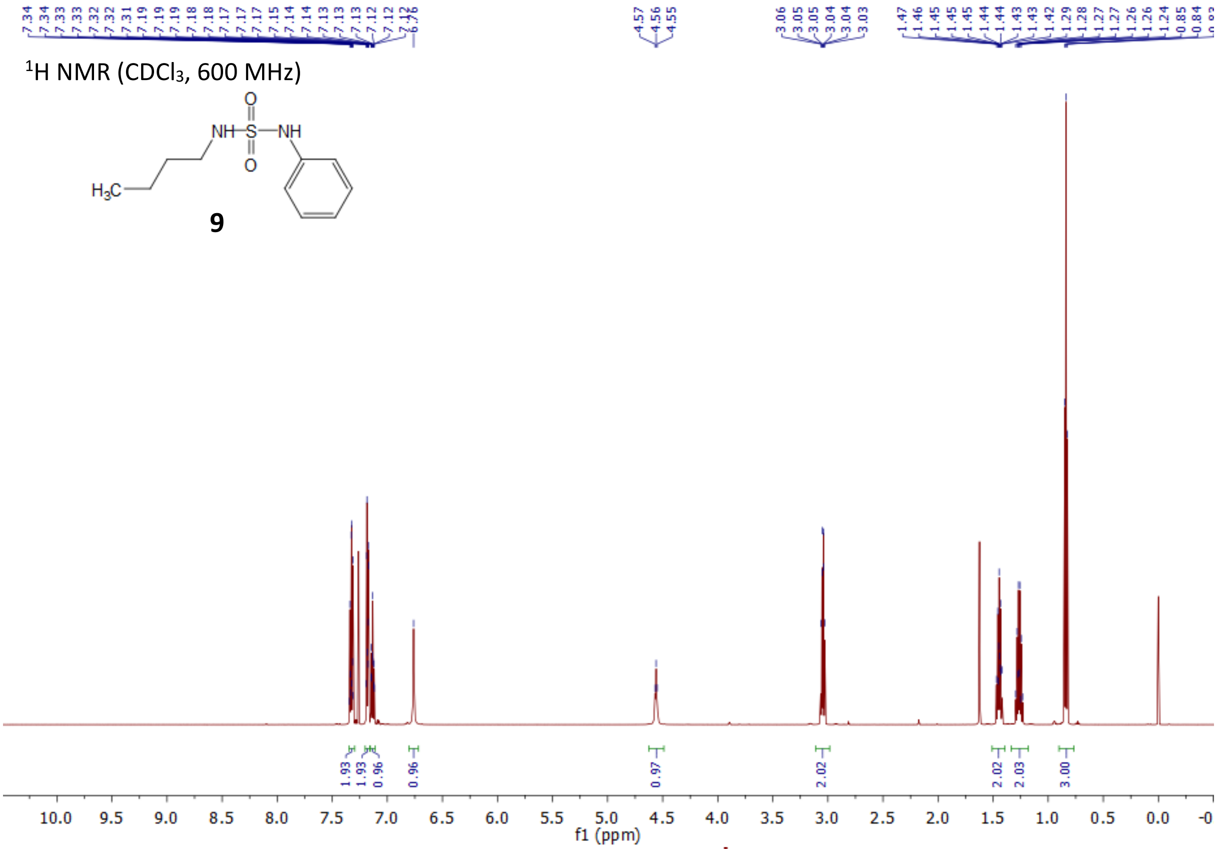

7.34
7.34
7.33
7.33
7.32
7.32
7.31
7.19
7.19
7.19
7.18
7.18
7.17
7.17
7.17
7.15
7.14
7.14
7.13
7.13
7.13
7.12
7.12
6.76
4.57
4.56
4.55
3.06
3.05
3.05
3.04
3.04
3.03
1.47
1.46
1.45
1.45
1.45
1.44
1.44
1.43
1.43
1.42
1.29
1.28
1.27
1.27
1.26
1.26
1.24
0.85
0.84
1H NMR (CDCl3, 600 MHz)
O
NH
S
NH
O
H3C
9
1.93
1.93
0.96
0.96
0.97
2.02
2.02
2.03
3.00
10.0
9.5
9.0
8.5
8.0
7.5
7.0
6.5
6.0
5.5
5.0
4.5
4.0
3.5
3.0
2.5
2.0
1.5
1.0
0.5
0.0
f1 (ppm)

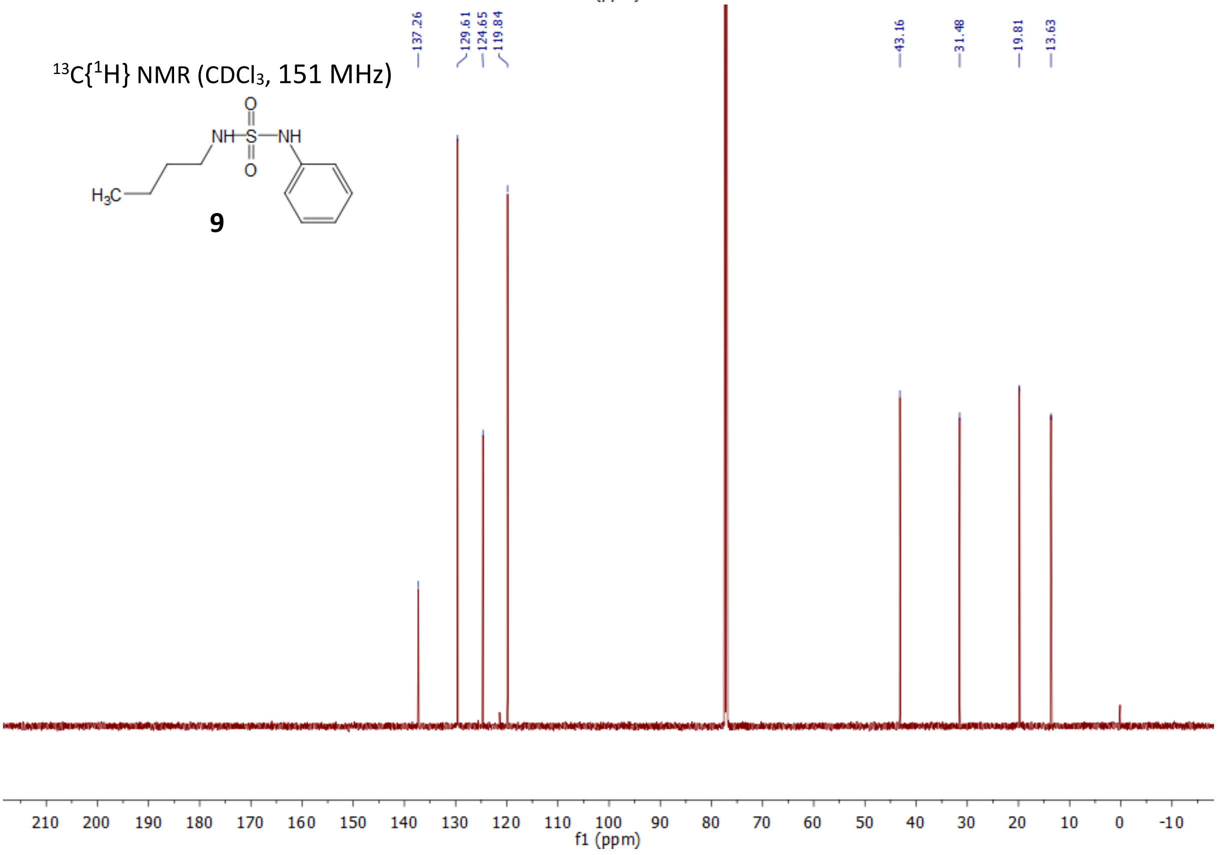

137.26
129.61
124.65
119.84
43.16
31.48
19.81
13.63
13C{1H} NMR (CDCl3, 151 MHz)
O
NH
S
NH
O
H3C
9
210
200
190
180
170
160
150
140
130
120
110
100
90
80
70
60
50
40
30
20
10
0
-10
f1 (ppm)

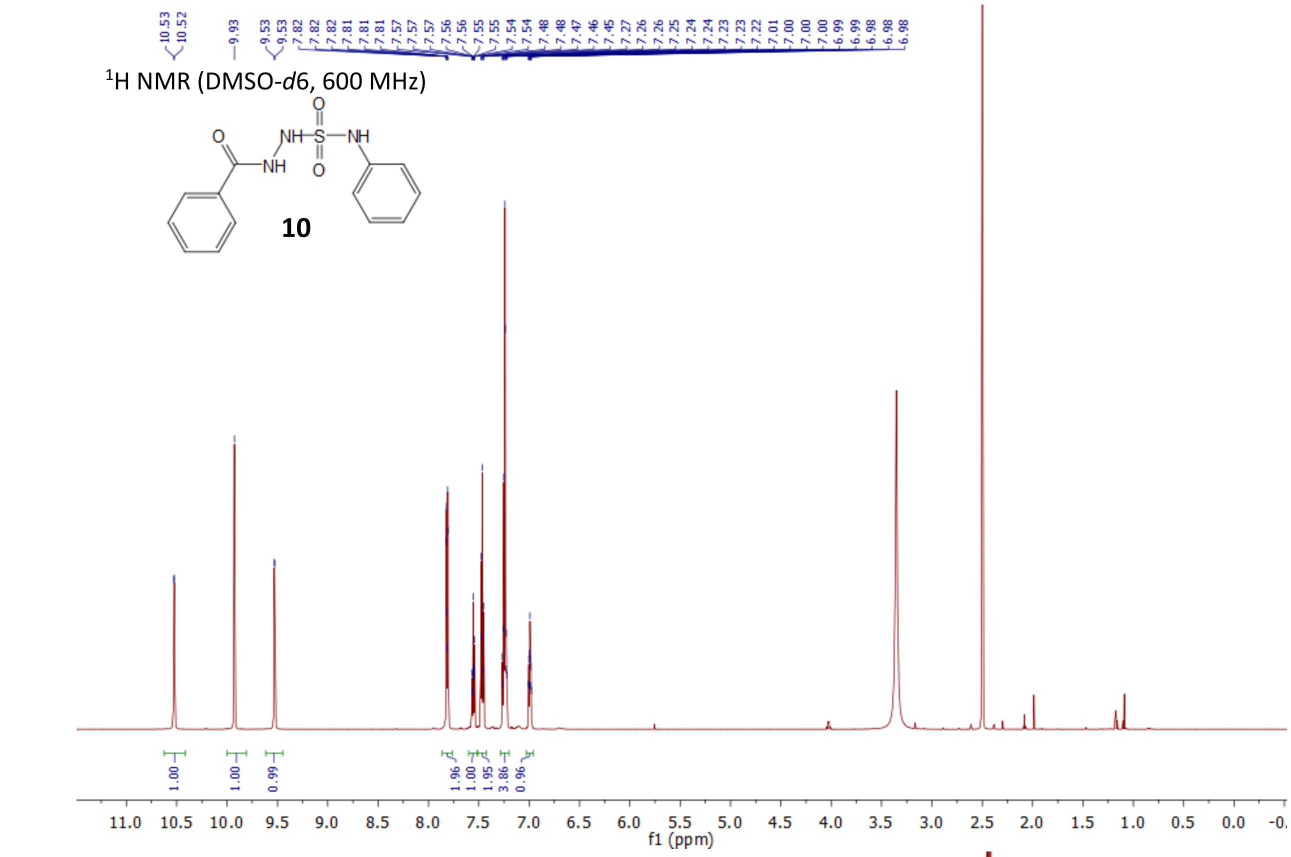

^{1}H NMR (DMSO-*d6*, 600 MHz)
10
f1 (ppm)

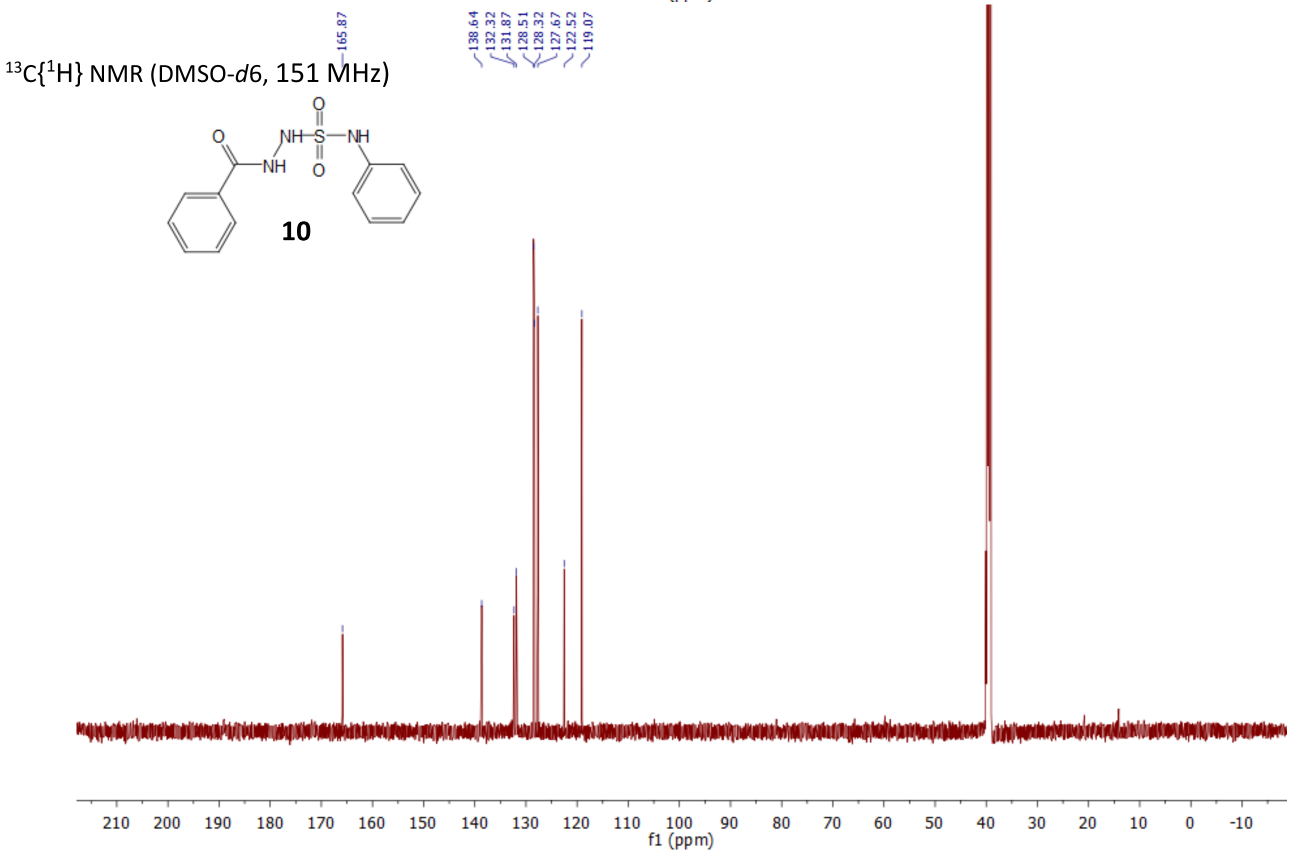

^{13}C{^{1}H} NMR (DMSO-*d6*, 151 MHz)
10
f1 (ppm)

^1H NMR ($CDCl_3$, 600 MHz)

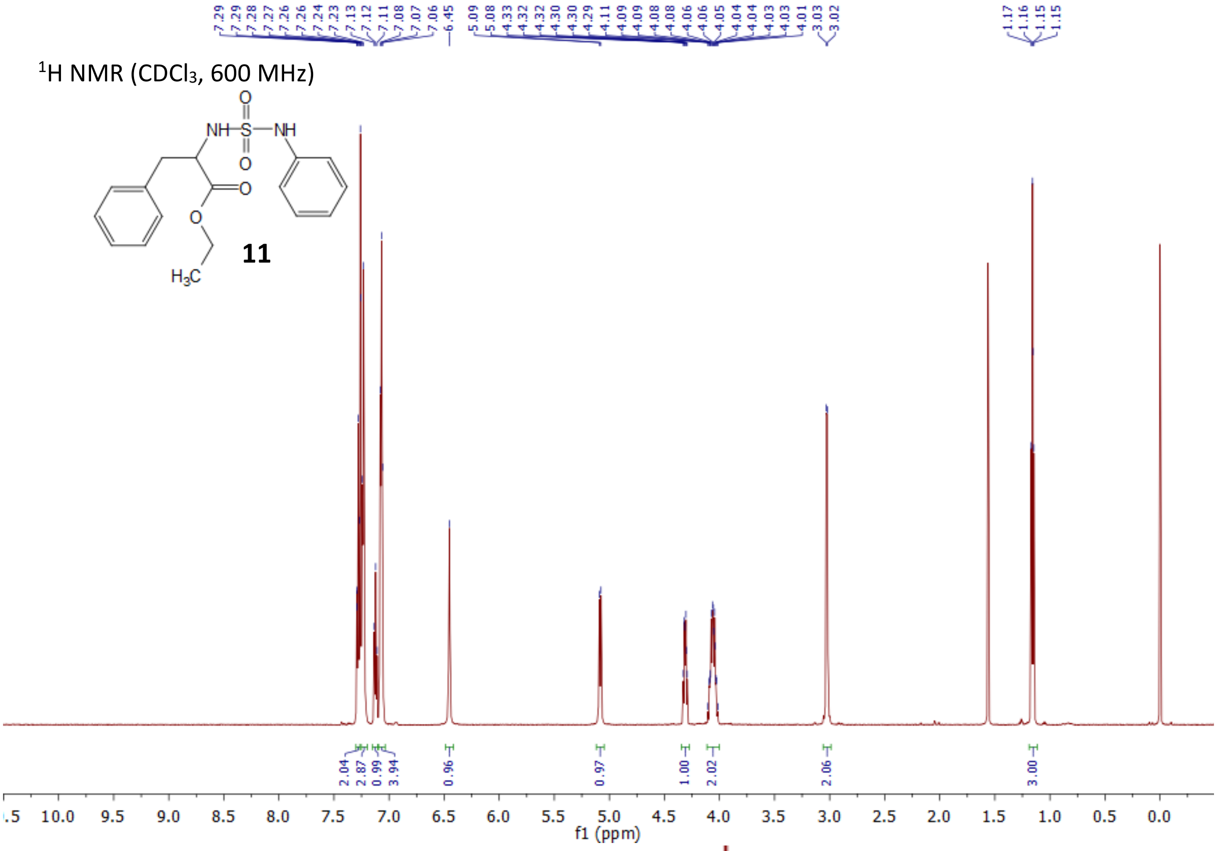

$^{13}C\{^1H\}$ NMR ($CDCl_3$, 151 MHz)

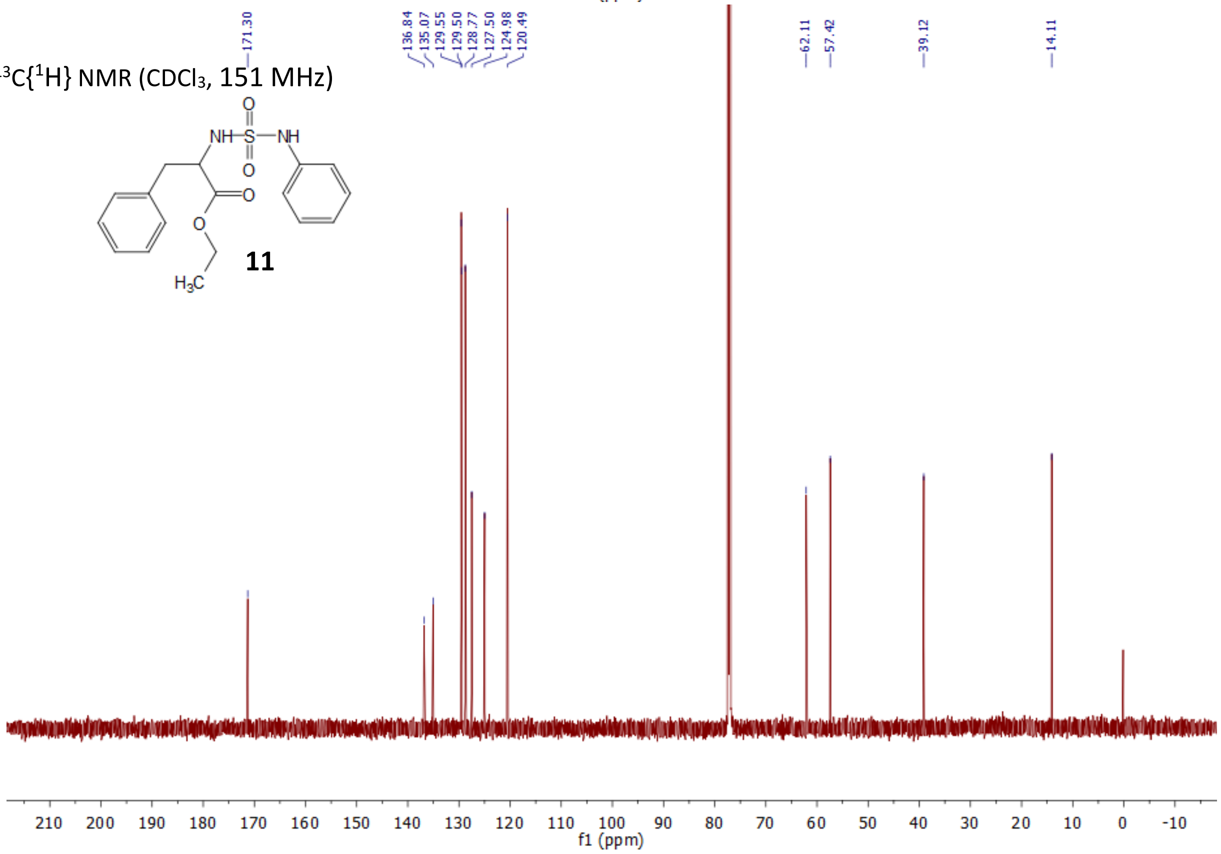

^{1}H NMR ($CDCl_3$, 600 MHz)

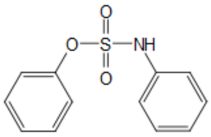

12

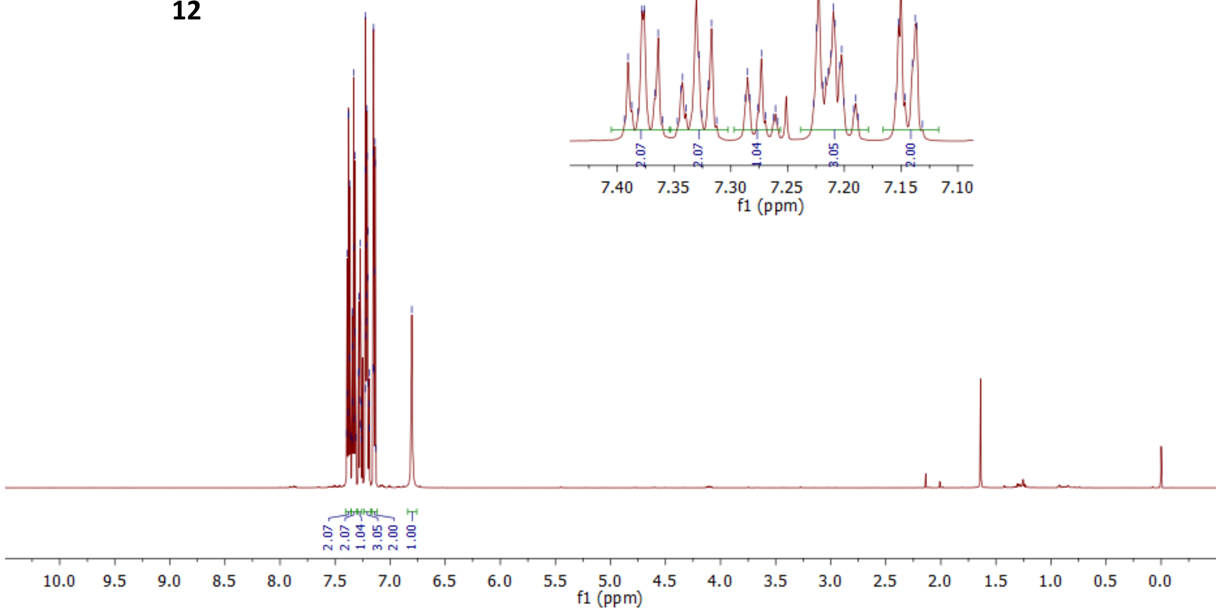

$^{13}C\{^{1}H\}$ NMR ($CDCl_3$, 151 MHz)

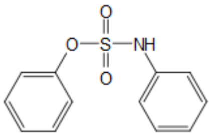

12

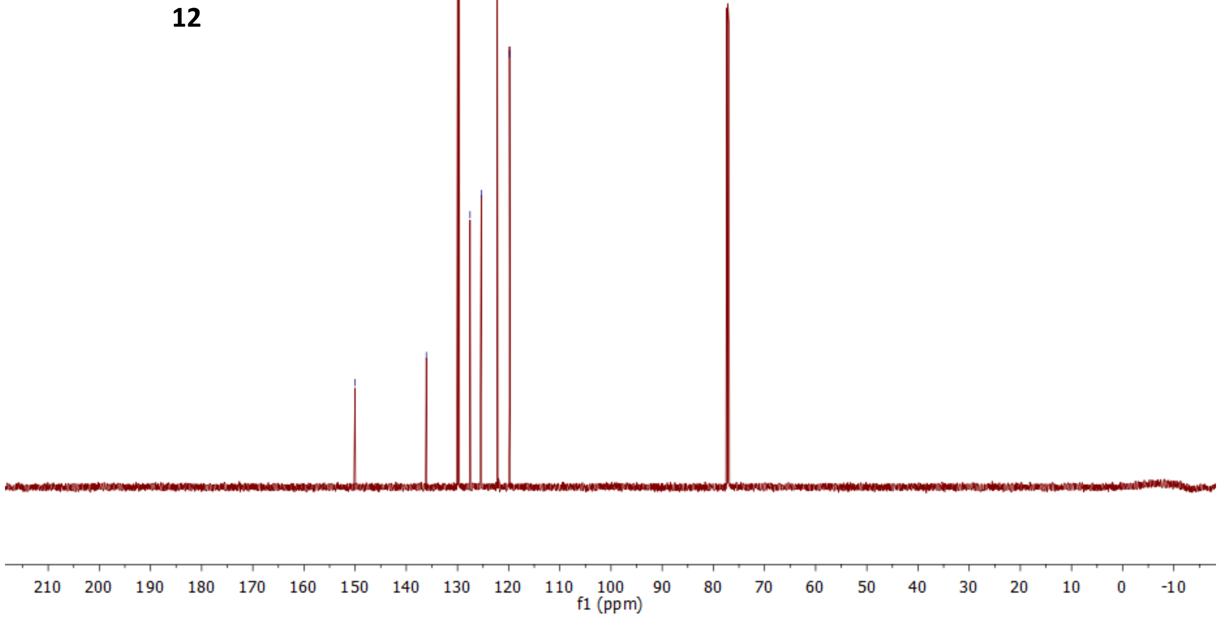

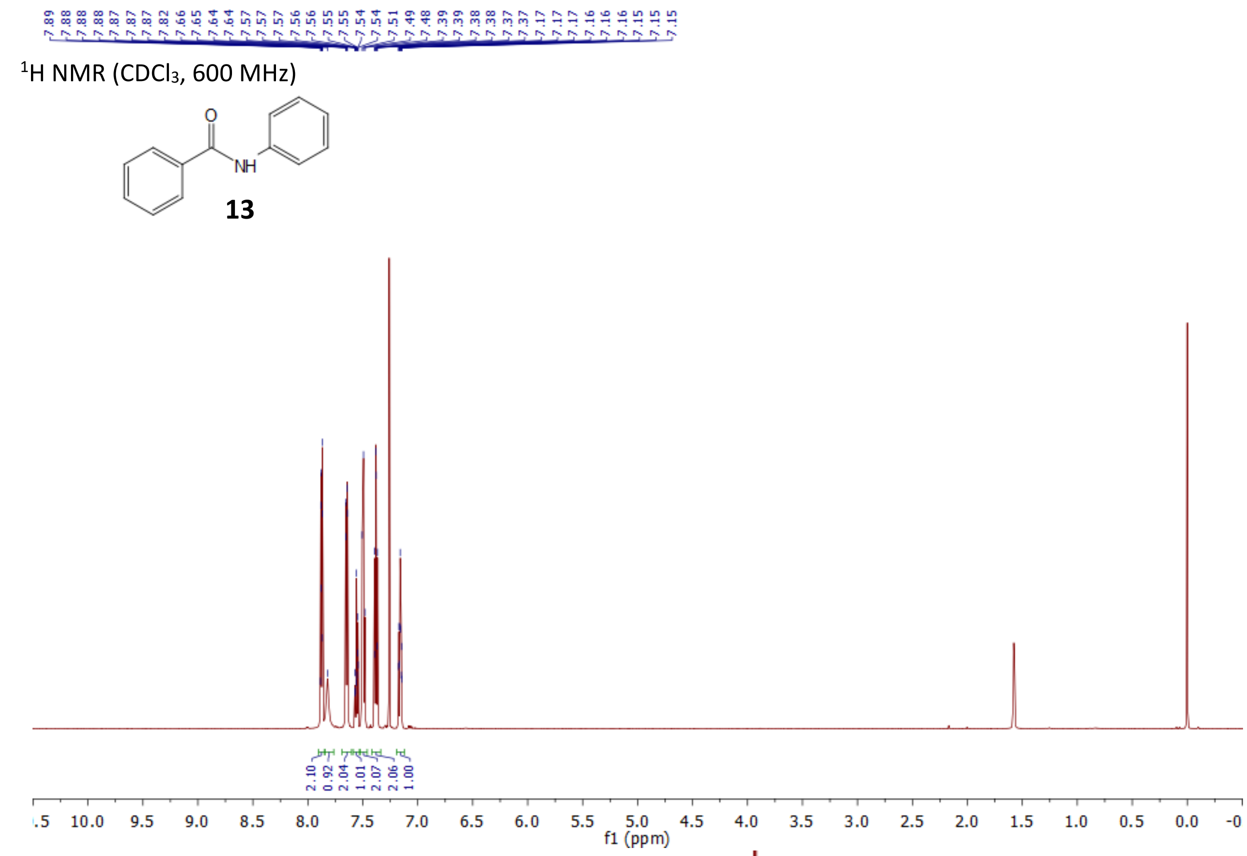
7.89 7.88 7.88 7.88 7.87 7.87 7.87 7.82 7.66 7.65 7.64 7.64 7.57 7.57 7.57 7.56 7.56 7.55 7.55 7.54 7.54 7.51 7.49 7.48 7.39 7.39 7.38 7.38 7.37 7.37 7.17 7.17 7.17 7.16 7.16 7.15 7.15 7.15
^{1}H NMR ($CDCl_3$, 600 MHz)
O
NH
13
2.10 0.92 2.04 1.01 2.07 2.06 1.00
f1 (ppm)

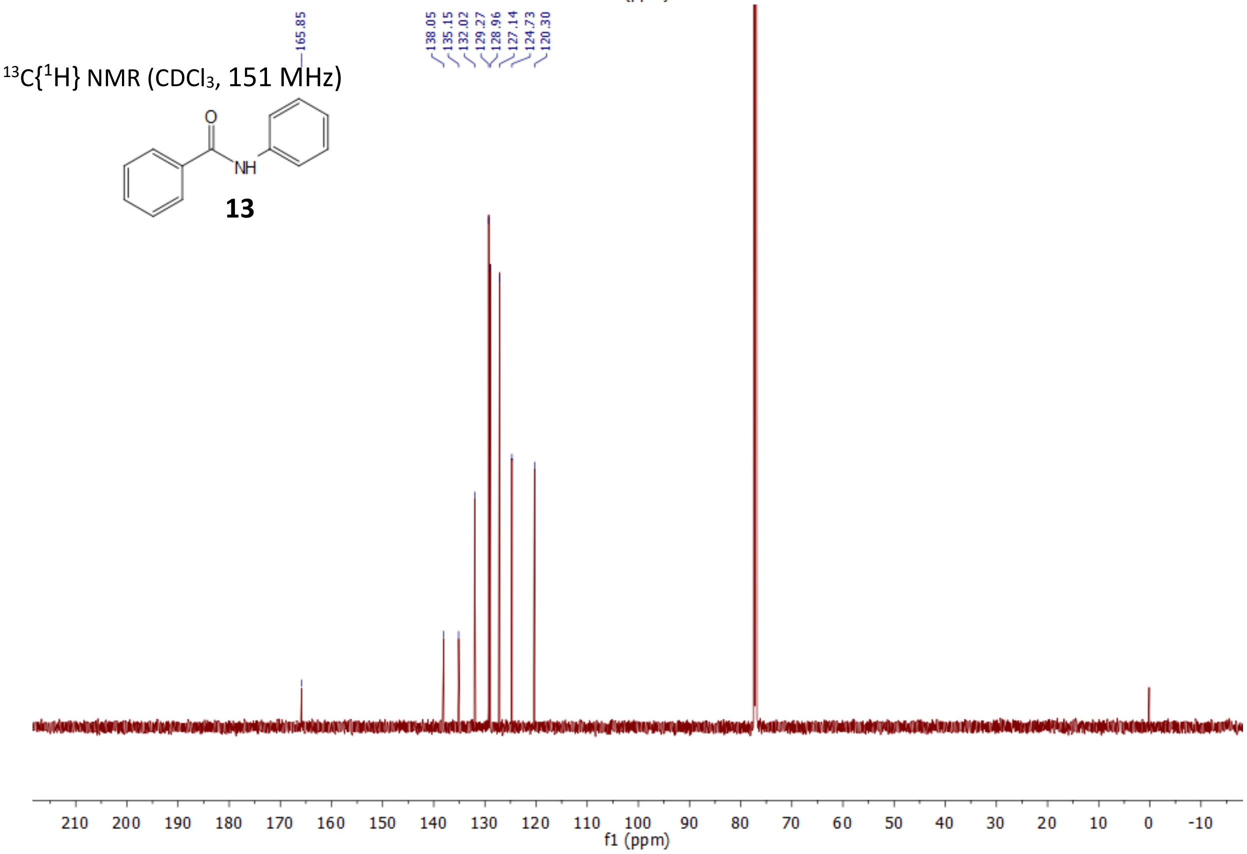
165.85
138.05 135.15 132.02 129.27 128.96 127.14 124.73 120.30
^{13}C{^{1}H} NMR ($CDCl_3$, 151 MHz)
O
NH
13
f1 (ppm)

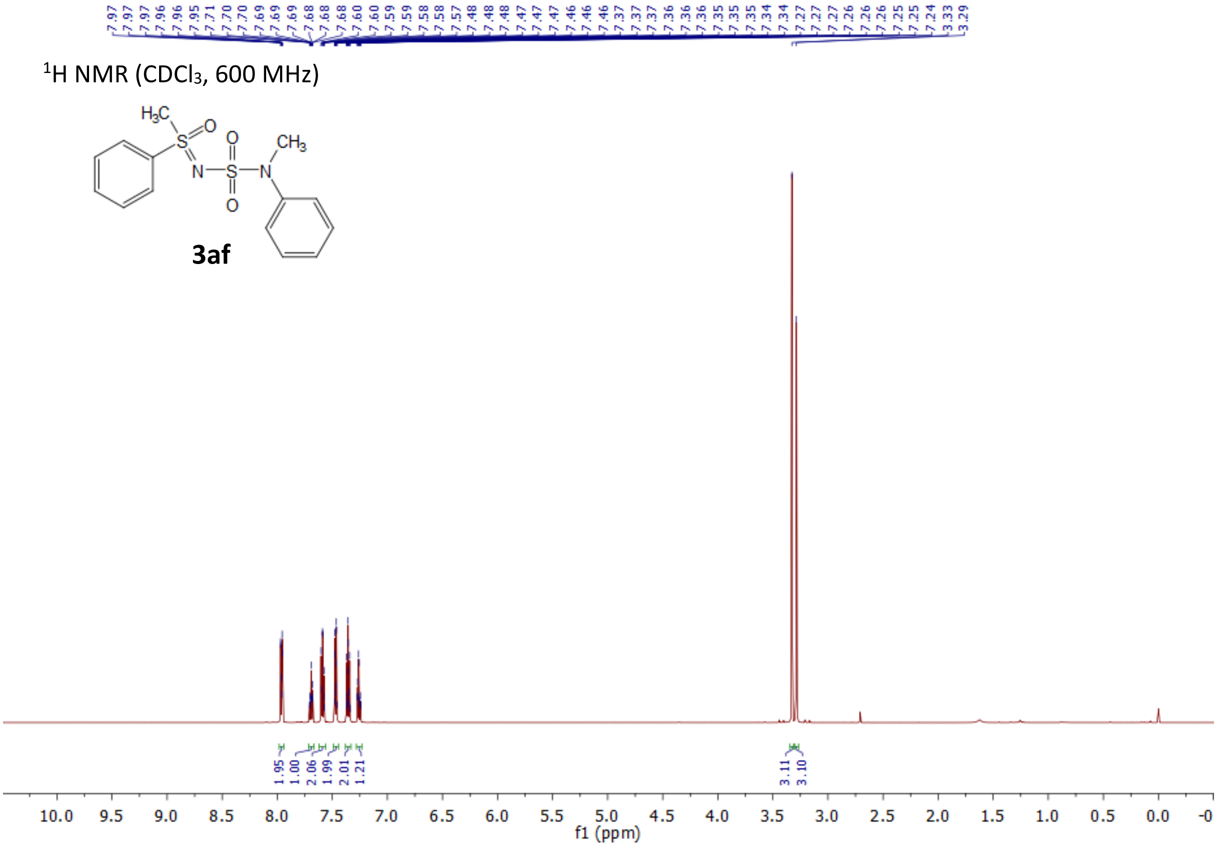

^{1}H NMR ($CDCl_3$, 600 MHz)
3af

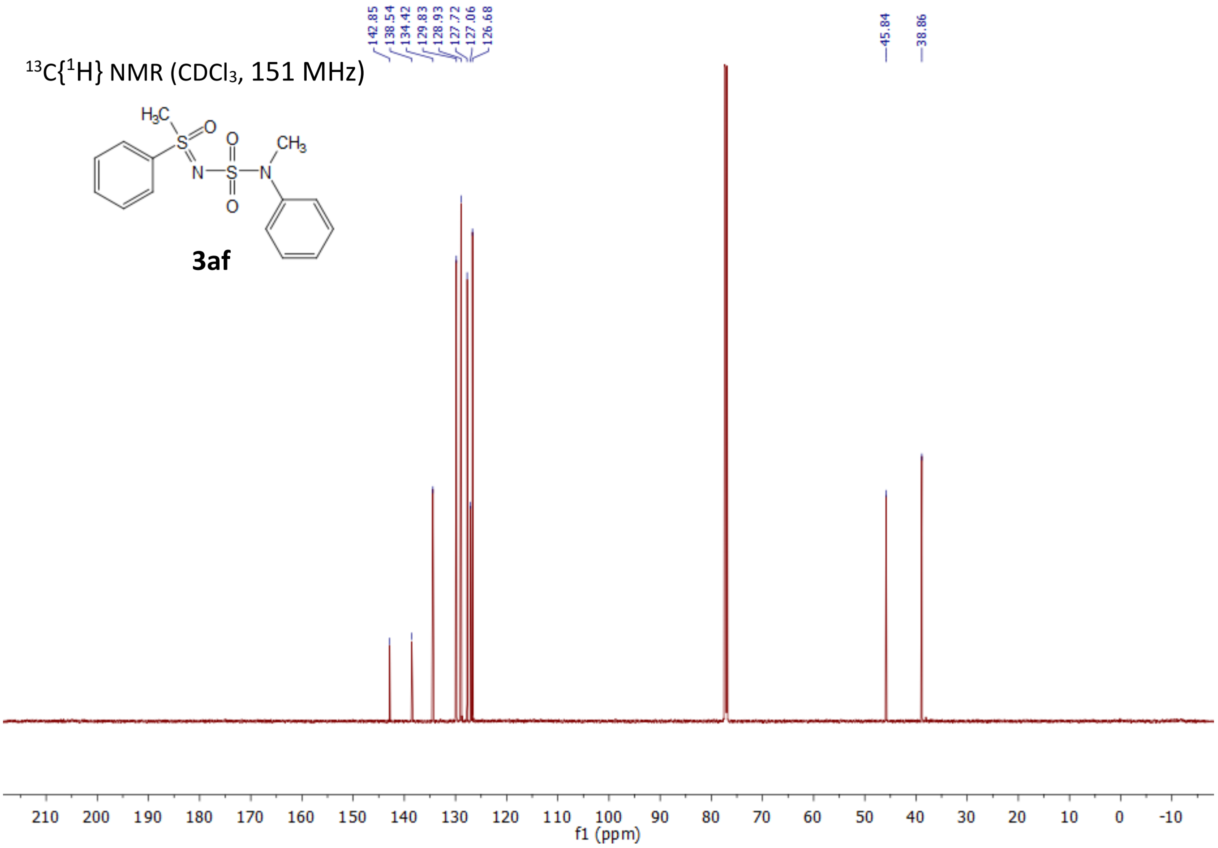

^{13}C{^{1}H} NMR ($CDCl_3$, 151 MHz)
3af

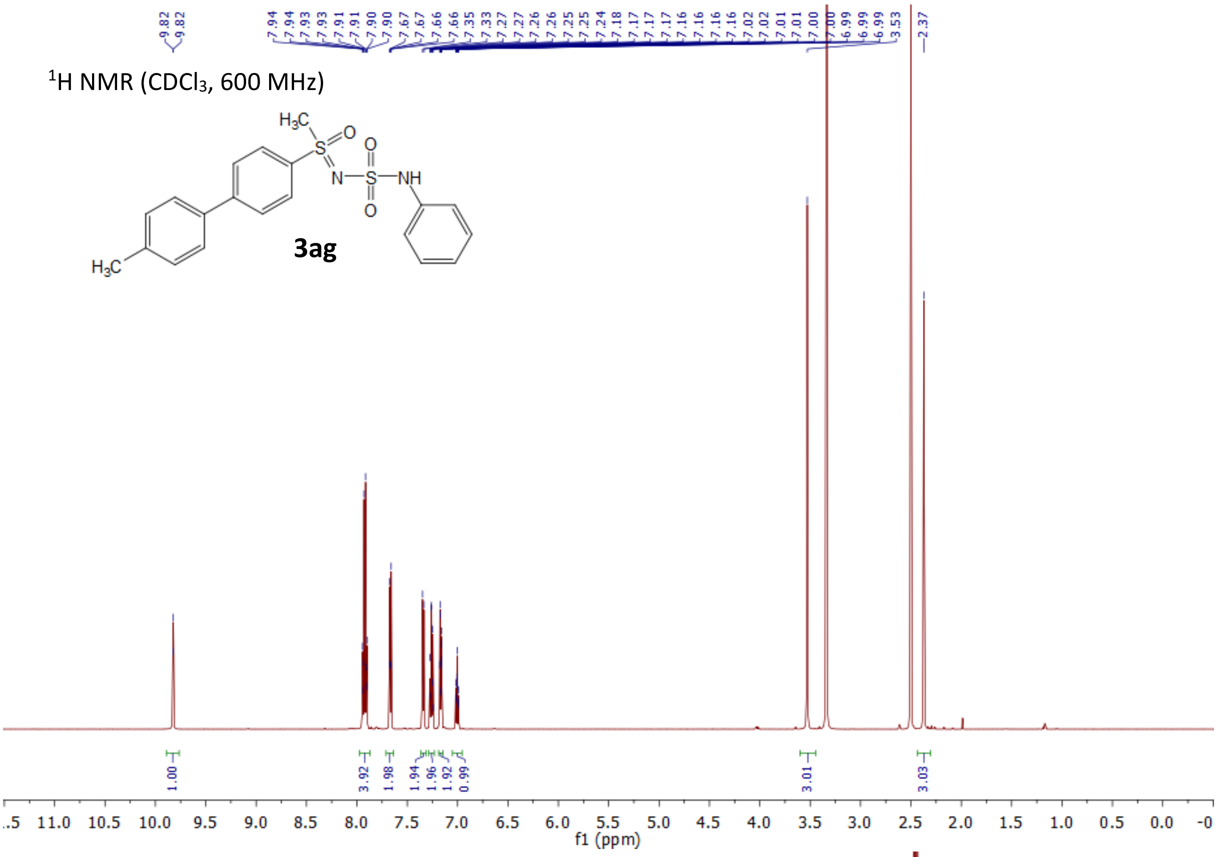
1H NMR (CDCl3, 600 MHz)
3ag
H3C
O
N
S
NH
9.82 9.82
7.94 7.94 7.93 7.93 7.91 7.91 7.90 7.90 7.67 7.67 7.66 7.66 7.35 7.33 7.27 7.27 7.26 7.26 7.25 7.25 7.24 7.18 7.17 7.17 7.17 7.16 7.16 7.16 7.02 7.02 7.01 7.01 7.00 7.00 6.99 6.99 6.99 3.53
2.37
1.00
3.92
1.98
1.94
1.96
1.92
0.99
3.01
3.03
f1 (ppm)

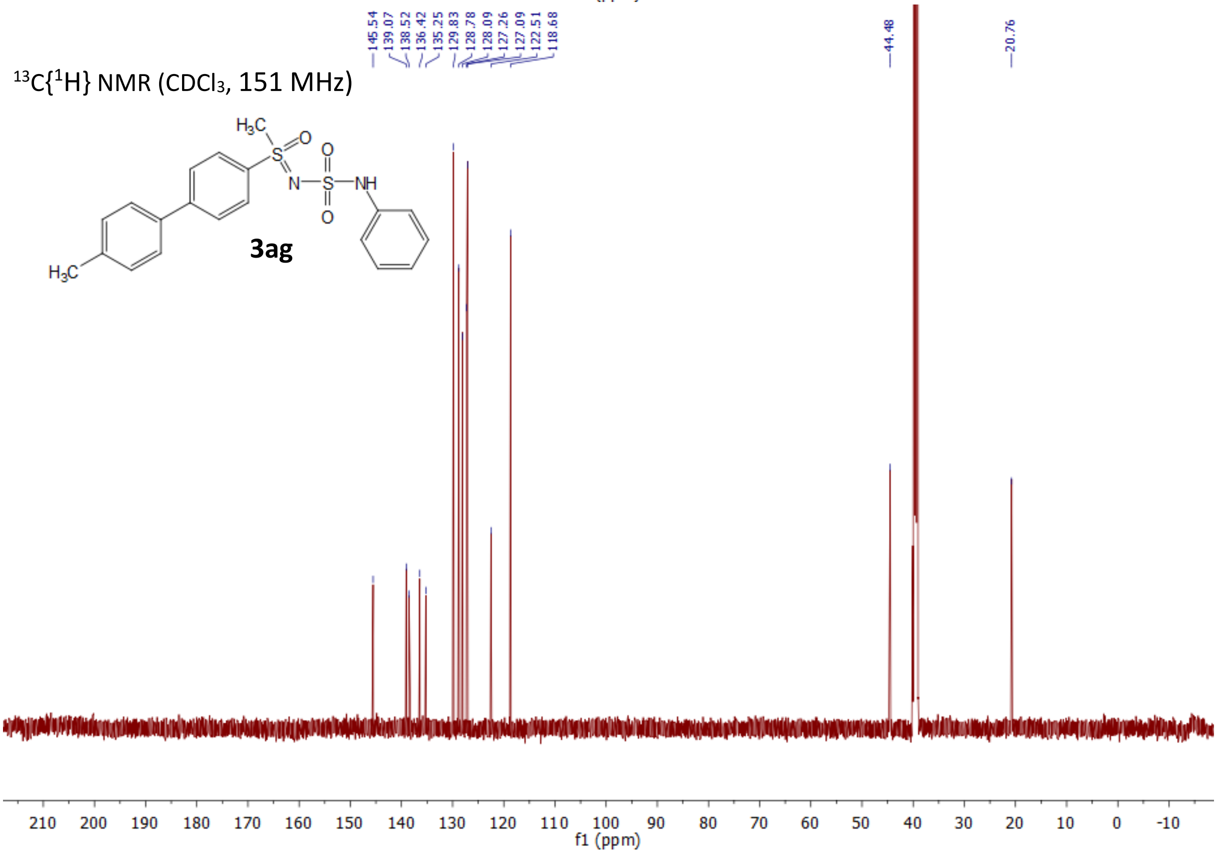
13C{1H} NMR (CDCl3, 151 MHz)
3ag
H3C
O
N
S
NH
145.54
139.07
138.52
136.42
135.25
129.83
128.78
128.09
127.26
127.09
122.51
118.68
44.48
20.76
f1 (ppm)

^1H NMR ($CDCl_3$, 600 MHz)

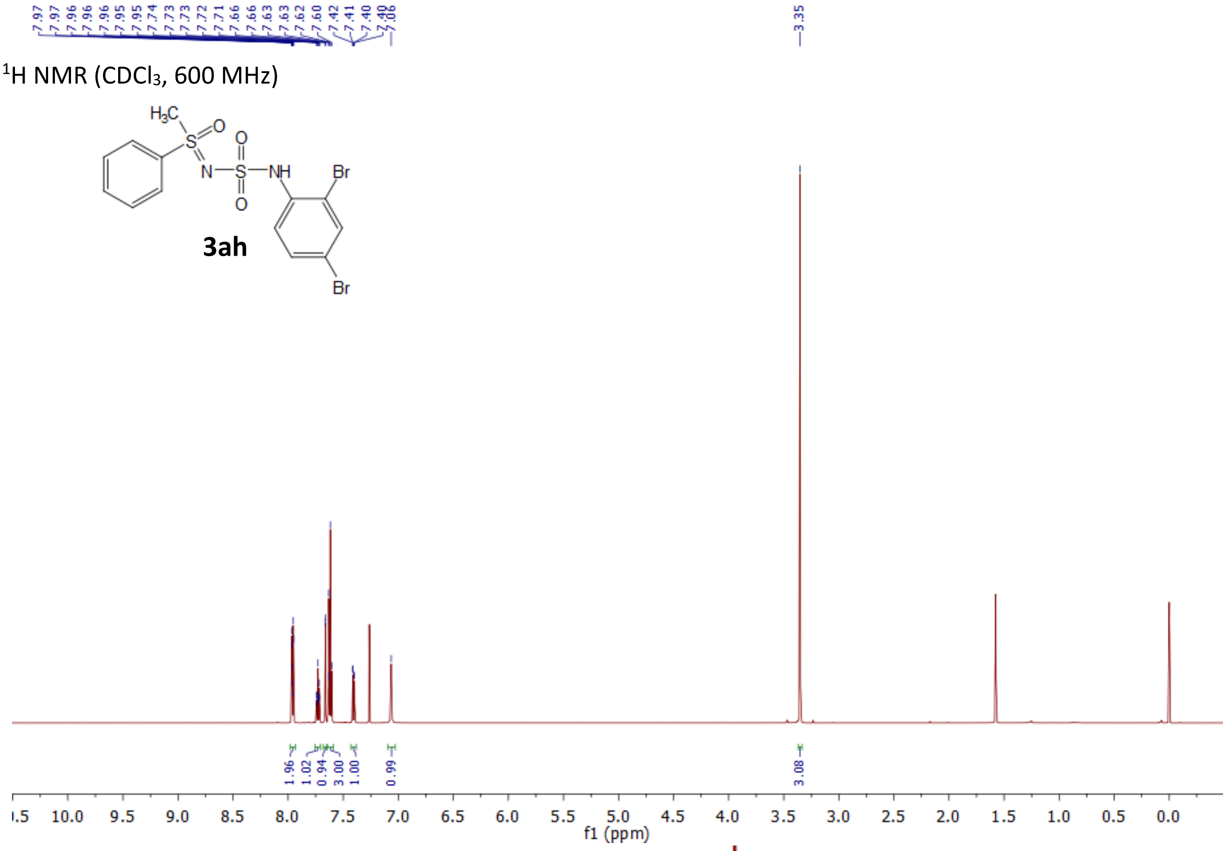

$^{13}C\{^1H\}$ NMR ($CDCl_3$, 151 MHz)

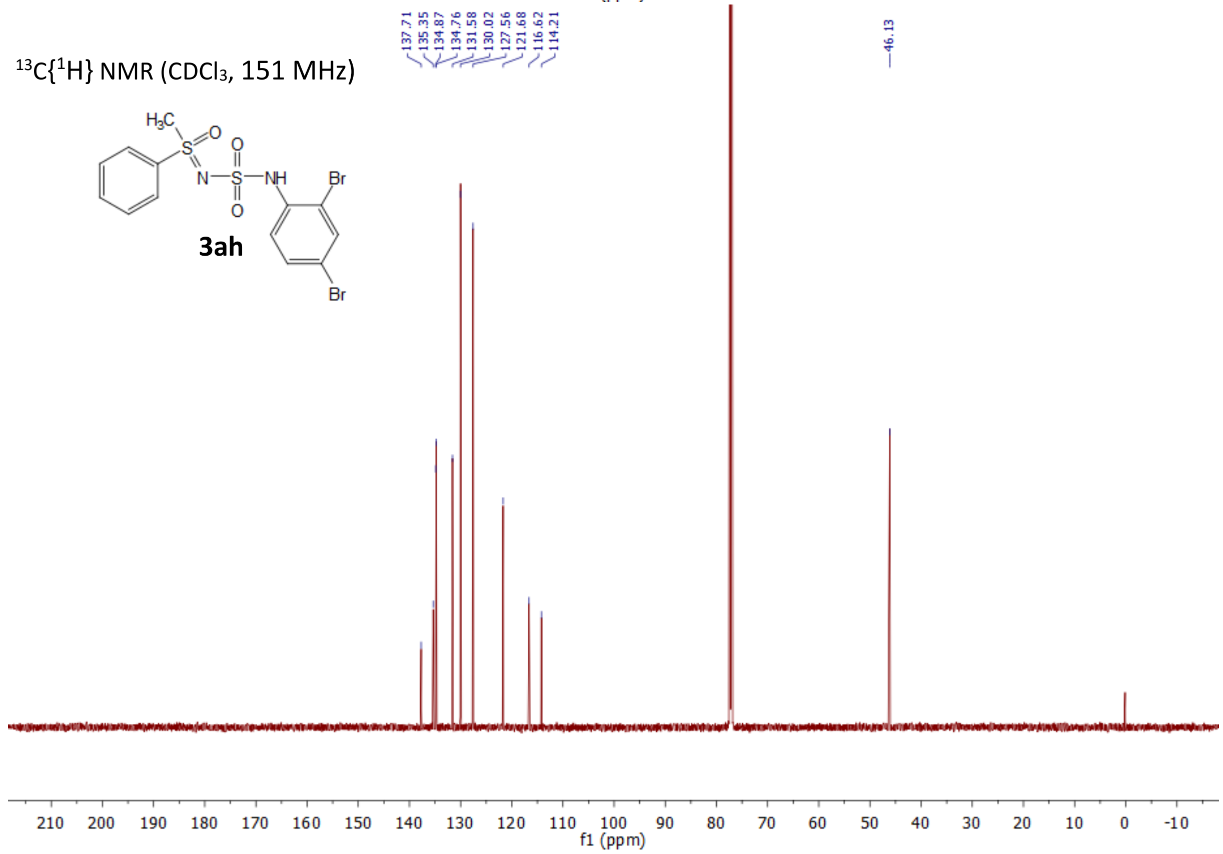

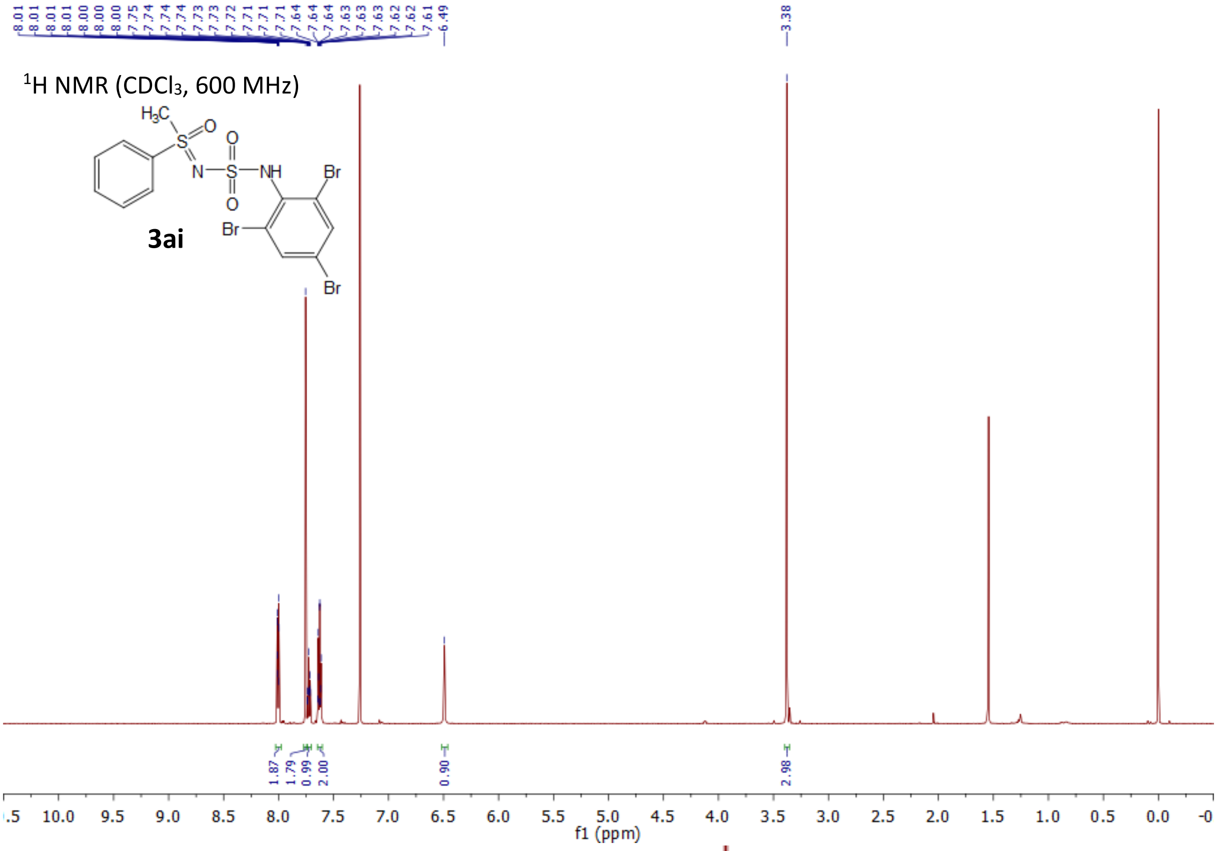
8.01
8.01
8.01
8.01
8.00
8.00
8.00
7.75
7.74
7.74
7.74
7.73
7.73
7.72
7.71
7.71
7.64
7.64
7.64
7.63
7.63
7.63
7.62
7.62
7.61
6.49
3.38
^{1}H NMR ($CDCl_3$, 600 MHz)
H_3C
S
O
N
S
NH
O
O
Br
Br
Br
3ai
1.87
1.79
0.99
2.00
0.90
2.98
f1 (ppm)

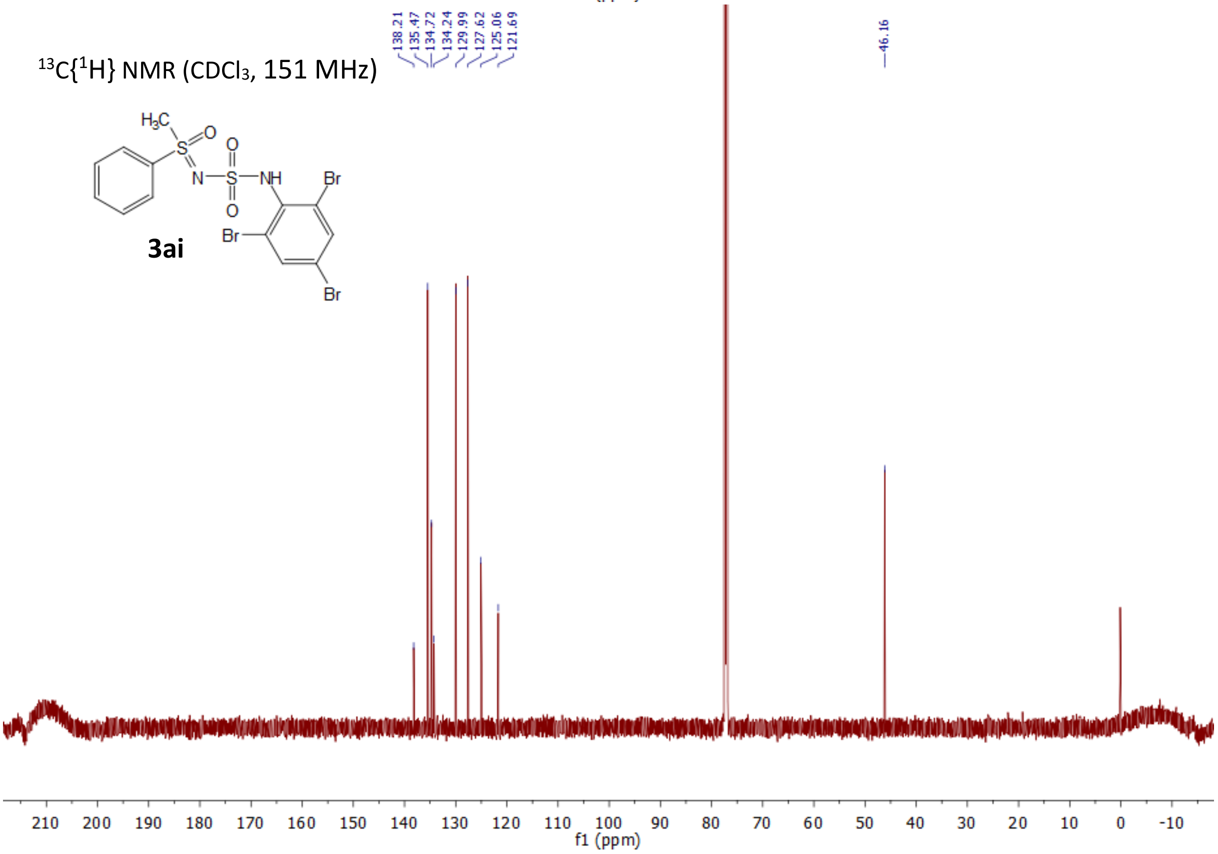
138.21
135.47
134.72
134.24
129.99
127.62
125.06
121.69
46.16
$^{13}C\{^{1}H\}$ NMR ($CDCl_3$, 151 MHz)
H_3C
S
O
N
S
NH
O
O
Br
Br
Br
3ai
f1 (ppm)

Computational details

All DFT calculations were performed with ORCA 6.0.[8] Structures and energies were computed at the wB97X-D4/def2-TZVP level of theory. The wB97X-D4/def2-TZVP level of theory is a robust choice for calculating reaction barriers because the range-separated wB97X functional accurately models the electronic structure of transition states while the D4 correction includes critical dispersion effects.[9,10] The flexible triple-zeta def2-TZVP basis set, equipped with polarization functions, provides a proper description of the orbital distortions that occur during bond breaking and formation.[11] Together, this combination ensures a balanced and physically sound treatment of the potential energy surface, leading to reliable barrier heights. Geometry optimizations were carried out without symmetry constraints and verified by analytical frequency analyses—minima exhibited no imaginary modes, while transition states located via the climbing-image nudged elastic band (CI-NEB) method[12] displayed a single imaginary frequency. Solvent effects (ethyl acetate) were included via the conductor-like PCM (C-PCM).[13] Coulomb integrals employed the RI approximation with Weigend Coulomb-fitting basis sets[14], and exchange was treated via the chain-of-spheres (COSX) algorithm[15] using default Grid 5 integration. Zero-point energy, thermal enthalpy, and entropy corrections at 298.15 K and 1 atm were applied to calculate Gibbs free energies.

Calculated energies and cartesian coordinates for different species

All structures are optimized at wB97X-D4/def2-TZVP/CPCM(ETHYLACETATE) level. Energies are in Hartrees.

M01

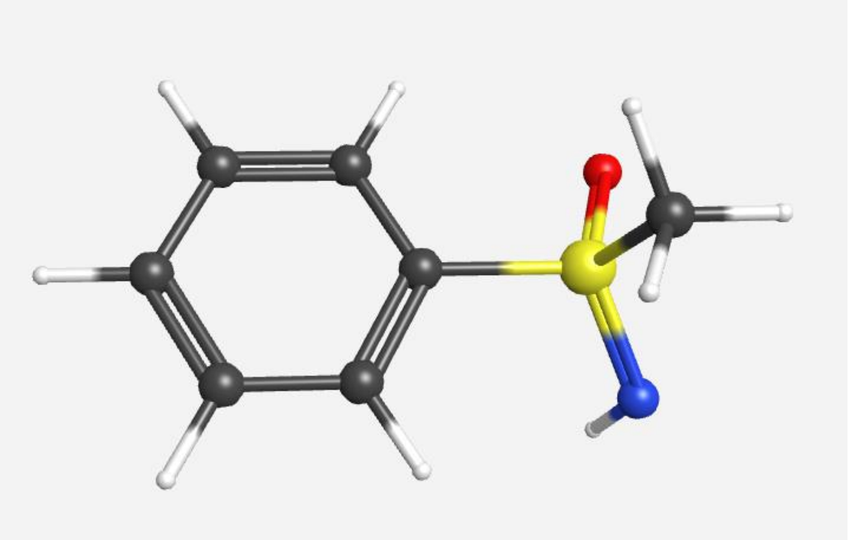

===================================

THERMODYNAMIC PROPERTIES (all values in Hartree)

===================================

Property	T = 298 K	T = 323 K
Zero Point Energy	0.151188	0.151188
Inner Energy	-800.183520	-800.182033
Enthalpy	-800.182576	-800.181010
Electronic entropy	0.000000	0.000000
Rotational entropy	0.014122	0.015430
Vibrational entropy	0.010475	0.012597
Translational entropy	0.019483	0.021323
Entropy	0.044080	0.049350
Gibbs Energy	-800.226655	-800.230361

=================================

FINAL COORDINATES (Å)

=================================

Atom	X	Y	Z
C	2.561219	-11.426019	2.632916
S	3.587855	-10.074383	2.156117
O	4.572898	-9.846269	3.203937
N	4.006916	-10.412688	0.732986
H	4.520962	-9.638144	0.318623
C	2.486371	-8.679738	2.202762
C	2.441483	-7.878269	3.336366
C	1.572908	-6.794755	3.363404
C	0.761972	-6.524853	2.266295
C	0.815235	-7.334656	1.136482
C	1.681289	-8.419175	1.100583
H	1.741579	-9.055370	0.225453
H	0.185116	-7.119213	0.280734
H	0.086465	-5.676420	2.290263
H	1.532016	-6.158946	4.240852
H	3.085848	-8.097729	4.178558
H	2.199634	-11.229448	3.641630
H	1.737343	-11.519028	1.927756
H	3.191555	-12.315767	2.614307

M02

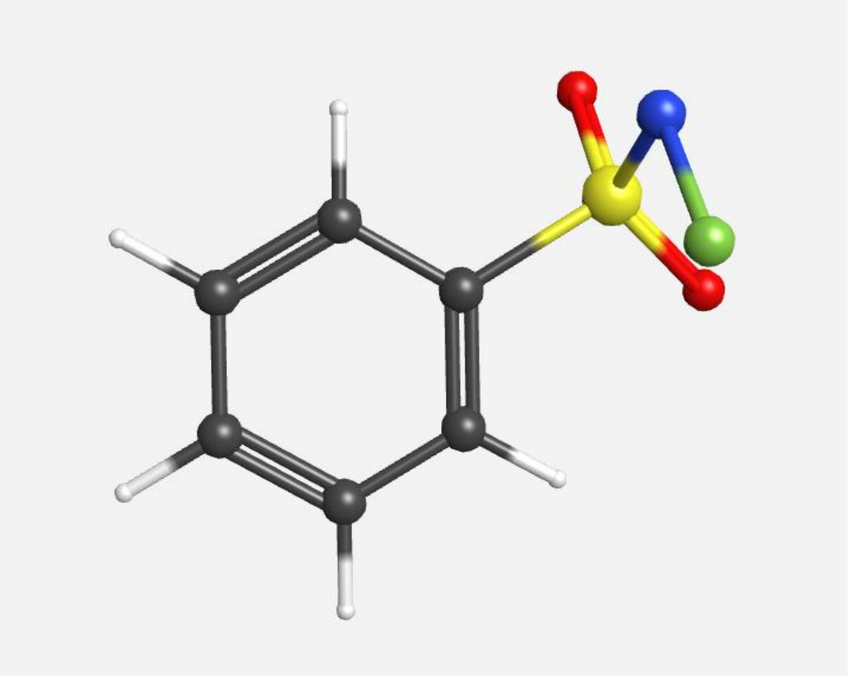

==

THERMODYNAMIC PROPERTIES (all values in Hartree)

==

Property	T = 298 K	T = 323 K
Zero Point Energy	0.107466	0.107466
Inner Energy	-934.897963	-934.896537
Enthalpy	-934.897020	-934.895514
Electronic entropy	0.000000	0.000000
Rotational entropy	0.014374	0.015703
Vibrational entropy	0.010684	0.012744
Translational entropy	0.019646	0.021500
Entropy	0.044703	0.049947
Gibbs Energy	-934.941723	-934.945461

==================================

FINAL COORDINATES (Å)

==================================

Atom	X	Y	Z
S	-0.038241	0.079263	0.067010
O	-0.237992	-1.353079	-0.063740
O	-0.472924	0.914238	-1.036744
N	-0.685127	0.432880	1.481567
F	-0.484426	1.884890	1.606597
C	1.715153	0.356703	0.193152
C	2.469025	-0.502250	0.986762
C	3.833059	-0.291288	1.120493
C	4.440087	0.780492	0.469684
C	3.680484	1.637846	-0.315378
C	2.312321	1.425869	-0.457321
H	1.708226	2.082681	-1.070638
H	4.151189	2.473626	-0.822148
H	5.507149	0.945187	0.577114
H	4.425480	-0.961982	1.733732
H	1.984807	-1.329723	1.492747

F^-

===

THERMODYNAMIC PROPERTIES COMPARISON (all values in Hartree)

===

Property	T = 298 K	T = 323 K
Zero Point Energy	0.000000	0.000000
Inner Energy	-99.972643	-99.972643
Enthalpy	-99.971699	-99.971699
Electronic entropy	0.000000	0.000000
Rotational entropy	0.000000	0.000000
Vibrational entropy	0.000000	0.000000
Translational entropy	0.016510	0.016510
Entropy	0.016510	0.016510
Gibbs Energy	-99.988209	-99.988209

================================

FINAL COORDINATES

================================

Atom	X	Y	Z
F	0.000000	0.000000	0.000000

M03

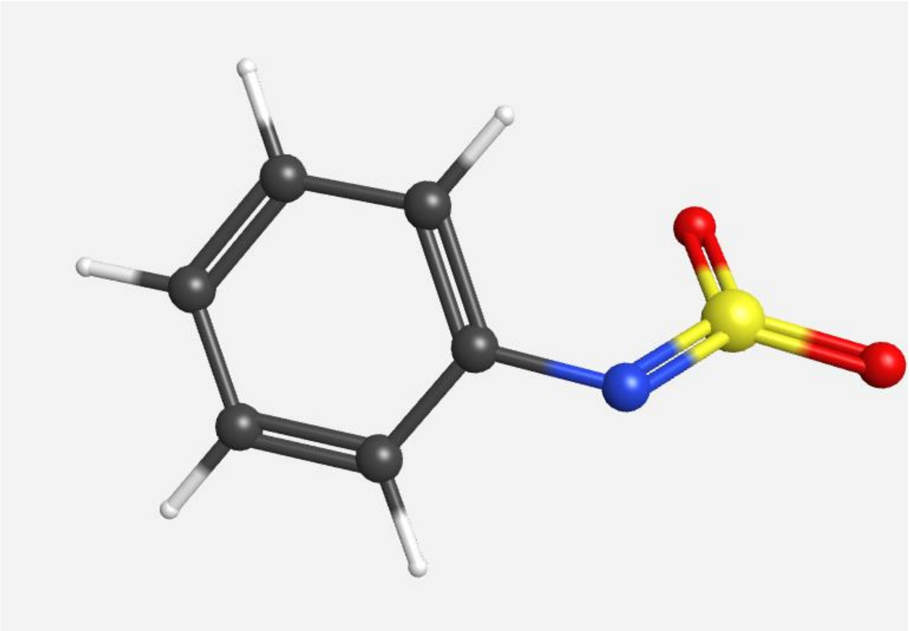

==

THERMODYNAMIC PROPERTIES (all values in Hartree)

==

Property	T = 298 K	T = 323 K
Zero Point Energy	0.105982	0.105982
Inner Energy	-834.935087	-834.933850
Enthalpy	-834.934143	-834.932827
Electronic entropy	0.000000	0.000000
Rotational entropy	0.014136	0.015446
Vibrational entropy	0.008821	0.010533
Translational entropy	0.019482	0.021323
Entropy	0.042440	0.047302
Gibbs Energy	-834.976583	-834.980129

======================================

FINAL COORDINATES (Å)

======================================

Atom	X	Y	Z
S	2.728335	0.458962	-0.390005
O	3.563745	1.552238	-0.769620
O	3.137273	-0.414595	0.657697
N	1.414265	0.171745	-1.025113
C	0.883705	0.987903	-2.073220
C	0.463611	0.342427	-3.230476
C	-0.107586	1.083850	-4.255979
C	-0.268415	2.458584	-4.121710
C	0.143887	3.091867	-2.954830
C	0.722037	2.360684	-1.925341
H	1.038613	2.851757	-1.012243
H	0.015182	4.162687	-2.840921
H	-0.718313	3.034543	-4.922835
H	-0.431409	0.582585	-5.161554
H	0.587704	-0.730884	-3.317618

R (M01+M03)

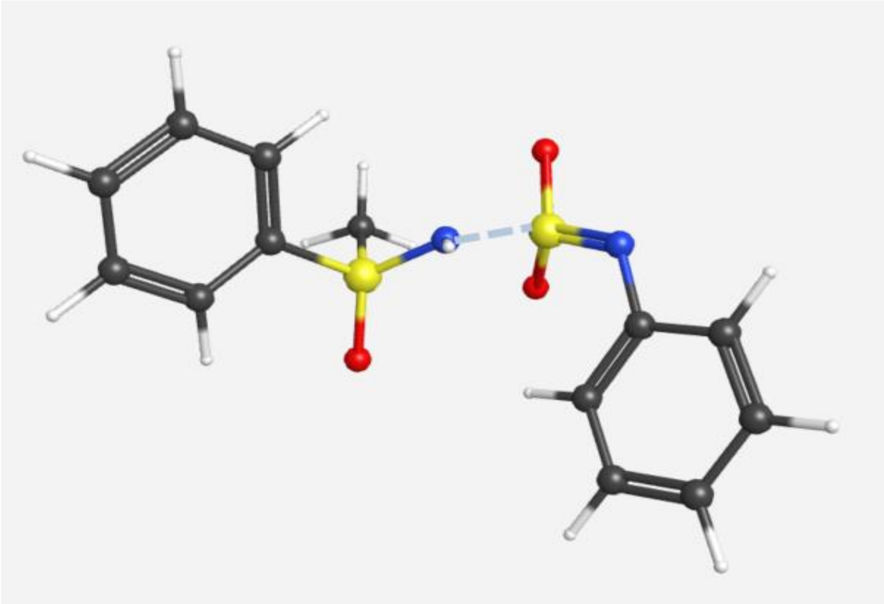

==

THERMODYNAMIC PROPERTIES (all values in Hartree)

===

Property	T = 298 K	T = 323 K
Zero Point Energy	0.260554	0.260554
Inner Energy	-1635.148903	-1635.146009
Enthalpy	-1635.147960	-1635.144986
Electronic entropy	0.000000	0.000000
Rotational entropy	0.016327	0.017820
Vibrational entropy	0.026487	0.031241
Translational entropy	0.020464	0.022387
Entropy	0.063278	0.071448
Gibbs Energy	-1635.211237	-1635.216434

==

FINAL COORDINATES (Å)

==

Atom	X	Y	Z
C	2.373453	0.721469	1.929073
S	0.743800	0.626170	1.280773
O	0.202011	1.936590	1.047828
N	0.854434	-0.384592	0.062217
S	2.099977	-0.267508	-1.229853
O	2.469091	1.127503	-1.145661
O	3.091813	-1.204385	-0.777925
N	1.362844	-0.767235	-2.454992
C	0.360888	-0.032112	-3.115390
C	-0.118819	-0.586092	-4.309047
C	-1.125289	0.035176	-5.031245
C	-1.686940	1.225764	-4.577825
C	-1.219530	1.779883	-3.393809
C	-0.206097	1.166083	-2.664791
H	0.144972	1.636267	-1.756262
H	-1.643665	2.708705	-3.025128
H	-2.475328	1.713051	-5.141093
H	-1.474945	-0.415269	-5.954814
H	0.317406	-1.516320	-4.657094
H	-0.021574	-0.809899	-0.223456
C	-0.225657	-0.245600	2.451997
C	-1.207255	0.468374	3.125503
C	-1.967421	-0.195593	4.078652
C	-1.732074	-1.539436	4.342678
C	-0.739538	-2.236320	3.658031
C	0.025628	-1.591532	2.700337

H	0.796324	-2.122323	2.154083
H	-0.564336	-3.284889	3.868164
H	-2.328840	-2.052327	5.088912
H	-2.743677	0.339199	4.613245
H	-1.373560	1.514868	2.903184
H	2.290865	1.300336	2.849010
H	2.738783	-0.286919	2.115679
H	2.982239	1.238923	1.189027

TS (M01+M03)

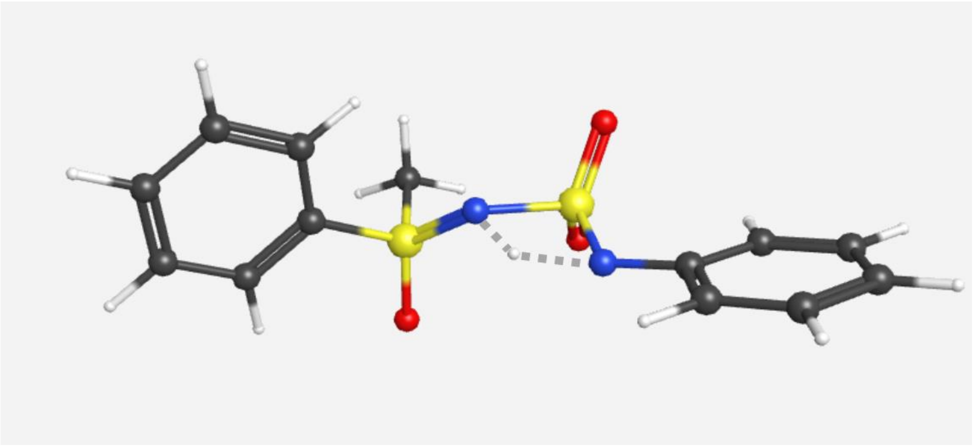

==

THERMODYNAMIC PROPERTIES (all values in Hartree)

==

Property	T = 298 K	T = 323 K
Zero Point Energy	0.255605	0.255605
Inner Energy	-1635.114007	-1635.111173
Enthalpy	-1635.113063	-1635.110150
Electronic entropy	0.000000	0.000000

Rotational entropy	0.016383	0.017881
Vibrational entropy	0.025918	0.030549
Translational entropy	0.020464	0.022387
Entropy	0.062765	0.070817
Gibbs Energy	-1635.175828	-1635.180967

==============================

FINAL COORDINATES (Å)

==============================

Atom	X	Y	Z
C	2.106227	1.020147	2.365200
S	0.433792	0.809386	1.856758
O	-0.305283	2.031394	2.033032
N	0.484131	0.138653	0.428303
S	1.271773	0.884892	-0.898307
O	1.679684	2.223305	-0.545823
O	2.323090	0.025792	-1.373994
N	-0.124468	0.739709	-1.645576
C	-0.261868	0.661572	-3.036180
C	-1.411064	0.051306	-3.546694
C	-1.612322	-0.031137	-4.916573
C	-0.672221	0.485536	-5.803074
C	0.469986	1.094083	-5.296658
C	0.677072	1.190641	-3.926361
H	1.566403	1.686324	-3.552294
H	1.210714	1.507827	-5.973406
H	-0.830094	0.417411	-6.873728
H	-2.511210	-0.507862	-5.294351
H	-2.141195	-0.355093	-2.854814

H	-0.428258	0.160123	-0.420716
C	-0.235773	-0.465566	2.856656
C	-1.108268	-0.086964	3.867784
C	-1.628172	-1.072826	4.695629
C	-1.267680	-2.401352	4.505198
C	-0.388261	-2.759824	3.486947
C	0.137068	-1.789137	2.649188
H	0.814874	-2.051552	1.846398
H	-0.115005	-3.798579	3.342210
H	-1.677163	-3.167512	5.154399
H	-2.316094	-0.800694	5.487601
H	-1.379295	0.953480	3.996415
H	2.073925	1.355982	3.401594
H	2.624626	0.067447	2.266298
H	2.540329	1.783087	1.718935

P (M01 + M03)

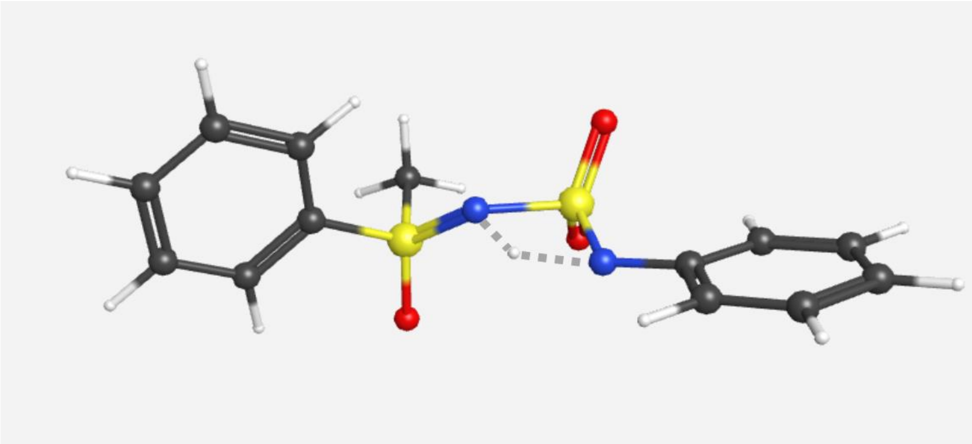

===

THERMODYNAMIC PROPERTIES (all values in Hartree)

===

Property	T = 298 K	T = 323 K
Zero Point Energy	0.255605	0.255605
Inner Energy	-1635.114007	-1635.111173
Enthalpy	-1635.113063	-1635.110150
Electronic entropy	0.000000	0.000000
Rotational entropy	0.016383	0.017881
Vibrational entropy	0.025918	0.030549
Translational entropy	0.020464	0.022387
Entropy	0.062765	0.070817
Gibbs Energy	-1635.175828	-1635.180967

==============================

FINAL COORDINATES (Å)

==============================

Atom	X	Y	Z
C	2.106227	1.020147	2.365200
S	0.433792	0.809386	1.856758
O	-0.305283	2.031394	2.033032
N	0.484131	0.138653	0.428303
S	1.271773	0.884892	-0.898307
O	1.679684	2.223305	-0.545823
O	2.323090	0.025792	-1.373994
N	-0.124468	0.739709	-1.645576
C	-0.261868	0.661572	-3.036180
C	-1.411064	0.051306	-3.546694
C	-1.612322	-0.031137	-4.916573
C	-0.672221	0.485536	-5.803074
C	0.469986	1.094083	-5.296658
C	0.677072	1.190641	-3.926361
H	1.566403	1.686324	-3.552294
H	1.210714	1.507827	-5.973406
H	-0.830094	0.417411	-6.873728
H	-2.511210	-0.507862	-5.294351
H	-2.141195	-0.355093	-2.854814
H	-0.428258	0.160123	-0.420716
C	-0.235773	-0.465566	2.856656
C	-1.108268	-0.086964	3.867784
C	-1.628172	-1.072826	4.695629
C	-1.267680	-2.401352	4.505198
C	-0.388261	-2.759824	3.486947
C	0.137068	-1.789137	2.649188

H	0.814874	-2.051552	1.846398
H	-0.115005	-3.798579	3.342210
H	-1.677163	-3.167512	5.154399
H	-2.316094	-0.800694	5.487601
H	-1.379295	0.953480	3.996415
H	2.073925	1.355982	3.401594
H	2.624626	0.067447	2.266298
H	2.540329	1.783087	1.718935

NITRENE INTERMEDIATE

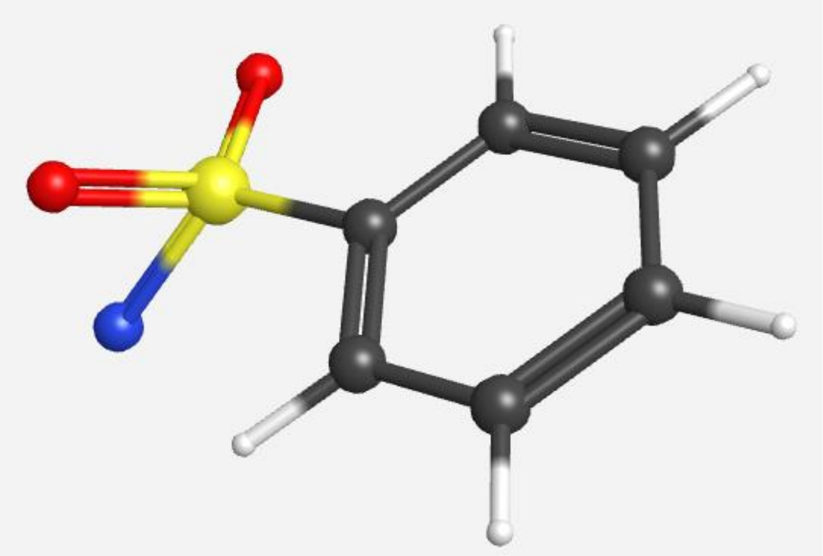

==

THERMODYNAMIC PROPERTIES COMPARISON (all values in Hartree)

==

Property	T = 298 K	T = 323 K
Zero Point Energy	0.104583	0.104583
Inner Energy	-834.834726	-834.833456
Enthalpy	-834.833782	-834.832433
Electronic entropy	0.000000	0.000000
Rotational entropy	0.014039	0.015340

Vibrational entropy	0.008581	0.010329
Translational entropy	0.019482	0.021323
Entropy	0.042102	0.046993
Gibbs Energy	-834.875884	-834.879425

==

FINAL COORDINATES (Å)

==

Coordinates from: nitren-opt_298.out

Atom	X	Y	Z
S	-0.054130	0.130515	0.155650
N	0.645105	-1.030966	0.893785
O	0.395685	1.481330	0.096525
O	0.490635	-0.916374	-0.806965
C	-1.795102	0.090211	0.148078
C	-2.504296	1.275485	-0.001970
C	-3.891104	1.218366	-0.012691
C	-4.537460	-0.004467	0.124550
C	-3.809005	-1.182167	0.276506
C	-2.425081	-1.142923	0.292791
H	-1.838090	-2.044444	0.418937
H	-4.321643	-2.130647	0.386597
H	-5.621331	-0.042379	0.115949
H	-4.465136	2.130284	-0.128471
H	-1.981247	2.217076	-0.107971

Rearrangement M02 -> M03

Here is a summary of the calculated Gibbs Free Energy change (ΔG) for the rearrangement of species **M02**. The analysis is based on computational data from DFT calculations (B3LYP-D4/def2-TZVP) with a CPCM solvent model for ethyl acetate.

The rearrangement process under consideration is:

M02 -> M03 + F^-

The calculated Gibbs Free Energy changes (ΔG_r) confirm the thermodynamic favorability of this transformation at elevated temperatures.

- **ΔG at 323 K (50 °C): -60.05 kJ/mol**

The significant negative ΔG values at 323 K indicate that the rearrangement is a highly exergonic and spontaneous process. The reaction's spontaneity is maintained at the elevated experimental temperature, confirming it is a thermodynamically viable pathway.

Reaction Profile Calculations

The reaction profile for the N-functionalization of *S*-methyl-*S*-phenylsulfoximine was recalculated using a more rigorous computational protocol (wB97X-D4/def2-TZVP with the CPCM solvent model for ethyl acetate). This updated analysis confirms that the reaction is kinetically feasible at 50 °C (323 K), despite a significant energy barrier.

The Gibbs Free Energy (ΔG) for each step in the reaction M01 + M03 ⟶ R ⟶ TS ⟶ P was calculated using the provided thermodynamic data. The energy of the separated reactants (M01 + M03) was set as the zero-point reference for each temperature. The results are summarized below.

Reaction Step	ΔG at 323 K (kJ/mol)
**M01 + M03** (Reactants)	0.00
**R** (Pre-reaction Complex)	-15.61
**TS** (Transition State)	+77.51
**P** (Product)	-104.28

Based on these calculations:

- The **activation energy** from the stable pre-reaction complex to the transition state is **+93.12 kJ/mol** at 323 K.
- The **overall reaction** is highly exergonic **104.28 kJ/mol** at 323 K.

References

1. Sheldon, A. R. The E factor at 30: a passion for pollution prevention. *Green Chem.* **2023**, *25*, 1704–1728.
2. Nelson, C. B.; L'Heureux, J. S.; Wong, J. M.; Kuhn, L. S.; Ghiglietti, E.; Lipshutz, H. B. Environmentally friendly Miyaura Borylations allowing for green, 1-pot borylation/Suzuki–Miyaura couplings. *Green Chem.* **2024**, *26*, 10115–10122.
3. Van Aken, K.; Strekowski, L.; Patiny, L. EcoScale, a semi-quantitative tool to select an organic preparation based on economical and ecological parameters. *Beilstein J. Org. Chem.* **2006**, *2*, 3–17.
4. CrysAlisPro (Version 1.171.39.46e), Rigaku Oxford Diffraction, Yarnton, UK, **2018**.
5. Sheldrick, G.M. Crystal structure refinement with SHELXL. *Acta Cryst. A.* **2015**, *71*, 3–8.
6. Dolomanov, O. V.; Bourhis, L. J.; Gildea, R. J.; Howard J. A. K.; Puschmann H. OLEX2: a complete structure solution, refinement and analysis program. *J. Appl. Cryst.* **2009**, *42*, 339–341.
7. Macrae, C. F.; Bruno I. J.; Chisholm, J. A.; Edgington, P. R.; McCabe, P.; Pidcock, E.; Rodriguez-Monge, L.; Taylor, R.; van de Streek, J.; Wood, P. A. Mercury CSD 2.0 – new features for the visualization and investigation of crystal structures. *J. Appl. Cryst.* **2008**, *41*, 466-470.
8. Neese, F. The ORCA program system. *WIREs Comput. Mol. Sci.* **2012**, *2*, 73–78.
9. Chai, J. -D.; Head-Gordon, M. Systematic optimization of long-range corrected hybrid density functionals. *J. Chem. Phys.* **2008**, *128*, 084106.
10. Caldeweyher, E.; Ehlert, S.; Hansen, A.; Neugebauer, H.; Spicher, S.; Bannwarth, C.; Grimme, S. A generally applicable atomic-charge dependent London dispersion correction. *J. Chem. Phys.* **2019**, *150*, 154122.
11. Weigend, F.; Ahlrichs, R. Balanced basis sets of split valence, triple zeta valence and quadruple zeta valence quality for H to Rn: Design and assessment of accuracy. *Phys. Chem. Chem. Phys.* **2005**, *7*, 3297–3305.
12. Henkelman, G.; Uberuaga, B. P.; Jónsson, H. A climbing image nudged elastic band method for finding saddle points and minimum energy paths. *J. Chem. Phys.* **2000**, *113*, 9901–9904.
13. Cossi, M.; Scalmani, G.; Rega, N.; Barone, V. Energies, structures, and electronic properties of molecules in solution with the C-PCM solvation model. *J. Comput. Chem.* **2003**, *24*, 669–681.
14. Weigend, F. Accurate Coulomb-fitting basis sets for H to Rn. *Phys. Chem. Chem. Phys.* **2006**, *8*, 1057–1065.
15. Odell, A.; Delin, A.; Johansson, B.; Bock, N.; Challacombe, M.; Niklasson, M. N. A. Higher-order symplectic integration in Born–Oppenheimer molecular dynamics. *J. Chem. Phys.* **2009**, *131*, 244106.
